# Recyclable heterogeneous metal foil-catalyzed cyclopropenation of alkynes and diazoacetates under solvent-free mechanochemical reaction conditions†
†Electronic supplementary information (ESI) available. See DOI: 10.1039/c8sc00443a


**DOI:** 10.1039/c8sc00443a

**Published:** 2018-04-24

**Authors:** Longrui Chen, Devonna Leslie, Michael G. Coleman, James Mack

**Affiliations:** a Department of Chemistry , University of Cincinnati , Cincinnati , Ohio 45221-0037 , USA . Email: james.mack@uc.edu; b School of Chemistry and Materials Science , Rochester Institute of Technology , Rochester , New York 14623-5604 , USA . Email: mgcsch@rit.edu

## Abstract

Silver and copper foil were found to be effective, versatile and selective heterogeneous catalysts for the cyclopropenation of terminal and internal alkynes under mechanochemical reaction conditions. This methodology enables the functionalization of a wide range of terminal or internal alkynes under ambient, aerobic, and solvent-free conditions. Finally, we have demonstrated a unique and versatile one-pot domino Sonogashira-cyclopropenation mechanochemical reaction for the formation of complex cyclopropenes.

## Abstract 2

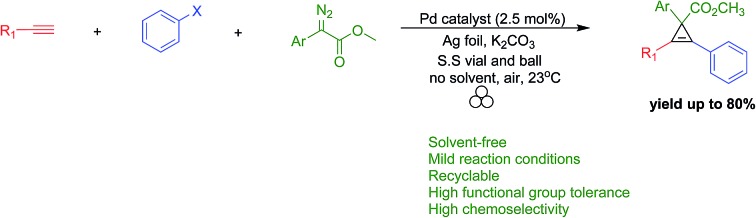
Silver and copper foil were found to be effective, versatile and selective heterogeneous catalysts for the cyclopropenation of terminal and internal alkynes under mechanochemical reaction conditions.

## Introduction

**Scheme 1:**
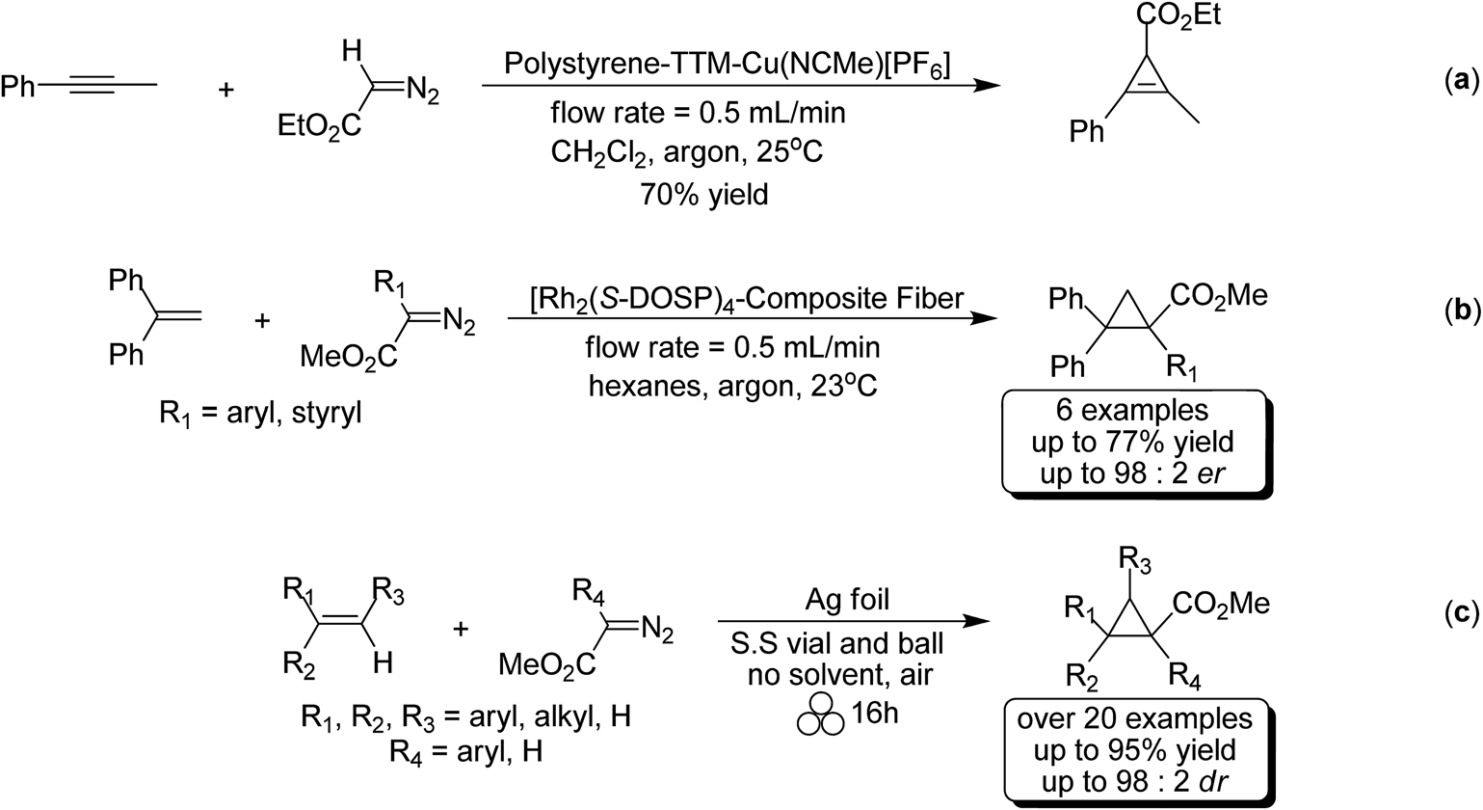
Heterogeneous transition metal-catalyzed [2 + 1] cycloaddition reactions.

The study of cyclopropenyl-containing compounds has been an active area of research for several decades due to its high ring strain.^1^ A wide variety of chemical and physical transformations that are not known for other π systems can be accessed by the release of the resultant potential energy.^2^–^8^ In fact, the reactivity of the cyclopropenyl moiety has been harnessed *via* a rapid and selective inverse demand Diels–Alder reaction with a tetrazine fluorescent tag for bioorthogonal chemical reporting/imaging.^9^–^17^ Despite their broad molecular utility, the harsh reaction conditions, poor atom economy, non-recoverable transition metal catalysts, large volumes of solvent required and slow syringe pump additions used in traditional synthetic methods severely limits the accessibility of cyclopropenyl compounds for study.^2^,^18^–^23^ In order to circumvent these synthetic impediments, the conversion of homogenous transition metal-catalyzed [2 + 1] cycloadditions of unsaturated hydrocarbons and diazoacetates into heterogeneous catalytic processes has been the prevailing research strategy for several decades. Towards that goal, laboratory scale continuous flow reactors have been developed for the synthesis of cyclopropene and cyclopropane compounds *via* a stationary bed of immobilized catalysts.^24^–^31^ Two impressive examples depicted here are as efficient and selective as their homogeneous-catalyzed counterparts [Scheme 1(a) and (b)].^25^,^26^ Batch reactor processes have also been extensively studied for the heterogeneous cyclopropanation of olefins and many forms of immobilized catalysts have been developed.^32^–^70^ Appreciating the technical, environmental and economic challenges associated with the homogenous transition metal-catalyzed [2 + 1] cycloadditions of unsaturated hydrocarbons and diazoacetates, our research group recently developed a solvent-free mechanochemical ball milling reaction using a highly selective and active silver foil catalyst in a stainless steel (S.S) vial [Scheme 1(c)].^71^ A highlight of this batch reactor design is that the silver-foil catalyst is recoverable, recyclable, shelf stable and commercially available. Moreover, this batch reactor design is a general and experimentally straightforward operation. No additional precautions such as inert atmospheric (Ar or N_2_), anhydrous reaction conditions, or freshly distilled reagents are necessary to preserve the catalytic activity. We observed that the silver foil can be recycled at least twenty times without any appreciable drop in yield or diastereoselectivity. Finally, forgoing the long syringe pump additions of diazoacetate (∼0.1 M) diluted in large volumes of solvent is a particularly attractive ‘green’ feature attributed to this solvent-free mechanochemical cyclopropanation reaction.

Mechanochemistry is a ‘chemical reaction that is induced by the direct absorption of mechanical energy.’^72^ While it is unlikely that the solvent-free mechanochemical laboratory procedure comes to the mind of contemporary synthetic chemists as a useful and simple experimental procedure, it is one of the earliest recorded chemical methods known to convert physical energy, namely heat and pressure, into chemical energy.^73^,^74^ This ancient synthetic method has been extensively used over the past century for the formation of new macromolecular products, metal–organic frameworks, compositions and cocrystals by grinding, extrusive and milling forces from poorly soluble solid–solid, solid–liquid and liquid–liquid inorganic reactants.^75^–^91^ Mechanochemical methods have also been utilized for the dehalogenation of pollutants that are resistant to degradation through chemical, biological, and photolytic processes.^92^–^101^ In recent years, solvent-free mechanochemistry has emerged as a ‘green’ alternative to the unsustainable use of petroleum-based solvents in classic organic chemistry transformations.^102^–^106^ With respect to fine chemical manufacturing, it is also an effective experimental procedure, known as medicinal mechanochemistry, for the synthesis of many classes of organic compounds, active pharmaceutical ingredients (APIs), and commercially-available drugs.^75^,^89^,^105^–^113^

**Fig. 1:**
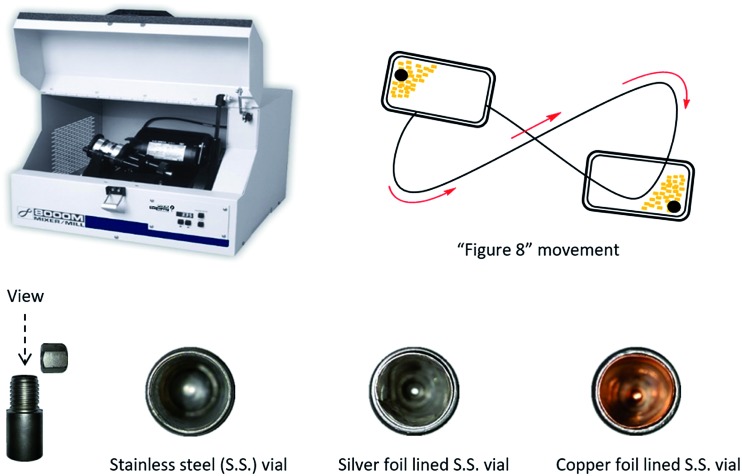
SPEX 8000M vibratory mixer/mill® and reaction vials.

Unlike solution-phase organic chemistry, solvent-free ball milling conditions are generally conducted without the need for inert gas, external heat, or solvent. The stainless-steel reactors vials are machined from stainless-steel rods and the organic/inorganic reactants are added in the open atmosphere with one or more stainless-steel balls. In our previous work, diazoacetate and an excess of the unsaturated hydrocarbon (trap) were added to a stainless steel vial lined with a silver foil catalyst and a single stainless steel ball. Similar to the analogous solution-phase cycloaddition reactions, the excess trap serves to suppress any undesired diazoacetate dimerization pathways and does not serve as a solvent in this mechanochemical process. This work demonstrated that solid–solid ball milling of a diazoacetate in the presence of excess alkene was as effective as liquid–liquid ball milling which suggested that efficient mixing of the reaction media was achieved. The silver foil lined reaction vial was mechanochemically ball milled using an SPEX 8000M vibratory mixer/mill® at 18 Hz to furnish cyclopropane compounds in high yields (Fig. 1).^71^ Moreover, the stainless-steel vials, metal foil, and excess unsaturated hydrocarbons were easily recovered and recycled for later use.

## Results

Encouraged by our previous results, we set out to test if our solvent-free mechanochemical batch reactor design could also be utilized for the highly selective and efficient [2 + 1] cycloadditions of unsaturated hydrocarbons by simply changing the catalytic foil insert. We began by measuring the catalytic efficiency of copper and silver foils for the cyclopropenation of terminal and internal acetylenes in a SPEX 8000M vibratory mixer/mill® at 18 Hz (Table 1) under various reaction conditions. After optimizing the ratio of reactants (1 : 5; diazo : alkyne, respectively) and mixing time (16 h), the copper foil-catalyzed cyclopropenation of phenyl acetylene afforded cyclopropene product **1** in 88% isolated yield (entry 1). However, when 1-phenyl-propyne was treated to identical mechanochemical conditions, a significantly lower yield of the corresponding cyclopropene product **2** was observed (entry 2). We then simply replaced the copper foil with silver foil and milled phenyl acetylene and methyl phenyldiazoacetate and did not observe any appreciable amount of cyclopropene product **1** (entry 3). Lastly, we turned our attention to 1-phenyl-propyne and methyl phenyldiazoacetate (1 eq.) under silver foil-catalyzed mechanochemical reaction conditions. To our delight, cyclopropene product **2** was isolated (90% yield) after 16 hours of milling (entry 4). These initial results are in agreement with previous work which demonstrated that silver(i) salts are effective homogeneous catalysts for the cyclopropenation of internal acetylenic compounds, while ineffective for terminal alkynes.^114^

**Table 1:** Heterogeneous metal-foil-catalyzed cyclopropenation of acetylenes under mechanochemical conditions

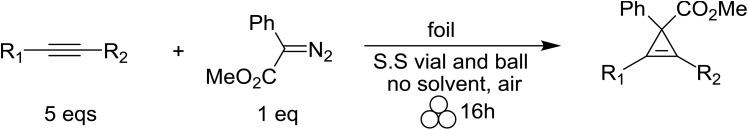					
Entry	Foil	R_1_	R_2_	Product	Isolated yield^*a*^ (%)
1	Cu	Ph	H	**1**	88
2	Cu	Ph	CH_3_	**2**	10
3	Ag	Ph	H	**1**	Trace
4	Ag	Ph	CH_3_	**2**	90
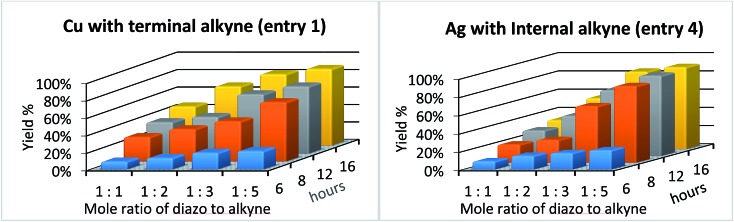					

^*a*^All reactions were performed on a 1 mmol scale of diazoacetate.

Having established the general boundaries for the catalytic activity and selectivity of metal foil-catalyzed mechanochemical cyclopropenation reactions, various terminal alkynes were investigated using copper foil (Table 2). Electron-rich and electron-poor terminal acetylenes were all obtained in good to excellent yields (entries 1–10). 1-Cyclohexenyl acetylene afforded the cyclopropene product **13** exclusively in good yield (entry 11). 3-Thiophenyl acetylene afforded the corresponding cyclopropene product **14** in 90% yield (entry 12). Remarkably, 4-ethynylaniline cleanly underwent the mechanochemical cyclopropenation in the presence of the unprotected aniline in excellent isolated yield (entry 13). To the best of our knowledge, this is the first instance where an unprotected N–H bond was chemically inert to carbene transfer and afforded cyclopropene **15** without additional protection and deprotection steps.^22^,^115^,^116^ Next, we surveyed a range of electronically diverse diazoacetate compounds and determined that dimethyl diazomalonate and ethyl diazoacetate compounds were ineffective for the copper foil-catalyzed cyclopropenation of phenyl acetylene under solvent-free mechanochemical milling conditions (entries 14–16).

**Table 2:** Copper foil-catalyzed mechanochemical cyclopropenation of terminal alkynes^*a*^

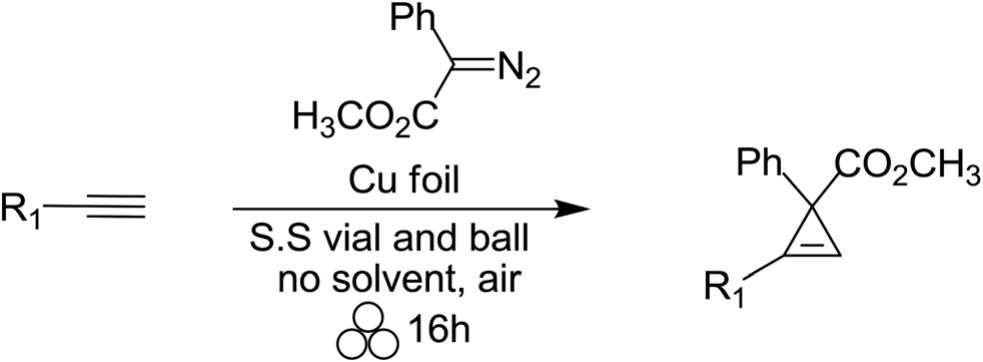			
Entry	R_1_	Product	Isolated yield (%)
1	4-(OCH_3_)Ph	**3**	88
2	4-(CH_3_)Ph	**4**	95
3	3-(CH_3_)Ph	**5**	88
4	4-(C(CH_3_)_3_)Ph	**6**	81
5	4-(F)Ph	**7**	85
6	4-(Br)Ph	**8**	85
7	2-(Cl)Ph	**9**	82
8	3-(Cl)Ph	**10**	84
9	2,4,6-(CH_3_)Ph	**11**	78
10	2-(CH_3_)-4-(OCH_3_)Ph	**12**	90
11	1-Cyclohexenyl	**13**	86
12	3-Thiophenyl	**14**	90
13	4-(NH_2_)Ph	**15**	94
14^*b*^	Ph	**16**	81
15^*c*^	Ph	**17**	Trace^*e*^
16^*d*^	Ph	**18**	Trace^*e*^

^*a*^Reation conditions: diazoacetate (1.00 eq.), alkyne (5.00 eq.), and copper foil added to a SPEX 8000M mixer/mill (18 Hz) for 16 h.

^*b*^1 eq. of methyl *p*-bromophenyldiazoacetate was used.

^*c*^1 eq. of ethyl diazoacetate was used.

^*d*^1 eq. of dimethyl diazomalonate was used.

^*e*^Recovered starting material.

Next, we investigated the scope of the silver foil-catalyzed mechanochemical cyclopropenation reaction with various internally substituted acetylenic compounds (Table 3). 1-Phenyl substituted aliphatic alkynes (entries 1 and 2) were isolated in excellent yields (>92%). Interestingly, 1-TMS-2-phenylacetylene underwent cyclopropenation in relatively low yield by GC analysis (entry 3). It is important to note that the analogous solution phase, homogeneous, Ag(i)-catalyzed reaction is completely unreactive.^114^ Increasing the steric bulk of the silyl group had a detrimental effect on the reactivity of the mechanochemical cyclopropenation methodology (entries 4 and 5), while diaryl-substituted acetylenes were efficiently converted into the cyclopropene products **24–26** in isolated yields up to 85% (entries 6–8). Various electron rich and electron poor aryl diazoacetates were also tested and all afforded excellent yields (>80%) of the corresponding cyclopropene products (entries 10–14). As stated earlier, the electronic character of the diazoacetate plays an important role in its reactivity with the transition metal catalyst, and ultimately, the efficiency of the carbene transfer.^117^,^118^ For example, homogeneous Ag(i) salts effectively catalyze an interesting C–Cl insertion with ethyl diazoacetate when using methylene halide or haloform as solvents.^114^,^119^ We discovered that eliminating dichloromethane as the commonly used solvent afforded the corresponding cyclopropene products **34** and **35**, in the presence of ethyl diazoacetate, under solvent-free silver-foil catalyzed mechanochemical reaction conditions (entries 16 and 17). Finally, diethyl diazomalonate was completely unreactive and only starting materials were recovered (entry 18). This finding is in agreement with previous work that stable metal–carbene complexes are observed in the solution phase reactions of diazomalonate compounds and silver(i) salts.^120^,^121^

**Table 3:** Silver foil-catalyzed mechanochemical cyclopropenation of internal alkynes^*a*^

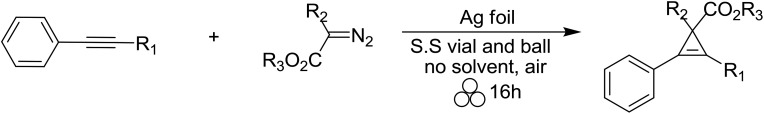					
Entry	R_1_	R_2_	R_3_	Product	Yield (%)^*b*^
1	CH_2_CH_3_	Ph	CH_3_	**19**	92
2	(CH_2_)_3_CH_3_	Ph	CH_3_	**20**	95
3	Si(CH_3_)_3_	Ph	CH_3_	**21**	20^*c*^
4	Si(CH(CH_2_))_3_	Ph	CH_3_	**22**	Trace^*e*^
5	Si(Ph)_3_	Ph	CH_3_	**23**	Trace^*e*^
6	Ph	Ph	CH_3_	**24**	84
7	4-(CH_3_)Ph	Ph	CH_3_	**25**	85
8	4-(Br)Ph	Ph	CH_3_	**26**	65
9	3-Thiophenyl	Ph	CH_3_	**27**	86
10	CH_3_	4-(OCH_3_)Ph	CH_3_	**28**	86
11	CH_3_	4-(C(CH_3_)_3_)Ph	CH_3_	**29**	84
12	CH_3_	4-BrPh	CH_3_	**30**	89
13	CH_3_	4-CF_3_Ph	CH_3_	**31**	88
14	(CH_2_)_3_CH_3_	4-BrPh	CH_3_	**32**	80
15	(CH_2_)_3_CH_3_	4-(CH_3_)Ph	CH_3_	**33**	79
16	CH_3_	H	CH_2_CH_3_	**34**	80^*c*^
17	(CH_2_)_3_CH_3_	H	CH_2_CH_3_	**35**	85^*c*^
18	(CH_2_)_3_CH_3_	CO_2_CH_3_	CH_3_	**36**	Trace^*e*^
19^*d*^		Ph	CH_3_	**37**	85

^*a*^Reation conditions: diazoatate (1.00 eq.), alkyne (5.00 eq,), and silver foil added to a SPEX 8000M mixer/mill (18 Hz) for 16 h.

^*b*^Isolated yields unless otherwise stated.

^*c*^GC yield.

^*d*^4-Octyne was used.

^*e*^Recovered starting material.

Having established the efficiency of this mechanochemical reaction, we set out to explore several intermolecular experiments to measure the chemoselectivity of the mechanochemical reaction using an equimolar mixture of olefins and acetylenes in the presence of methyl phenyldiazoacetate (Table 4). After 16 h of mechanochemical milling, we discovered that silver foil highly favors the cyclopropanation of olefins in the presence of terminal acetylenes (entry 1). These findings suggest that the silver foil does not lose its catalytic activity despite known silver acetylide catalyst deactivation pathways for the solution-phase, homogeneous silver-catalyzed counterpart.^114^ On the other hand, copper foil was mildly chemoselective and favored the cyclopropenation pathway for a mixture monosubstituted alkenes and terminal alkynes (entry 1). The silver foil-catalyzed [2 + 1] cycloaddition was observed to be less selective for 1,2-disubstituted alkenes over their internally substituted acetylenic counterparts (entries 2–5). While the copper foil-catalyzed [2 + 1] cycloaddition of 1,2-disubstituted alkenes and internal alkynes favored the cyclopropanation reaction pathway (entries 2–5). Finally, the mechanochemical milling of diphenyl-substituted olefins and diphenylacetylene resulted in a highly chemoselective ratio of cycloaddition products (entries 6 and 7), where silver foil favored cyclopropenation and copper foil favored cyclopropanation.

**Table 4:** Metal foil-catalyzed cycloaddition of unsaturated hydrocarbons with methyl phenyldiazoacetate^*a*^

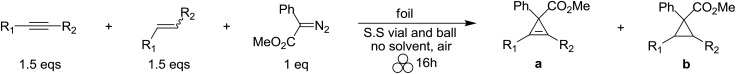						
Entry	R_1_	R_2_	Ag foil		Cu foil	
			Yield^*b*^ (%)	Product (**a** : **b**)^*c*^ ^,^^*d*^	Yield^*b*^ (%)	Product (**a** : **b**)^*c*^ ^,^^*d*^
1	Ph	H	91	**1** : **38** (2 : 98)	92	**1** : **38** (65 : 35)
2	*n*-Butyl	*n*-Butyl (*trans*)	80	**39** : **40** (75 : 25)	40	**39** : **40** (15 : 85)
3	*n*-Butyl	*n*-Butyl (*cis*)	83	**39** : **41** (70 : 30)	48	**39** : **41** (10 : 90)
4	Ph	CH_3_ (*trans*)	90	**2** : **42** (55 : 45)	86	**2** : **42** (15 : 85)
5	Ph	CH_3_ (*cis*)	90	**2** : **43** (55 : 45)	88	**2** : **43** (15 : 85)
6	Ph	Ph (*trans*)	80	**24** : **44** (95 : 5)	30	**24** : **44** (5 : 95)
7	Ph	Ph (*cis*)	82	**24** : **45** (90 : 10)	42	**24** : **45** (5 : 95)

^*a*^Reaction Conditions: diazoacetate (1.00 eq.), alkyne (1.50 eq.), alkyne (1.5 eq.) and metal foil added to a SPEX 8000M mixer/mill (18 Hz) for 16 h.

^*b*^GC yield.

^*c*^Determined by GC analysis.

^*d*^Diastereomeric ratios were not measured.

In order to further test the chemoselectivity of this mechanochemical [2 + 1] cycloaddition reaction, we extended this study to include compounds containing enyne moieties. The mechanochemical metal foil-catalyzed cycloaddition of enyne moieties afforded astonishingly chemoselective transformations (Table 5).

**Table 5:** Metal foil-catalyzed cycloaddition of enynes with methyl phenyldiazoacetate^*a*^

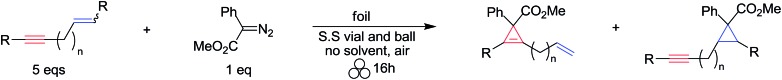					
Entry	Enyne	Ag foil		Cu foil	
		Product	Yield^*b*^ (%)	Product	Yield^*b*^ (%)
1	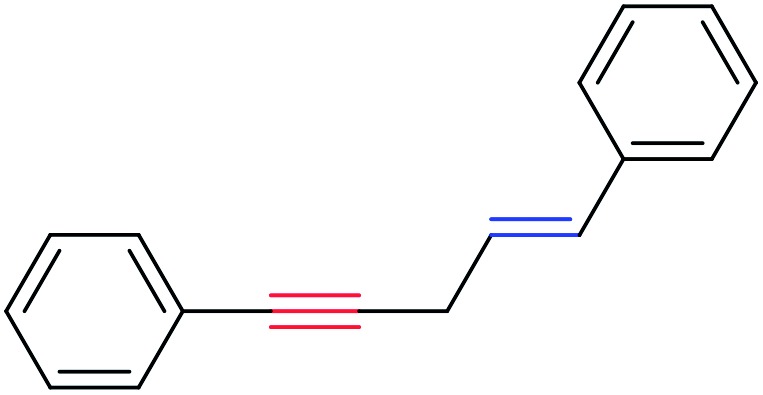	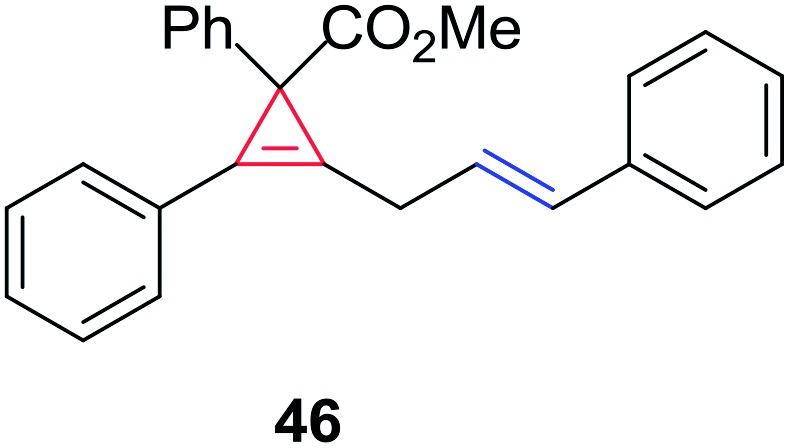	68	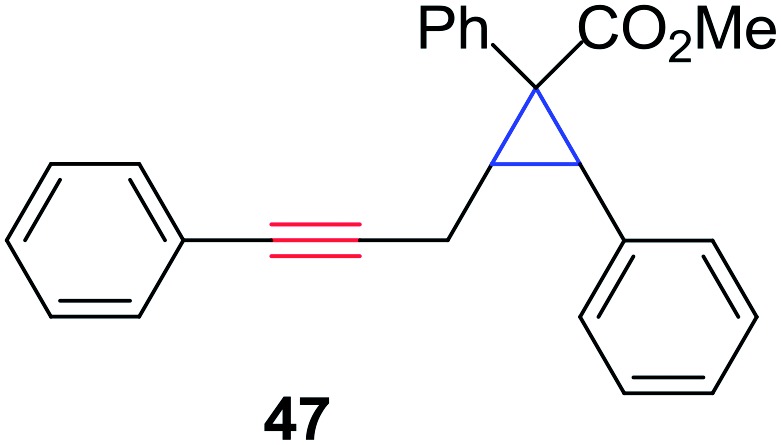	55
2	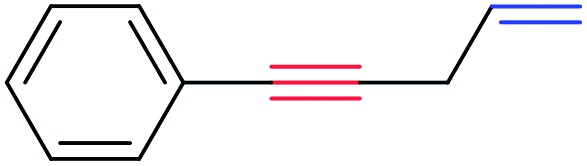	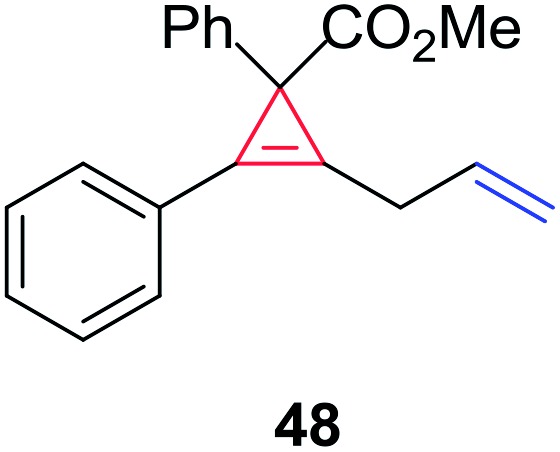	74	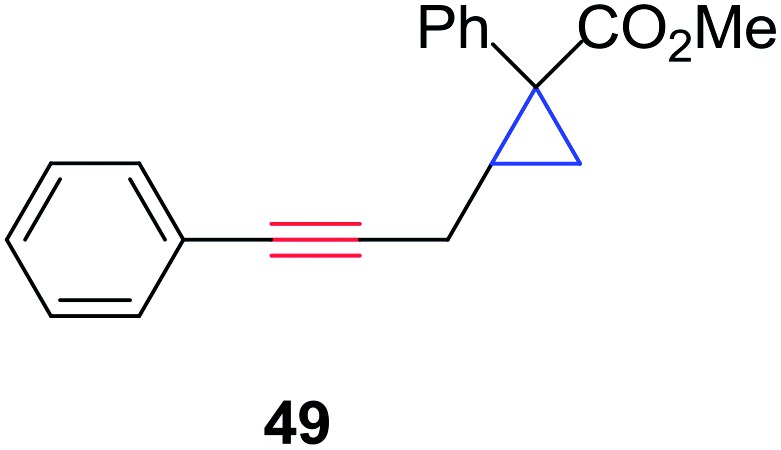	80
3	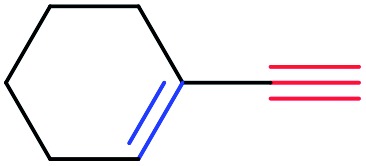	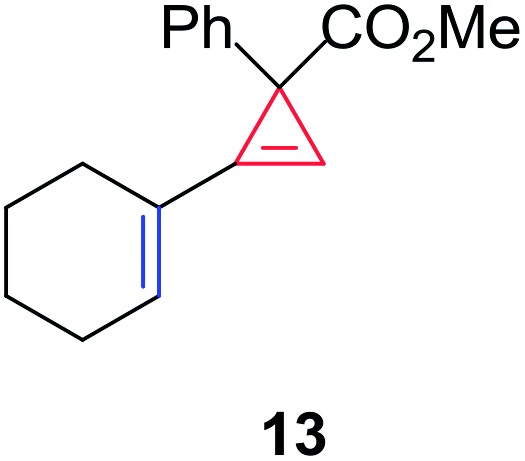	0^*c*^	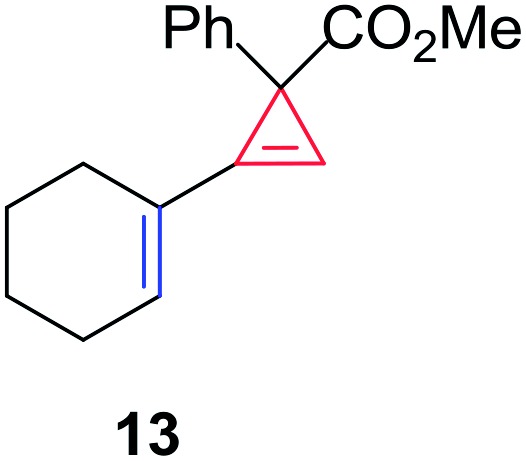	86
4	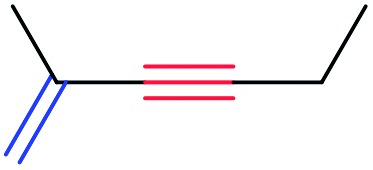	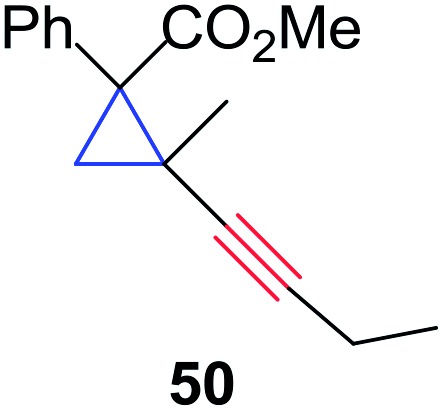	74	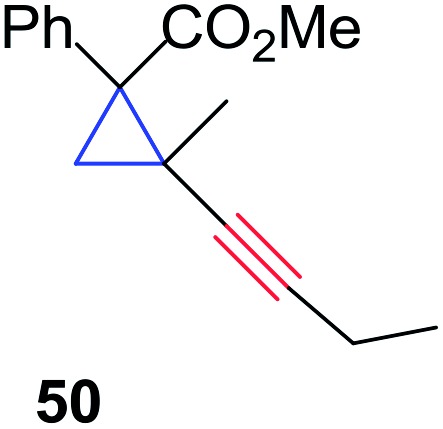	85

^*a*^Reaction conditions: diazoacetate (1.00 eq.), enyne (5.00 eq.) and copper foil added to a SPEX 8000M mixer/mill (18 Hz) for 16 h.

^*b*^GC yield.

^*c*^Recovered starting materials.

The silver foil-catalyzed process resulted in the cyclopropenation exclusively for internal alkynes (entries 1 and 2). After simply switching the silver to copper foil, we observed the exclusive formation of the corresponding cyclopropanation products (entries 1 and 2). Interestingly, 1-cyclohexenyl acetylene resulted in complete deactivation of the silver foil catalyst and no cycloaddition product was isolated (entry 3). This finding may suggest that the substrate was deactivated on the silver foil which is contrary to the intermolecular process observed earlier (Table 4, entry 1). Copper foil effectively and selectively converted the trisubstituted olefin into the cyclopropane product **13** in good yield (entry 3). However, the cyclopropanation of 1,1-disubstituted alkene moiety yielded that same cyclopropane product **50** for both silver and copper foil (entry 4). Overall, these highly chemoselective mechanochemical transformations gave rise to interesting building blocks that are useful for other organic transformations.

In addition to developing ‘solvent-free’ mechanochemical organic chemistry methods, we are also interested in realizing strategies for the construction of complex organic compounds from two or more synthetic steps in a single highly atom-economical ‘one-pot’ procedure. In recent years, several multicomponent coupling reactions have been developed by combining well-established individual reactions without the need for extraneous use of solvent in workups or purification steps.^122^–^128^ Based on the mechanochemical Sonogashira coupling reaction previously developed by our group,^129^ we envisioned a three-component coupling of a terminal alkyne, aryl halide and a diazoacetate compound as the first example of a transition-metal catalyzed domino mechanochemical reaction. At the outset, we were cognizant that the terminal alkynes were susceptible to cyclopropenation with copper foil, while silver(i) salts are inert to terminal alkynes but remain catalytically active. Previous work suggests that silver foil may be capable of serving as an effective catalyst^130^ and co-catalyst^131^–^138^ for the Sonogashira coupling reaction, and later, catalyzed the cyclopropenation of the newly formed internal alkynes in the presence of a diazoacetate compound. To test this hypothesis, we first examined the feasibility of the Pd(ii)/silver foil-catalyzed Sonogashira mechanochemical reaction. Gratifyingly, we observed good to excellent yields of the internal alkyne product by GC analysis (Table 6).

**Table 6:** Optimization of the Pd(ii)/silver foil-catalyzed mechanochemical Sonogashira reaction^*a*^

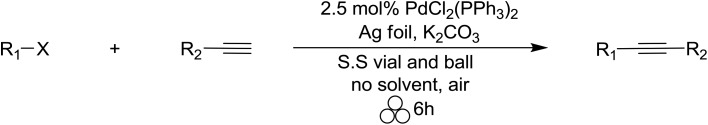					
Entry	X	R_1_	R_2_	Product	Yield^*b*^ (%)
1	I	4-(Cl)Ph	Ph	**51**	90
2	I	4-(Br)Ph	Ph	**52**	88
3	I	4-(F)Ph	Ph	**53**	87
4	Br	4-(F)Ph	Ph	**53**	80
5	I	4-(CH_2_CH_3_)Ph	Ph	**54**	93
6	Br	4-(CH_2_CH_3_)Ph	Ph	**54**	95
7	Br	4-(C(CH_3_)_3_)Ph	Ph	**55**	90
8	Br	4-(CH_3_)Ph	Ph	**56**	90
9	I	4-(CH_3_)Ph	Ph	**56**	95
10	I	4-(Cl)Ph	CH_2_CH_2_CH_2_CH_3_	**57**	92
11	I	4-(Br)Ph	CH_2_CH_2_CH_2_CH_3_	**58**	88
12	I	4-(F)Ph	CH_2_CH_2_CH_2_CH_3_	**59**	90
13	I	4-(CH_3_)Ph	CH_2_CH_2_CH_2_CH_3_	**60**	93
14	Br	4-(CH_3_)Ph	CH_2_CH_2_CH_2_CH_3_	**60**	93
15	Br	4-(C(CH_3_)_3_Ph	CH_2_CH_2_CH_2_CH_3_	**61**	93
16	I	Ph	4-(Cl)Ph	**51**	85
17	Br	Ph	4-(Cl)Ph	**51**	77
18	I	Ph	4-(Br)Ph	**52**	88
19	Br	Ph	4-(Br)Ph	**52**	80
20	I	Ph	4-(F)Ph	**53**	89
21	I	Ph	4-(CH_2_CH_3_)Ph	**54**	93
22	Br	Ph	4-(CH_2_CH_3_)Ph	**54**	86
23	I	Ph	4-(CH_3_)Ph	**56**	95
24	Br	Ph	4-(CH_3_)Ph	**56**	85
25	I	Ph	CH_2_CH_2_Ph	**62**	83
26	I	4-(Cl)Ph	CH_2_CH_2_Ph	**63**	80
27	Br	4-(CH_3_)Ph	CH_2_CH_2_Ph	**64**	85

^*a*^Reaction conditions: halide (1.05 mmol), alkyne (1.00 mmol), base (1.5 mmol), Pd catalyst and silver foil added to a SPEX 8000M mixer/mill (18 Hz) for h.

^*b*^GC yields.

Our initial investigations of the cross coupling product of aryl iodides and bromides and phenyl acetylene afforded excellent yields by GC analysis (80–95%) (entries 1–9). Similar to our original report of the Sonogashira reaction under mechanochemical conditions, the reaction is much faster with iodine than bromine and is virtually unreactive with chlorine.^129^ Such that entries 2 and 11 only provided coupling across the carbon iodide bond. 1-Hexyne proved to be an equally effective cross coupling partner with aryl iodides or bromides (entries 10–15). A diverse range of *p*-substituted arylacetylene compounds (**51–56**) were observed by GC analysis from either iodobenzene or bromobenzene starting materials in good yields (>77%). No diyne byproducts resulting from homo coupling or multiple Sonogashira couplings were detected. *Para*-substituted aryl iodides and bromides were easily coupled to 4-phenyl-1-butyne to afford the corresponding Sonogashira products (**62–64**, entries 25–27) in good yield (>78%) by GC analysis.

Given the fact that silver foil is an effective co-catalyst for the cross coupling of aryl halides and terminal acetylenic compounds *via* mechanochemical milling, we then turned our attention back towards our initial hypothesis that a domino one-pot Sonogashira coupling followed by cyclopropenation would be a straightforward and general method for the synthesis of a diverse library of fully substituted cyclopropene compounds (Table 7).

**Table 7:** Optimization of the one-pot Sonogashira coupling/cyclopropenation mechanochemical reaction^*a*^

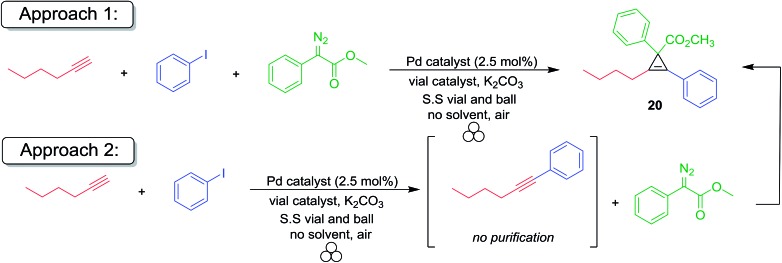					
Entry	Palladium	Vial catalyst^*b*^	Approach	Reaction time (h)	Yield^*c*^ %
1	None	Stainless steel vial	1	16	0^*d*^
2	PdCl_2_(PPh_3_)_2_	Stainless steel vial	1	16	0^*e*^
3	Pd(PPh_3_)_4_^*g*^	Stainless steel vial	1	16	0^*e*^
4	PdCl_2_(PPh_3_)_2_	Nickel vial	1	16	0^*d*^
5	PdCl_2_(PPh_3_)_2_	Ag foil (S.S.)	1	16	40
6	Pd(OAc)_2_	Ag foil (S.S.)	1	16	Trace
7	Pd(PPh_3_)_4_^*g*^	Ag foil (S.S.)	1	16	38
8	Pd(dba)_2_	Ag foil (S.S.)	1	16	10
9	PdCl_2_(PPh_3_)_2_	Cu foil (S.S.)	1	16	0^*f*^
10	Pd(OAc)_2_	Ag foil (S.S.)	2	6 + 16	Trace
11	Pd(PPh_3_)_4_^*g*^	Ag foil (S.S.)	2	6 + 16	75
12	Pd(dba)_2_	Ag foil (S.S.)	2	6 + 16	60
13	PdCl_2_(PPh_3_)_2_	Ag foil (S.S.)	2	6 + 16	76
14	PdCl_2_(PPh_3_)_2_	Ag foil (S.S.)	2	6 + 12	75
15	PdCl_2_(PPh_3_)_2_	Ag foil (S.S.)	2	6 + 10	72
16	**PdCl**_**2**_ **(PPh**_**3**_ **)**_**2**_	**Ag foil (S.S.)**	2	**6 + 8**	**72**
17	PdCl_2_(PPh_3_)_2_	Cu foil (S.S.)	2	16 + 16	0^*f*^

^*a*^Reactions conditions: diazoacetate (1 eq.), aryl halide (3 eq.), K_2_CO_3_ (3.5 eq.) and Pd (0.026) catalyst added in a custom made reaction vial.

^*b*^Lined metal foil in vials as active catalyst during grinding.

^*c*^All yields are the average of isolated yield of two experiments.

^*d*^Recovered only starting material and small amount of dimer of diazo.

^*e*^Only Sonogashira-coupling product indicated.

^*f*^Mostly alkyne homo-coupling product and small amount of cyclopropenation product with terminal alkyne was observed.

^*g*^Conducted in inert atmosphere.

Since the predictive models of mechanochemical catalysis are still in their infancy, we used surveyed several reaction conditions and experimental procedures to determine which were optimal. First, we attempted a one-pot domino reaction where methyl phenyl diazoacetate, phenylacetylene, iodobenzene, 5% palladium tetrakis triphenylphosphine and potassium carbonate were all added to a stainless steel reactor and ball milled for 16 h (approach 1). In the absence of silver foil, no product was observed irrespective of the reaction conditions (entries 1–4, 9, and 17). However, upon the addition of silver foil, the cross coupled/cyclopropenation product was isolated albeit in modest yield (entries 5, 7 and 8). Catalytic amounts of palladium(ii) acetate were found to be completely unreactive towards Sonogashira coupling by mechanochemical ball milling (entries 6 and 10). Copper foil was observed to be effective for the Sonogashira coupling reaction (approach 1, entry 9) but failed to yield any appreciable amount of the cyclopropene product. In an effort to increase the efficiency of the desired process, we elected to add the diazoacetate compound after ball milling the Sonogashira reactants for 6 hours without purification. This was then followed by additional ball milling of the solvent-free reaction mixture (approach 2). Under traditional solution based conditions both the Sonogashira and cyclopropenation reactions are typically conducted under strictly inert atmospheric reaction conditions. To test this, we conducted the reaction using Pd(PPh_3_)_4_ under inert conditions using a dry box (entries 7 and 11) and compared the reaction using PdCl_2_(PPh_3_)_2_ under aerobic conditions (entries 5 and 13). In our previous report, we demonstrated both catalysts gave rise to similar yields of Sonogashira products.^129^ Since both reactions under approach 1 and approach 2 gave similar reaction yields, we concluded there is no preference for inert atmospheric mechanochemical ball milling reaction conditions. After optimization of the milling time and palladium salt, the two-step reaction resulted in good yields (60–75%) of the desired fully substituted cyclopropene product (entries 10–16). To the best of our knowledge, this represents the first example of a catalytic one-pot multi-component coupling/cycloaddition domino methodology for the organometallic synthesis of highly functionalized organic compounds. Lastly, copper foil was an effective co-catalyst for only the Sonogashira coupling reaction and no cyclopropenation of the internal alkyne was observed (entry 17).

To survey the general nature for the ‘one-pot’ Pd/Ag foil-catalyzed cross coupling/cyclopropenation mechanochemical milling reaction, we studied a variety of alkynes, aryl halides and aryl diazoacetates (Table 8).

**Table 8:** One-pot domino Sonogashira coupling/cyclopropenation mechanochemical reaction using approach 2

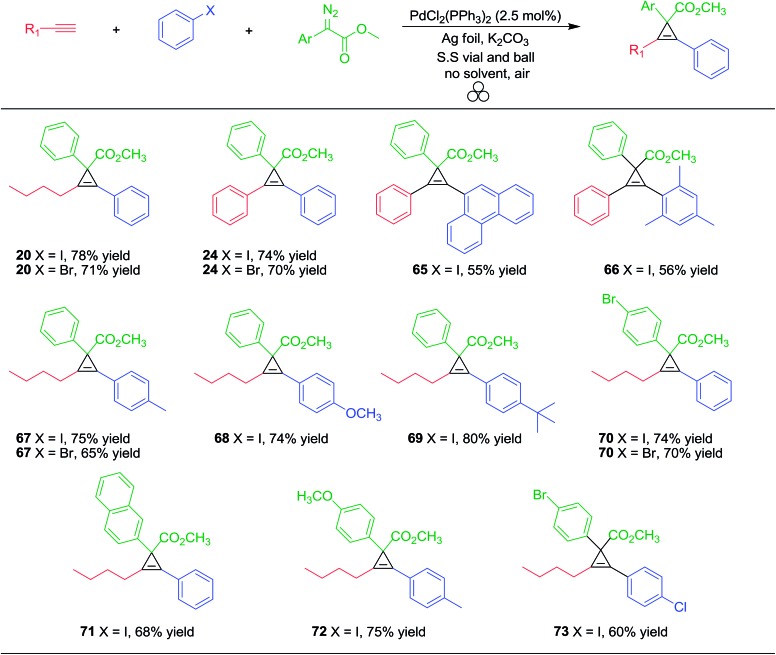

Aliphatic and aryl alkynes coupled well with aryl iodides or bromides to afford internal alkynes, which upon treatment with aryl diazoacetate, effectively produced the cyclopropene products **20**, **24**, **65–69** in good overall yields (55–80%). In order to test the reactivity of electron rich and electron poor aryl diazoacetates for cyclopropenation, we subjected the coupling products of 1-hexyne and various aryl halides to yield cyclopropene products (**70–73**) in good overall yields. Compounds **70** and **73** did not produce any Sonogashira type reactivity at the aryl bromide site, presumably due the significant effect substituents have on the rate of Sonogashira coupling of aryl bromides compared to iodides under mechanochemical conditions.^129^ Overall, this multi-component strategy is highly general and atom economical (*e.g.*, atom economy ranges 68–73%) where no solvent or inert atmospheric conditions are required.

## Conclusions

This mechanochemical ball milling reaction is a general, robust and straightforward methodology for the synthesis of cycloadducts from unsaturated hydrocarbons and diazoacetate compounds. The metal foil is switchable and recyclable without the need for solvent, inert gas, or heat. Using the same batch reactor, we were able to insert silver foil to heterogeneously catalyze the cyclopropenation of internal alkynes or insert copper foil to catalyze the cyclopropenation of terminal alkynes in excellent overall yields. Our findings indicated that the solid–solid milling of a diazoacetate and alkyne was equally as effective as liquid–liquid milling. Ethyl diazoacetate was used effectively for cyclopropenation transformations that are not known for traditional systems due to side reactions involving the halogenated solvent. An additional feature of this solvent-free mechanochemical [2 + 1] cycloaddition chemistry is the highly selective cyclopropenation of substrates containing unprotected amino and silyl groups. This feature obviates the need for synthetic operations such as the use of blocking groups or temporary modifications to the reagent. Finally, a one-pot domino Sonogashira coupling/cyclopropenation solvent-free mechanochemical reaction as an organometallic-catalyzed three-component coupling of a terminal acetylene, aryl halide and diazoacetate was discovered that resulted in good overall yields of a library of poly-substituted cyclopropene compounds. Due to lack of commercially available alkynes, a linear synthetic approach to derive a library of cyclopropenyl-containing compounds would be inefficient and require complex reaction set-ups and purification procedures. Future work will explore and measure the selectivity and efficiency of other carbene transfer reactions using solvent-free mechanochemical ball milling conditions.

## Conflicts of interest

There are no conflicts to declare.

## Supplementary Material

Supplementary information
