# Supplementary material for: Recyclable heterogeneous metal foil-catalyzed cyclopropenation of alkynes and diazoacetates under solvent-free mechanochemical reaction conditions
Source: Chem Sci. 2018 Apr 24;9(20):4650–61. doi: 10.1039/c8sc00443a (PMC5969500; doi:10.1039/c8sc00443a)
Supplement: Supplementary file 1 [file SC-009-C8SC00443A-s001.pdf]

Electronic Supplementary Material (ESI) for Chemical Science.
This journal is © The Royal Society of Chemistry 2018

ELECTRONIC SUPPLEMENTARY INFORMATION

Recyclable Metal Foil-Catalyzed Cyclopropenation of Alkynes and Diazoacetates under Solvent-Free Mechanochemical Conditions

Longrui Chen[1], Devonna Leslie[2], Michael G. Coleman[2] and James Mack[1]

1. *Department of Chemistry, University of Cincinnati, Cincinnati, Ohio 45221-0037*
2. *School of Chemistry and Materials Science, Rochester Institute of Technology, Rochester, New York 14623-5604*

Supporting Information

Table of Contents

1. General Information……………………………………………………2 – 3
2. Safety Considerations……………………………………………………3
3. General Experimental Procedures……………………………………………4 – 9
4. Experimental Data……………………………………………………10 – 31
5. Spectral Data……………………………………………………32 – 107
6. References……………………………………………………108

General Information

All cyclopropenation and Sonogashira coupling reactions by mechanochemical ball milling were carried out in a SPEX 8000M Miller/Mill, using a custom made stainless steel vial with stainless steel ball bearing. The stainless steel rods used to make stainless steel vials are made of Super-Corrosion-Resistant 316 Stainless Steel purchased from McMaster-Carr Supply. All custom made reaction vials were made by the machine shop at the University of Cincinnati. The silver (99.95% purity) and copper (99.9% purity) foils were purchased from ESPI Metals. Simriz 486 Perfluoroelastomer O-rings (6/16'' ID × 7/16'' OD × 3/32'' width) were purchased from Small Parts Inc. ^{1}H Nuclear Magnetic Resonance (NMR) spectra were obtained using a Bruker Avance 400 MHz spectrometer, ^{13}C NMR spectra were recorded at 100 MHz and all chemical shift values are reported in ppm on the δ scale. NMR chemical shifts are reported in ppm and referenced to the residual solvent peak $CDCl_3$ (δ = 7.26 ppm, ^{1}H; δ = 77.00 ppm ^{13}C) as an internal standard or tetramethylsilane (δ = 0.00 ppm, ^{1}H) as an external standard. Chemical shifts are reported in parts per million (ppm), multiplicities are indicated by s (singlet), d (doublet), t (triplet), q (quartet), m (multiplet) and br (broad). Coupling constants, J, are reported in Hertz. All samples were dissolved in $CDCl_3$ with a TMS internal standard prior to analysis. Mass spectral determinations were carried out by using ESI as ionization source. Analytical TLC were performed on silica gel plates purchased from Agela Technologies. Flash column chromatography was performed on a CombiFlash® Automated Flash Chromatography system by using RediSep Rf Gold® high performance flash columns (fine spherical silica gel 20-40 µm). GC-MS yields for compounds **1**[1], **2**[2], **13**[1], **21**[3], **24**[2], **34**[4], **35**[4], **38**[5], **39** – **41**, **42 - 45**[6], **46, 47**, **48**[2], **49**, **50**[2], **51**[2], **52**[7], **53**[8], **54**[7], **55**[9], **56**[8], **57**[7], **58**[10], **59**[10], **60**[11], **61**[12], **62**[13], **63**[14], and **64**[15] were obtained using a Hewlett-Packard 6890 series GC-MS with a Zebron ZB-5, 15 mm × 0.25 mm × 0.25 mm column with chlorobenzene serving as an internal standard. The GC-MS peaks for products and internal standard rendered the GC yield and relative product ratio based on different peak areas.

Deuterated chloroform was obtained from Cambridge Isotope Laboratories Inc. and used without further purification. All terminal alkynes and aryl halides were purchased from Acros Organics and used without further purification. Palladium catalysts including trans-dichlorobis(triphenylphosphine)palladium(II), tetrakis(triphenylphosphine)palladium(0), palladium(II) acetate and bis(dibenzylideneacetone)palladium(0) were purchased from Strem Chemicals Inc. and used without further purification. Potassium carbonate (99+% purity) used in Sonogashira coupling reactions was purchased from Acros Organics. Methyl phenyldiazoacetate, methyl *p*-bromophenyldiazoacetate, methyl *p*-methoxyphenyldiazoacetate, methyl *p*-*tert*-butylphenyldiazoacetate, methyl *p*-trifluorophenyldiazoacetate, methyl 1-naphthyldiazoacetate, methyl *p*-methylphenyldiazoacetate, ethyl diazomalonate were prepared according to known procedures.[16] Ethyl diazoacetate was purchased as a 15% solution in toluene from Sigma Aldrich.

Safety Considerations

Diazoacetate compounds should be handled with care to avoid exposure and/or explosion. Proper personal protective equipment and engineering controls (i.e fume hoods) should be used at all times. Diazoacetate compounds should be refrigerated at 0°C and never exposed to direct sunlight for extended periods of time. Mechanochemical ball milling of diazoacetates can become pressurized due to the generation of N_2 gas in a sealed-vessel. Caution should be exercised to avoid exposure when opening the vial after milling.

General Experimental Procedures

Copper Foil-Catalyzed Cyclopropenation of Terminal Alkynes with Diazoacetate Compounds

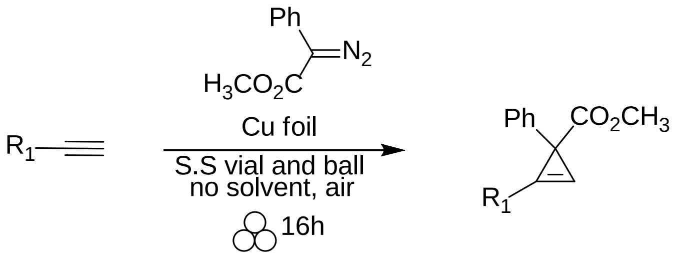

In all cases, the diazoacetate reagent was used as limiting reagent. Methyl phenyldiazoacetate (1.136 mmol), arylacetylene (5.681 mmol), were added to a custom-made 2.0 × 0.5 inches screw stainless steel vial, lined with a 1.67 × 1.56 inches copper foil sheet. The custom-made vial was equipped with a perfluoroelastomer O-ring and a 3/16'' stainless steel ball bearing (0.43g) was added. After placement of the vial in a SPEX SamplePrep 8000M mixer/mill, the reagents were ball milled at 18Hz for 16 hours. The resulting mixture was dissolved in ethyl acetate, filtered, and the organic layer was washed with water using a separatory funnel. The organic layer was then dried over anhydrous magnesium sulfate and filtered. Ethyl acetate was removed under reduced pressure using a rotary evaporator to afford the crude reaction mixture. The crude reaction mixture was purified by automated flash column chromatography on silica gel using 95:5 petroleum ether/ethyl acetate as eluent to afford the titled compound.

Silver Foil-Catalyzed Cyclopropenation of Internal Alkynes with Diazoacetate Compounds

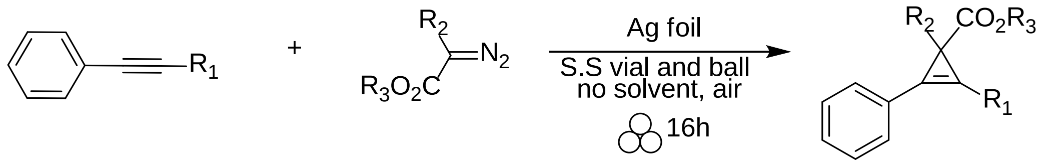

Diazoacetate (1.00 eq) and disubstituted acetylenic compounds (5 eqs) were added to a custom-made 2.0 × 0.5 inches screw stainless steel vial, lined with a 1.67 × 1.56 inches silver foil sheet. The custom-made vial was equipped with a perfluoroelastomer O-ring and a 3/16'' stainless steel ball bearing (0.43g) was added. After placement of the vial in a SPEX SamplePrep 8000M mixer/mill, the reagents were ball milled at 18 Hz for 16 hours. The resulting mixture was dissolved in ethyl acetate, filtered, and the organic layer was washed with water using a separatory funnel. The organic layer was dried over anhydrous magnesium sulfate and filtered. The ethyl acetate was then removed under reduced pressure via a rotary evaporator to afford a crude reaction mixture. The crude reaction mixture was purified by automated flash column chromatography on silica gel, using 95:5 petroleum ether/ethyl acetate as eluent to afford the titled compound.

Metal Foil-Catalyzed Cycloaddition of Unsaturated Hydrocarbons with Methyl Phenyldiazoacetate

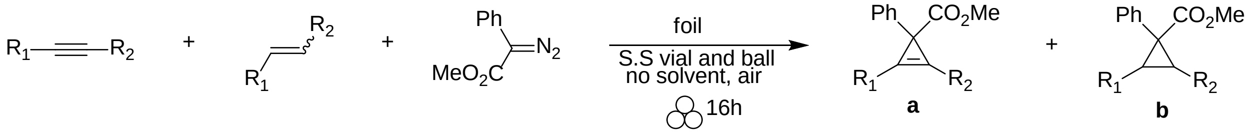

Methyl phenyldiazoacetate (1.0 eq), acetylene (1.5 eqs), and alkene (1.5 eqs) were added to a custom-made 2.0 × 0.5 inches screw stainless steel vial, lined with a 1.67 × 1.56 inches metal foil sheet. The custom-made vial was equipped with a perfluoroelastomer O-ring and a 3/16'' stainless steel ball bearing (0.43g) was added. After placement of the vial in a SPEX SamplePrep 8000M mixer/mill, the reagents were ball milled at 18Hz for 16 hours. The resulting mixture was dissolved in ethyl acetate, filtered, and the organic layer was washed with water using a separatory funnel. The organic layer was

dried over anhydrous magnesium sulfate and filtered. The ethyl acetate was then removed under reduced pressure via a rotary evaporator to afford a crude mixture of the product(s). The crude product mixture was purified by automated flash column chromatography on silica gel, using 95:5 petroleum ether/ethyl acetate as eluent and analyzed by GC-MS (chlorobenzene, internal standard) to afford the titled compound.

Metal Foil-Catalyzed Cycloaddition of Enynes with Methyl Phenyldiazoacetate

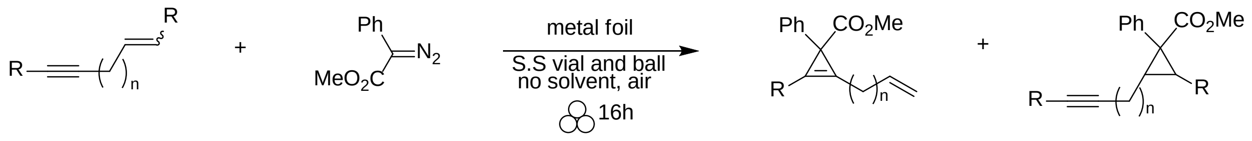

Methyl phenyldiazoacetate (1 eq) and enyne (eqs) were added to a custom-made 2.0 × 0.5 inches screw stainless steel vial, lined with a 1.67 × 1.56 inches metal foil sheet. The custom-made vial was equipped with a perfluoroelastomer O-ring and a 3/16'' stainless steel ball bearing (0.43g) was added. After placement of the vial in a SPEX SamplePrep 8000M mixer/mill, the reagents were ball milled at 18 Hz for 16 hours. The resulting reaction mixture was dissolved in ethyl acetate, filtered, and the organic layer was washed with water using a separatory funnel. The organic layer was dried over anhydrous magnesium sulfate and filtered. The ethyl acetate was then removed under reduced pressure via a rotary evaporator to afford a crude mixture of the product. The crude reaction mixture was purified by automated flash column chromatography on silica gel, using 95:5 petroleum ether/ethyl acetate as eluent and analyzed by ^{1}H NMR and GC-MS (chlorobenzene, internal standard). After running both copper foil and silver foil reactions, the pure products were mixed (1:1) and a subsequent GC-MS was ran to unequivocally quantify the titled cycloaddition product.

Silver Foil-Catalyzed Sonogashira Coupling Reactions

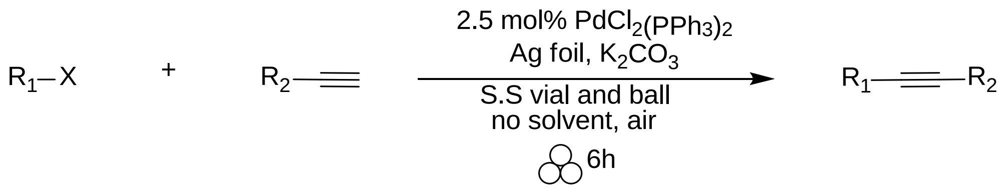

In all cases, the terminal alkyne was used as limiting reagent. Phenylacetylene (1.96 mmol), iodobenzene (1.96 mmol), palladium catalyst (0.049 mmol) and potassium carbonate (1.96 mmol), were added to a custom-made 2.0 × 0.5 inches screw stainless steel vial and lined with a 1.67 × 1.56 inches silver foil sheet. The custom-made vial was equipped with a perfluoroelastomer O-ring and a 3/16'' stainless steel ball bearing (0.43g) was added. After placement of the vial in a SPEX SamplePrep 8000M mixer/mill, the reagents were ball milled at 18 Hz for 6 hours. The resulting mixture was dissolved with ethyl acetate, filtered, and the organic layer was washed with water using a separatory funnel. The organic layer was dried over anhydrous magnesium sulfate and filtered. The ethyl acetate was then removed under reduced pressure via a rotary evaporator to afford a crude reaction mixture. The crude product mixture was purified by automated flash column chromatography on silica gel, using 95:5 petroleum ether/ethyl acetate as eluent and analyzed by GC-MS (chlorobenzene, internal standard) to afford the titled compound.

Silver Foil-Catalyzed Tandem 'One-Pot' Multi-Component Sonogashira/Cyclopropenation Reaction

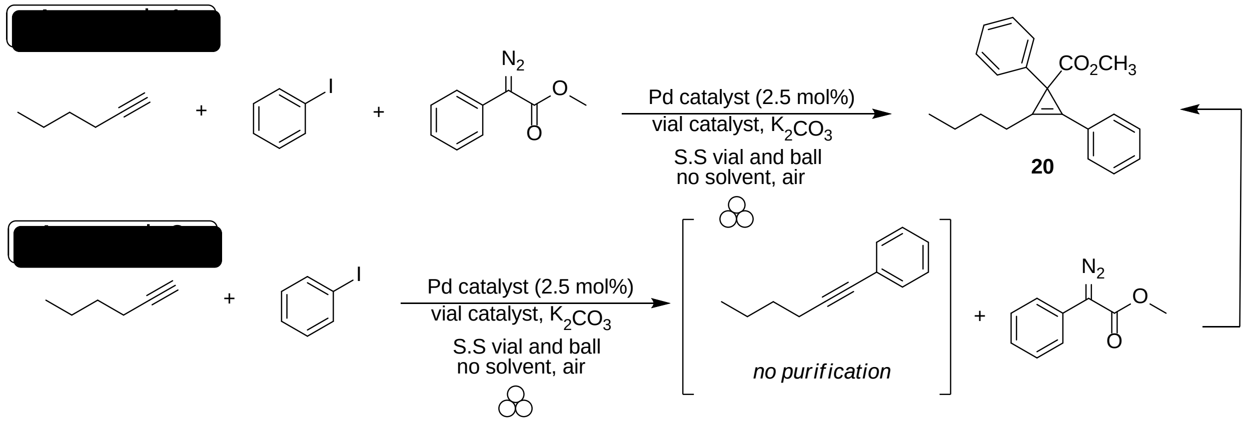

Approach 1: Methyl phenyldiazoacetate (1.136 mmol), phenylacetylene (3.409 mmol), iodobenzene (3.409 mmol), palladium catalyst (0.085 mmol), potassium carbonate (3.409 mmol), were added to a custom-made 2.0 × 0.5 inches screw stainless steel vial and lined with a 1.67 × 1.56 inches silver foil sheet. The custom-made vial was equipped with a perfluoroelastomer O-ring and a 3/16'' stainless steel ball bearing (0.43g) was added. After placement of the vial in a SPEX SamplePrep 8000M mixer/mill, the reagents were ball milled at 18Hz for 22 hours. When reaction was finished, the resulting mixture was dissolved in ethyl acetate, filtered, and the organic layer was washed with water using a separatory funnel. The organic layer was dried over anhydrous magnesium sulfate and filtered. The ethyl acetate was then removed under reduced pressure via a rotary evaporator to afford a crude reaction mixture. The crude reaction mixture was purified by automated flash column chromatography on silica gel , using 95:5 petroleum ether/ethyl acetate as eluent to afford the titled compound.

Approach 2: Phenylacetylene (3.409 mmol), iodobenzene (3.409 mmol), palladium catalyst (0.085 mmol), potassium carbonate (3.409 mmol), were added to a custom-made 2.0 × 0.5 inches screw stainless steel vial and lined with a 1.67 × 1.56 inches silver foil sheet. The custom-made vial was equipped with a perfluoroelastomer O-ring and a 3/16'' stainless steel ball bearing (0.43g) was added.

After placement of the vial in a SPEX SamplePrep 8000M mixer/mill, the reagents were ball milled at 18 Hz for 6 hours. Then, the vial was carefully opened and methyl phenyldiazoacetate (1.136 mmol) was added. The vial was reinstalled in the mixer/mill and ball milled at 18 HZ for another 16 hours. When the reaction mixture were ball milled for a total of 22 hours, the reaction vial was opened and the crude reaction mixture was dissolved in ethyl acetate, filtered, and the organic layer was washed with water using a separatory funnel. The organic layers were dried over anhydrous magnesium sulfate and filtered. The ethyl acetate was then removed under reduced pressure via a rotary evaporator to afford a crude reaction mixture. The crude reaction mixture was purified by automated flash column chromatography on silica gel , using 95:5 petroleum ether/ethyl acetate as eluent to afford the titled compound.

Experimental Data

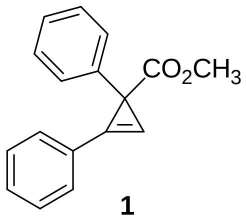

1

Methyl 1,2-diphenylcycloprop-2-ene-1-carboxylate (1)

Purified by column chromatography (silica gel, 5% ethyl acetate/petroleum ether to 15% ethyl acetate/petroleum ether gradient) to afford compound **1** (Cu foil: 88% yield, Ag foil: <5% yield). **^{1}H NMR** ($CDCl_3$, 400 MHz): δ (ppm) = 7.52 (dd, *J*= 8.0 Hz, 1.6 Hz, 2H), 7.33-7.27 (m, 5H), 7.21-7.17 (m, 2H), 7.12-7.09 (m, 2H), 3.61 (s, 3H). **^{13}C NMR** ($CDCl_3$, 100 MHz): δ (ppm) = 173.9, 139.7, 128.9, 128.8, 127.8, 127.1, 127.0, 125.4, 124.3, 116.2, 99.2, 51.1, 32.5. **GC-MS**: $C_{17}H_{14}O_2$ [M^+]: 250. The spectroscopic data is consistent with previously reported data.[1]

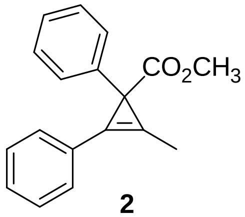

2

Methyl 2-methyl-1,3-diphenylcycloprop-2-ene-1-carboxylate (2)

Purified by column chromatography (silica gel, 5% ethyl acetate/petroleum ether to 15% ethyl acetate/petroleum ether gradient) to afford compound **2** (Cu foil: 10% yield, Ag foil: 90% yield). ^{1}H NMR ($CDCl_3$, 400 MHz): δ (ppm) = 7.54 (d, *J*= 7.2 Hz, 2H), 7.41-7.16 (m, 8H), 3.69 (s, 3H), 2.38 (s, 3H). ^{13}C NMR ($CDCl_3$, 100 MHz): δ (ppm) = 175.2, 141.1, 129.2, 128.8, 128.8, 128.2, 128.0, 126.6, 126.2, 111.3, 108.5, 51.9, 35.3, 9.6. GC-MS: $C_{18}H_{16}O_2$ [M^+]: 264. The spectroscopic data is consistent with previously reported data.[2]

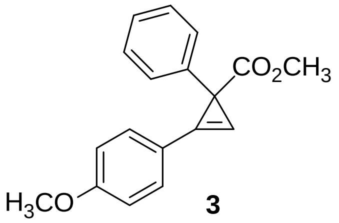

3

Methyl 2-(4-methoxyphenyl)-1-phenylcycloprop-2-ene-1-carboxylate (3)

Purified by column chromatography (silica gel, 5% ethyl acetate/petroleum ether to 15% ethyl acetate/petroleum ether gradient) to afford compound **3** in 88% yield. **^{1}H NMR** ($CDCl_3$, 400 MHz): δ (ppm) = 7.59 (d, *J*= 7.6 Hz, 1H), 7.47-7.43 (m, 3H), 7.28-7.14 (m, 3H), 6.90 (d, *J*= 8.8 Hz, 2H), 6.40 (d, *J*= 2.4 Hz, 1H), 3.76 (s, 3H), 3.65 (s, 3H). **^{13}C NMR** ($CDCl_3$, 100 MHz): δ (ppm) = 169.8, 145.0, 142.5, 140.6, 127.8, 126.5, 126.4, 124.7, 123.6, 119.6, 113.0, 54.2, 53.2, 51.3. **GC-MS**: $C_{18}H_{16}O_3$ [M^+]: 280. The spectroscopic data is consistent with previously reported data.[1]

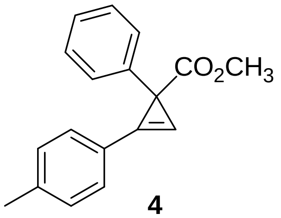

4

Methyl 1-phenyl-2-(*p*-tolyl)cycloprop-2-ene-1-carboxylate (4)

Purified by column chromatography (silica gel, 5% ethyl acetate/petroleum ether to 15% ethyl acetate/petroleum ether gradient) to afford compound **4** in 95% yield. **^{1}H NMR** ($CDCl_3$, 400 MHz): δ (ppm) = 7.42 (d, *J*= 8.0 Hz, 2H), 7.30 (d, *J*= 7.2 Hz, 2H), 7.20-7.17 (m, 2H), 7.14-7.10 (m, 3H), 7.04 (s, 1H), 3.61 (s, 3H), 2.28 (s, 3H). **^{13}C NMR** ($CDCl_3$, 100 MHz): δ (ppm) = 175.2, 141.0, 140.4, 129.9, 129.6, 128.2, 128.0, 126.4, 122.6, 116.9, 99.1, 52.2, 33.4, 21.6. **GC-MS**: $C_{18}H_{16}O_2$ [M^+]: 264. **HRMS (ESI)** m/z calculated for $C_{18}H_{17}O_2$, 265.1264, found 265.1265 [MH^+].

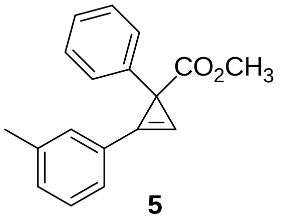

5

Methyl 1-phenyl-2-(*m*-tolyl)cycloprop-2-ene-1-carboxylate (5)

Purified by column chromatography (silica gel, 5% ethyl acetate/petroleum ether to 15% ethyl acetate/petroleum ether gradient) to afford compound **5** in 88% yield. **^{1}H NMR** ($CDCl_3$, 400 MHz): δ (ppm) = 7.43-7.37 (m, 4H), 7.32-7.26 (m, 3H), 7.21-7.17 (m, 3H), 3.70 (s, 3H), 2.35 (s, 3H). **^{13}C NMR** ($CDCl_3$, 100 MHz): δ (ppm) = 175.1, 140.9, 138.6, 130.9, 130.4, 128.8, 128.2, 128.1, 127.1, 126.5, 125.2, 117.1, 100.1, 52.2, 33.5, 21.3. **GC-MS**: $C_{18}H_{16}O_2$ [M^+]: 264. **HRMS (ESI)** m/z calculated for $C_{18}H_{17}O_2$, 265.1265, found 265.1265 [MH^+].

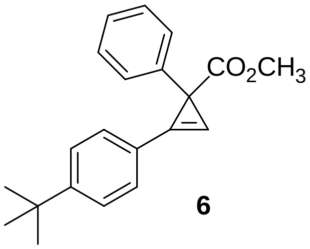

6

Ethyl 2-(4-(tert-butyl)phenyl)-1-phenylcycloprop-2-ene-1-carboxylate (6)

Purified by column chromatography (silica gel, 5% ethyl acetate/petroleum ether to 15% ethyl acetate/petroleum ether gradient) to afford compound **6** in 81% yield. **^{1}H NMR** ($CDCl_3$, 400 MHz): δ (ppm) = 7.46 (d, *J*= 8.4 Hz, 2H), 7.35-7.29 (m, 4H), 7.19-7.16 (m, 2H), 7.11-7.07 (m, 1H), 7.04 (s, 1H), 3.60 (s, 3H), 1.21 (s, 9H). **^{13}C NMR** ($CDCl_3$, 100 MHz): δ (ppm) = 174.0, 152.3, 139.9, 128.6, 127.1, 126.9, 125.3, 124.8, 121.4, 115.9, 98.0, 51.0, 33.8, 32.3, 30.1. **GC-MS**: $C_{21}H_{22}O_2$ [M^+]: 306. **HRMS (ESI)** m/z calculated for $C_{21}H_{23}O_2$, 307.1634, found 307.1633 [MH^+].

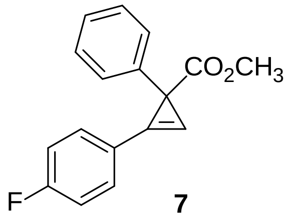

7

Methyl 2-(4-fluorophenyl)-1-phenylcycloprop-2-ene-1-carboxylate (7)

Purified by column chromatography (silica gel, 5% ethyl acetate/petroleum ether to 15% ethyl acetate/petroleum ether gradient) to afford compound **7** in 85% yield. **^{1}H NMR** ($CDCl_3$, 400 MHz): δ (ppm) = 7.60-7.57 (m, 2H), 7.36 (d, *J*= 6.8 Hz, 2H), 7.29-7.26 (m, 2H), 7.21-7.18 (m, 1H), 7.16 (s, 1H), 7.10-7.06 (m, 2H), 3.69 (s, 3H). **^{13}C NMR** ($CDCl_3$, 100 MHz): δ (ppm) = 174.8, 140.7, 131.9, 131.8, 128.1, 128.1, 126.6, 121.8, 116.3, 116.1, 99.8, 52.2, 33.7. **GC-MS**: $C_{17}H_{13}FO_2$ [M^+]: 268. **HRMS (ESI)** m/z calculated for $C_{17}H_{14}FO_2$, 269.0977, found 269.0976 [MH^+].

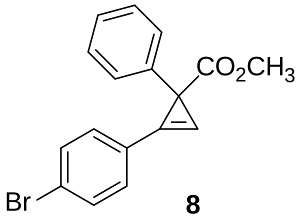

8

Methyl 2-(4-bromophenyl)-1-phenylcycloprop-2-ene-1-carboxylate (8)

Purified by column chromatography (silica gel, 5% ethyl acetate/petroleum ether to 15% ethyl acetate/petroleum ether gradient) to afford compound **8** in 85% yield. **^{1}H NMR** ($CDCl_3$, 400 MHz): δ (ppm) = 7.53 (d, *J*= 8.8 Hz, 2H), 7.45 (d, *J*= 8.4 Hz, 2H), 7.34 (d, *J*= 8.0 Hz, 2H), 7.29-7.25 (m, 2H), 7.24 (s, 1H), 7.22-7.18 (m, 1H), 3.70 (s, 3H). **^{13}C NMR** ($CDCl_3$, 100 MHz): δ (ppm) = 174.6, 140.5, 132.2, 131.2, 128.1, 128.1, 126.6, 124.4, 124.4, 116.6, 101.3, 52.2, 33.7. **GC-MS**: $C_{17}H_{13}BrO_2$ [M^+]: 328. The spectroscopic data is consistent with previously reported data.[1]

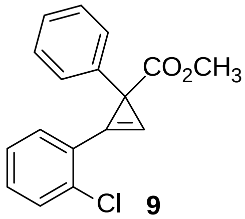

9

Methyl 2-(2-chlorophenyl)-1-phenylcycloprop-2-ene-1-carboxylate (9)

Purified by column chromatography (silica gel, 5% ethyl acetate/petroleum ether to 15% ethyl acetate/petroleum ether gradient) to afford compound **9** in 82% yield. **^{1}H NMR** ($CDCl_3$, 400 MHz): δ (ppm) = 7.55-7.52 (m, 1H), 7.47-7.44 (m, 2H), 7.39-7.37 (m, 2H), 7.31-7.27 (m, 4H), 7.23-7.19 (m, 1H), 3.71 (s, 3H). **^{13}C NMR** ($CDCl_3$, 100 MHz): δ (ppm) = 174.7, 140.5, 136.2, 131.6, 130.9, 130.1, 128.2, 128.1, 127.0, 126.6, 124.2, 114.9, 104.9, 52.3, 33.2. **HRMS (ESI)** m/z calculated for $C_{17}H_{14}ClO_2$, 285.0655, found 285.0654 [MH^+].

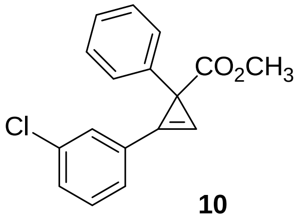

10

Methyl 2-(3-chlorophenyl)-1-phenylcycloprop-2-ene-1-carboxylate (10)

Purified by column chromatography (silica gel, 5% ethyl acetate/petroleum ether to 15% ethyl acetate/petroleum ether gradient) to afford compound **10** in 84% yield. **^{1}H NMR** ($CDCl_3$, 400 MHz): δ (ppm) = 7.59 (s, 1H), 7.49-7.49 (m, 1H), 7.37-7.33 (m, 4H), 7.31-7.27 (m, 3H), 7.23-7.21 (m, 1H), 3.71 (s, 3H). **^{13}C NMR** ($CDCl_3$, 100 MHz): δ (ppm) = 174.6, 140.3, 134.8, 130.2, 130.1, 129.6, 128.2, 128.1, 127.9, 127.2, 126.7, 116.5, 102.1, 52.3, 33.8. **GC-MS**: $C_{17}H_{13}ClO_2$ [M^+]: 284. **HRMS (ESI)** m/z calculated for $C_{17}H_{14}ClO_2$, 285.0655, found 285.0655 [MH^+].

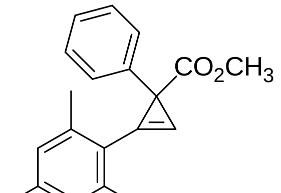

11

Methyl 2-mesityl-1,3-diphenylcycloprop-2-ene-1-carboxylate (11)

Purified by column chromatography (silica gel, 5% ethyl acetate/petroleum ether to 10% ethyl acetate/petroleum ether gradient) to afford compound **11** in 78% yield. **^{1}H NMR** ($CDCl_3$, 400 MHz): δ (ppm) = 7.60-7.58 (d, *J*= 8.0 Hz, 2H), 7.45-7.38 (m, 4H), 7.36-7.32 (m, 1H), 7.25-7.22 (m, 2H), 7.17-7.14 (m, 1H), 6.92 (s, 2H), 3.69 (s, 3H), 2.33 (s, 6H), 2.30 (s, 3H). **^{13}C NMR** ($CDCl_3$, 100 MHz): δ (ppm) = 175.2, 141.4, 139.2, 139.2, 130.1, 129.1, 129.0, 128.7, 128.2, 128.0, 127.5, 126.2, 123.2, 110.6, 109.2, 52.0, 37.1, 21.4, 21.2. **GC-MS**: $C_{26}H_{24}O_2$ [M^+]: 368. **HRMS (ESI)** m/z calculated for $C_{26}H_{24}O_2Na$, 391.1855, found 391.1856 [MNa^+].

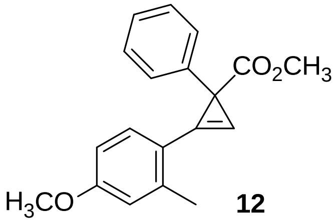

12

Methyl 2-(4-methoxy-2-methylphenyl)-1-phenylcycloprop-2-ene-1-carboxylate (12)

Purified by column chromatography (silica gel, 5% ethyl acetate/petroleum ether to 15% ethyl acetate/petroleum ether gradient) to afford compound **12** in 90% yield. **^{1}H NMR** ($CDCl_3$, 400 MHz): δ (ppm) = 7.33-7.28 (m, 3H), 7.18-7.14 (m, 2H), 7.09-7.05 (m, 1H), 6.97 (s, 1H), 6.70 (d, *J*= 2.4 Hz, 1H), 6.63 (dd, *J*= 8.4 Hz, 2.8 Hz, 2H), 3.65 (s, 3H), 3.58 (s, 3H), 2.36 (s, 3H). **^{13}C NMR** ($CDCl_3$, 100 MHz): δ (ppm) = 175.3, 160.9, 142.2, 141.3, 132.1, 128.2, 128.0, 126.4, 117.0, 117.0, 115.5, 111.2, 99.3, 55.3, 52.1, 32.3, 20.3. **GC-MS**: $C_{19}H_{18}O_3$ [M^+]: 294. **HRMS (ESI)** m/z calculated for $C_{19}H_{19}O_3$, 295.1394, found 295.1394 [MH^+].

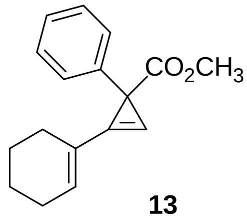

13

Methyl 2-(cyclohex-1-en-1-yl)-1-phenylcycloprop-2-ene-1-carboxylate (13)

Purified by column chromatography (silica gel, 5% ethyl acetate/petroleum ether to 15% ethyl acetate/petroleum ether gradient) to afford compound **13** in 86% yield. **^{1}H NMR** ($CDCl_3$, 400 MHz): δ (ppm) = 7.34-7.25 (m, 4H), 7.22-7.17 (m, 1H), 6.85 (s, 1H), 6.23 (m, 1H), 3.68 (s, 3H), 2.40-2.17 (m, 4H), 1.72-1.60 (m, 4H). **^{13}C NMR** ($CDCl_3$, 100 MHz): δ (ppm) = 175.4, 141.3, 136.1, 128.4, 127.9, 126.2, 124.2, 118.8, 97.7, 52.1, 33.4, 26.8, 25.7, 22.2, 21.7. **GC-MS**: $C_{17}H_{18}O_2$ [M^+]: 254. **HRMS (ESI)** m/z calculated for $C_{17}H_{19}O_2$, 255.1379, found 255.1378 [MH^+]. The spectroscopic data is consistent with previously reported data.[1]

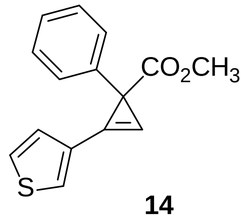

14

Methyl 1-phenyl-2-(thiophen-3-yl)cycloprop-2-ene-1-carboxylate (14)

Purified by column chromatography (silica gel, 5% ethyl acetate/petroleum ether to 15% ethyl acetate/petroleum ether gradient) to afford compound **14** in 90% yield. **^{1}H NMR** ($CDCl_3$, 400 MHz): δ (ppm) = 7.60-7.59 (m, 1H), 7.38-7.26 (m, 6H), 7.22-7.18 (m, 1H), 7.04 (s, 1H), 3.70 (s, 3H). **^{13}C NMR** ($CDCl_3$, 100 MHz): δ (ppm) = 174.9, 140.8, 128.3, 128.2, 128.1, 126.8, 126.7, 126.6, 112.2, 97.8, 52.2, 33.5. **GC-MS**: $C_{15}H_{12}O_2S$ [M^+]: 256. **HRMS (ESI)** m/z calculated for $C_{15}H_{13}O_2S$, 266.0675, found 266.0675 [MH^+].

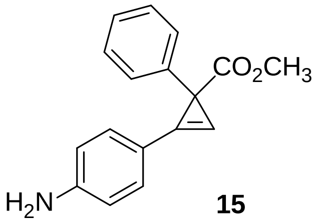

15

Methyl 2-(4-aminophenyl)-1-phenylcycloprop-2-ene-1-carboxylate (15)

Purified by column chromatography (silica gel, 5% ethyl acetate/petroleum ether to 15% ethyl acetate/petroleum ether gradient) to afford compound **15** in 94% yield. **^{1}H NMR** ($CDCl_3$, 400 MHz): δ (ppm) = 7.45 (d, *J*= 8.0 Hz, 2H), 7.35-7.28 (m, 3H), 7.23 (d, *J*= 8.8 Hz, 2H), 6.44 (d, *J*= 8.8 Hz, 2H), 5.20 (d, *J*= 5.6 Hz, 1H), 5.05 (d, *J*= 6.0 Hz, 1H), 3.69 (s, 3H), 2.91 (s, 1H). **^{13}C NMR** ($CDCl_3$, 100 MHz): δ (ppm) = 171.9, 146.1, 137.0, 133.4, 129.0, 128.5, 127.2, 113.0, 110.9, 84.4, 75.1, 60.3, 52.9. **HRMS (ESI)** m/z calculated for $C_{17}H_{16}NO_2$, 266.1175, found 266.1175 [MH^+].

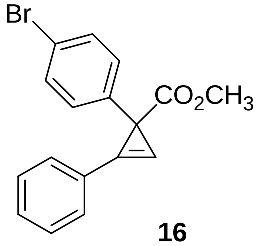

16

Methyl 1-(4-bromophenyl)-2-phenylcycloprop-2-ene-1-carboxylate (16)

Purified by column chromatography (silica gel, 5% ethyl acetate/petroleum ether to 15% ethyl acetate/petroleum ether gradient) to afford compound **16** in 81% yield. **^{1}H NMR** ($CDCl_3$, 400 MHz): δ (ppm) = 7.58 (dd, *J*= 8.0, 1.6 Hz, 2H), 7.44-7.38 (m, 5H), 7.26 (d, *J*= 8.4 Hz, 2H), 7.16 (s, 1H), 3.69 (s, 3H). **^{13}C NMR** ($CDCl_3$, 100 MHz): δ (ppm) = 174.5, 139.9, 131.1, 130.2, 130.0, 129.9, 129.0, 125.0, 120.4, 116.9, 99.7, 52.3, 33.0. **GC-MS**: $C_{17}H_{13}BrO_2$ [M^+]: 328.

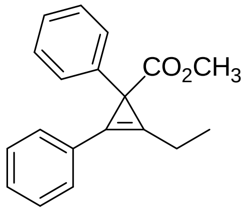

19

Methyl 2-ethyl-1,3-diphenylcycloprop-2-ene-1-carboxylate (19)

Purified by column chromatography (silica gel, 5% ethyl acetate/petroleum ether to 15% ethyl acetate/petroleum ether gradient) to afford compound **19** in 92% yield. **^{1}H NMR** ($CDCl_3$, 400 MHz): δ (ppm) = 7.55 (d, *J*= 7.2 Hz, 2H), 7.41-7.16 (m, 8H), 3.69 (s, 3H), 2.76 (q, *J*= 7.6 Hz, 2H), 1.34 (t, *J*= 7.6 Hz, 3H). **^{13}C NMR** ($CDCl_3$, 100 MHz): δ (ppm) = 175.3, 141.3, 129.3, 128.8, 128.8, 128.2, 128.0, 126.5, 126.2, 116.4, 107.7, 51.9, 35.4, 18.5, 12.2. **GC-MS**: $C_{19}H_{18}O_2$ [M^+]: 278. The spectroscopic data is consistent with previously reported data.[2]

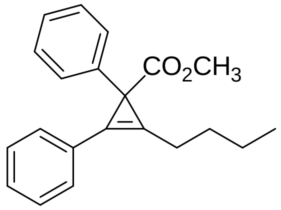

20

Methyl 2-butyl-1,3-diphenylcycloprop-2-ene-1-carboxylate (20)

Purified by column chromatography (silica gel, 5% ethyl acetate/petroleum ether to 15% ethyl acetate/petroleum ether gradient) to afford compound **20** in 95% yield. **^{1}H NMR** ($CDCl_3$, 400 MHz): δ (ppm) = 7.54 (d, *J*= 7.2 Hz, 2H), 7.41-7.30 (m, 5H), 7.27-7.22 (m, 2H), 7.19-7.15 (m, 1H), 3.68 (s, 3H), 2.73 (t, *J*= 7.6 Hz, 2H), 1.73 (q, *J*= 7.6 Hz, 2H), 1.45-1.37 (m, 2H), 0.92 (t, *J*= 7.2 Hz, 3H). **^{13}C NMR** ($CDCl_3$, 100 MHz): δ (ppm) = 175.3, 141.2, 129.3, 128.8, 128.7, 128.2, 128.0, 126.6, 126.1, 115.6, 107.7, 51.9, 35.3, 29.6, 24.6, 22.5, 13.7. **GC-MS**: $C_{21}H_{22}O_2$ [M^+]: 306. The spectroscopic data is consistent with previously reported data.[2]

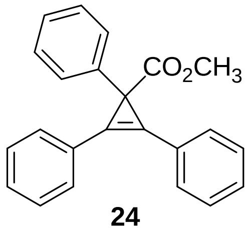

24

Methyl 1,2,3-triphenylcycloprop-2-ene-1-carboxylate (24)

Purified by column chromatography (silica gel, 5% ethyl acetate/petroleum ether to 15% ethyl acetate/petroleum ether gradient) to afford compound **24** in 84% yield. **^{1}H NMR** ($CDCl_3$, 400 MHz): δ (ppm) = 7.64 (d, *J*=7.2 Hz, 4H), 7.39-7.33 (m, 6H), 7.28-7.24 (m, 2H), 7.17-7.13 (m, 2H), 7.08 (d, *J*=7.6 Hz, 1H), 3.59 (s, 3H). **^{13}C NMR** ($CDCl_3$, 100 MHz): δ (ppm) = 174.5, 140.1, 129.9, 129.5, 129.1, 128.2, 128.1, 126.6, 126.5, 111.3, 52.1, 35.4. **GC-MS**: $C_{23}H_{18}O_2$ [M^+]: 326. The spectroscopic data is consistent with previously reported data.[2]

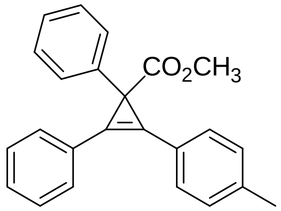

25

Methyl 1,2-diphenyl-3-(p-tolyl)cycloprop-2-ene-1-carboxylate (25)

Purified by column chromatography (silica gel, 5% ethyl acetate/petroleum ether to 15% ethyl acetate/petroleum ether gradient) to afford compound **25** in 85% yield. **^{1}H NMR** ($CDCl_3$, 400 MHz): δ (ppm) = 7.72 (d, *J*= 8.0 Hz, 2H), 7.63 (d, *J*= 8.0 Hz, 2H), 7.47-7.44 (m, 4H), 7.39-7.36 (m, 1H), 7.29-7.23 (m, 4H), 7.19-7.15 (m, 1H), 3.70 (s, 3H), 2.40 (s, 3H). **^{13}C NMR** ($CDCl_3$, 100 MHz): δ (ppm) = 174.5, 140.1, 139.8, 129.9, 129.7, 129.2, 128.9, 128.1, 128.0, 126.7, 126.3, 123.7, 111.1, 110.1, 52.1, 35.2, 21.6. **GC-MS**: $C_{24}H_{20}O_2$ [M^+]: 340. **HRMS (ESI)** m/z calculated for $C_{24}H_{20}O_2Na$, 363.1354, found 363.1355 [MNa^+].

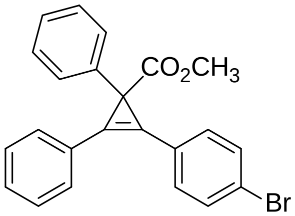

26

Methyl 2-(4-bromophenyl)-1,3-diphenylcycloprop-2-ene-1-carboxylate (26)

Purified by column chromatography (silica gel, 5% ethyl acetate/petroleum ether to 15% ethyl acetate/petroleum ether gradient) to afford compound **26** in 65% yield. **^{1}H NMR** ($CDCl_3$, 400 MHz): δ (ppm) = 7.74-7.70 (m, 4H), 7.49-7.40 (m, 5H), 7.28-7.25 (m, 2H), 7.19-7.14 (m, 3H), 3.71 (s, 3H). **^{13}C NMR** ($CDCl_3$, 100 MHz): δ (ppm) = 174.2, 139.8, 131.8, 131.7, 130.1, 129.8, 129.5, 129.0, 128.1, 128.0, 126.5, 116.4, 116.2, 110.6, 110.4, 52.1, 35.4. **GC-MS**: $C_{23}H_{17}BrO_2$ [M^+]: 404. **HRMS (ESI)** m/z calculated for $C_{23}H_{18}BrO_2$, 405.0459, found 405.0459 [MH^+].

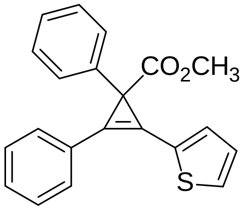

27

Methyl 1,2-diphenyl-3-(thiophen-3-yl)cycloprop-2-ene-1-carboxylate (27)

Purified by column chromatography (silica gel, 5% ethyl acetate/petroleum ether to 15% ethyl acetate/petroleum ether gradient) to afford compound **27** in 86% yield. **^{1}H NMR** ($CDCl_3$, 400 MHz): δ (ppm) = 7.69 (d, *J*= 6.8 Hz, 2H), 7.67-7.66 (m, 1H), 7.48-7.44 (m, 4H), 7.40-7.36 (m, 2H), 7.26-7.24 (m, 3H), 7.20 (d, *J*= 7.2 Hz, 1H), 3.71 (s, 3H). **^{13}C NMR** ($CDCl_3$, 100 MHz): δ (ppm) = 174.3, 140.0, 129.6, 129.2, 129.0, 128.3, 128.1, 128.1, 127.6, 127.5, 126.8, 126.6, 126.5, 108.5, 106.4, 52.1, 35.5. **GC-MS**: $C_{21}H_{16}O_2S$ [M^+]: 332. **HRMS (ESI)** m/z calculated for $C_{21}H_{17}O_2S$, 333.0949, found 333.0948 [MH^+].

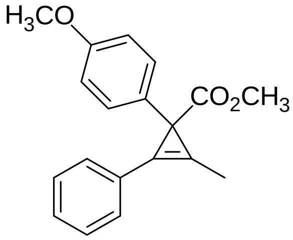

28

Methyl 1-(4-methoxyphenyl)-2-methyl-3-phenylcycloprop-2-ene-1-carboxylate (28)

Purified by column chromatography (silica gel, 5% ethyl acetate/petroleum ether to 15% ethyl acetate/petroleum ether gradient) to afford compound **28** in 86% yield. **^{1}H NMR** ($CDCl_3$, 400 MHz): δ (ppm) = 7.53 (d, *J*= 7.2 Hz, 2H), 7.41-7.38 (m, 2H), 7.34-7.27 (m, 3H), 6.81 (d, *J*= 8.8 Hz, 2H), 3.76 (s, 3H), 3.68 (s, 3H), 2.37 (s, 3H). **^{13}C NMR** ($CDCl_3$, 100 MHz): δ (ppm) = 175.5, 158.0, 133.3, 129.3, 129.2, 128.8, 128.7, 126.7, 113.5, 111.6, 108.6, 55.2, 52.0, 34.6, 9.72. **GC-MS**: $C_{19}H_{18}O_3$ [M^+]: 294. **HRMS (ESI)** m/z calculated for $C_{19}H_{18}O_3$a, 317.1148, found 317.1147 [MNa^+].

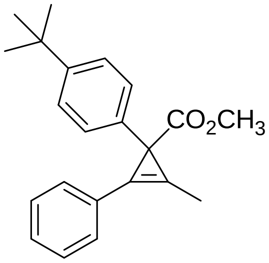

29

Methyl 1-(4-(tert-butyl)phenyl)-2-methyl-3-phenylcycloprop-2-ene-1-carboxylate (29)

Purified by column chromatography (silica gel, 5% ethyl acetate/petroleum ether to 15% ethyl acetate/petroleum ether gradient) to afford compound **29** in 84% yield. **^{1}H NMR** ($CDCl_3$, 400 MHz): δ (ppm) = 7.45 (d, *J*= 7.2 Hz, 2H), 7.31-7.20 (m, 7H), 3.60 (s, 3H), 2.29 (s, 3H), 1.19 (s, 9H). **^{13}C NMR** ($CDCl_3$, 100 MHz): δ (ppm) = 175.4, 148.9, 138.0, 129.3, 128.8, 128.7, 127.8, 126.7, 125.0, 111.4, 108.6, 51.9, 34.9, 34.4, 31.4, 9.79. **GC-MS**: $C_{22}H_{24}O_2$ [M^+]: 320. **HRMS (ESI)** m/z calculated for $C_{22}H_{25}O_2$, 321.1849, found 321.1848 [MH^+]. The spectroscopic data is consistent with previously reported data.[2]

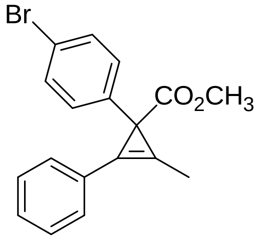

30

Methyl 1-(4-bromophenyl)-2-methyl-3-phenylcycloprop-2-ene-1-carboxylate (30)

Purified by column chromatography (silica gel, 5% ethyl acetate/petroleum ether to 15% ethyl acetate/petroleum ether gradient) to afford compound **30** in 89% yield. **^{1}H NMR** ($CDCl_3$, 400 MHz): δ (ppm) = 7.43 (d, *J*= 6.8 Hz, 2H), 7.34-7.23 (m, 5H), 7.16 (d, *J*= 8.8 Hz, 2H), 3.60 (s, 3H), 2.28 (s, 3H). **^{13}C NMR** ($CDCl_3$, 100 MHz): δ (ppm) = 173.6, 139.0, 130.0, 128.8, 128.1, 127.9, 127.8, 125.1, 119.0, 109.7, 106.9, 51.0, 33.6, 8.5. **GC-MS**: $C_{18}H_{15}BrO_2$ [M^+]: 342. **HRMS (ESI)** m/z calculated for $C_{18}H_{16}BrO_2$, 343.0327, found 343.0328 [MH^+]. The spectroscopic data is consistent with previously reported data.[2]

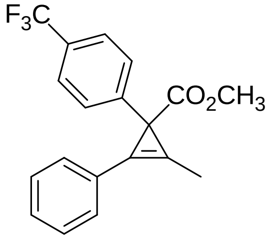

31

Methyl 2-methyl-3-phenyl-1-(4-(trifluoromethyl)phenyl)cycloprop-2-ene-1-carboxylate (31)

Purified by column chromatography (silica gel, 5% ethyl acetate/petroleum ether to 15% ethyl acetate/petroleum ether gradient) to afford compound **31** in 88% yield. **^{1}H NMR** ($CDCl_3$, 400 MHz): δ (ppm) = 7.46-7.39 (m, 6H), 7.36-7.26 (m, 3H), 3.63 (s, 3H), 2.30 (s, 3H). **^{13}C NMR** ($CDCl_3$, 100 MHz): δ (ppm) = 173.4, 144.1, 128.2, 128.1, 127.9, 127.3, 124.9, 123.9, 123.9, 123.8, 109.5, 106.7, 51.0, 34.0, 8.4. **GC-MS**: $C_{19}H_{15}F_3O_2$ [M^+]: 332. **HRMS (ESI)** m/z calculated for $C_{19}H_{16}F_3O_2$, 333.1095, found 333.1096 [MH^+].

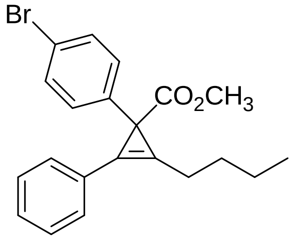

32

Methyl 1-(4-bromophenyl)-2-butyl-3-phenylcycloprop-2-ene-1-carboxylate (32)

Purified by column chromatography (silica gel, 5% ethyl acetate/petroleum ether to 10% ethyl acetate/petroleum ether gradient) to afford compound **32** in 80% yield. **^{1}H NMR** ($CDCl_3$, 400 MHz): δ (ppm) = 7.42 (d, *J*= 6.8 Hz, 2H), 7.33-7.24 (m, 5H), 7.16 (d, *J*= 8.8 Hz, 2H), 3.59 (s, 3H), 2.62 (t, *J*= 7.6 Hz, 2H), 1.63 (q, *J*= 7.6 Hz, 2H), 1.36-1.30 (m, 2H), 0.84 (t, *J*= 7.2 Hz, 3H). **^{13}C NMR** ($CDCl_3$, 100 MHz): δ (ppm) = 174.8, 140.3, 131.0, 129.9, 129.2, 128.9, 128.9, 126.2, 119.9, 115.1, 107.2, 51.9, 34.8, 29.5, 24.5, 22.5, 13.7. **GC-MS**: $C_{21}H_{21}BrO_2$ [M^+]: 384. **HRMS (ESI)** m/z calculated for $C_{21}H_{21}O_2BrNa$, 407.0616, found 407.0617 [MNa^+].

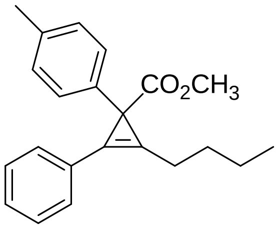

33

Methyl 2-butyl-3-phenyl-1-(p-tolyl)cycloprop-2-ene-1-carboxylate (33)

Purified by column chromatography (silica gel, 5% ethyl acetate/petroleum ether to 15% ethyl acetate/petroleum ether gradient) to afford compound **33** in 79% yield. **^{1}H NMR** ($CDCl_3$, 400 MHz): δ (ppm) = 7.53 (d, *J*= 7.2 Hz, 2H), 7.41-7.37 (m, 2H), 7.33-7.29 (m, 1H), 7.25 (d, *J*= 8.4 Hz, 2H), 7.06 (d, *J*= 8.0 Hz, 2H), 3.68 (s, 3H), 2.73 (t, *J*= 7.6 Hz, 2H), 2.29 (s, 3H), 1.74 (q, *J*= 7.6 Hz, 2H), 1.46-1.40 (m, 2H), 0.93 (t, *J*= 7.2 Hz, 3H). **^{13}C NMR** ($CDCl_3$, 100 MHz): δ (ppm) = 175.4, 138.2, 135.7, 129.3, 128.8, 128.7, 128.6, 128.1, 126.7, 115.7, 107.9, 51.8, 35.0, 29.6, 24.6, 22.5, 21.1, 13.7. **GC-MS**: $C_{22}H_{24}O_2$ [M^+]: 320. **HRMS (ESI)** m/z calculated for $C_{22}H_{25}O_2$, 321.1854, found 321.1854 [MH^+].

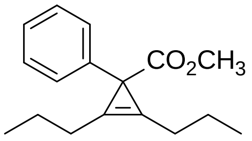

37

Methyl 1-phenyl-2,3-dipropylcycloprop-2-ene-1-carboxylate (37)

Purified by column chromatography (silica gel, 5% ethyl acetate/petroleum ether to 15% ethyl acetate/petroleum ether gradient) to afford compound **37** in 85% yield. **^{1}H NMR** ($CDCl_3$, 400 MHz): δ (ppm) = 7.20-7.07 (m, 5H), 3.58 (s, 3H), 2.42 (t, *J*= 7.6 Hz, 4H), 1.56-1.50 (m, 4H), 0.87 (t, *J*= 7.6 Hz, 6H). **^{13}C NMR** ($CDCl_3$, 100 MHz): δ (ppm) = 176.3, 142.7, 128.2, 127.9, 125.8, 109.9, 51.6, 34.9, 26.3, 20.6, 13.9. **GC-MS**: $C_{17}H_{22}O_2$ [M^+]: 258. **HRMS (ESI)** m/z calculated for $C_{17}H_{23}O_2$, 259.1692, found 259.1698 [MH^+].

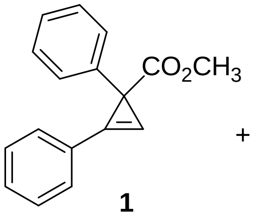

1

+

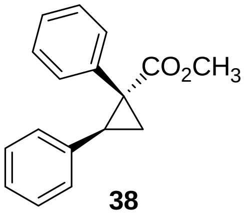

38

Ag-foil catalyzed: GC 91% Yield, (2 : 98, **1** : **38**); Cu-foil catalyzed: GC 92% Yield, (65 : 35, **1** : **38**)

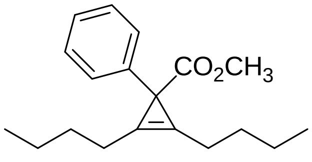

39

+

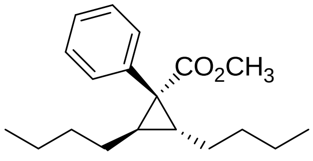

40

Ag-foil catalyzed: GC 80% Yield, (75 : 25, **39** : **40**); Cu-foil catalyzed: GC 40% Yield, (15 : 85, **39** : **40**)

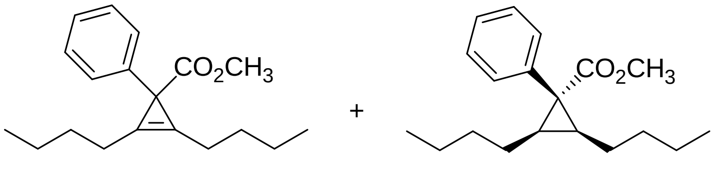

39 **41**

Ag-foil catalyzed: GC 83% Yield, (70 : 30, **39** : **41**); Cu-foil catalyzed: GC 48% Yield, (10 : 90, **39** : **41**)

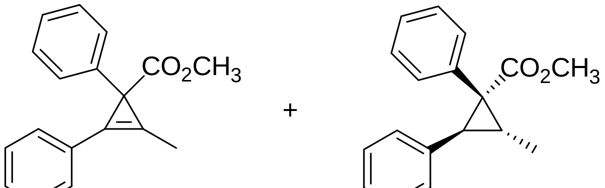

2 **42**

Ag-foil catalyzed: GC 90% Yield, (55 : 45, **2** : **42**); Cu-foil catalyzed: GC 86% Yield, (15 : 85, **2** : **42**)

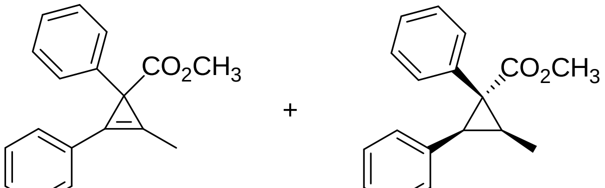

2 **43**

Ag-foil catalyzed: GC 90% Yield, (55 : 45, **2** : **43**); Cu-foil catalyzed: GC 88% Yield, (15 : 85, **2** : **43**)

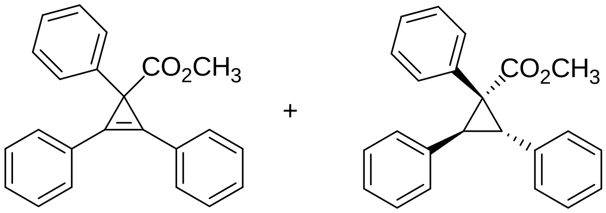

24 **44**

Ag-foil catalyzed: GC 80% Yield, (95 : 5, **24** : **44**); Cu-foil catalyzed: GC 30% Yield, (5 : 95, **24** : **44**)

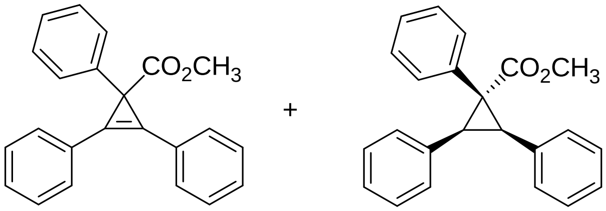

24 **45**

Ag-foil catalyzed: GC 82% Yield, (90 : 10, **24** : **45**); Cu-foil catalyzed: GC 42% Yield, (5 : 95, **24** : **45**)

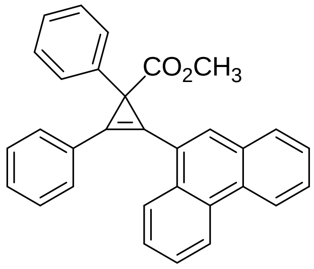

65

Methyl 2-(phenanthren-9-yl)-1,3-diphenylcycloprop-2-ene-1-carboxylate (65)

Purified by column chromatography (silica gel, 5% ethyl acetate/petroleum ether to 10% ethyl acetate/petroleum ether gradient) to afford compound **65** in 55% yield. **^{1}H NMR** ($CDCl_3$, 400 MHz): δ (ppm) = 8.77 (d, *J*= 8.0 Hz, 1H), 8.69 (d, *J*= 8.4 Hz, 1H), 8.55 (d, *J*= 7.2 Hz, 1H), 8.07 (s, 1H), 7.90 (d, *J*= 8.0 Hz, 1H), 7.82 (d, *J*= 6.8 Hz, 2H), 7.76-7.68 (m, 3H), 7.61-7.58 (m, 2H), 7.53-7.49 (m, 2H), 7.46-7.42 (m, 1H), 7.29-7.26 (m, 3H), 7.21-7.18 (m, 1H), 3.74 (s, 3H). **^{13}C NMR** ($CDCl_3$, 100 MHz): δ (ppm) = 174.5, 139.9, 131.2, 130.9, 130.7, 130.7, 130.2, 130.1, 129.5, 129.4, 129.1, 128.1, 128.1, 128.0, 127.2, 127.1, 127.0, 126.4, 126.2, 123.2, 122.9, 122.6, 112.7, 108.6, 52.2, 29.7. **HRMS (ESI)** m/z calculated for $C_{31}H_{23}O_2$, 427.1692, found 427.1691 [MH^+].

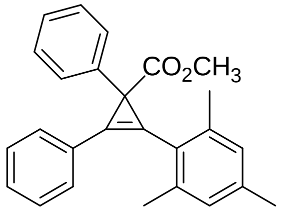

66

Methyl 2-mesityl-1,3-diphenylcycloprop-2-ene-1-carboxylate (66)

Purified by column chromatography (silica gel, 5% ethyl acetate/petroleum ether to 10% ethyl acetate/petroleum ether gradient) to afford compound **66** in 56% yield. **^{1}H NMR** ($CDCl_3$, 400 MHz): δ (ppm) = 7.60-7.58 (d, *J*= 8.0 Hz, 2H), 7.45-7.38 (m, 4H), 7.36-7.32 (m, 1H), 7.25-7.22 (m, 2H), 7.17-7.14 (m, 1H), 6.92 (s, 2H), 3.69 (s, 3H), 2.33 (s, 6H), 2.30 (s, 3H). **^{13}C NMR** ($CDCl_3$, 100 MHz): δ (ppm) = 175.2, 141.4, 139.2, 139.2, 130.1, 129.1, 129.0, 128.7, 128.2, 128.0, 127.5, 126.2, 123.2, 110.6, 109.2, 52.0, 37.1, 21.4,

21.2. **GC-MS**: $C_{26}H_{24}O_2$ [M^+]: 368. **HRMS (ESI)** m/z calculated for $C_{26}H_{24}O_2Na$, 391.1855, found 391.1856 [MNa^+].

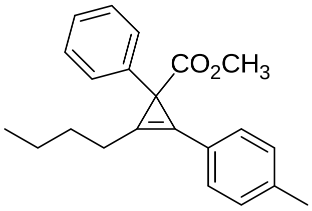

67

Methyl 2-butyl-1-phenyl-3-(*p*-tolyl)cycloprop-2-ene-1-carboxylate (67)

Purified by column chromatography (silica gel, 5% ethyl acetate/petroleum ether to 15% ethyl acetate/petroleum ether gradient) to afford compound **67** (I = 75% yield and Br = 65% yield). **^{1}H NMR** ($CDCl_3$, 400 MHz): δ (ppm) = 7.43 (d, *J*= 8.4 Hz, 2H), 7.35 (d, *J*= 6.8 Hz, 2H), 7.26-7.16 (m, 5H), 3.68 (s, 3H), 2.71 (t, *J*= 7.6 Hz, 2H), 2.37 (s, 3H), 1.72 (q, *J*= 7.6 Hz, 2H), 1.44-1.39 (m, 2H), 0.91 (t, *J*= 7.2 Hz, 3H). **^{13}C NMR** ($CDCl_3$, 100 MHz): δ (ppm) = 175.4, 141.4, 138.9, 129.5, 129.2, 128.1, 127.9, 126.0, 123.7, 114.3, 107.4, 51.8, 35.2, 29.6, 24.5, 22.5, 21.5, 13.7. **GC-MS**: $C_{22}H_{24}O_2$ [M^+]: 320. **HRMS (ESI)** m/z calculated for $C_{22}H_{24}O_2Na$, 343.1668, found 343.1668 [MNa^+].

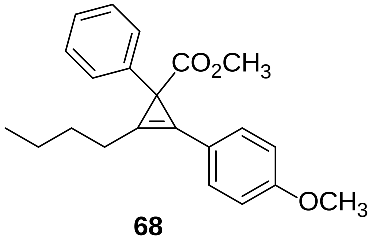

68

Methyl 2-butyl-3-(4-methoxyphenyl)-1-phenylcycloprop-2-ene-1-carboxylate (68)

Purified by column chromatography (silica gel, 5% ethyl acetate/petroleum ether to 20% ethyl acetate/petroleum ether gradient) to afford compound **68** in 74% yield. **^{1}H NMR** ($CDCl_3$, 400 MHz): δ (ppm) = 7.38 (d, *J*= 8.8 Hz, 2H), 7.26 (d, *J*= 8.4 Hz, 2H), 7.17-7.13 (m, 2H), 7.08-7.05 (m, 1H), 6.83 (d, *J*= 8.8 Hz, 2H), 3.69 (s, 3H), 3.58 (s, 3H), 2.61 (t, *J*= 7.6 Hz, 2H), 1.62 (q, *J*= 7.6 Hz, 2H), 1.35-1.29 (m, 2H), 0.82 (t, *J*=

7.2 Hz, 3H). **^{13}C NMR** ($CDCl_3$, 100 MHz): δ (ppm) = 174.4, 158.9, 140.4, 129.7, 127.0, 126.9, 124.9, 118.1, 113.3, 111.6, 106.0, 54.2, 50.7, 34.2, 28.6, 23.4, 21.4, 12.7. **GC-MS**: $C_{22}H_{24}O_3$ [M^+]: 336. **HRMS (ESI)** m/z calculated for $C_{22}H_{25}O_3$, 337.1798, found 337.1797 [MH^+].

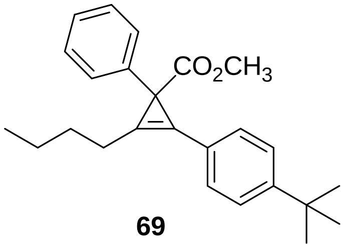

69

Methyl 2-butyl-3-(4-(tert-butyl)phenyl)-1-phenylcycloprop-2-ene-1-carboxylate (69)

Purified by column chromatography (silica gel, 5% ethyl acetate/petroleum ether to 15% ethyl acetate/petroleum ether gradient) to afford compound **69** in 80% yield. **^{1}H NMR** ($CDCl_3$, 400 MHz): δ (ppm) = 7.48 (d, *J*= 8.4 Hz, 2H), 7.42 (d, *J*= 8.4 Hz, 2H), 7.36 (d, *J*= 6.8 Hz, 2H), 7.26-7.22 (m, 2H), 7.18-7.14 (m, 1H), 3.67 (s, 3H), 2.71 (t, *J*= 7.6 Hz, 2H), 1.73 (q, *J*= 7.6 Hz, 2H), 1.45-1.39 (m, 2H), 1.32 (s, 9H), 0.92 (t, *J*= 7.2 Hz, 3H). **^{13}C NMR** ($CDCl_3$, 100 MHz): δ (ppm) = 175.4, 152.0, 141.4, 129.0, 128.2, 127.9, 126.0, 125.8, 123.7, 114.4, 107.5, 51.8, 35.2, 34.8, 31.2, 29.7, 24.6, 22.5, 13.8. **GC-MS**: $C_{25}H_{30}O_2$ [M^+]: 362. **HRMS (ESI)** m/z calculated for $C_{25}H_{31}O_2$, 363.2250, found 363.2251 [MH^+].

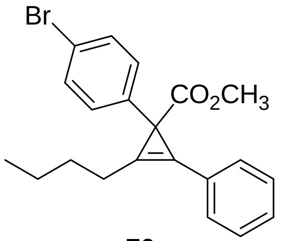

70

Methyl 1-(4-bromophenyl)-2-butyl-3-phenylcycloprop-2-ene-1-carboxylate (70)

Purified by column chromatography (silica gel, 5% ethyl acetate/petroleum ether to 10% ethyl acetate/petroleum ether gradient) to afford compound **70** (I = 74% yield and Br = 70% yield). **^{1}H NMR** ($CDCl_3$, 400 MHz): δ (ppm) = 7.42 (d, *J*= 6.8 Hz, 2H), 7.33-7.24 (m, 5H), 7.16 (d, *J*= 8.8 Hz, 2H), 3.59 (s, 3H), 2.62 (t, *J*= 7.6 Hz, 2H), 1.63 (q, *J*= 7.6 Hz, 2H), 1.36-1.30 (m, 2H), 0.84 (t, *J*= 7.2 Hz, 3H). **^{13}C NMR** ($CDCl_3$,

100 MHz): δ (ppm) = 174.8, 140.3, 131.0, 129.9, 129.2, 128.9, 128.9, 126.2, 119.9, 115.1, 107.2, 51.9, 34.8, 29.5, 24.5, 22.5, 13.7. **GC-MS**: $C_{21}H_{21}BrO_2$ [M^+]: 384. **HRMS (ESI)** m/z calculated for $C_{21}H_{21}O_2BrNa$, 407.0616, found 407.0617 [MNa^+].

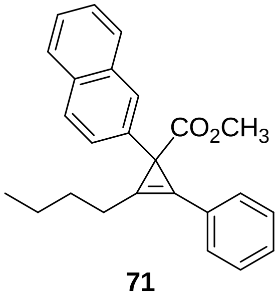

71

Methyl 2-butyl-1-(naphthalen-2-yl)-3-phenylcycloprop-2-ene-1-carboxylate (71)

Purified by column chromatography (silica gel, 5% ethyl acetate/petroleum ether to 10% ethyl acetate/petroleum ether gradient) to afford compound **71** in 68% yield. **^{1}H NMR** ($CDCl_3$, 400 MHz): δ (ppm) = 7.82 (s, 1H), 7.78-7.73 (m, 3H), 7.58 (d, *J*= 7.2 Hz, 2H), 7.49 (d, *J*= 8.4 Hz, 1H), 7.43-7.34 (m, 5H), 3.72 (s, 3H), 2.77 (t, *J*= 7.6 Hz, 2H), 1.75 (q, *J*= 7.6 Hz, 2H), 1.46-1.40 (m, 2H), 0.92 (t, *J*= 7.2 Hz, 3H). **^{13}C NMR** ($CDCl_3$, 100 MHz): δ (ppm) = 175.3, 138.9, 133.6, 132.1, 130.0, 129.6, 129.3, 128.9, 127.7, 127.4, 126.7, 126.4, 125.8, 125.3, 124.0, 115.6, 107.7, 51.9, 35.5, 29.6, 24.6, 22.5, 13.7. **GC-MS**: $C_{25}H_{24}O_2$ [M^+]: 356. **HRMS (ESI)** m/z calculated for $C_{25}H_{24}O_2Na$, 379.1668, found 379.1668 [MNa^+].

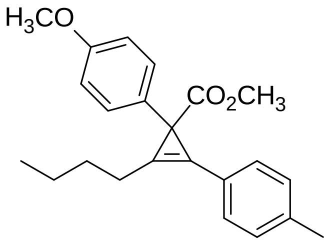

72

Methyl 2-butyl-1-(4-methoxyphenyl)-3-(p-tolyl)cycloprop-2-ene-1-carboxylate (72)

Purified by column chromatography (silica gel, 5% ethyl acetate/petroleum ether to 15% ethyl acetate/petroleum ether gradient) to afford compound **72** in 75% yield. **^{1}H NMR** ($CDCl_3$, 400 MHz): δ (ppm) = 7.35 (d, *J*= 8.0 Hz, 2H), 7.21-7.17 (m, 2H), 7.13 (d, *J*= 8.0 Hz, 2H), 6.71 (d, *J*= 8.8 Hz, 2H), 3.68 (s, 3H), 3.59

(s, 3H), 2.63 (t, *J*= 7.6 Hz, 2H), 1.64 (q, *J*= 7.6 Hz, 2H), 1.37-1.31 (m, 2H), 0.85 (t, *J*= 7.2 Hz, 3H). **^{13}C NMR** ($CDCl_3$, 100 MHz): δ (ppm) = 174.6, 156.8, 137.7, 132.6, 128.5, 128.2, 128.1, 122.8, 113.5, 112.3, 106.6, 54.1, 50.8, 33.5, 28.6, 23.5, 21.4, 20.4, 12.7. **GC-MS**: $C_{23}H_{26}O_3$ [M^+]: 350. **HRMS (ESI)** m/z calculated for $C_{23}H_{27}O_3$, 351.1985, found 351.1984 [MH^+].

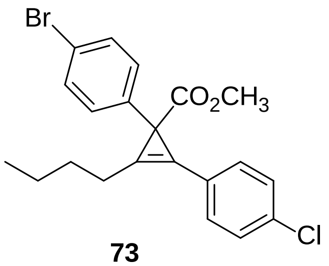

73

Methyl 1-(4-bromophenyl)-2-butyl-3-(4-chlorophenyl)cycloprop-2-ene-1-carboxylate (73)

Purified by column chromatography (silica gel, 5% ethyl acetate/petroleum ether to 15% ethyl acetate/petroleum ether gradient) to afford compound **73** in 60% yield. **^{1}H NMR** ($CDCl_3$, 400 MHz): δ (ppm) = 7.34 (d, *J*= 8.4 Hz, 2H), 7.29-7.29 (m, 4H), 7.12 (d, *J*= 8.4 Hz, 2H), 3.59 (s, 3H), 2.62 (t, *J*= 7.6 Hz, 2H), 1.61 (q, *J*= 7.6 Hz, 2H), 1.34-1.29 (m, 2H), 0.83 (t, *J*= 7.2 Hz, 3H). **^{13}C NMR** ($CDCl_3$, 100 MHz): δ (ppm) = 174.5, 140.0, 132.3, 131.1, 130.4, 129.8, 129.2, 124.7, 120.1, 115.9, 106.4, 52.0, 34.8, 29.5, 24.5, 22.5, 13.7. **GC-MS**: $C_{21}H_{20}BrClO_2$ [M^+]: 418. **HRMS (ESI)** m/z calculated for $C_{21}H_{21}BrClO_2$, 419.0407, found 419.0408 [MH^+].

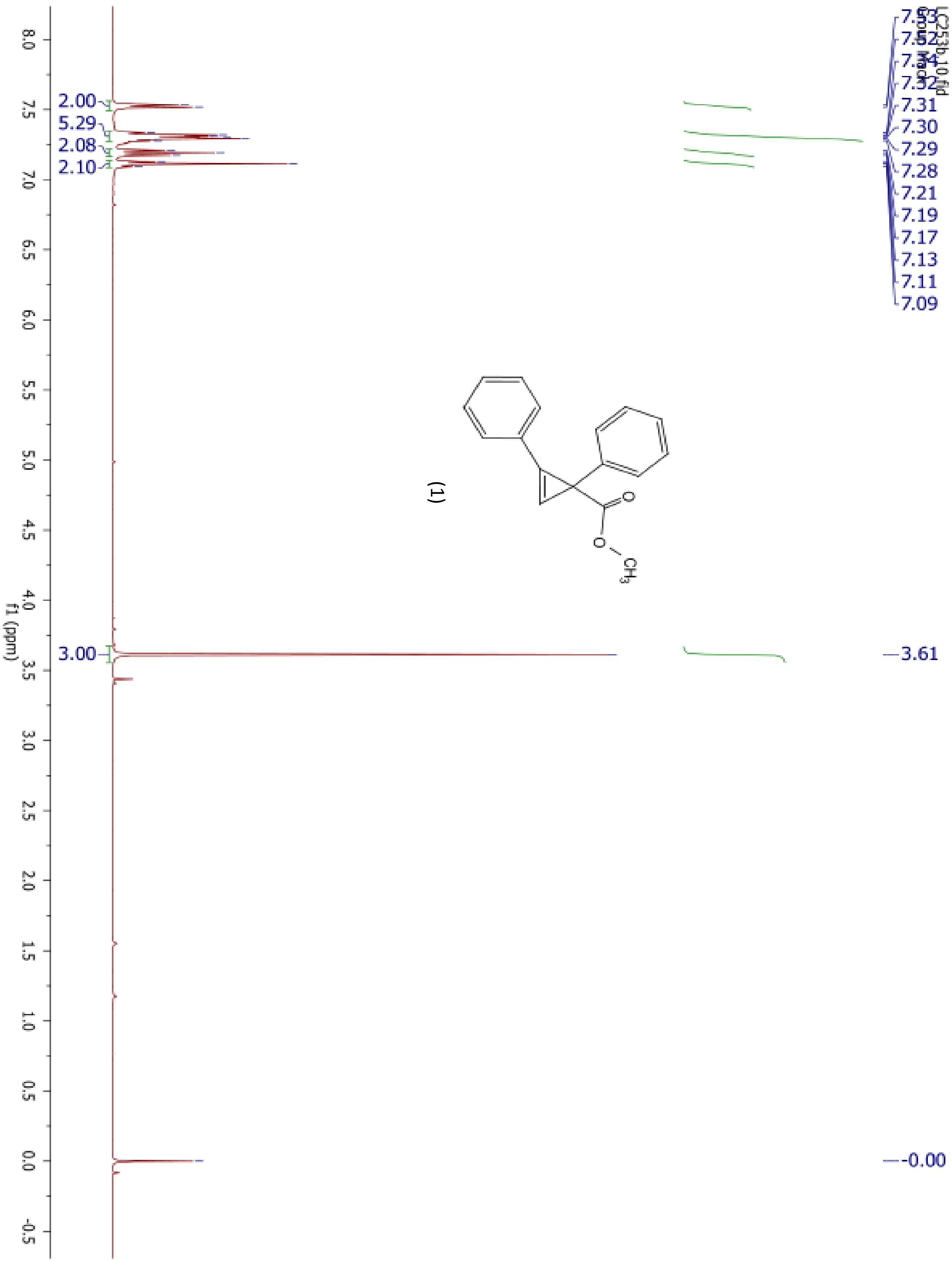

LC253b_10.fid
(1)
f1 (ppm)
7.53
7.52
7.34
7.32
7.31
7.30
7.29
7.28
7.21
7.19
7.17
7.13
7.11
7.09
3.61
-0.00
2.00
5.29
2.08
2.10
3.00

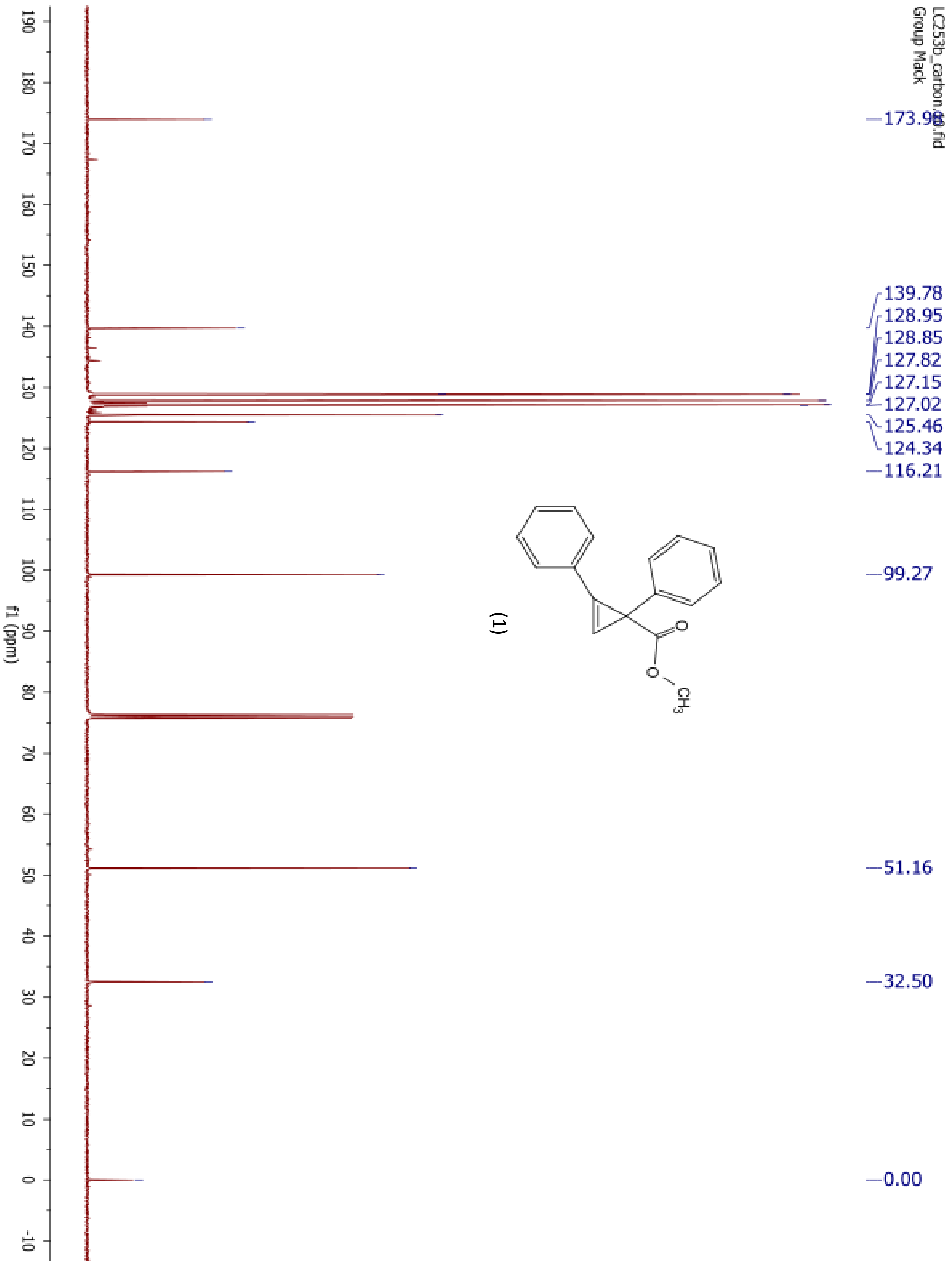
LC253b_carbon.[illegible].fid
Group Mack
173.9
139.78
128.95
128.85
127.82
127.15
127.02
125.46
124.34
116.21
99.27
51.16
32.50
0.00
(1)
CH3
O
O
190 180 170 160 150 140 130 120 110 100 90 80 70 60 50 40 30 20 10 0 -10
f1 (ppm)

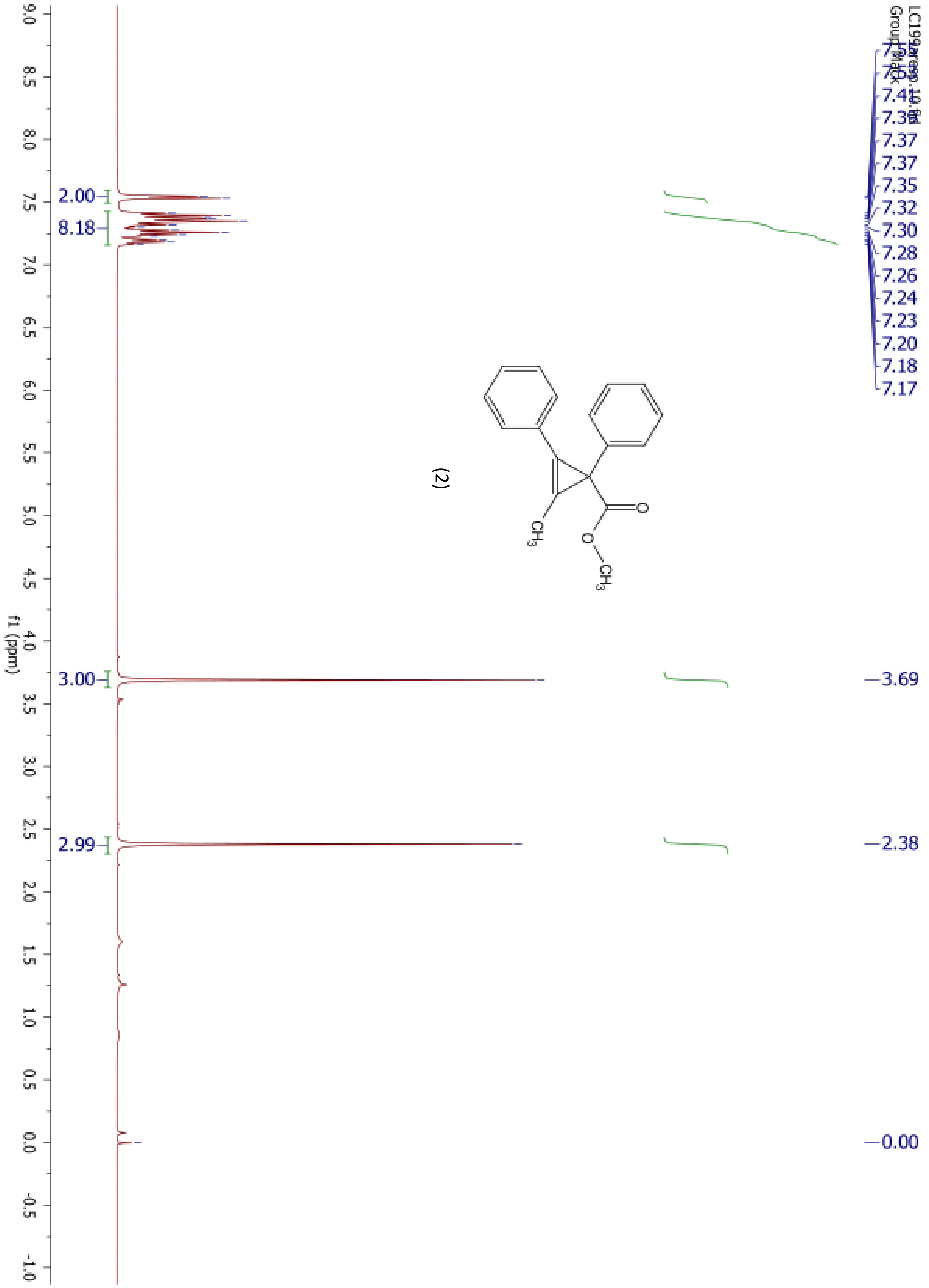

Group Mack
f1 (ppm)
2.00
8.18
3.00
2.99
7.41
7.37
7.37
7.35
7.32
7.30
7.28
7.26
7.24
7.23
7.20
7.18
7.17
3.69
2.38
0.00
(2)

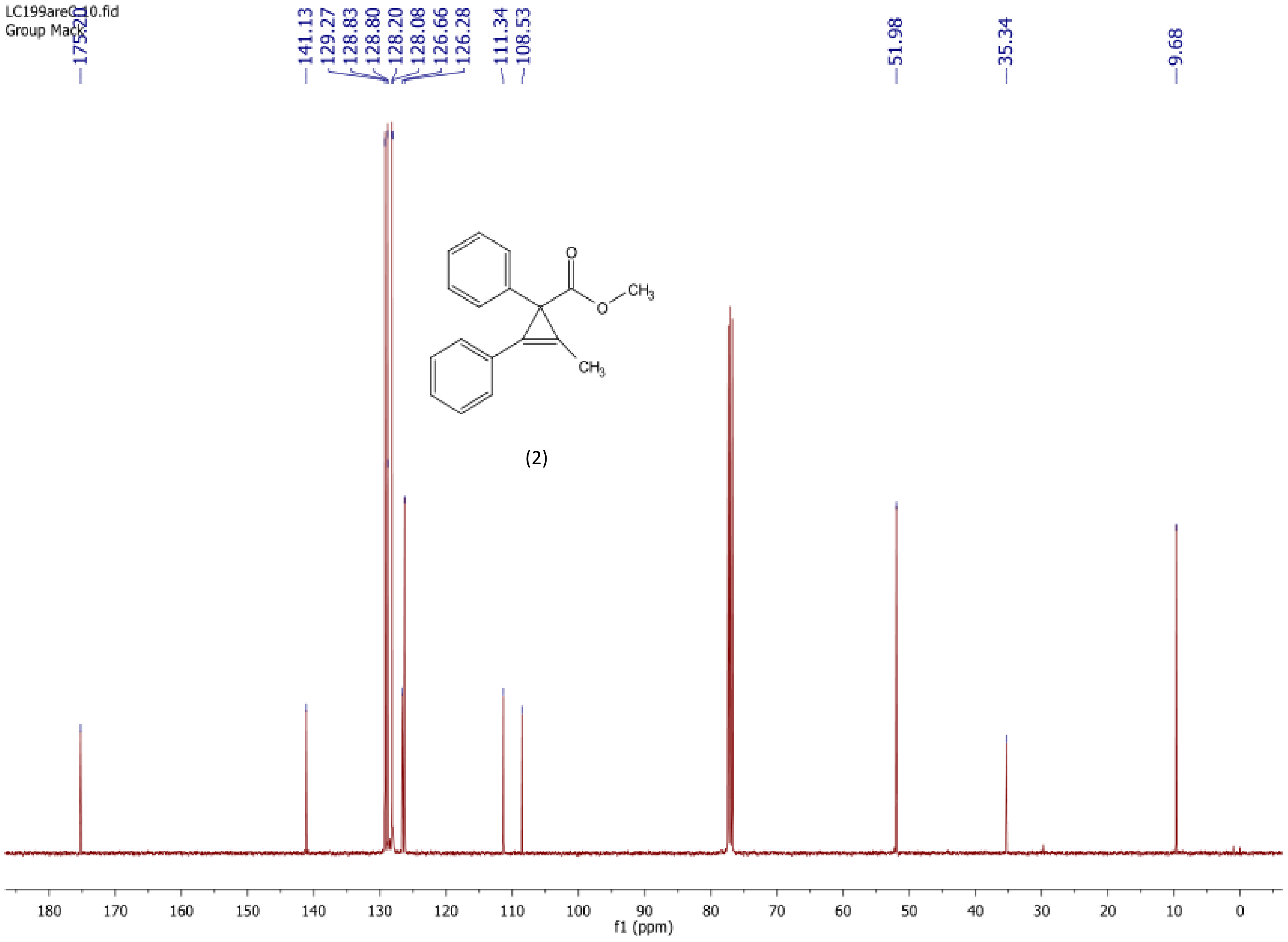
Group Mack
175.20
141.13
129.27
128.83
128.80
128.20
128.08
126.66
126.28
111.34
108.53
51.98
35.34
9.68
O
O
CH3
CH3
(2)
180 170 160 150 140 130 120 110 100 90 80 70 60 50 40 30 20 10 0
f1 (ppm)

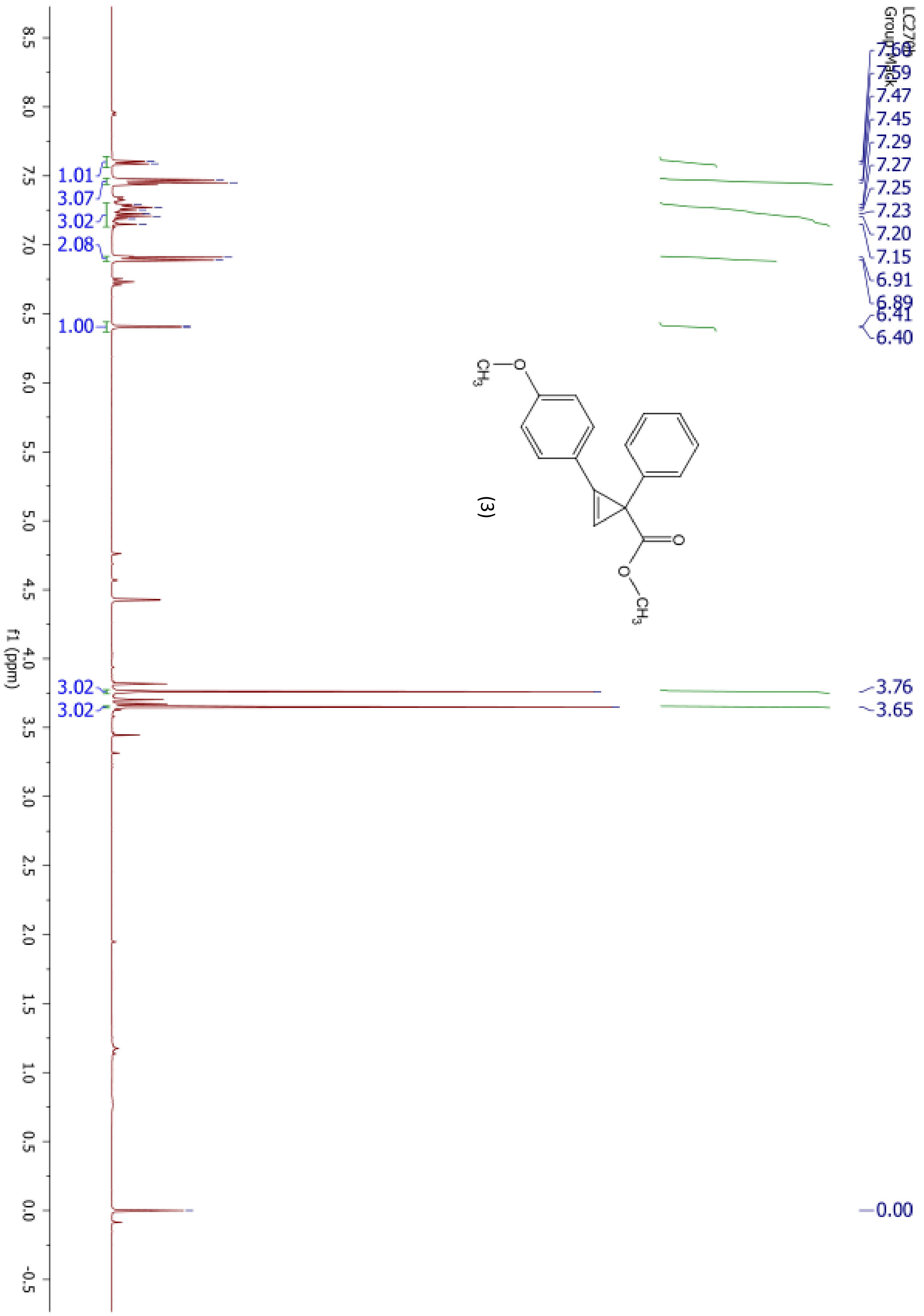

(3)
f1 (ppm)
1.01
3.07
3.02
2.08
1.00
3.02
3.02
7.60
7.59
7.47
7.45
7.29
7.27
7.25
7.23
7.20
7.15
6.91
6.89
6.41
6.40
3.76
3.65
0.00

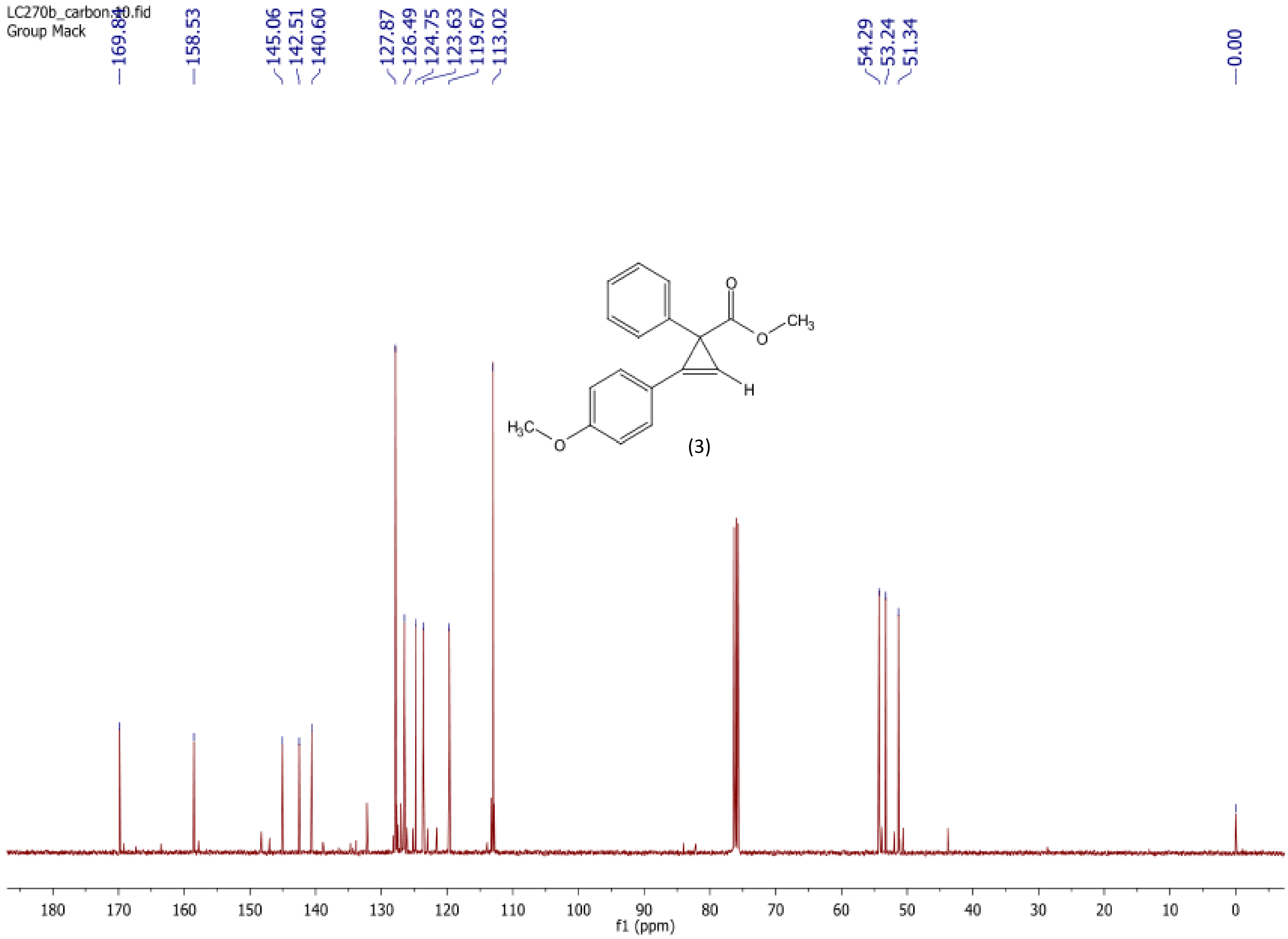
LC270b_carbon[illegible]0.fid
Group Mack
169.84
158.53
145.06
142.51
140.60
127.87
126.49
124.75
123.63
119.67
113.02
54.29
53.24
51.34
0.00
O
O
CH3
H
H3C
O
(3)
180
170
160
150
140
130
120
110
100
90
80
70
60
50
40
30
20
10
0
f1 (ppm)

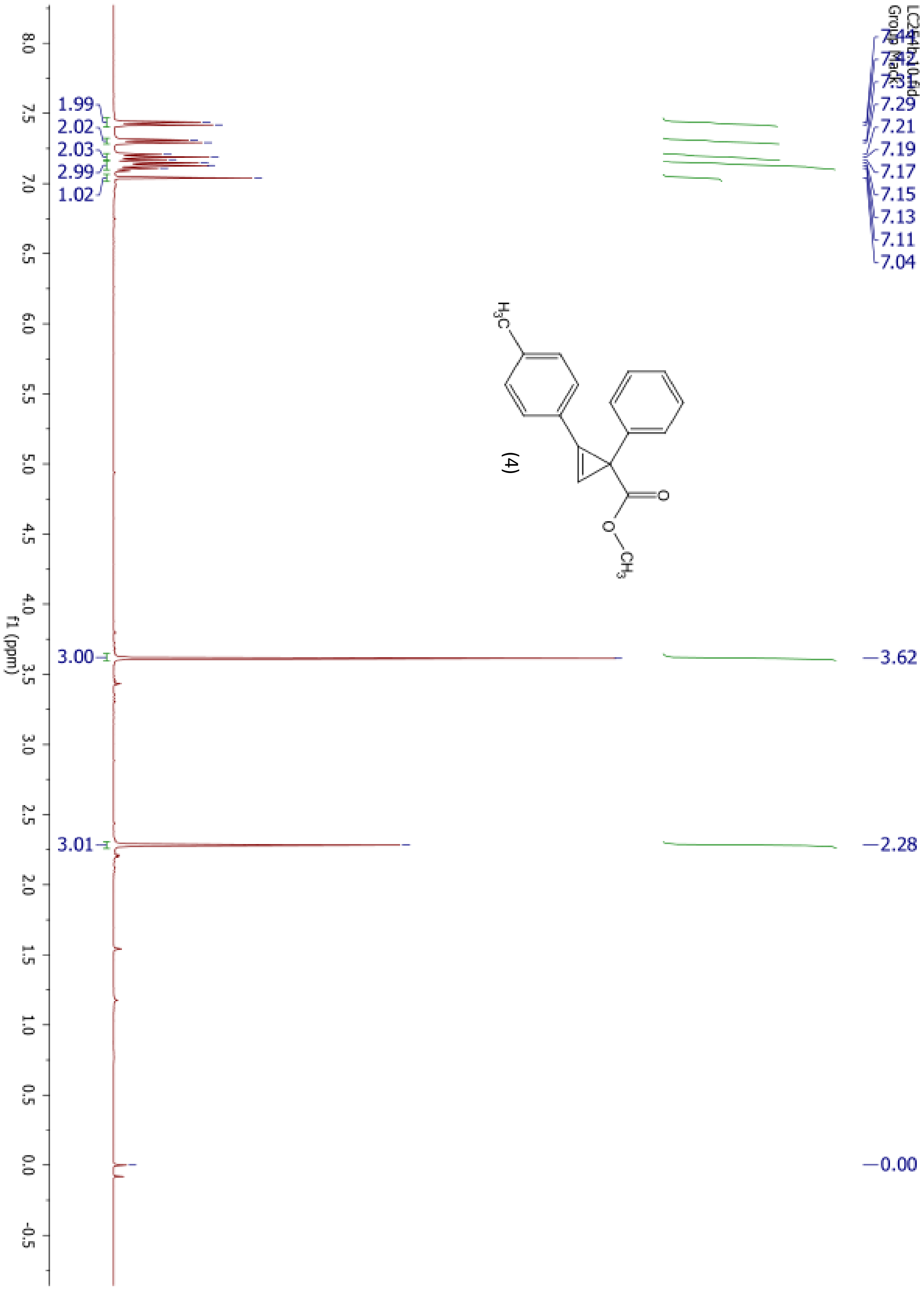
(4)
f1 (ppm)
7.44
7.29
7.21
7.19
7.17
7.15
7.13
7.11
7.04
3.62
2.28
0.00
1.99
2.02
2.03
2.99
1.02
3.00
3.01

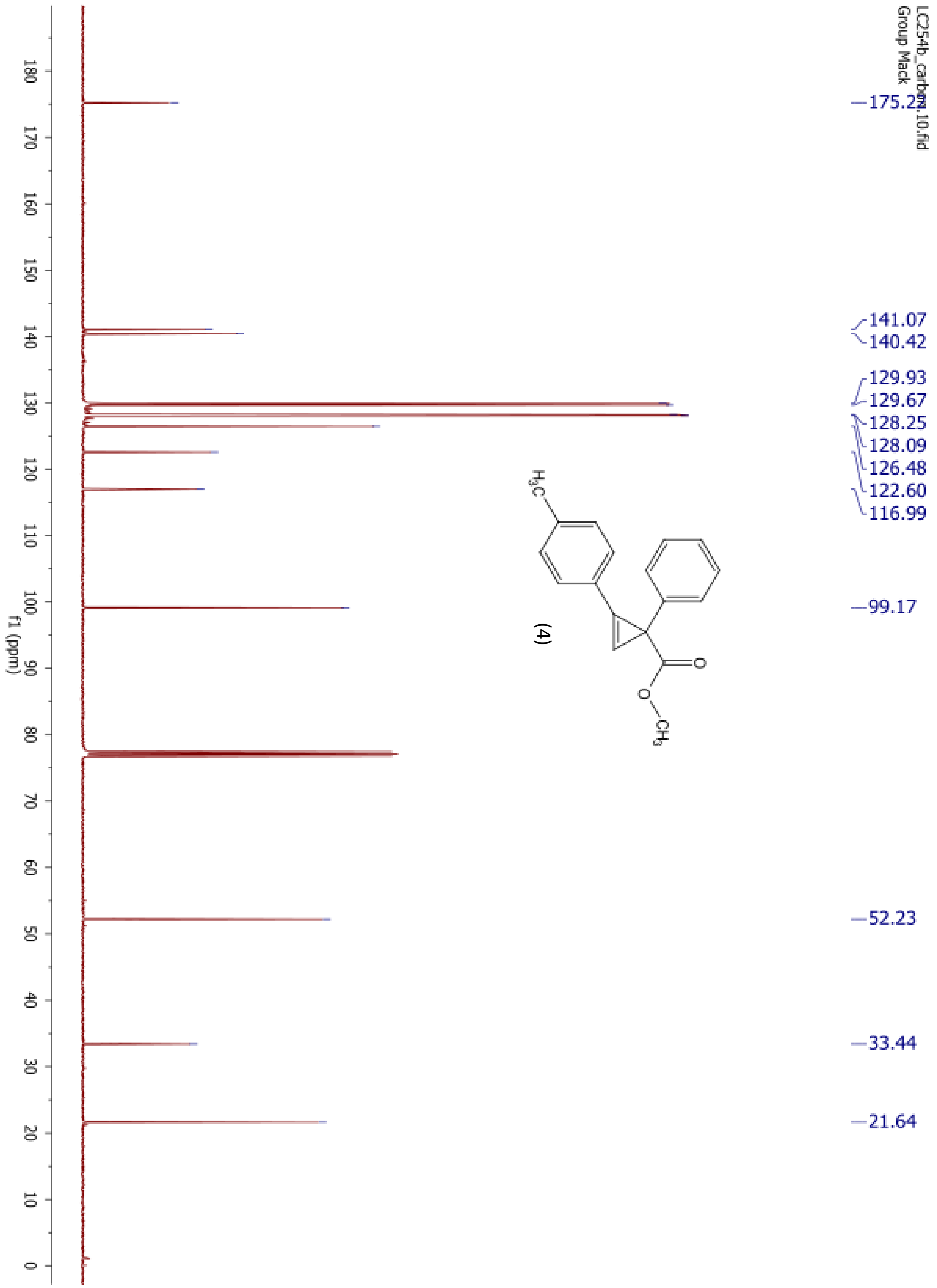

LC254b_carbon.10.fid
Group Mack
175.22
141.07
140.42
129.93
129.67
128.25
128.09
126.48
122.60
116.99
99.17
52.23
33.44
21.64
(4)
f1 (ppm)

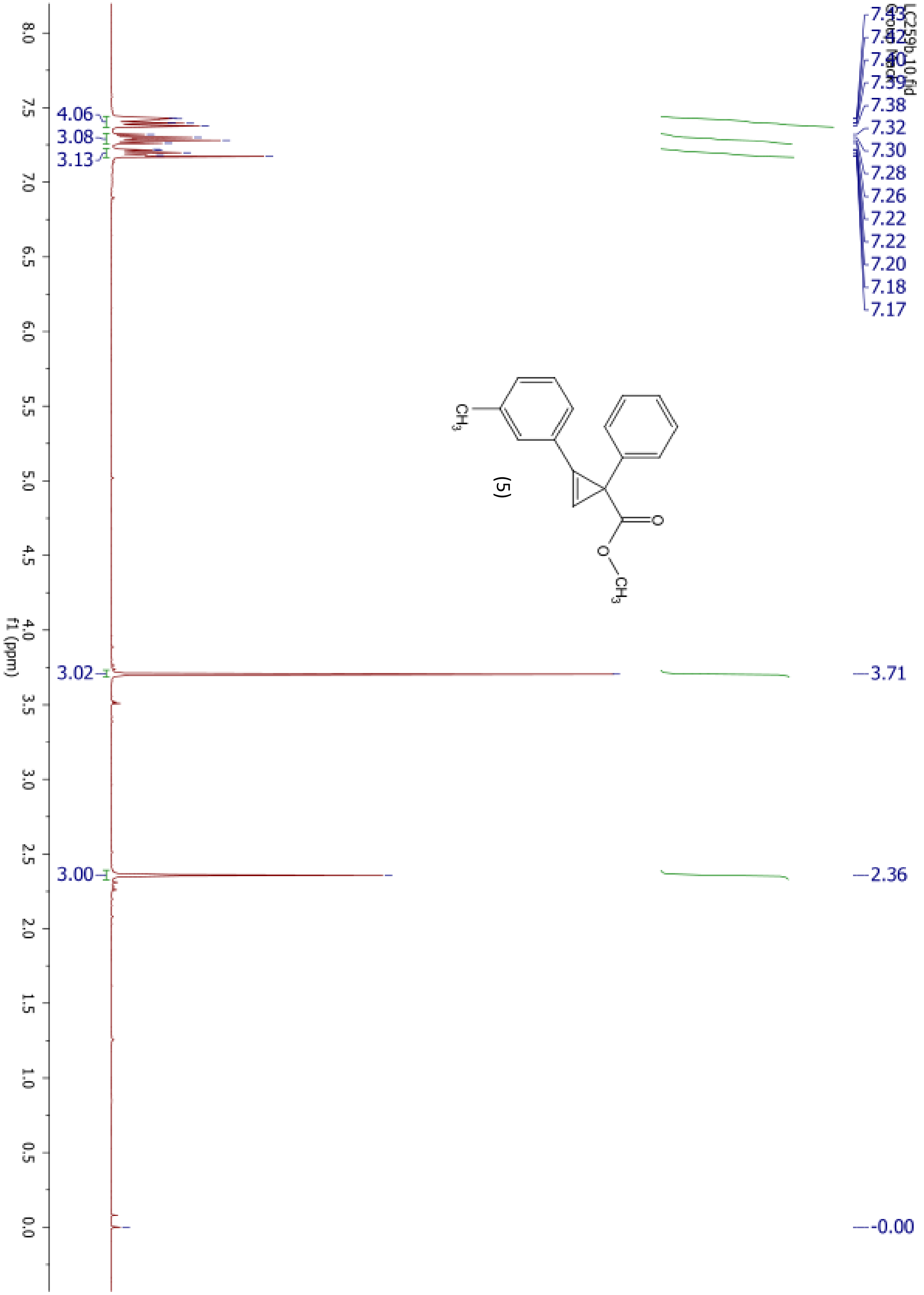

7.43
7.42
7.40
7.39
7.38
7.32
7.30
7.28
7.26
7.22
7.22
7.20
7.18
7.17
3.71
2.36
-0.00
4.06
3.08
3.13
3.02
3.00
f1 (ppm)
(5)
CH3
O
O
CH3

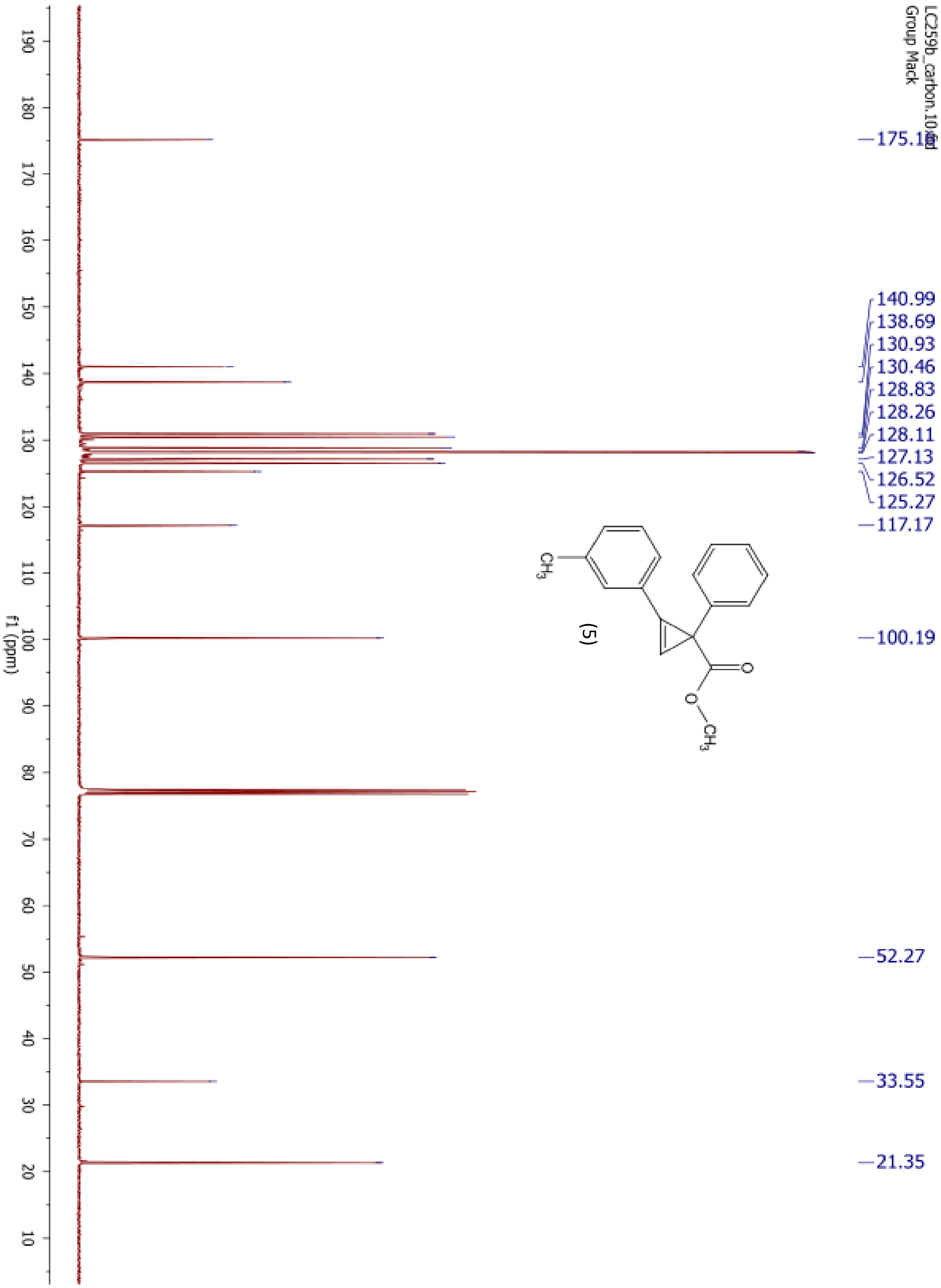
LC259b_carbon.10.fid
Group Mack
175.18
140.99
138.69
130.93
130.46
128.83
128.26
128.11
127.13
126.52
125.27
117.17
100.19
52.27
33.55
21.35
(5)
f1 (ppm)
190
180
170
160
150
140
130
120
110
100
90
80
70
60
50
40
30
20
10

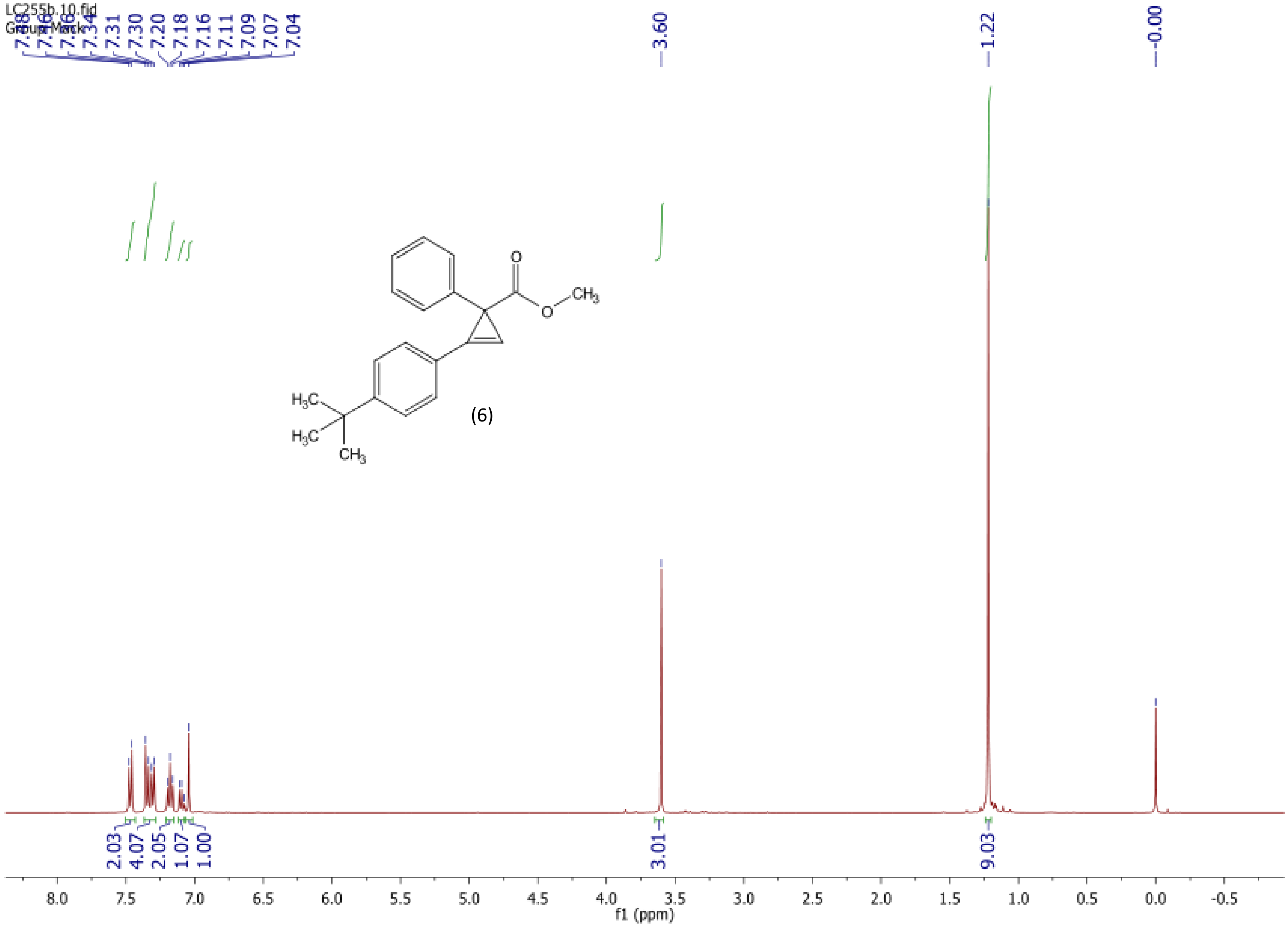
LC255b.10.fid
7.34
7.31
7.30
7.20
7.18
7.16
7.11
7.09
7.07
7.04
3.60
1.22
-0.00
H3C
H3C
CH3
O
O
CH3
(6)
2.03
4.07
2.05
1.07
1.00
3.01
9.03
8.0
7.5
7.0
6.5
6.0
5.5
5.0
4.5
4.0
3.5
3.0
2.5
2.0
1.5
1.0
0.5
0.0
-0.5
f1 (ppm)

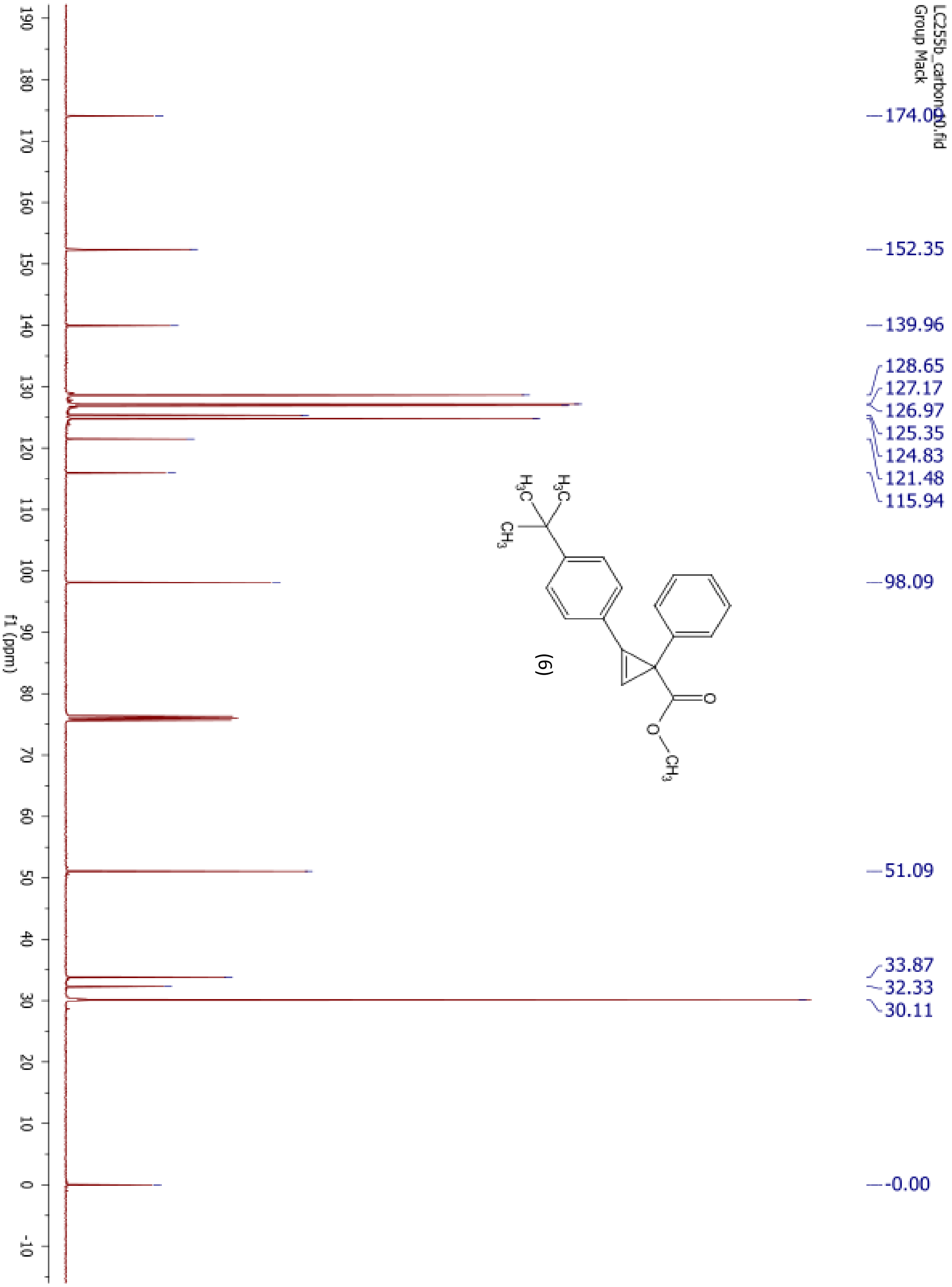
LC255b_carbon00.fid
Group Mack
174.00
152.35
139.96
128.65
127.17
126.97
125.35
124.83
121.48
115.94
98.09
51.09
33.87
32.33
30.11
-0.00
(6)
f1 (ppm)

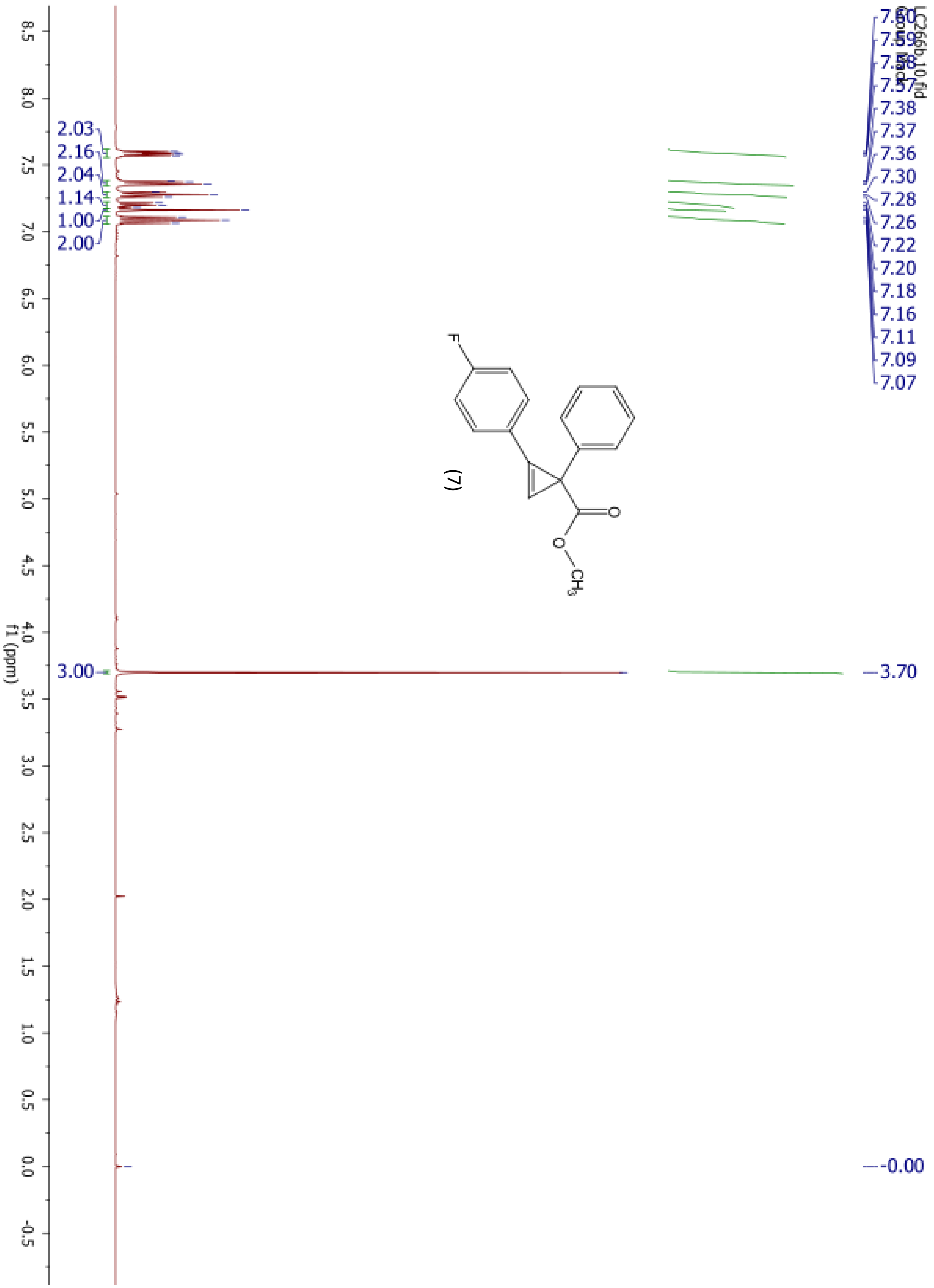
LC266b.10.fid
7.60
7.59
7.58
7.57
7.38
7.37
7.36
7.30
7.28
7.26
7.22
7.20
7.18
7.16
7.11
7.09
7.07
3.70
-0.00
2.03
2.16
2.04
1.14
1.00
2.00
3.00
(7)
F
O
CH3
f1 (ppm)

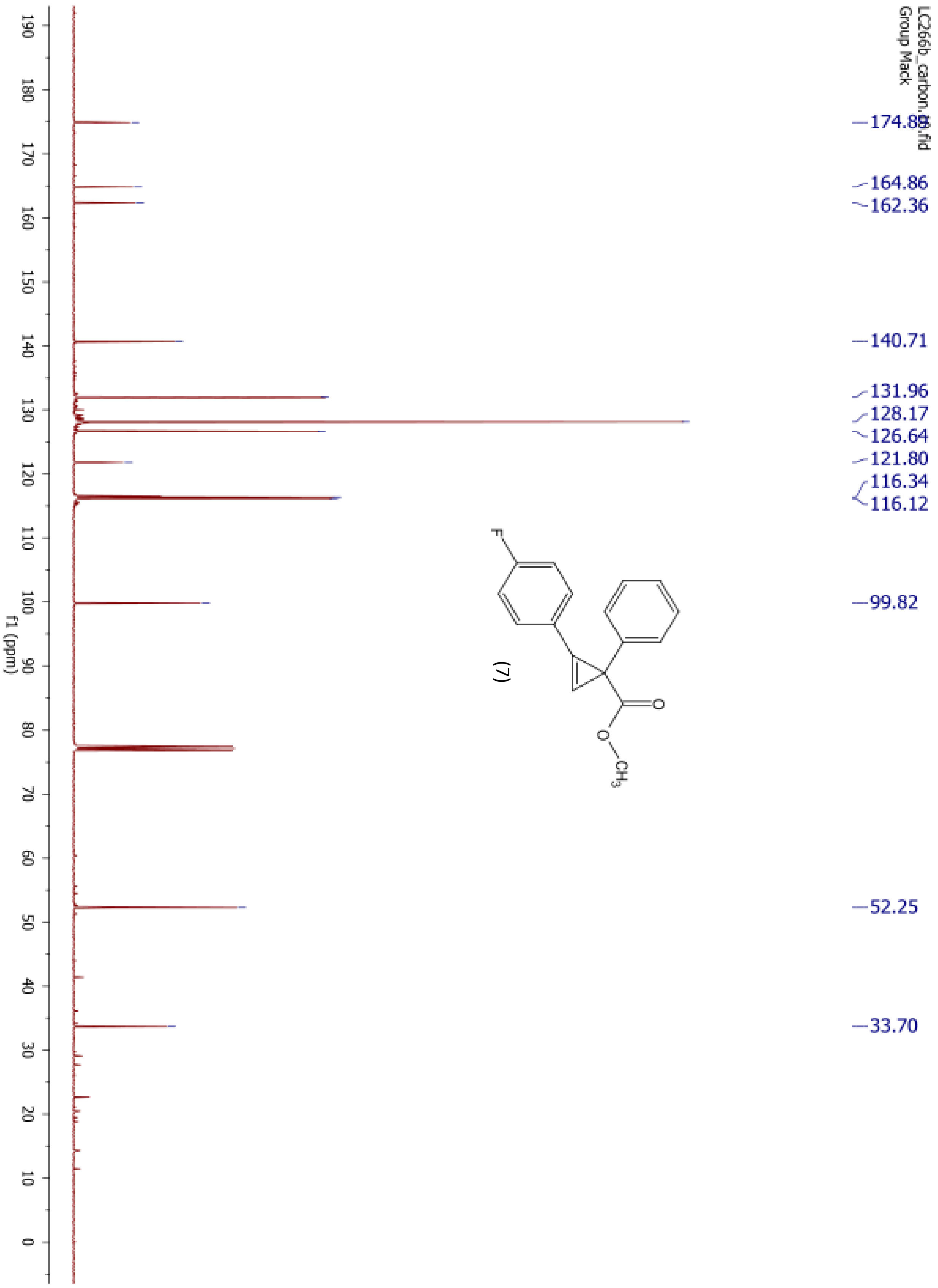
Group Mack
174.88
164.86
162.36
140.71
131.96
128.17
126.64
121.80
116.34
116.12
99.82
52.25
33.70
(7)
F
O
O
CH3
f1 (ppm)
190
180
170
160
150
140
130
120
110
100
90
80
70
60
50
40
30
20
10
0

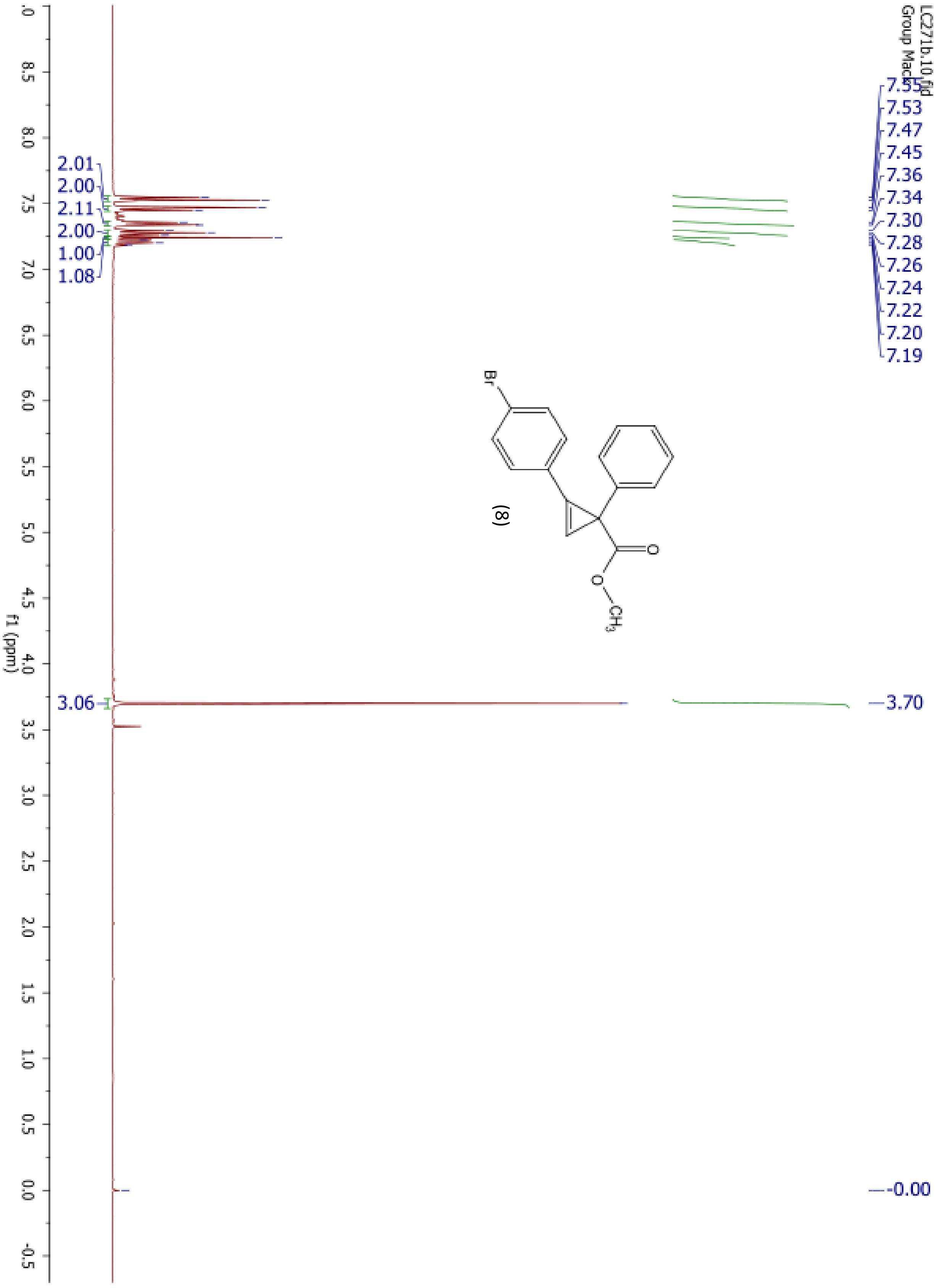
LC271b.10.fid
Group Mack
7.55
7.53
7.47
7.45
7.36
7.34
7.30
7.28
7.26
7.24
7.22
7.20
7.19
3.70
-0.00
2.01
2.00
2.11
2.00
1.00
1.08
3.06
(8)
Br
O
O
CH3
f1 (ppm)

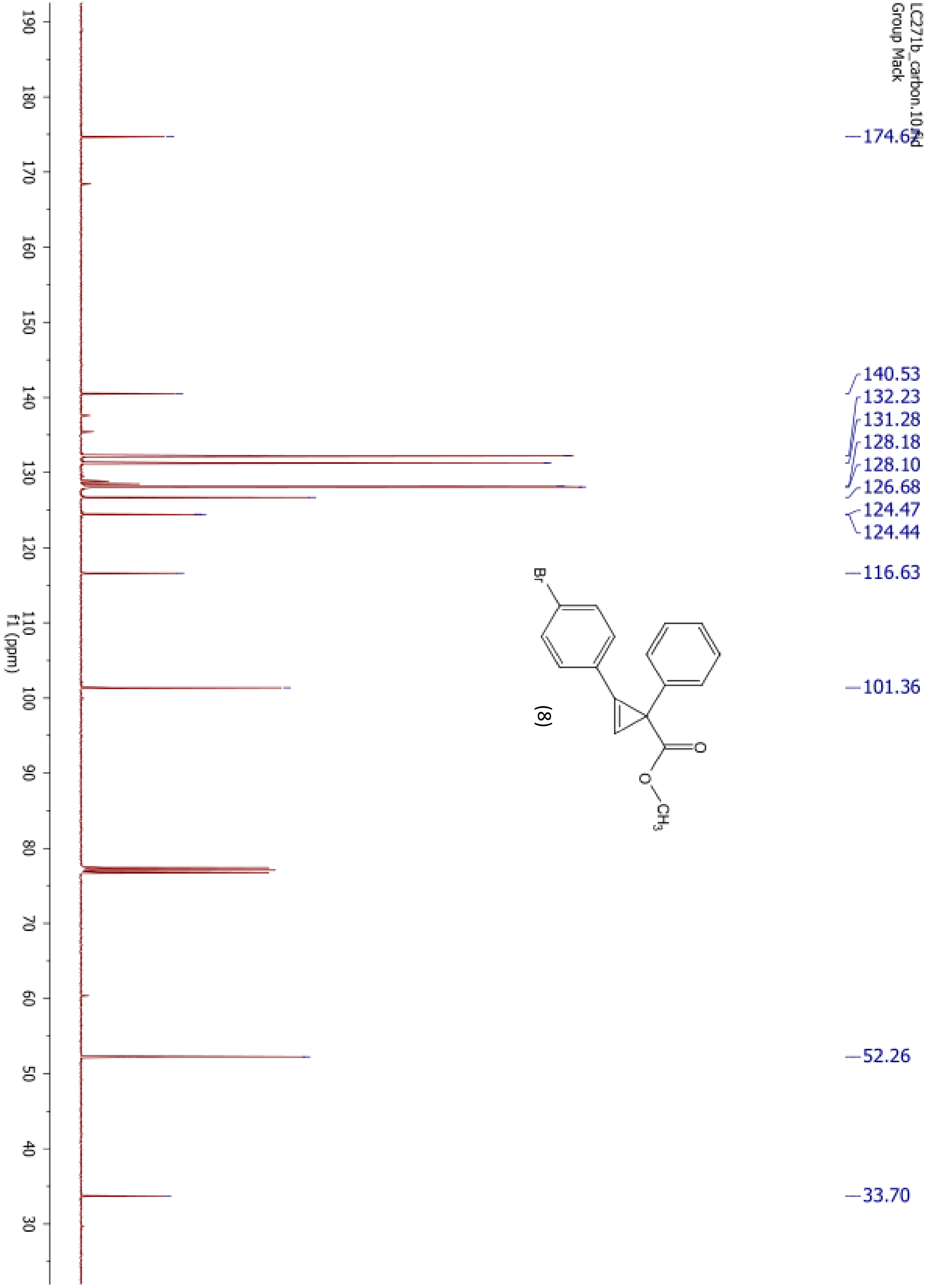
LC271b_carbon.10.fid
Group Mack
140.53
132.23
131.28
128.18
128.10
126.68
124.47
124.44
116.63
101.36
52.26
33.70
Br
(8)
O
O
CH3
f1 (ppm)

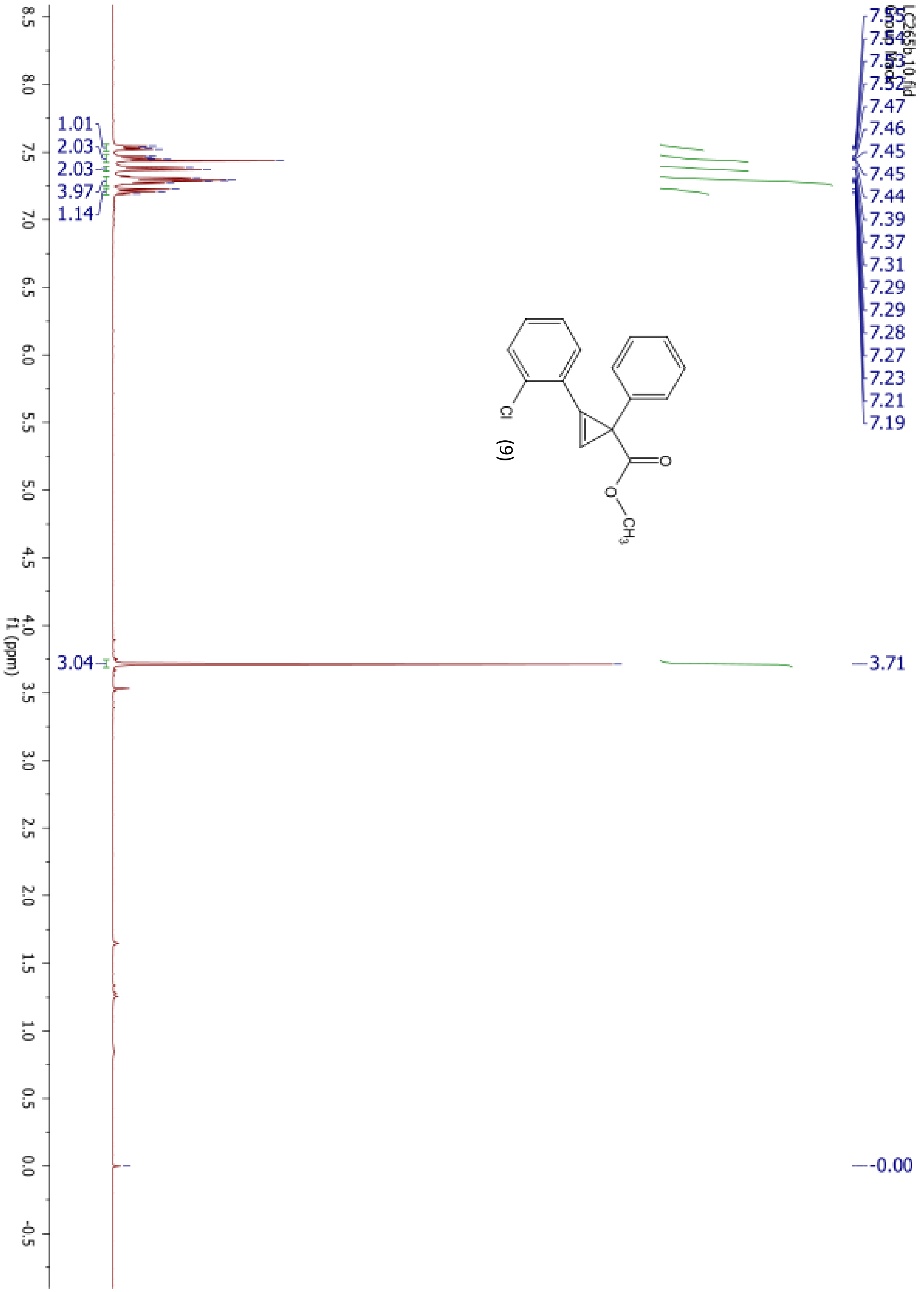

LC265b.10.fid
7.55
7.54
7.53
7.52
7.47
7.46
7.45
7.45
7.44
7.39
7.37
7.31
7.29
7.29
7.28
7.27
7.23
7.21
7.19
3.71
-0.00
1.01
2.03
2.03
3.97
1.14
3.04
f1 (ppm)
Cl
O
O
CH3
(9)

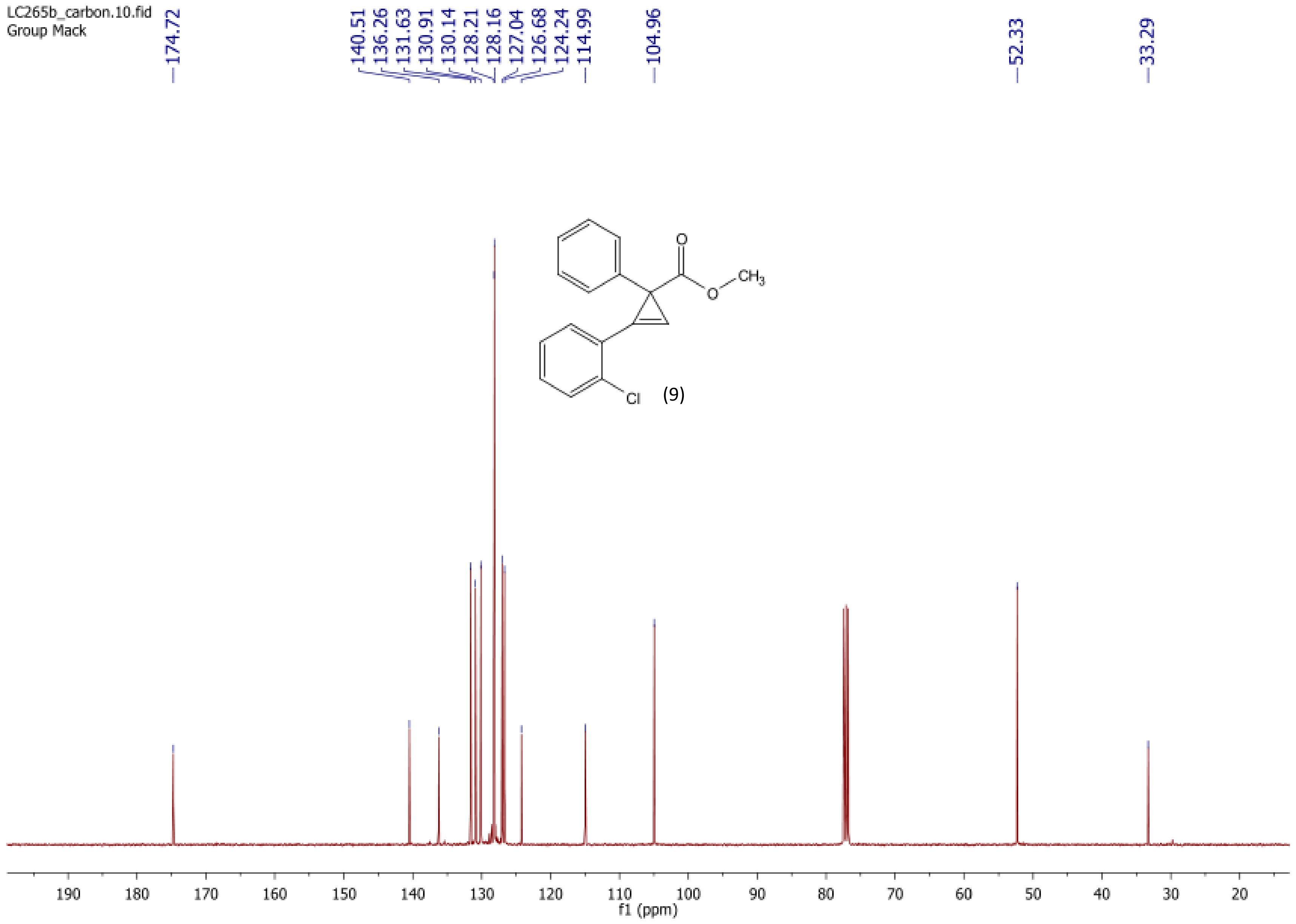
LC265b_carbon.10.fid
Group Mack
174.72
140.51
136.26
131.63
130.91
130.14
128.21
128.16
127.04
126.68
124.24
114.99
104.96
52.33
33.29
O
O
CH3
Cl
(9)
190
180
170
160
150
140
130
120
110
100
90
80
70
60
50
40
30
20
f1 (ppm)

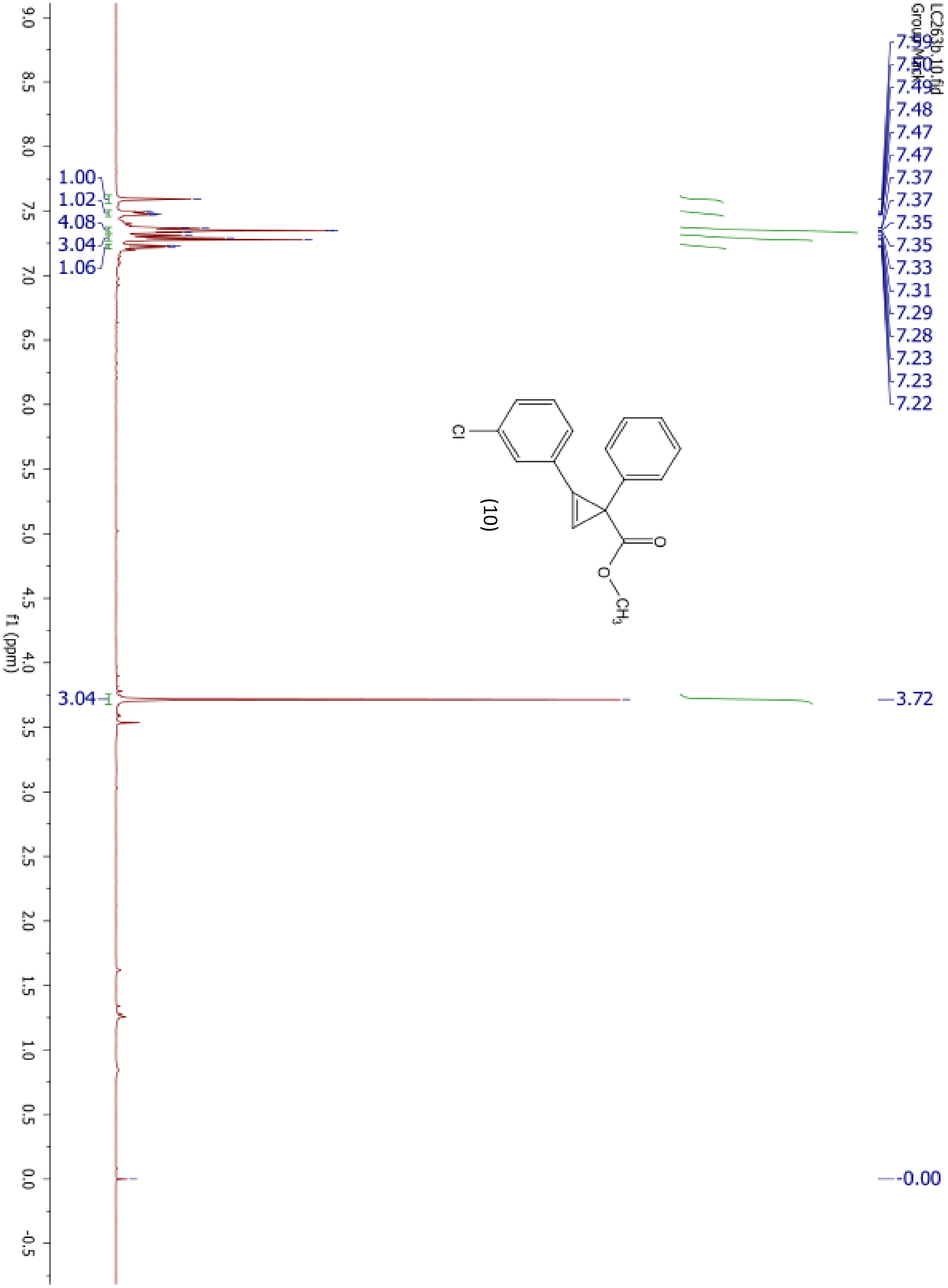

LC263b.10.fid
Group Mack
7.[illegible]9
7.50
7.49
7.48
7.47
7.47
7.37
7.37
7.35
7.35
7.33
7.31
7.29
7.28
7.23
7.23
7.22
3.72
-0.00
1.00
1.02
4.08
3.04
1.06
3.04
Cl
O
O
CH3
(10)
f1 (ppm)
9.0
8.5
8.0
7.5
7.0
6.5
6.0
5.5
5.0
4.5
4.0
3.5
3.0
2.5
2.0
1.5
1.0
0.5
0.0
-0.5

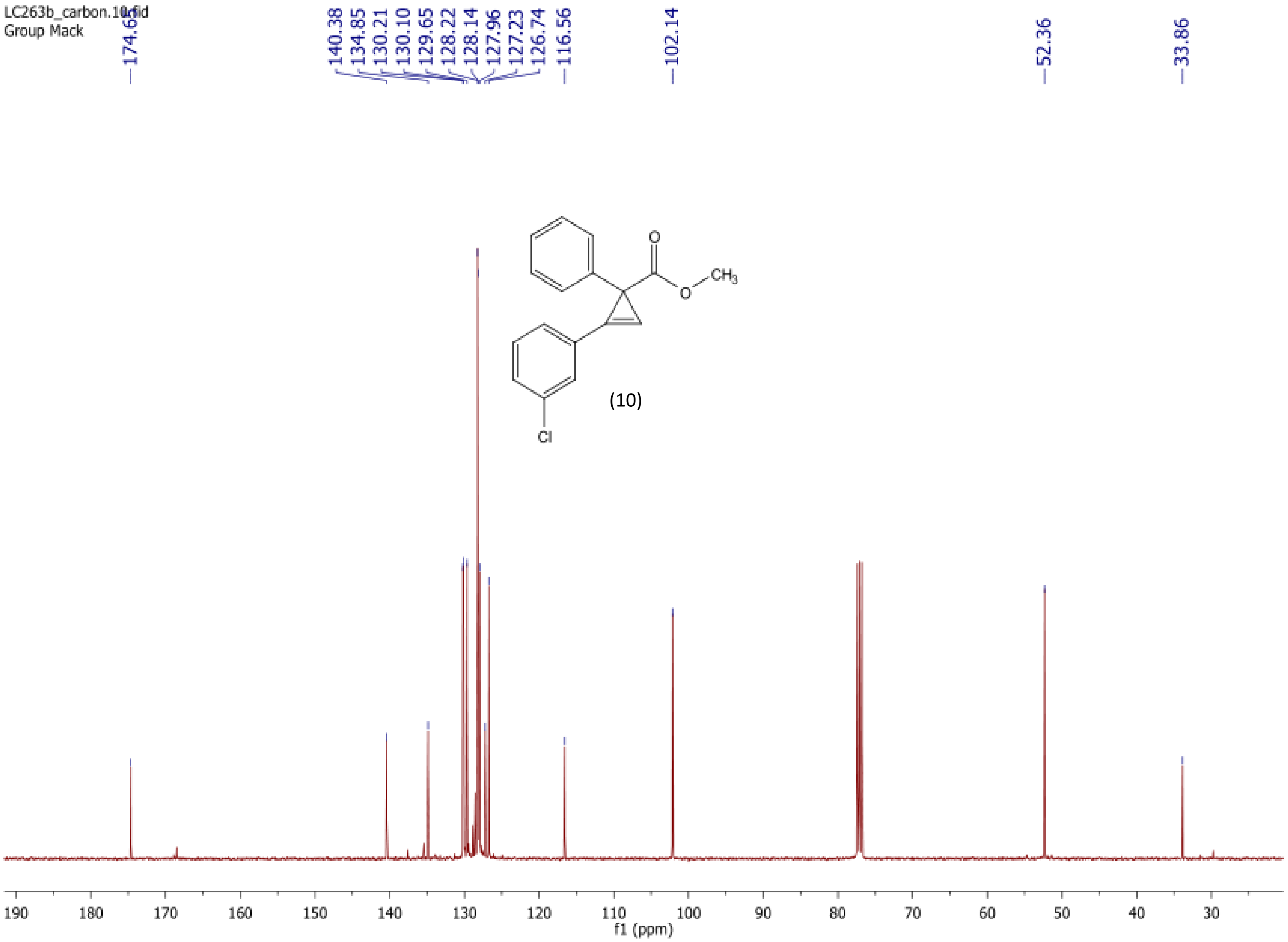
LC263b_carbon.10.fid
Group Mack
174.65
140.38
134.85
130.21
130.10
129.65
128.22
128.14
127.96
127.23
126.74
116.56
102.14
52.36
33.86
O
O
CH3
Cl
190
180
170
160
150
140
130
120
110
100
90
80
70
60
50
40
30
f1 (ppm)

(10)

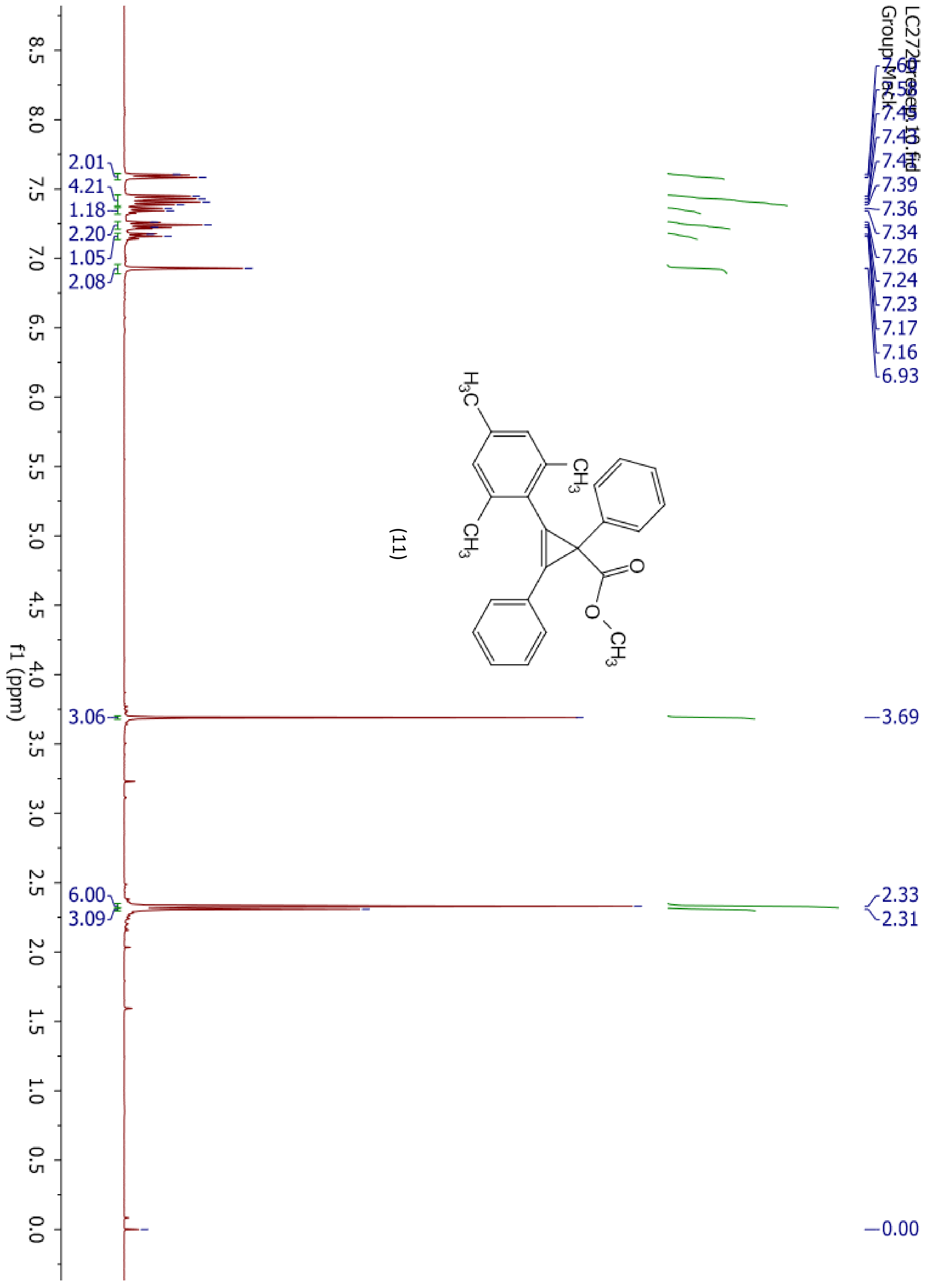
LC272bresep.10.fid
Group Mack
7.39
7.36
7.34
7.26
7.24
7.23
7.17
7.16
6.93
3.69
2.33
2.31
0.00
2.01
4.21
1.18
2.20
1.05
2.08
3.06
6.00
3.09
CH3
H3C
CH3
O
O
CH3
(11)
f1 (ppm)
8.5
8.0
7.5
7.0
6.5
6.0
5.5
5.0
4.5
4.0
3.5
3.0
2.5
2.0
1.5
1.0
0.5
0.0

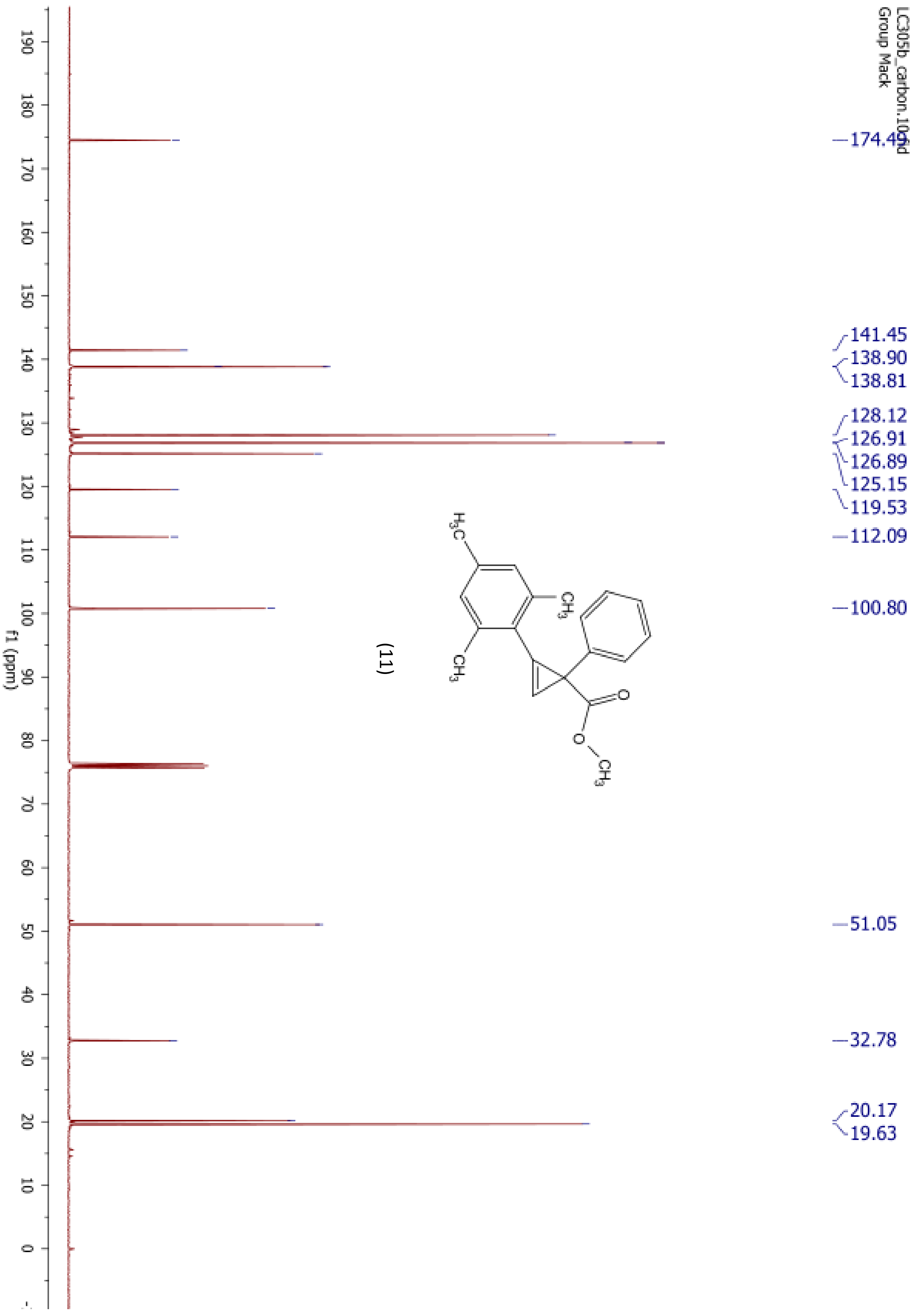
LC305b_carbon.10.fid
Group Mack
174.49
141.45
138.90
138.81
128.12
126.91
126.89
125.15
119.53
112.09
100.80
51.05
32.78
20.17
19.63
f1 (ppm)
H3C
CH3
CH3
O
O
CH3

(11)

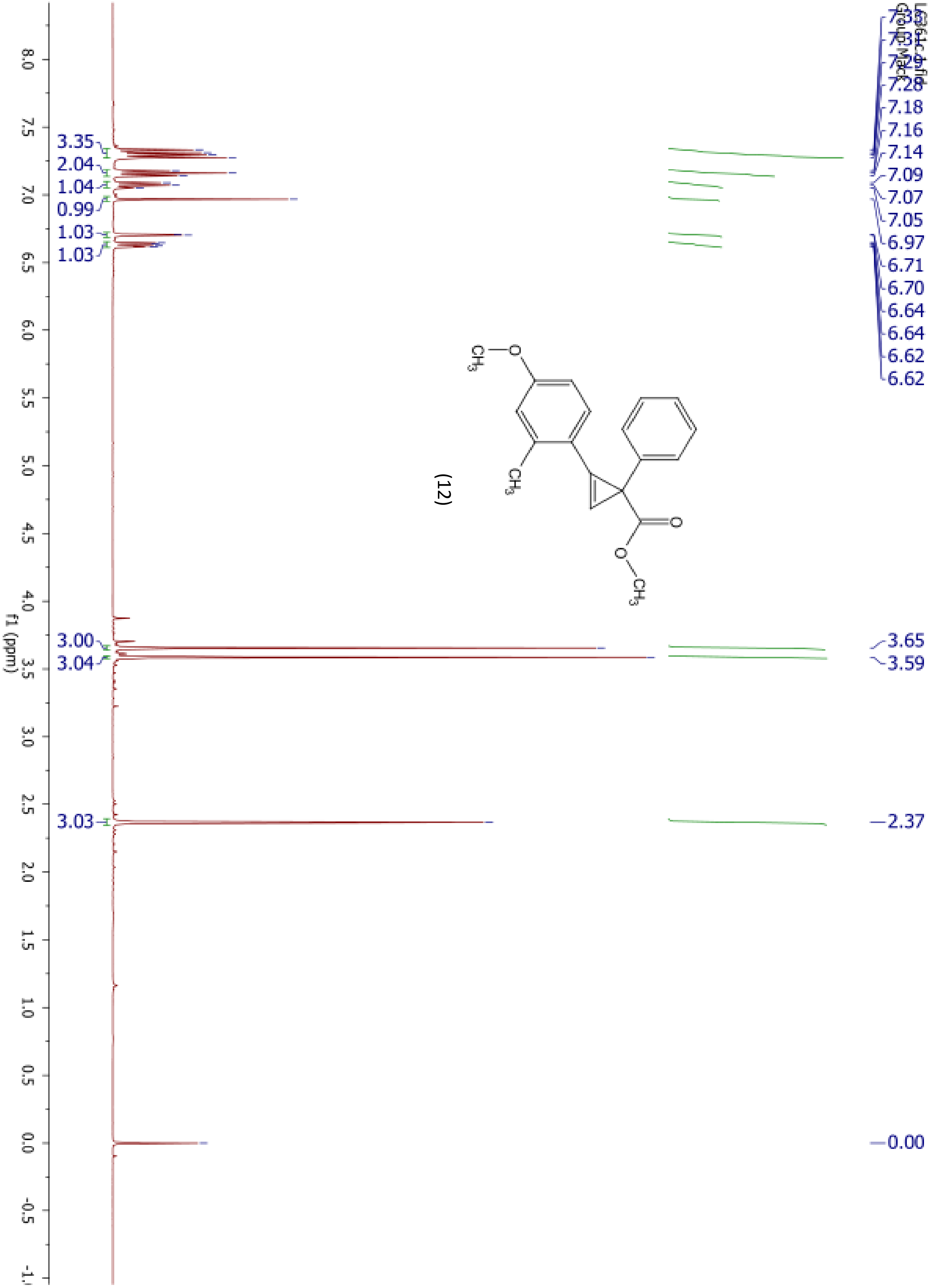
7.33
7.31
7.29
7.28
7.18
7.16
7.14
7.09
7.07
7.05
6.97
6.71
6.70
6.64
6.64
6.62
6.62
3.65
3.59
2.37
0.00
3.35
2.04
1.04
0.99
1.03
1.03
3.00
3.04
3.03
f1 (ppm)
CH3
O
CH3
O
O
CH3

(12)

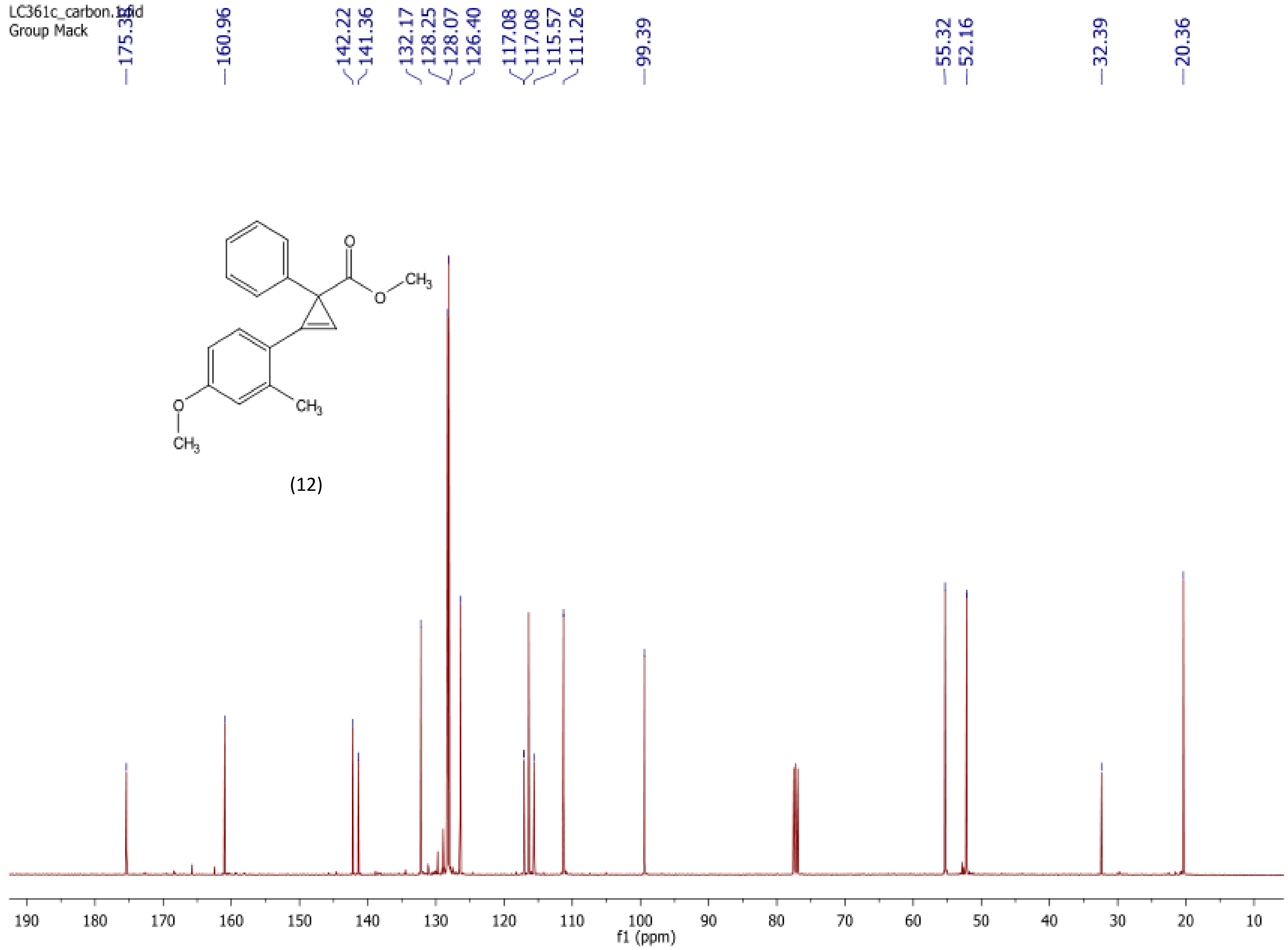
LC361c_carbon.fid
Group Mack
175.36
160.96
142.22
141.36
132.17
128.25
128.07
126.40
117.08
117.08
115.57
111.26
99.39
55.32
52.16
32.39
20.36
O
CH3
O
CH3
CH3
O
CH3
190 180 170 160 150 140 130 120 110 100 90 80 70 60 50 40 30 20 10
f1 (ppm)

(12)

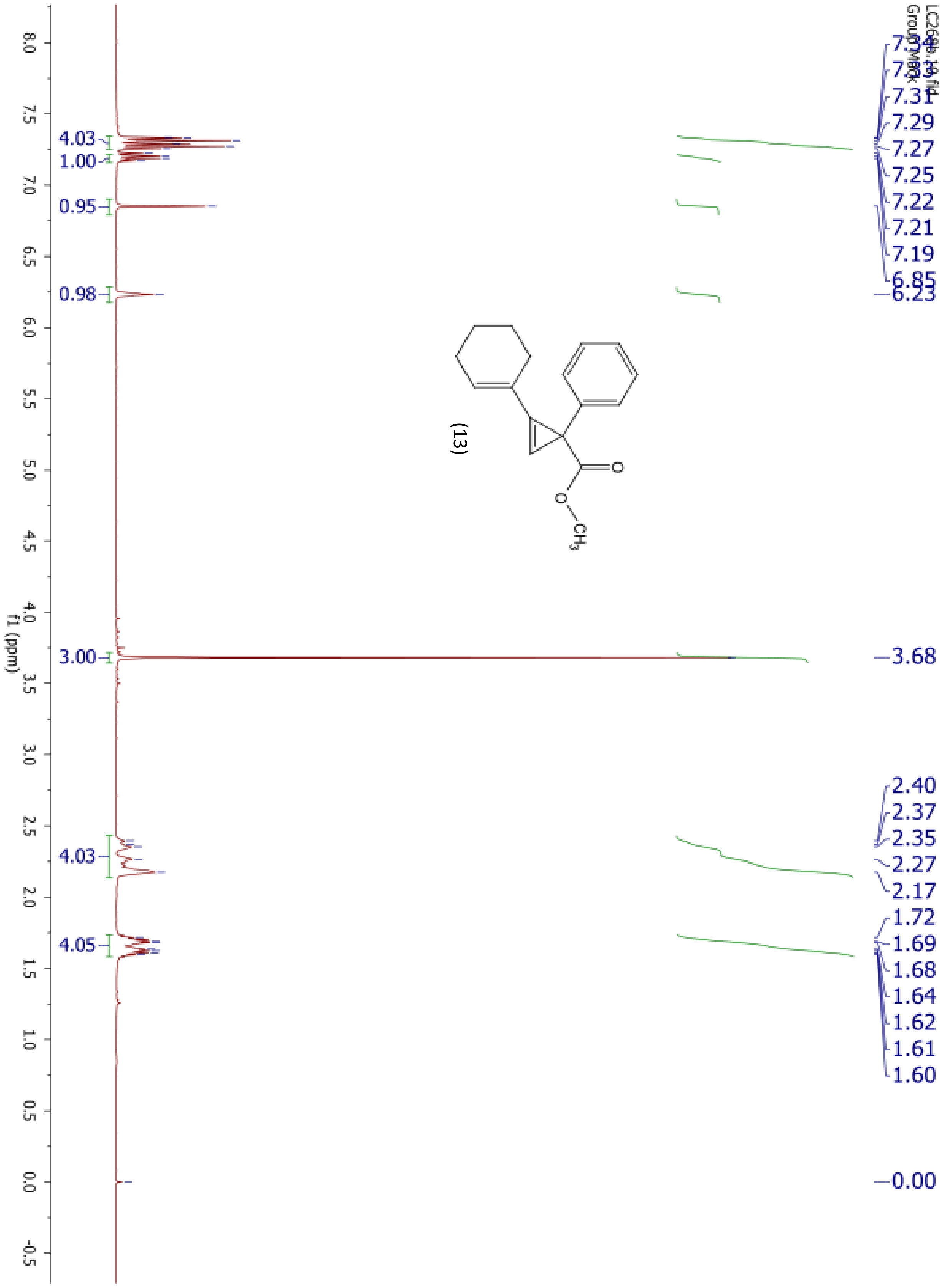
(13)
O
O
CH3
7.31
7.29
7.27
7.25
7.22
7.21
7.19
6.85
6.23
3.68
2.40
2.37
2.35
2.27
2.17
1.72
1.69
1.68
1.64
1.62
1.61
1.60
-0.00
4.03
1.00
0.95
0.98
3.00
4.03
4.05
f1 (ppm)
8.0
7.5
7.0
6.5
6.0
5.5
5.0
4.5
4.0
3.5
3.0
2.5
2.0
1.5
1.0
0.5
0.0
-0.5

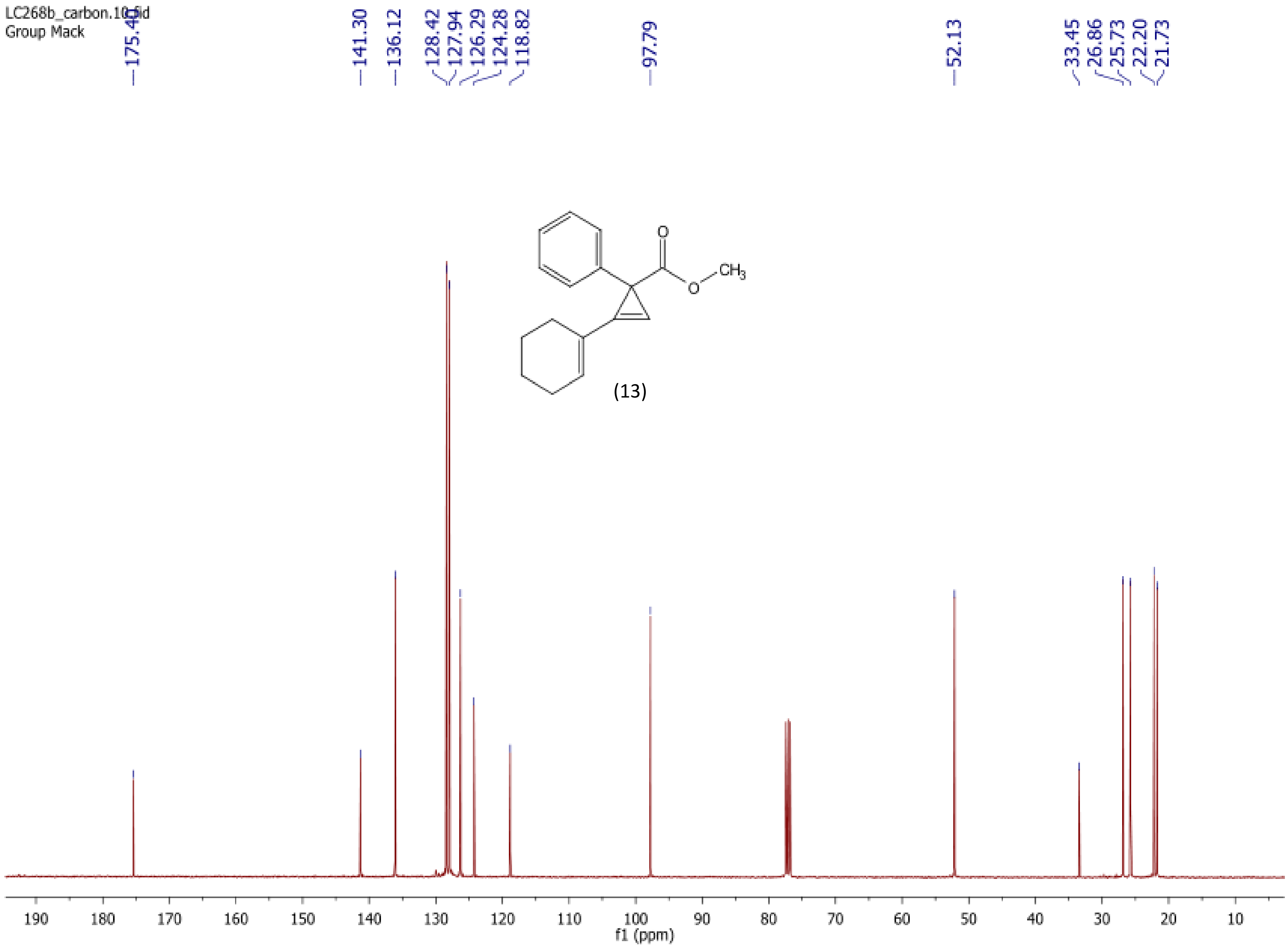
LC268b_carbon.10.fid
Group Mack
175.40
141.30
136.12
128.42
127.94
126.29
124.28
118.82
97.79
52.13
33.45
26.86
25.73
22.20
21.73
O
O
CH3
(13)
190
180
170
160
150
140
130
120
110
100
90
80
70
60
50
40
30
20
10
f1 (ppm)

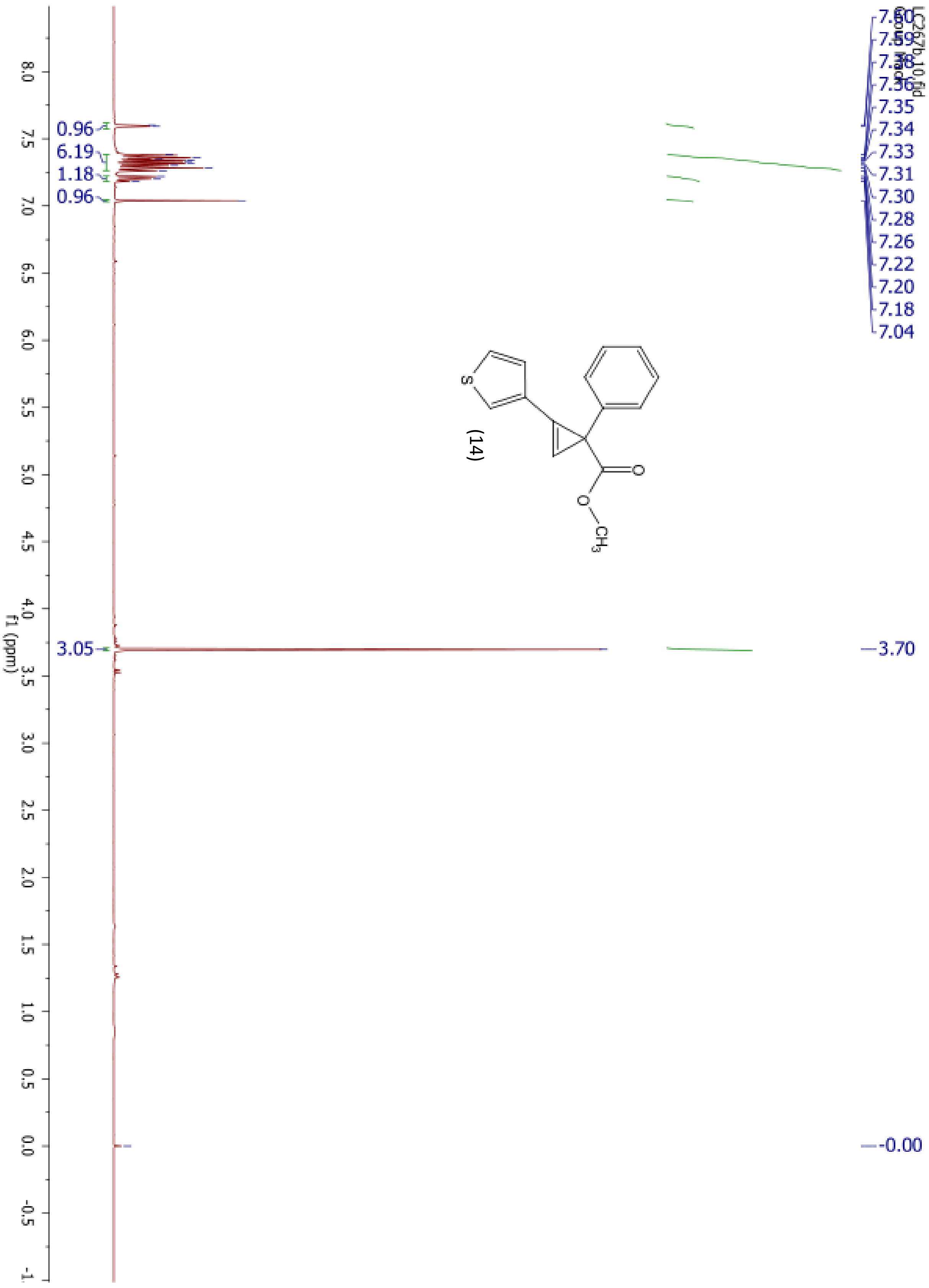
7.60
7.59
7.38
7.36
7.35
7.34
7.33
7.31
7.30
7.28
7.26
7.22
7.20
7.18
7.04
3.70
-0.00
0.96
6.19
1.18
0.96
3.05
f1 (ppm)
(14)

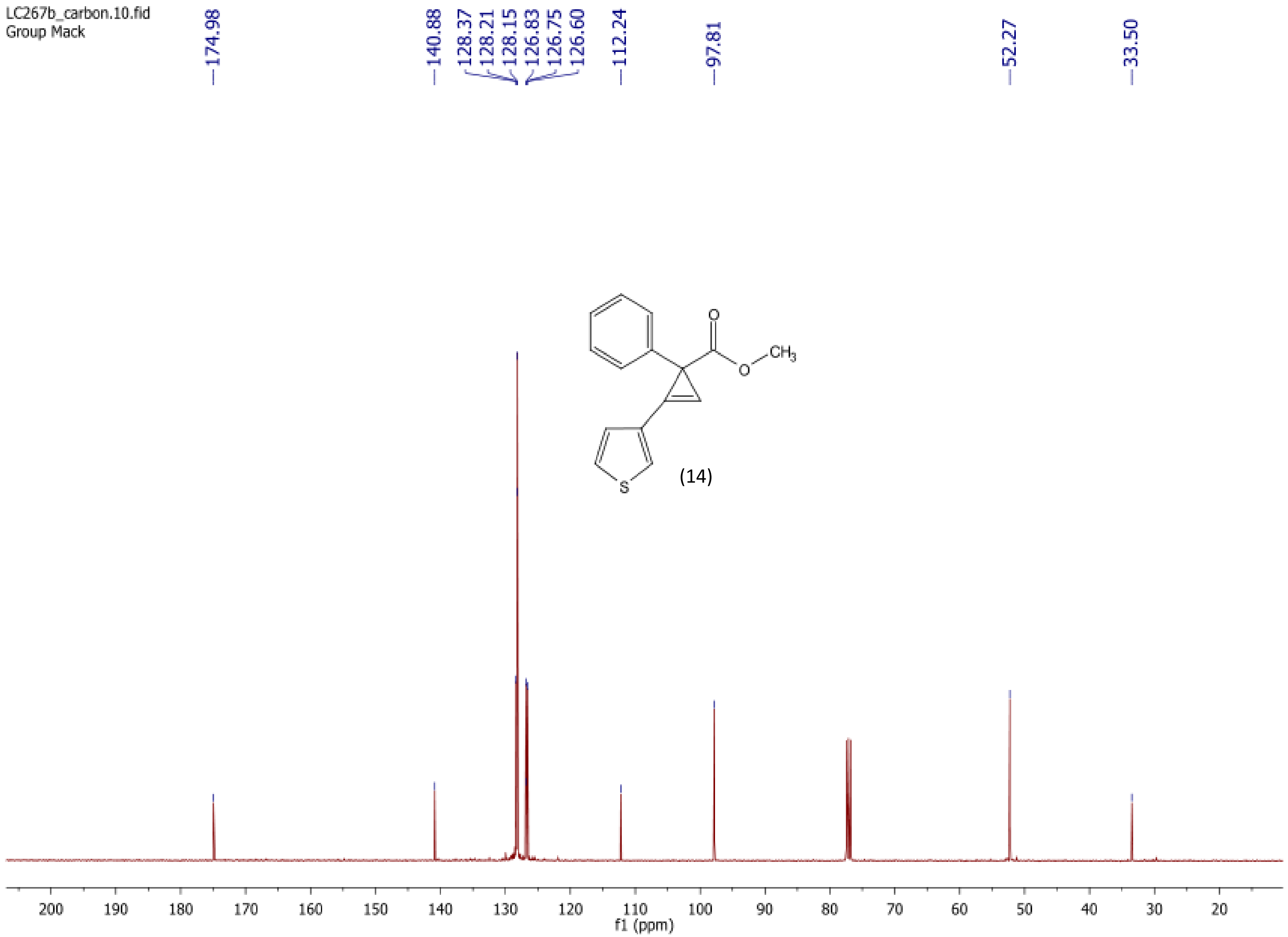
LC267b_carbon.10.fid
Group Mack
174.98
140.88
128.37
128.21
128.15
126.83
126.75
126.60
112.24
97.81
52.27
33.50
O
O
CH3
S
200
190
180
170
160
150
140
130
120
110
100
90
80
70
60
50
40
30
20
f1 (ppm)

(14)

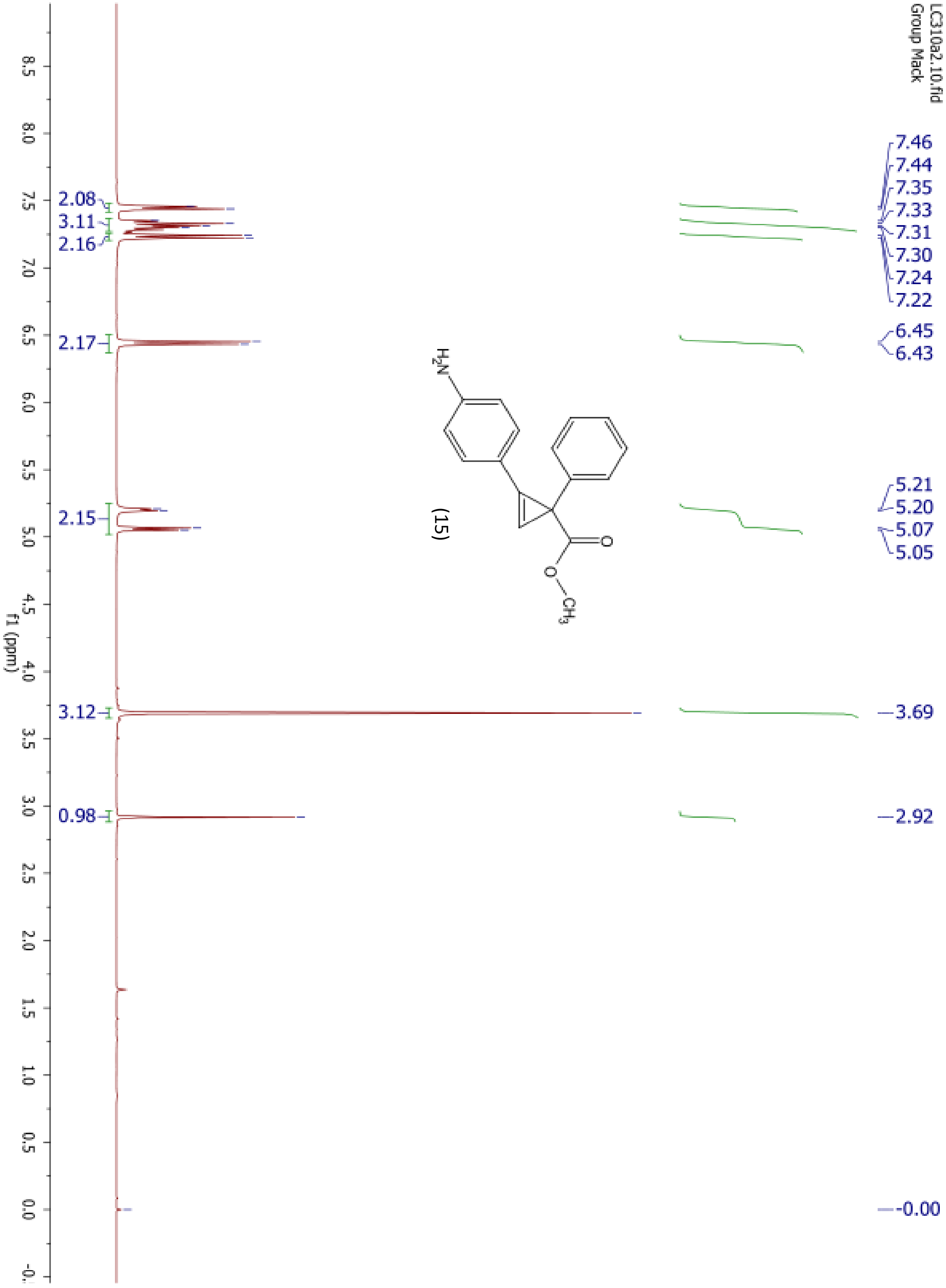
LC310a2.10.fid
Group Mack
7.46
7.44
7.35
7.33
7.31
7.30
7.24
7.22
6.45
6.43
5.21
5.20
5.07
5.05
3.69
2.92
-0.00
2.08
3.11
2.16
2.17
2.15
3.12
0.98
H2N
O
O
CH3
(15)
f1 (ppm)
8.5
8.0
7.5
7.0
6.5
6.0
5.5
5.0
4.5
4.0
3.5
3.0
2.5
2.0
1.5
1.0
0.5
0.0

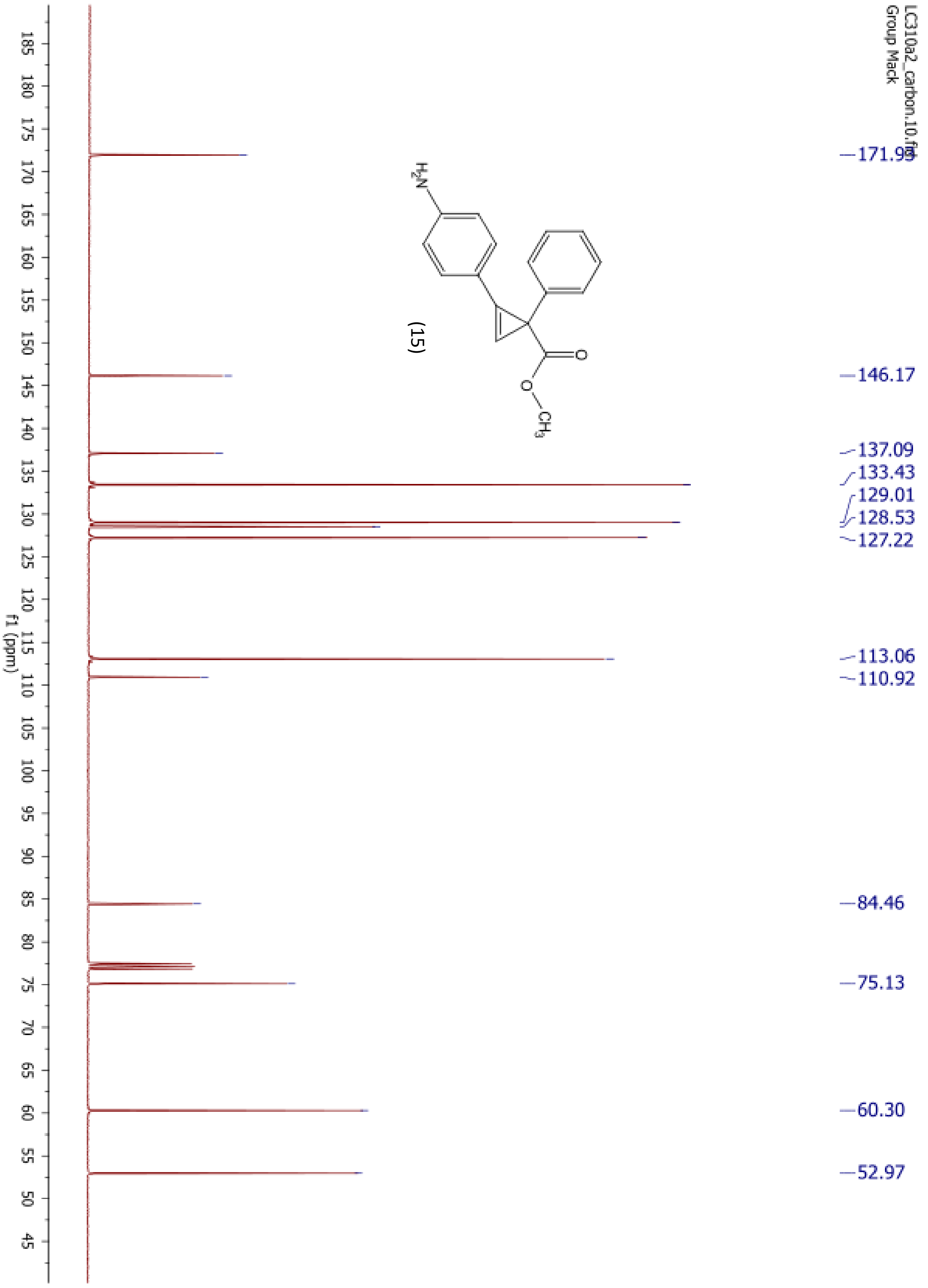
LC310a2_carbon.10.fid
Group Mack
(15)
H2N
O
O
CH3
171.93
146.17
137.09
133.43
129.01
128.53
127.22
113.06
110.92
84.46
75.13
60.30
52.97
f1 (ppm)

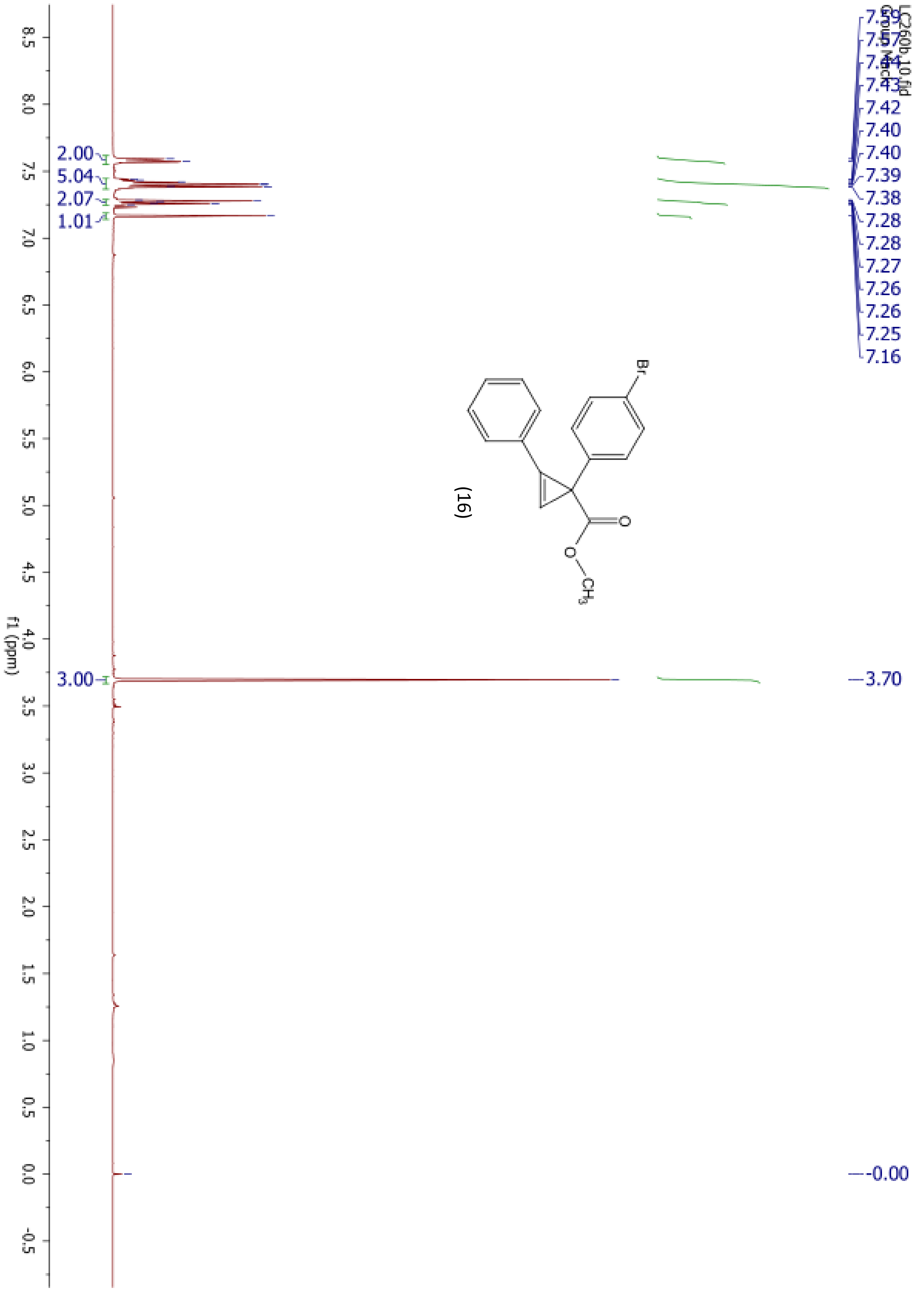
(16)
Br
O
CH3
f1 (ppm)
2.00
5.04
2.07
1.01
3.00
7.59
7.57
7.44
7.43
7.42
7.40
7.40
7.39
7.38
7.28
7.28
7.27
7.26
7.26
7.25
7.16
3.70
-0.00

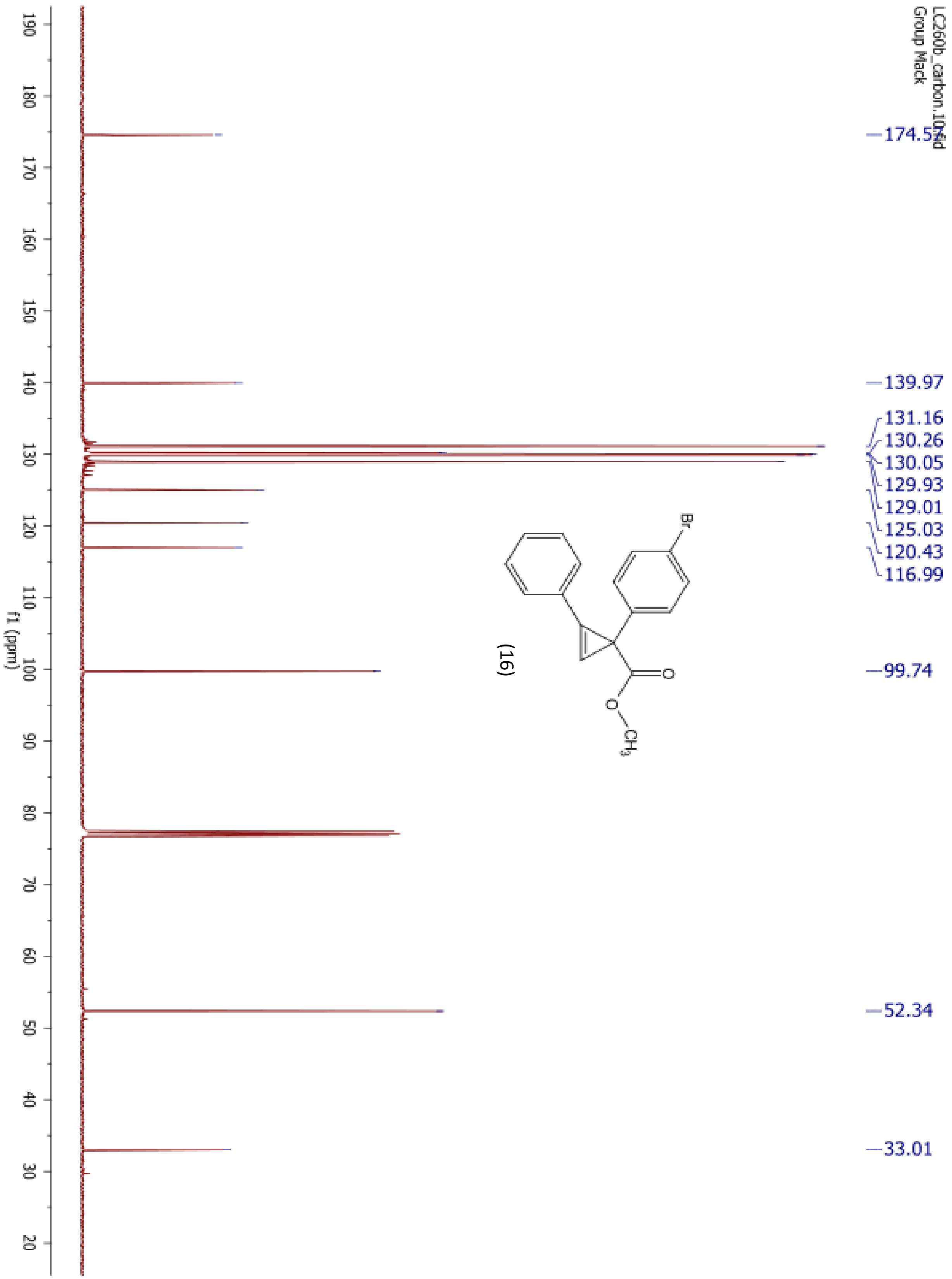
LC260b_carbon.10.fid
Group Mack
174.54
139.97
131.16
130.26
130.05
129.93
129.01
125.03
120.43
116.99
99.74
52.34
33.01
Br
O
O
CH_3
(16)
190
180
170
160
150
140
130
120
110
100
90
80
70
60
50
40
30
20
f1 (ppm)

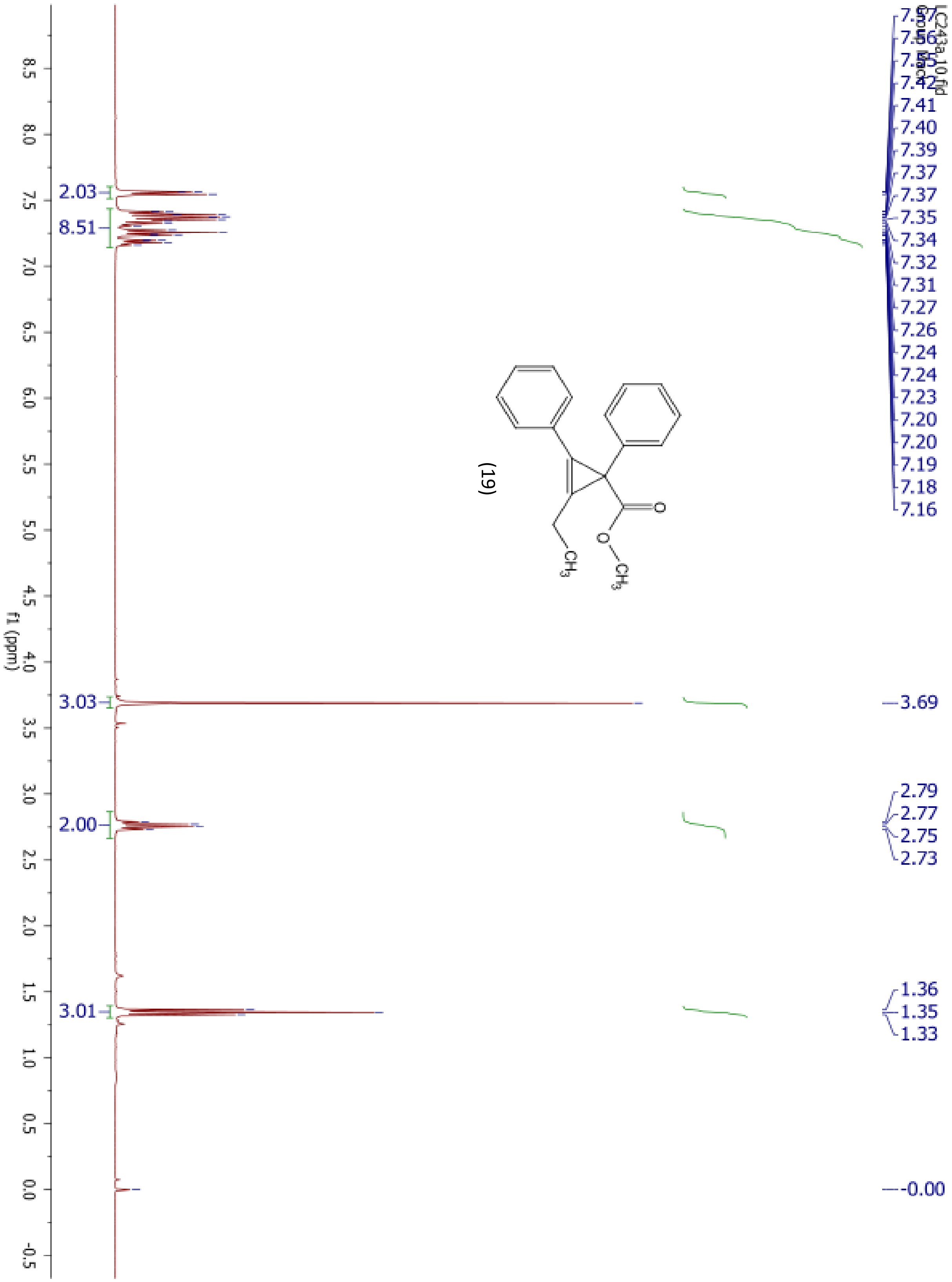

f1 (ppm)
2.03
8.51
3.03
2.00
3.01
7.41
7.40
7.39
7.37
7.37
7.35
7.34
7.32
7.31
7.27
7.26
7.24
7.24
7.23
7.20
7.20
7.19
7.18
7.16
3.69
2.79
2.77
2.75
2.73
1.36
1.35
1.33
-0.00
(19)

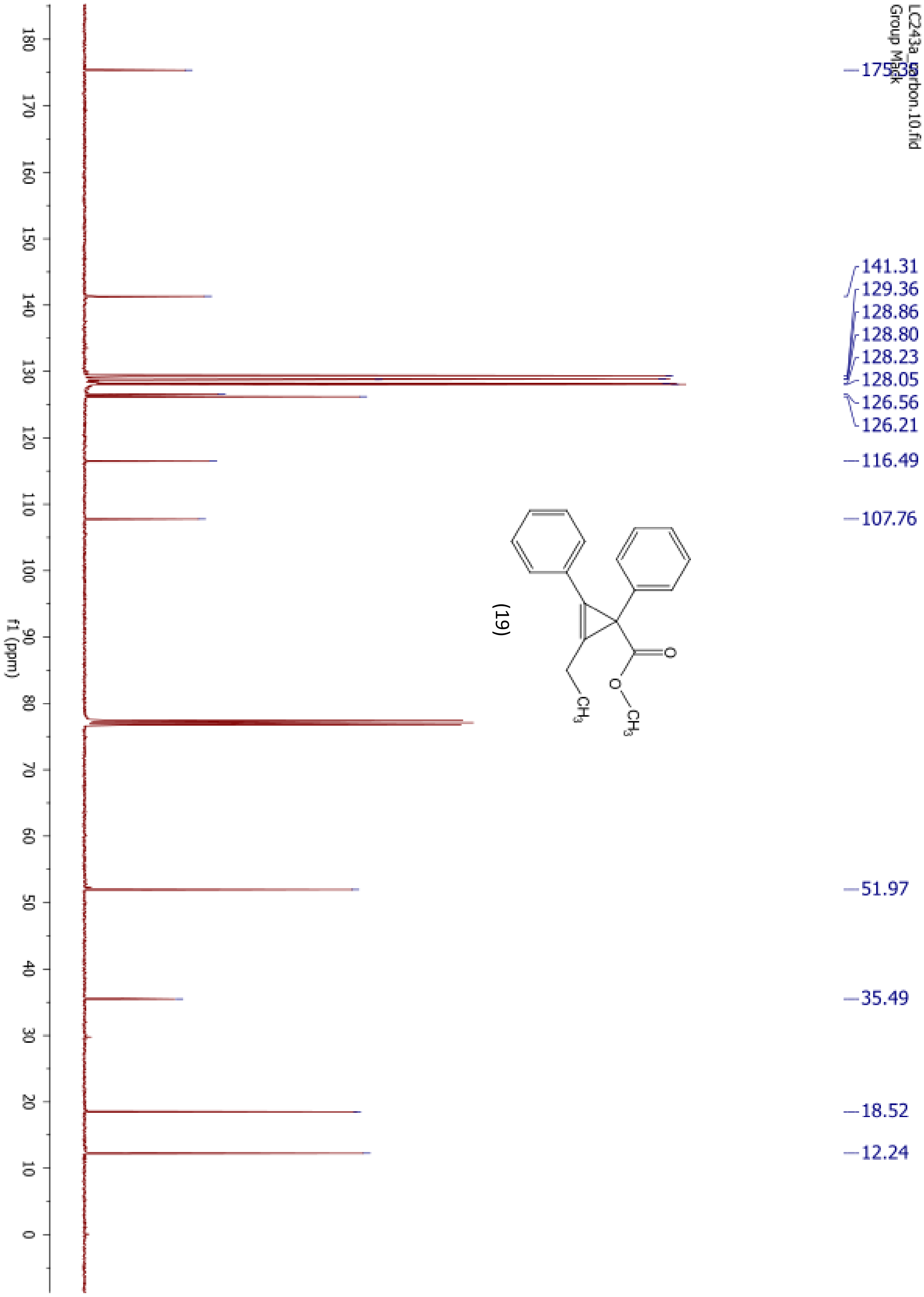
LC243a_carbon.10.fid
Group Mask
175.38
141.31
129.36
128.86
128.80
128.23
128.05
126.56
126.21
116.49
107.76
51.97
35.49
18.52
12.24
(19)
O
O
CH3
CH3
f1 (ppm)

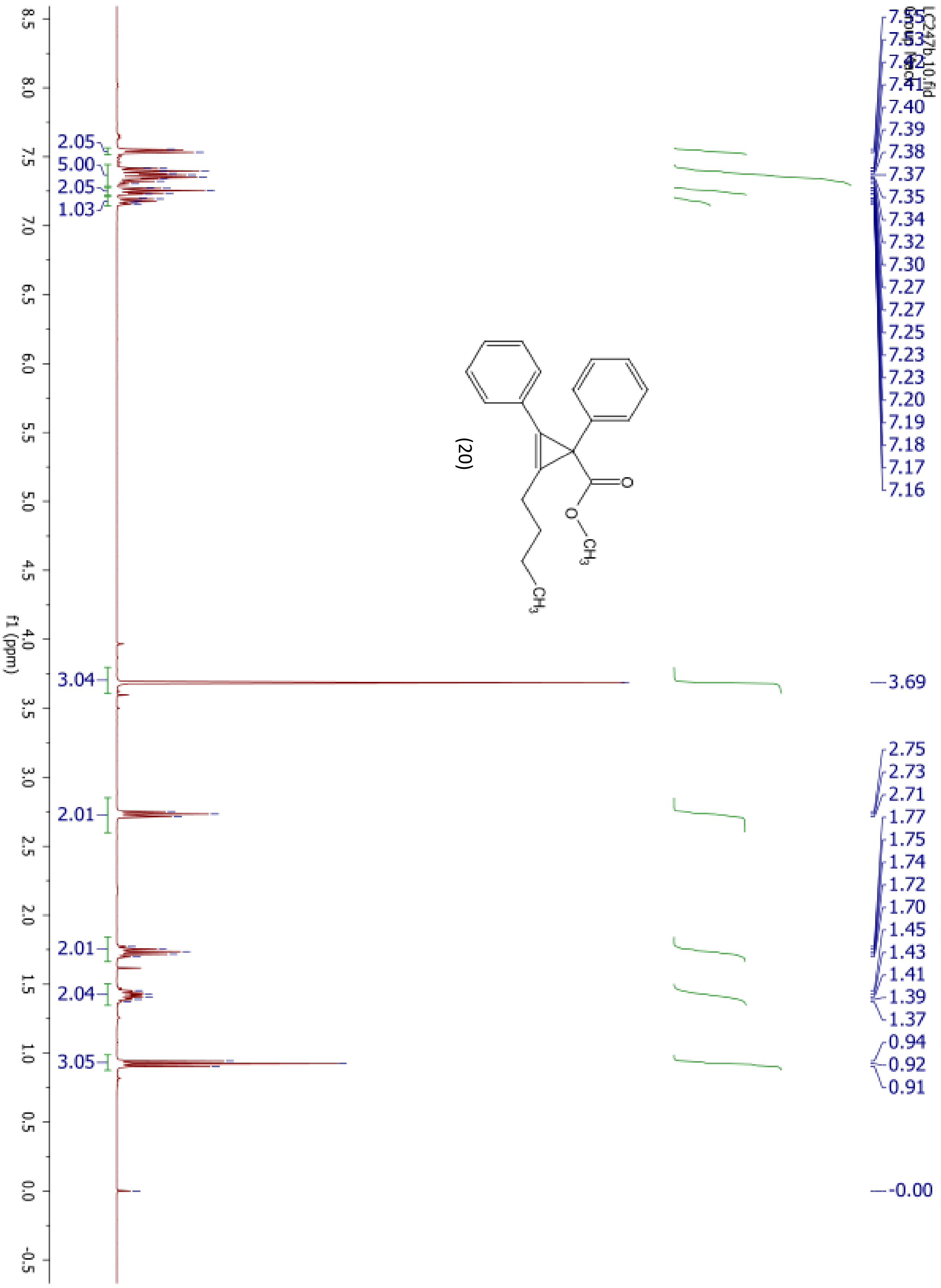

LC247b.10.fid
(20)
f1 (ppm)
2.05
5.00
2.05
1.03
3.04
2.01
2.01
2.04
3.05
3.69
-0.00

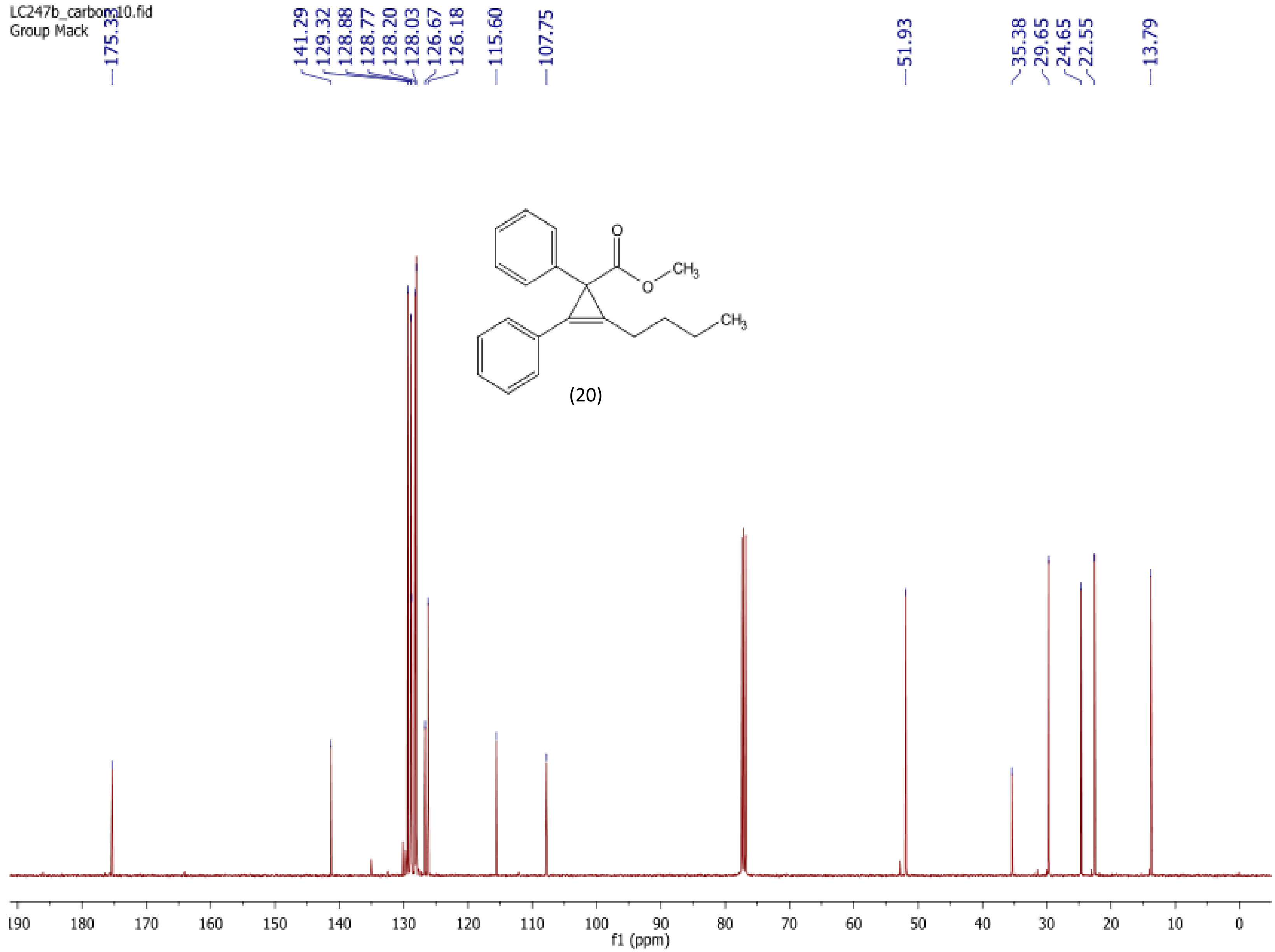
LC247b_carbon10.fid
Group Mack
175.33
141.29
129.32
128.88
128.77
128.20
128.03
126.67
126.18
115.60
107.75
51.93
35.38
29.65
24.65
22.55
13.79
O
O
CH3
CH3
f1 (ppm)
190 180 170 160 150 140 130 120 110 100 90 80 70 60 50 40 30 20 10 0

(20)

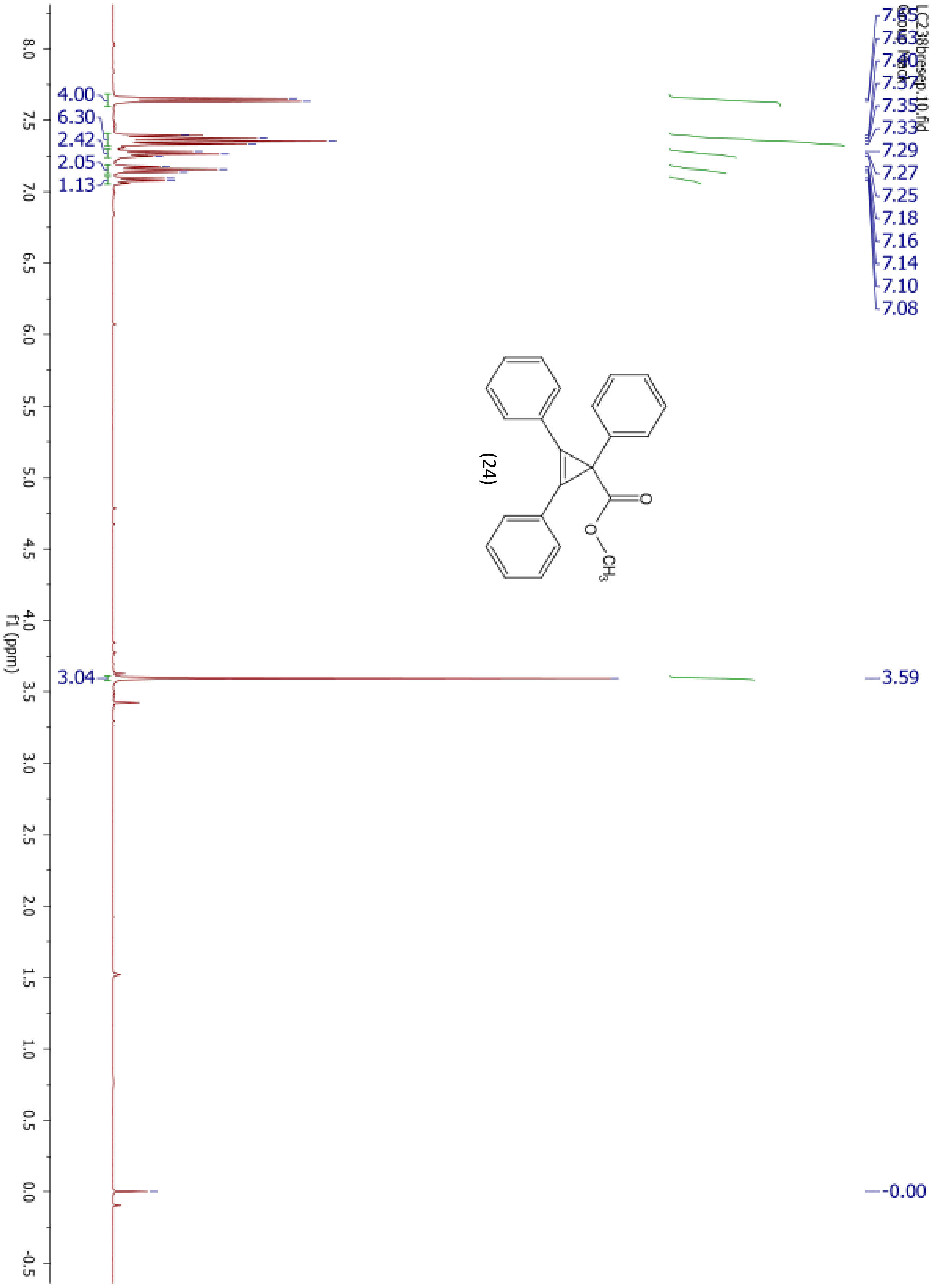
LC238bresep.10.fid
7.65
7.63
7.40
7.37
7.35
7.33
7.29
7.27
7.25
7.18
7.16
7.14
7.10
7.08
3.59
-0.00
4.00
6.30
2.42
2.05
1.13
3.04
f1 (ppm)
O
O
CH3

(24)

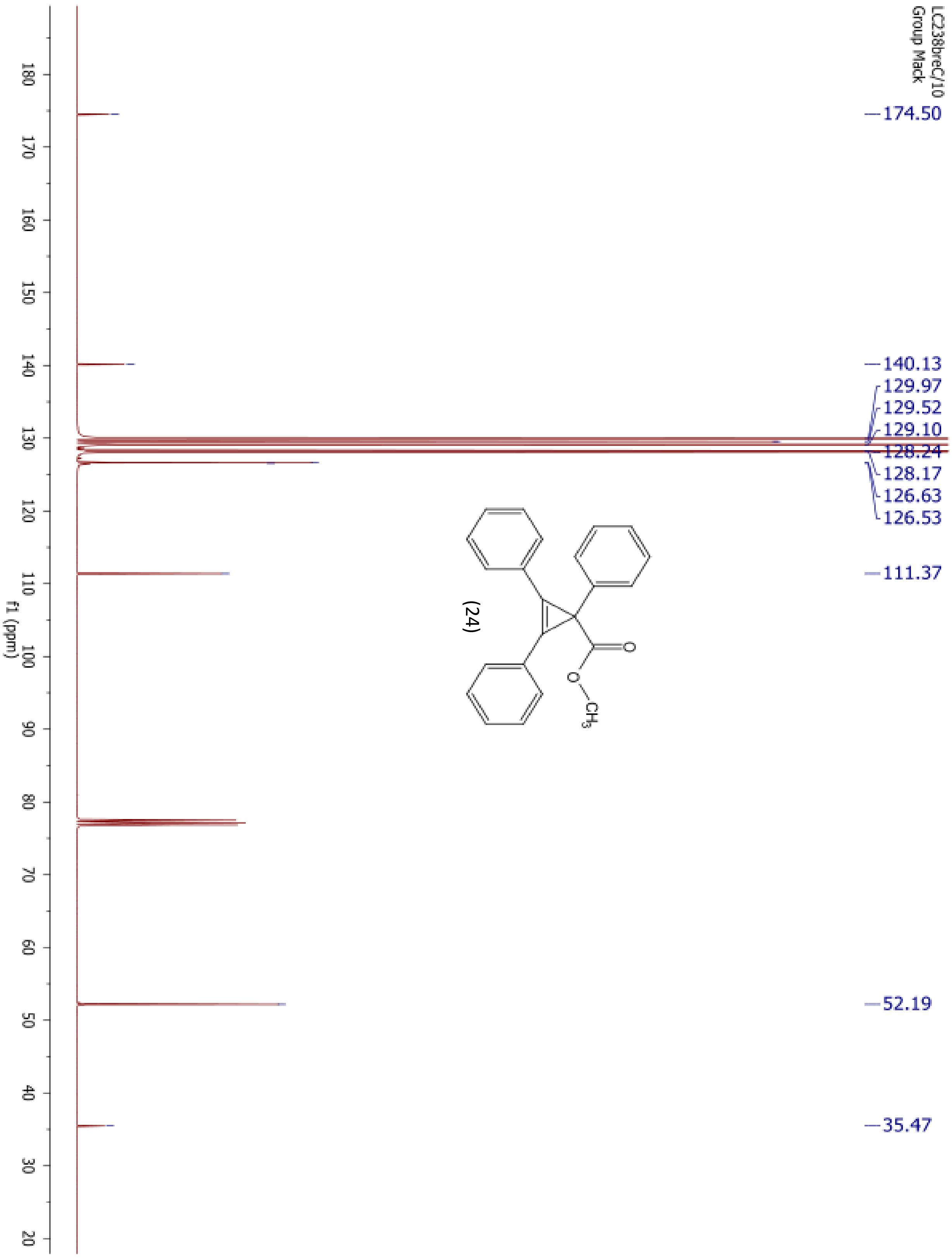

LC238breC/10
Group Mack
174.50
140.13
129.97
129.52
129.10
128.24
128.17
126.63
126.53
111.37
52.19
35.47
(24)
O
O
CH3
f1 (ppm)
180
170
160
150
140
130
120
110
100
90
80
70
60
50
40
30
20

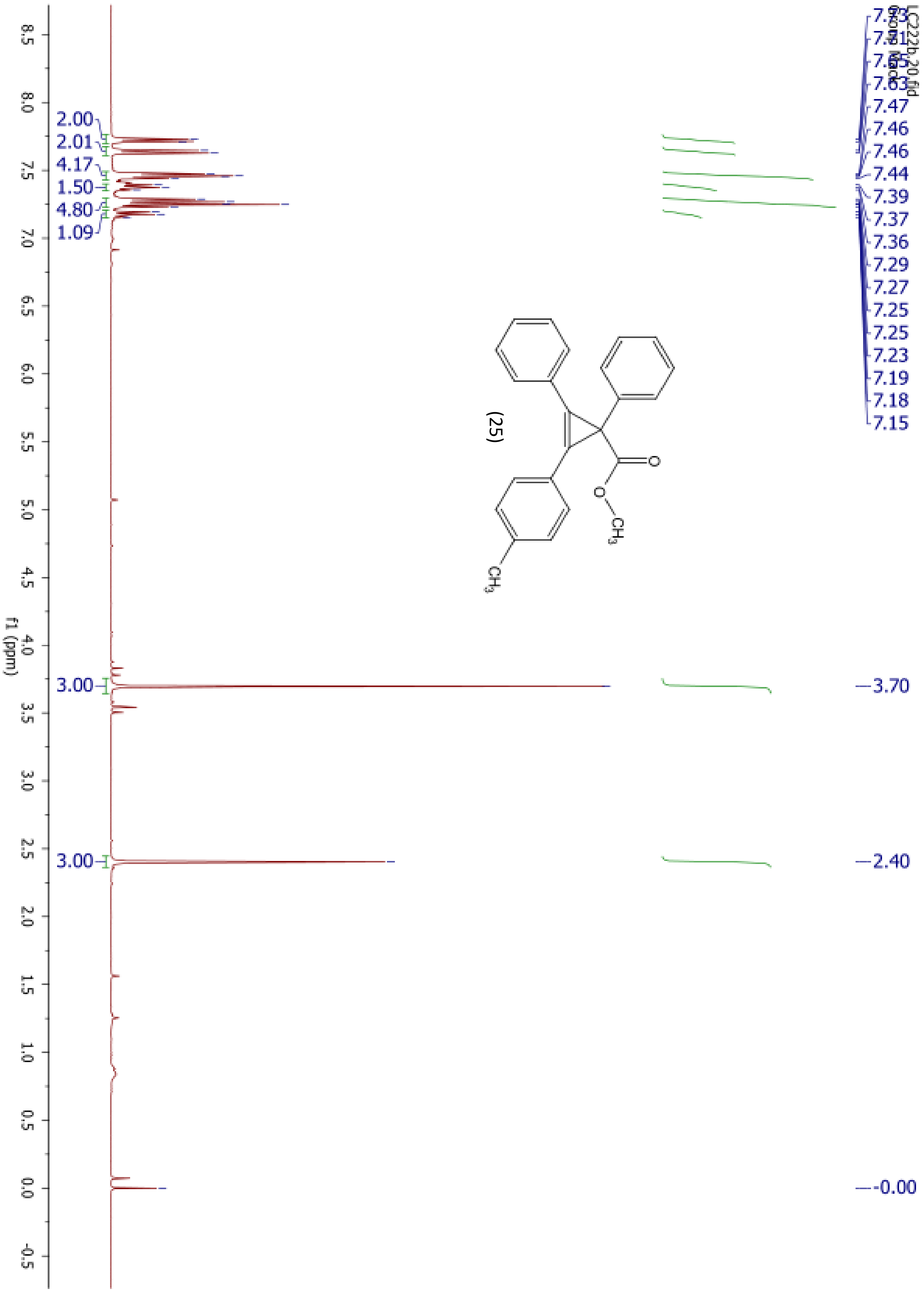
(25)
f1 (ppm)
2.00
2.01
4.17
1.50
4.80
1.09
3.00
3.00
7.73
7.71
7.65
7.63
7.47
7.46
7.46
7.44
7.39
7.37
7.36
7.29
7.27
7.25
7.25
7.23
7.19
7.18
7.15
3.70
2.40
-0.00

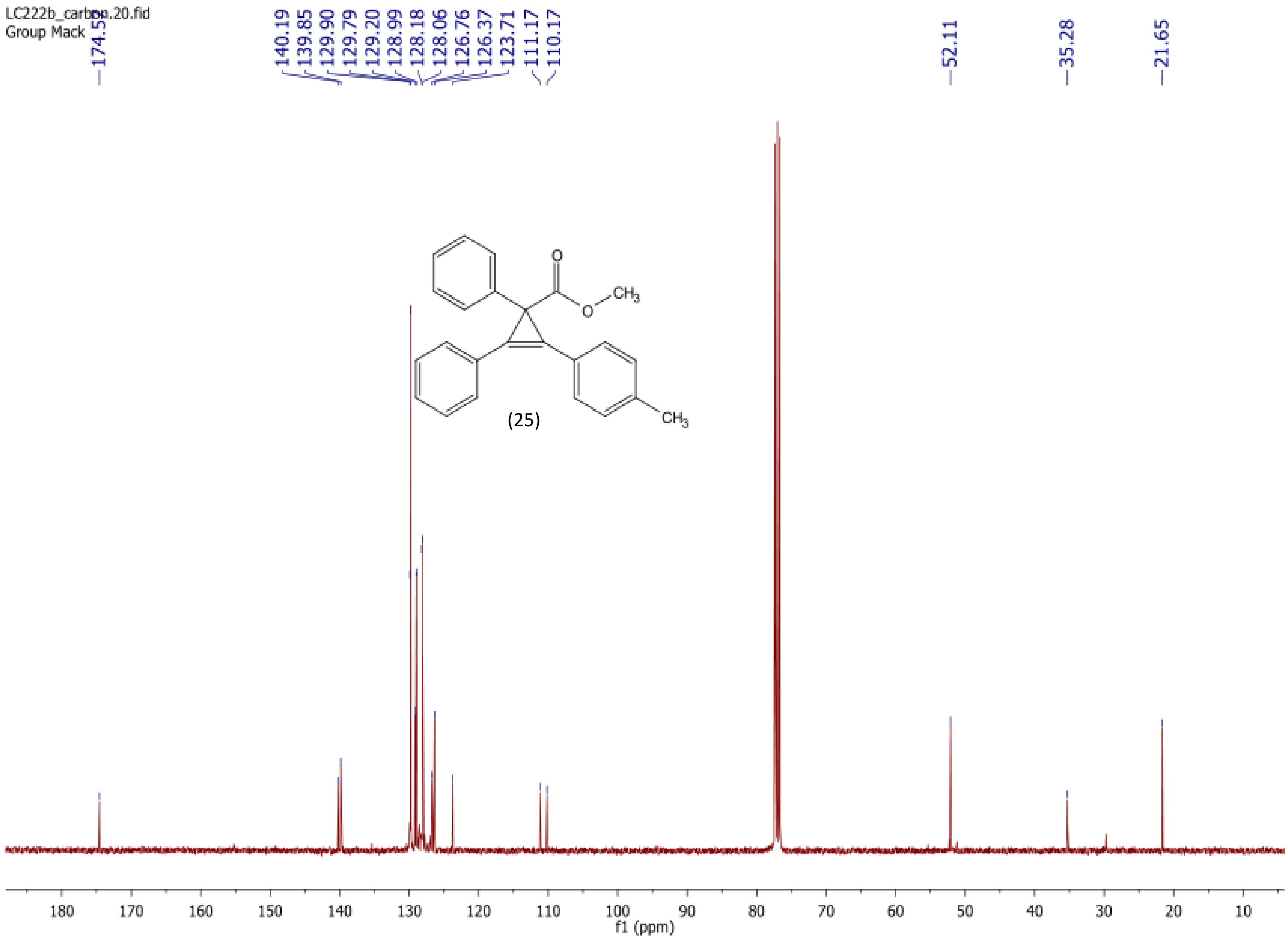
LC222b_carbon.20.fid
Group Mack
174.50
140.19
139.85
129.90
129.79
129.20
128.99
128.18
128.06
126.76
126.37
123.71
111.17
110.17
52.11
35.28
21.65
O
O
CH3
CH3
(25)
180
170
160
150
140
130
120
110
100
90
80
70
60
50
40
30
20
10
f1 (ppm)

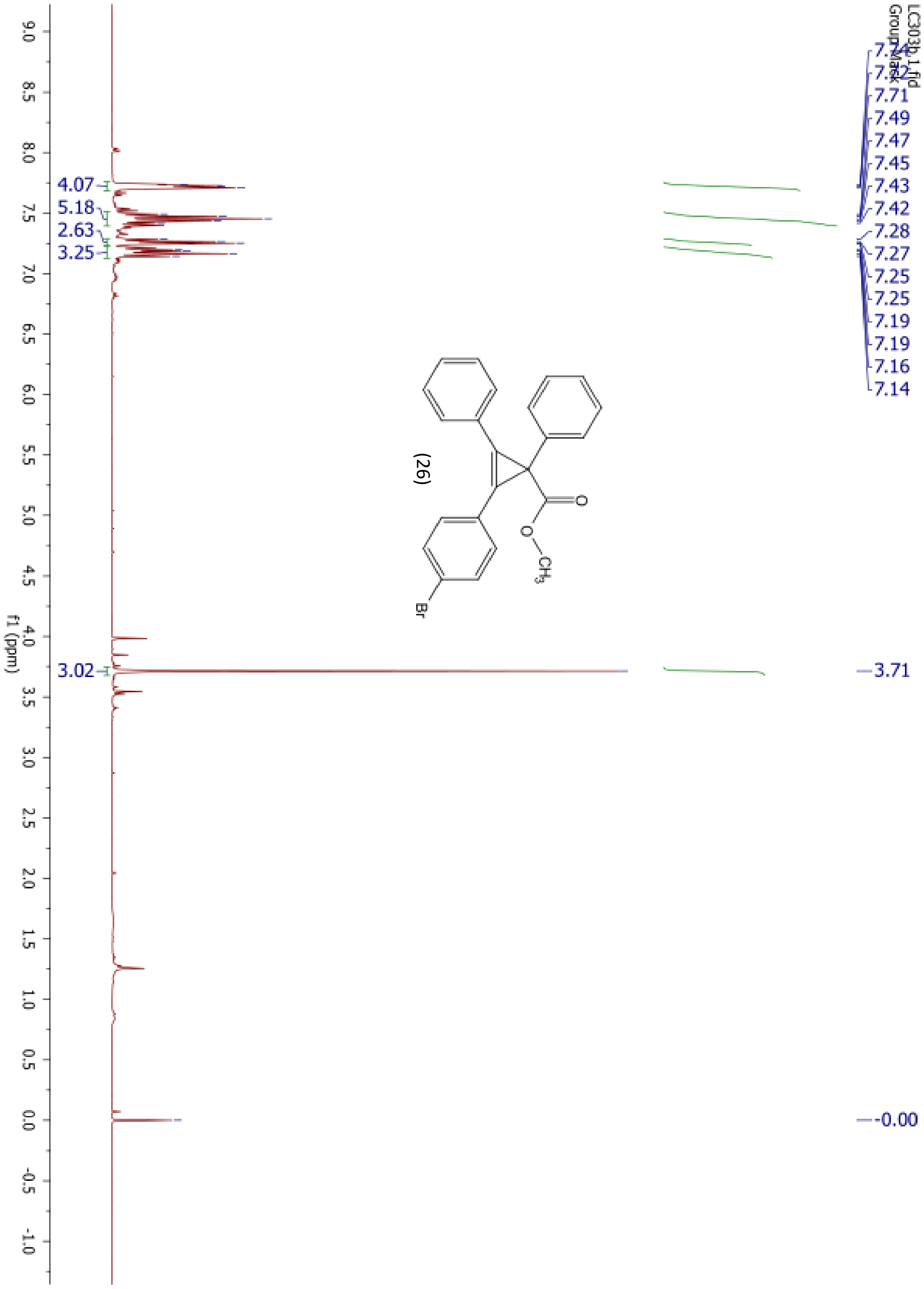
LC303b.1.fid
Group Mark
7.71
7.49
7.47
7.45
7.43
7.42
7.28
7.27
7.25
7.25
7.19
7.19
7.16
7.14
3.71
-0.00
4.07
5.18
2.63
3.25
3.02
(26)
Br
O
CH3
f1 (ppm)

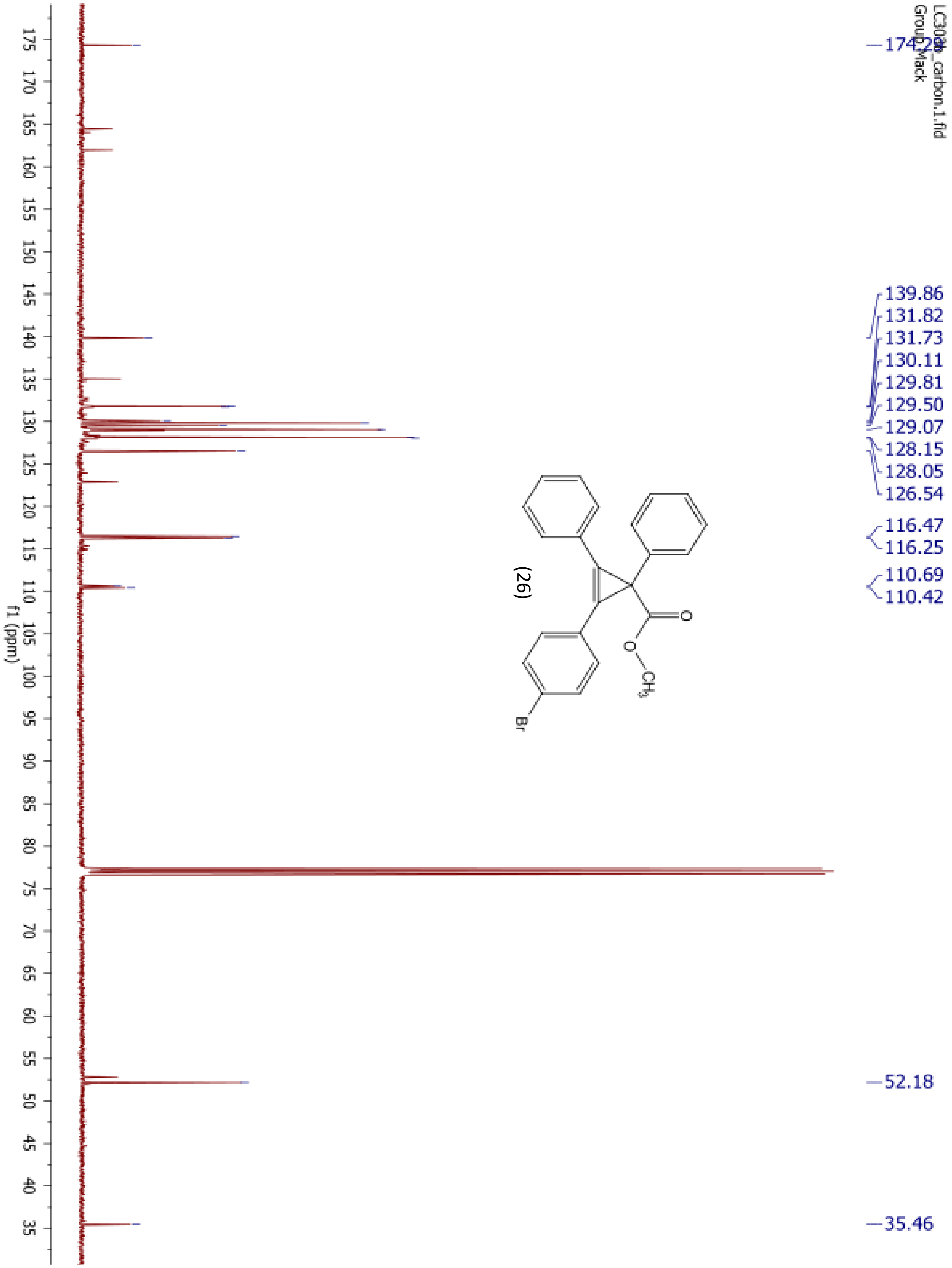

Group Mack
174.28
139.86
131.82
131.73
130.11
129.81
129.50
129.07
128.15
128.05
126.54
116.47
116.25
110.69
110.42
52.18
35.46
(26)
Br
O
CH3
f1 (ppm)

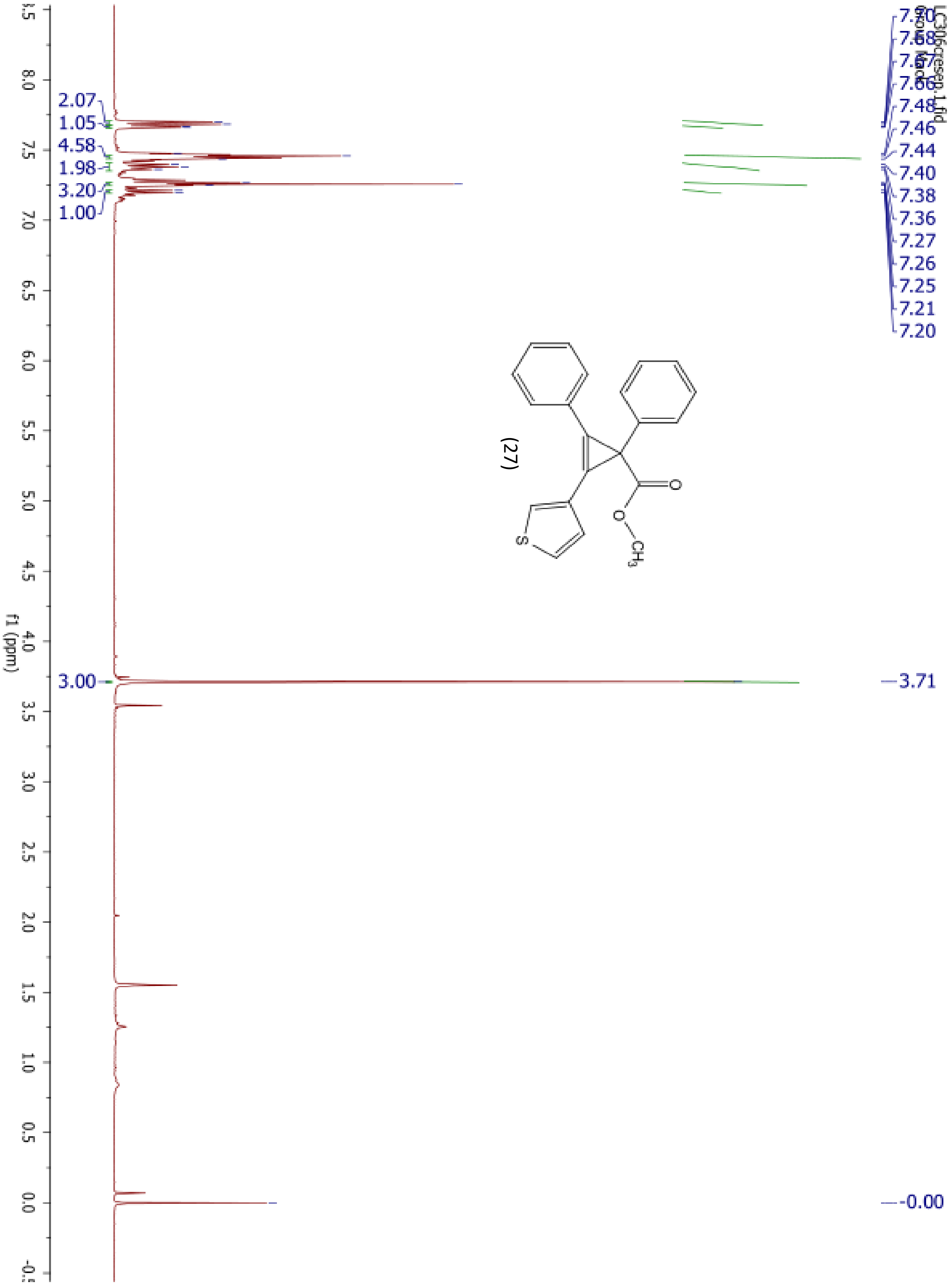

2.07
1.05
4.58
1.98
3.20
1.00
3.00
7.70
7.68
7.67
7.66
7.48
7.46
7.44
7.40
7.38
7.36
7.27
7.26
7.25
7.21
7.20
3.71
-0.00
(27)
f1 (ppm)

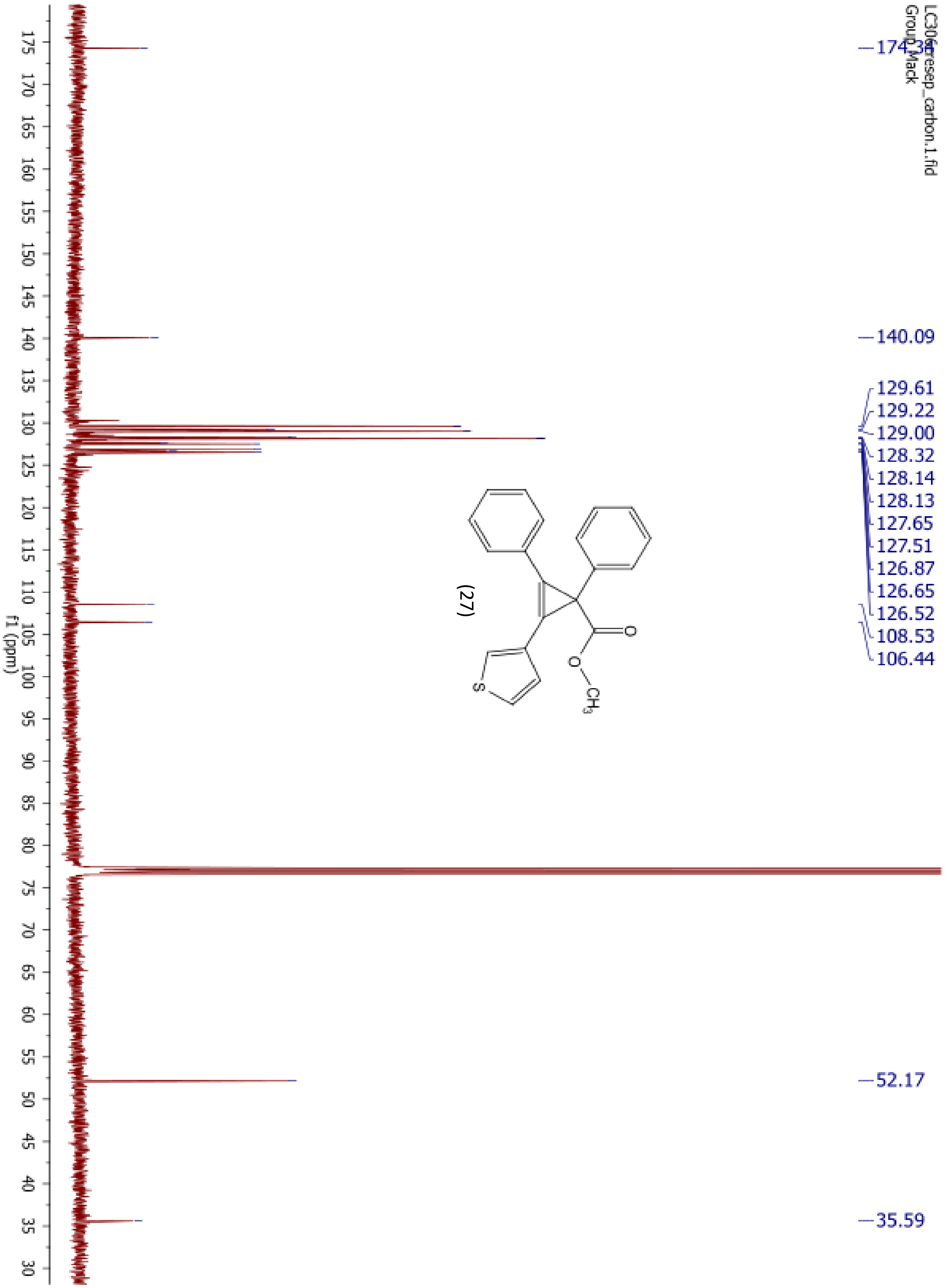

174.34
140.09
129.61
129.22
129.00
128.32
128.14
128.13
127.65
127.51
126.87
126.65
126.52
108.53
106.44
52.17
35.59
(27)
f1 (ppm)

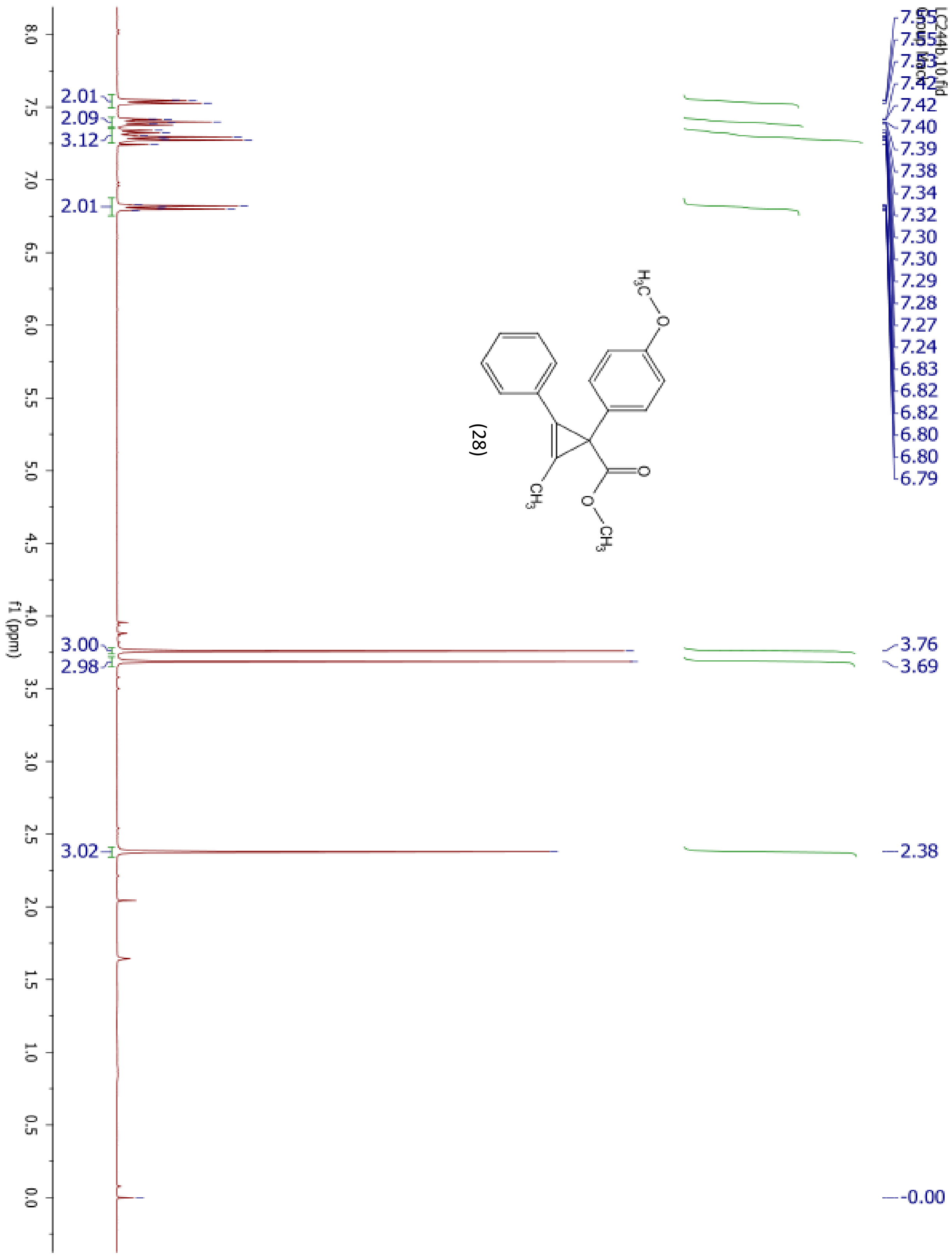
f1 (ppm)
2.01
2.09
3.12
2.01
3.00
2.98
3.02
7.55
7.55
7.53
7.42
7.42
7.40
7.39
7.38
7.34
7.32
7.30
7.30
7.29
7.28
7.27
7.24
6.83
6.82
6.82
6.80
6.80
6.79
3.76
3.69
2.38
-0.00
(28)

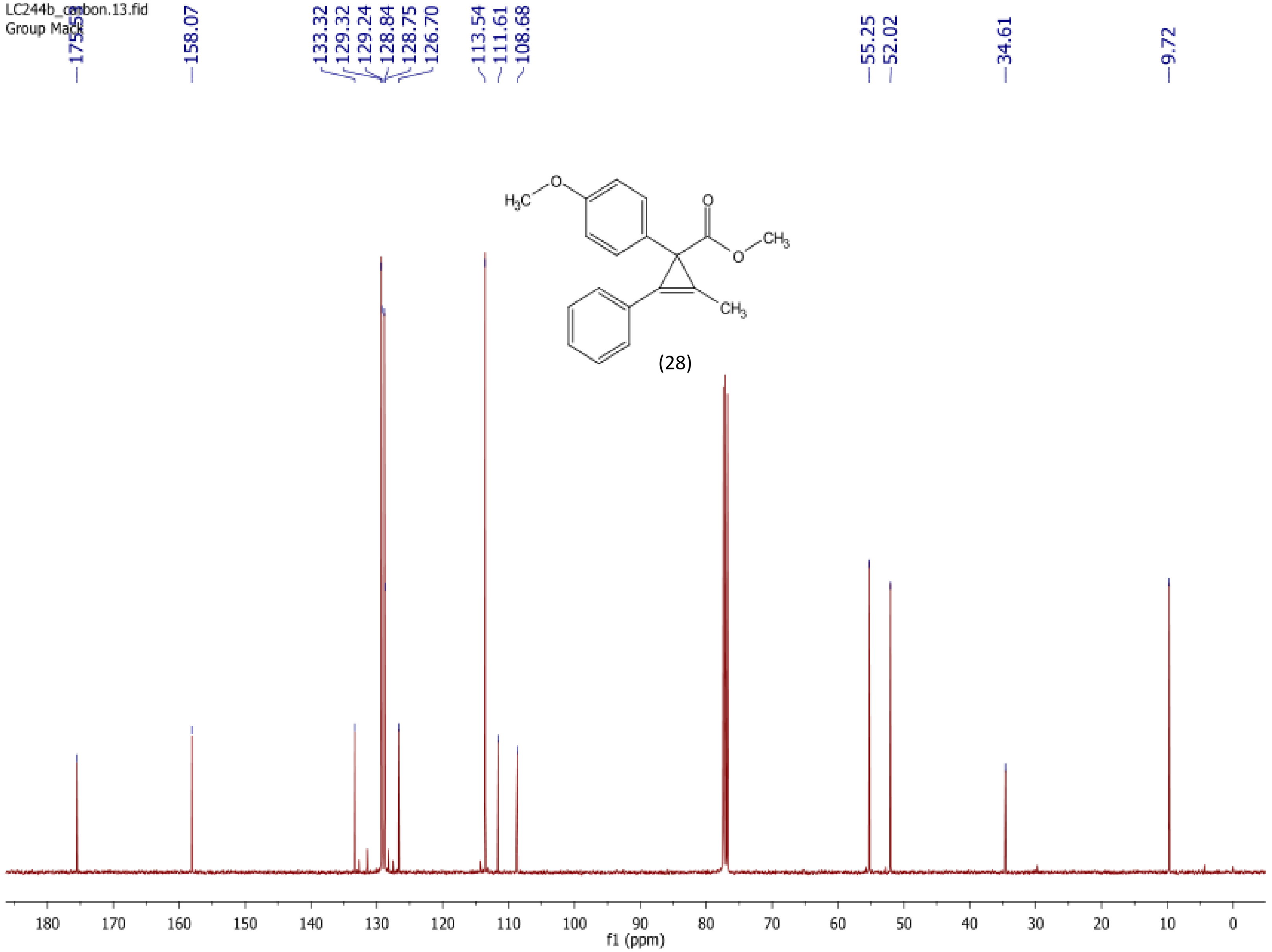
LC244b_carbon.13.fid
Group Mack
158.07
133.32
129.32
129.24
128.84
128.75
126.70
113.54
111.61
108.68
55.25
52.02
34.61
9.72
H_3C
O
O
O
CH_3
CH_3
(28)
180
170
160
150
140
130
120
110
100
90
80
70
60
50
40
30
20
10
0
f1 (ppm)

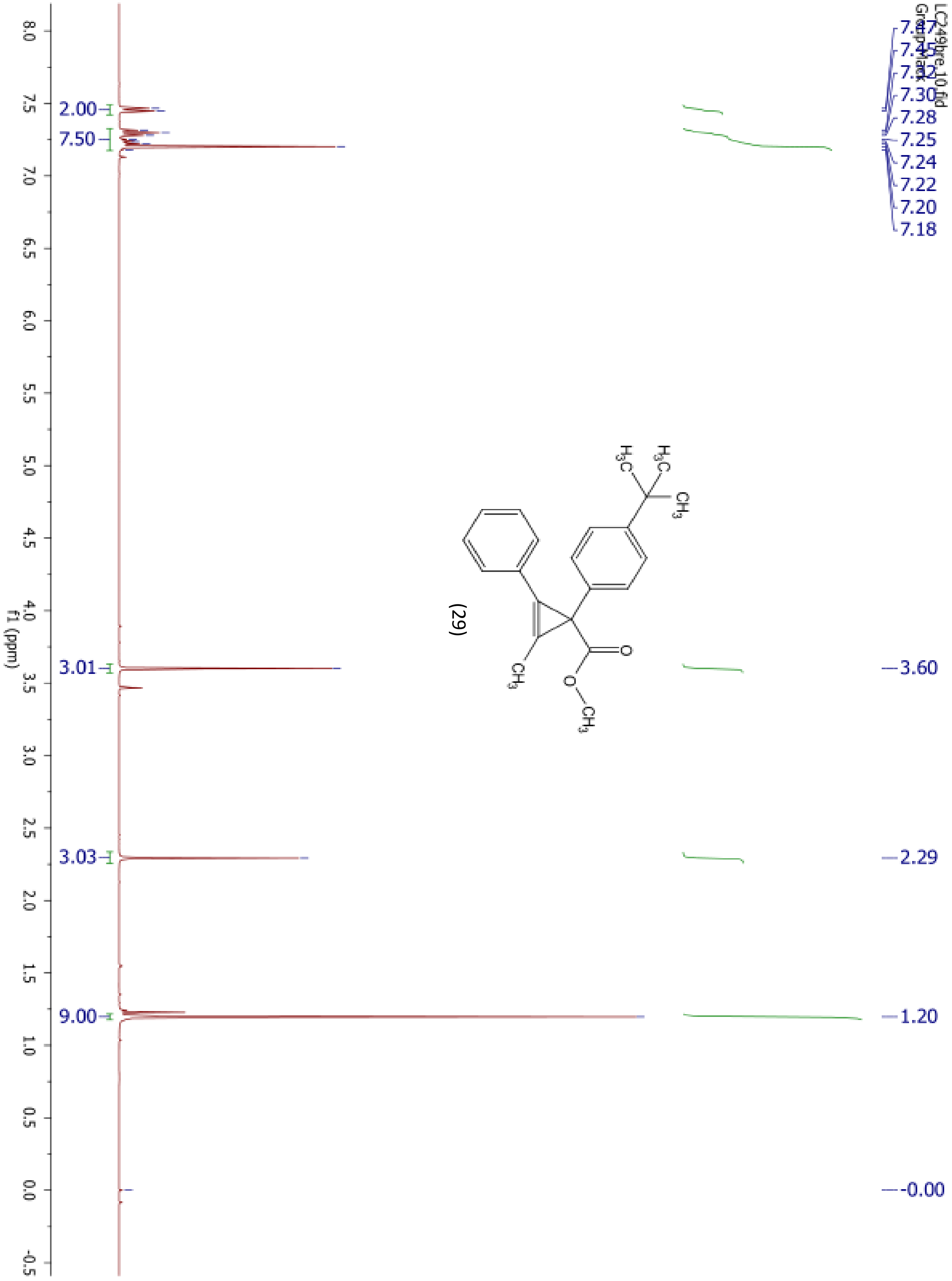
LC249hre_10.fid
Group Mack
7.30
7.28
7.25
7.24
7.22
7.20
7.18
3.60
2.29
1.20
-0.00
2.00
7.50
3.01
3.03
9.00
H3C
H3C
CH3
O
O
CH3
CH3
(29)
8.0
7.5
7.0
6.5
6.0
5.5
5.0
4.5
4.0
3.5
3.0
2.5
2.0
1.5
1.0
0.5
0.0
-0.5
f1 (ppm)

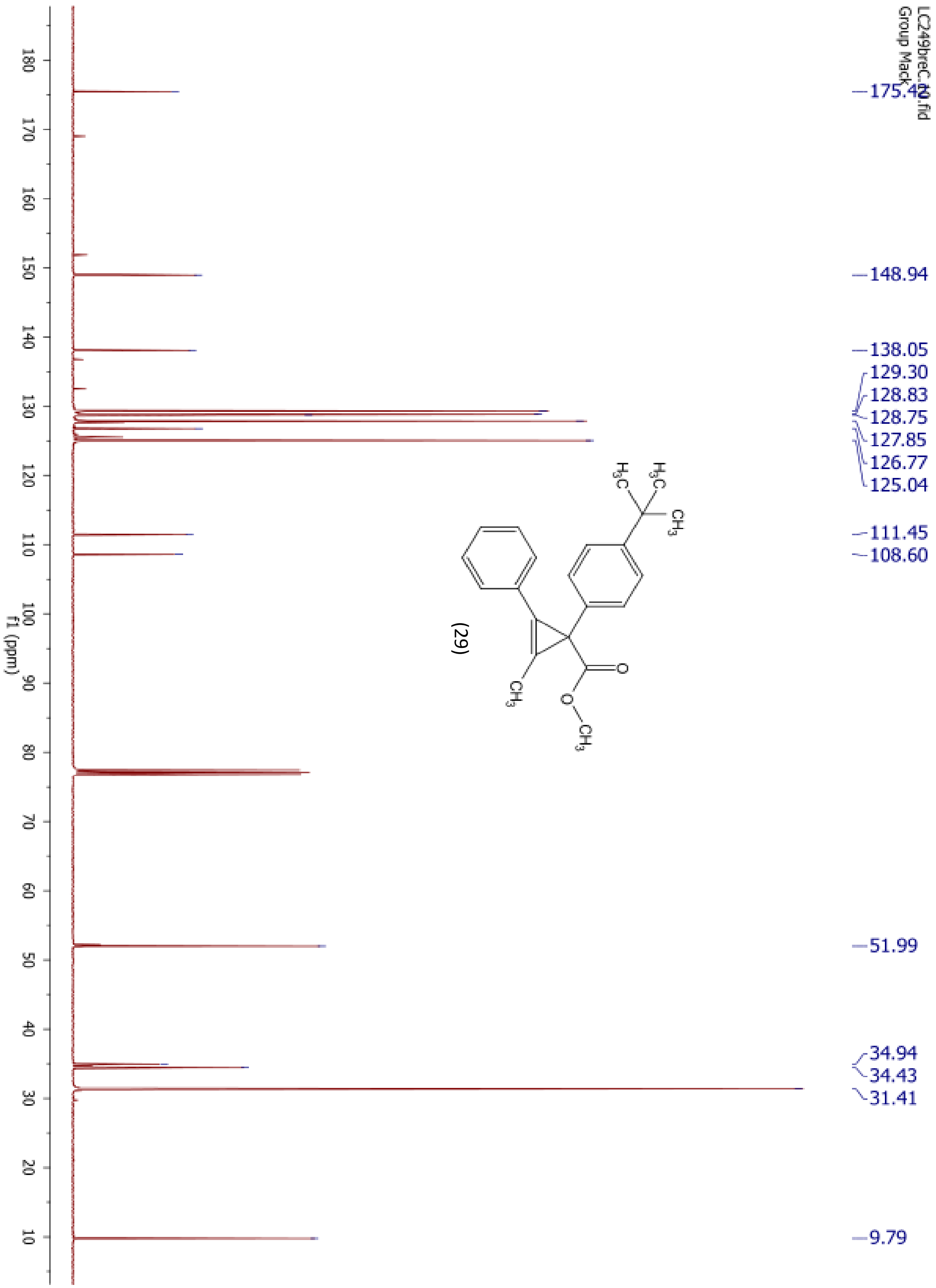

LC249breC.10.fid
Group Mack
175.42
148.94
138.05
129.30
128.83
128.75
127.85
126.77
125.04
111.45
108.60
51.99
34.94
34.43
31.41
9.79
(29)
f1 (ppm)

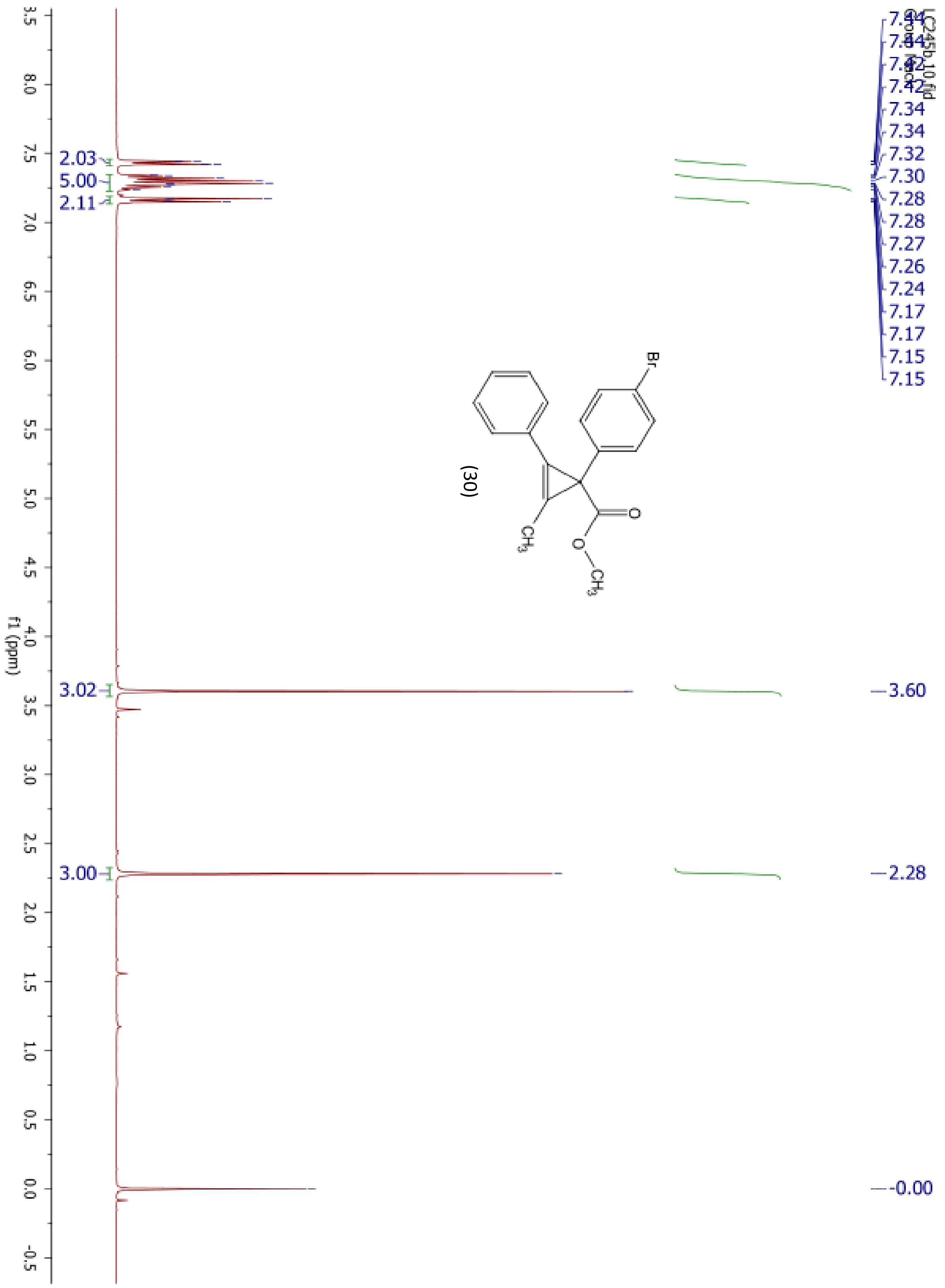

LC245b.10.fid
Proton Nick
7.44
7.44
7.42
7.42
7.34
7.34
7.32
7.30
7.28
7.28
7.27
7.26
7.24
7.17
7.17
7.15
7.15
3.60
2.28
-0.00
2.03
5.00
2.11
3.02
3.00
(30)
Br
O
CH3
f1 (ppm)

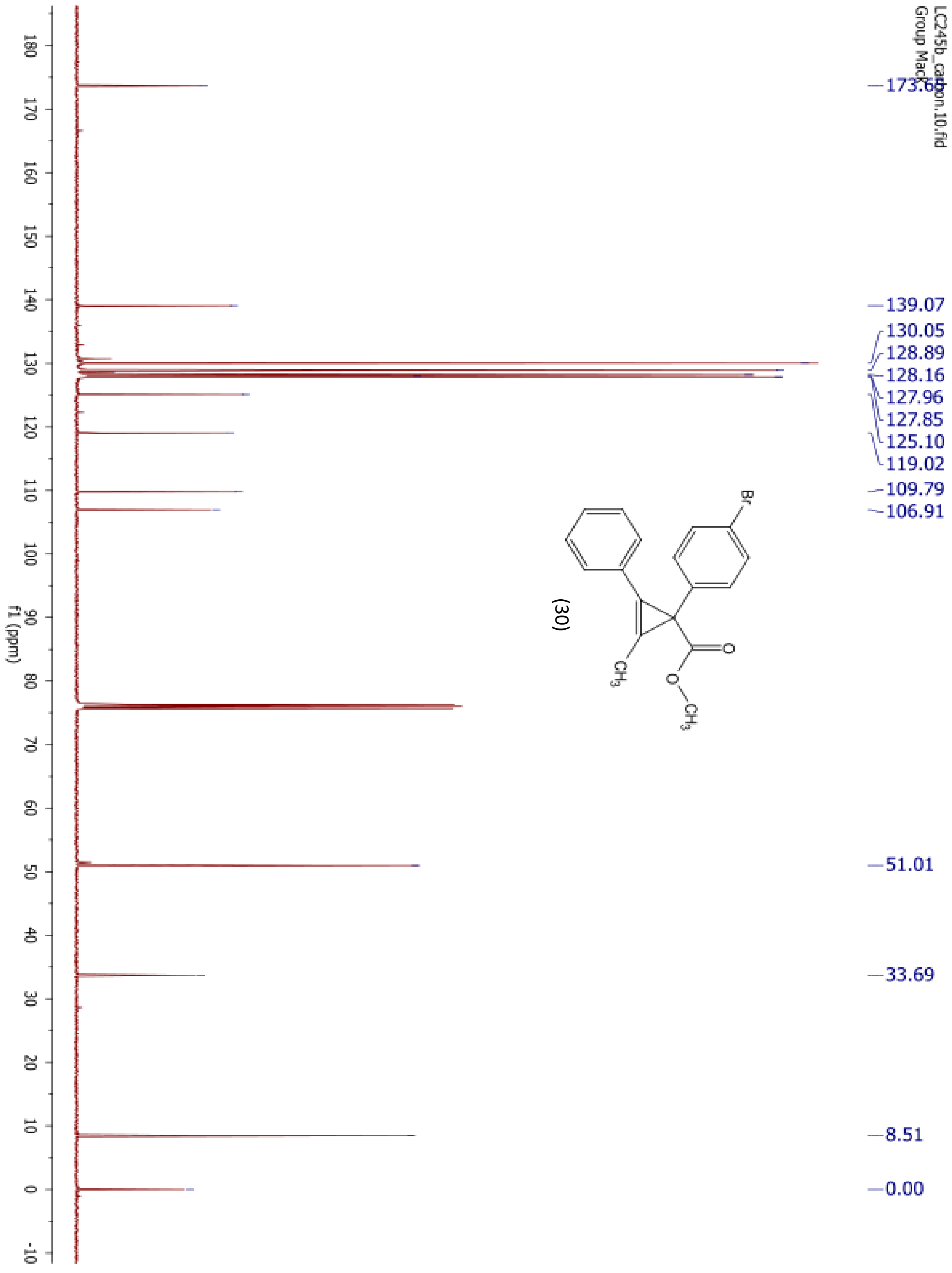
LC245b_carbon.10.fid
Group Mack
173.65
139.07
130.05
128.89
128.16
127.96
127.85
125.10
119.02
109.79
106.91
51.01
33.69
8.51
0.00
f1 (ppm)
Br
O
CH3
(30)

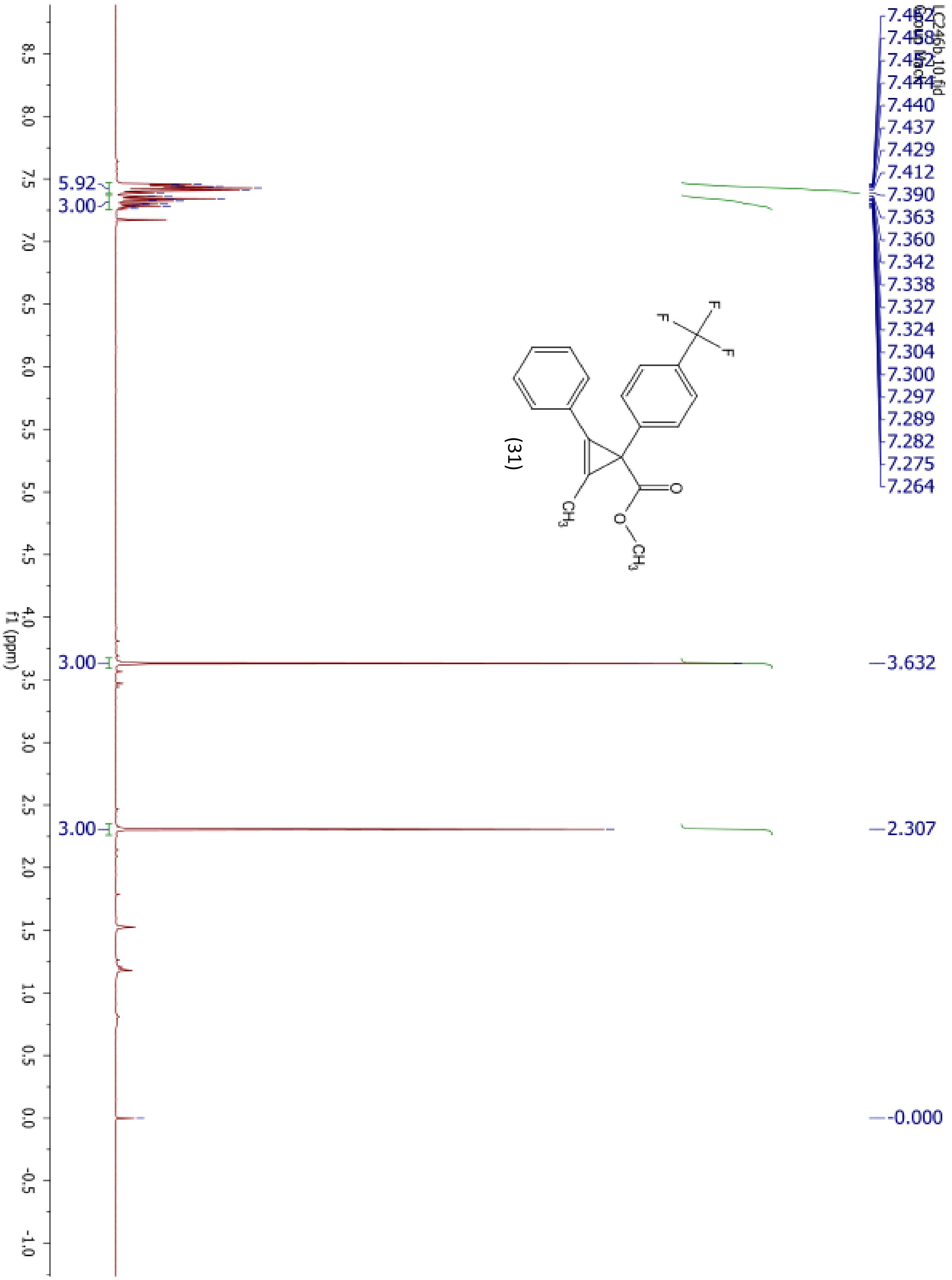
(31)
7.444
7.440
7.437
7.429
7.412
7.390
7.363
7.360
7.342
7.338
7.327
7.324
7.304
7.300
7.297
7.289
7.282
7.275
7.264
3.632
2.307
-0.000
5.92
3.00
3.00
3.00
f1 (ppm)

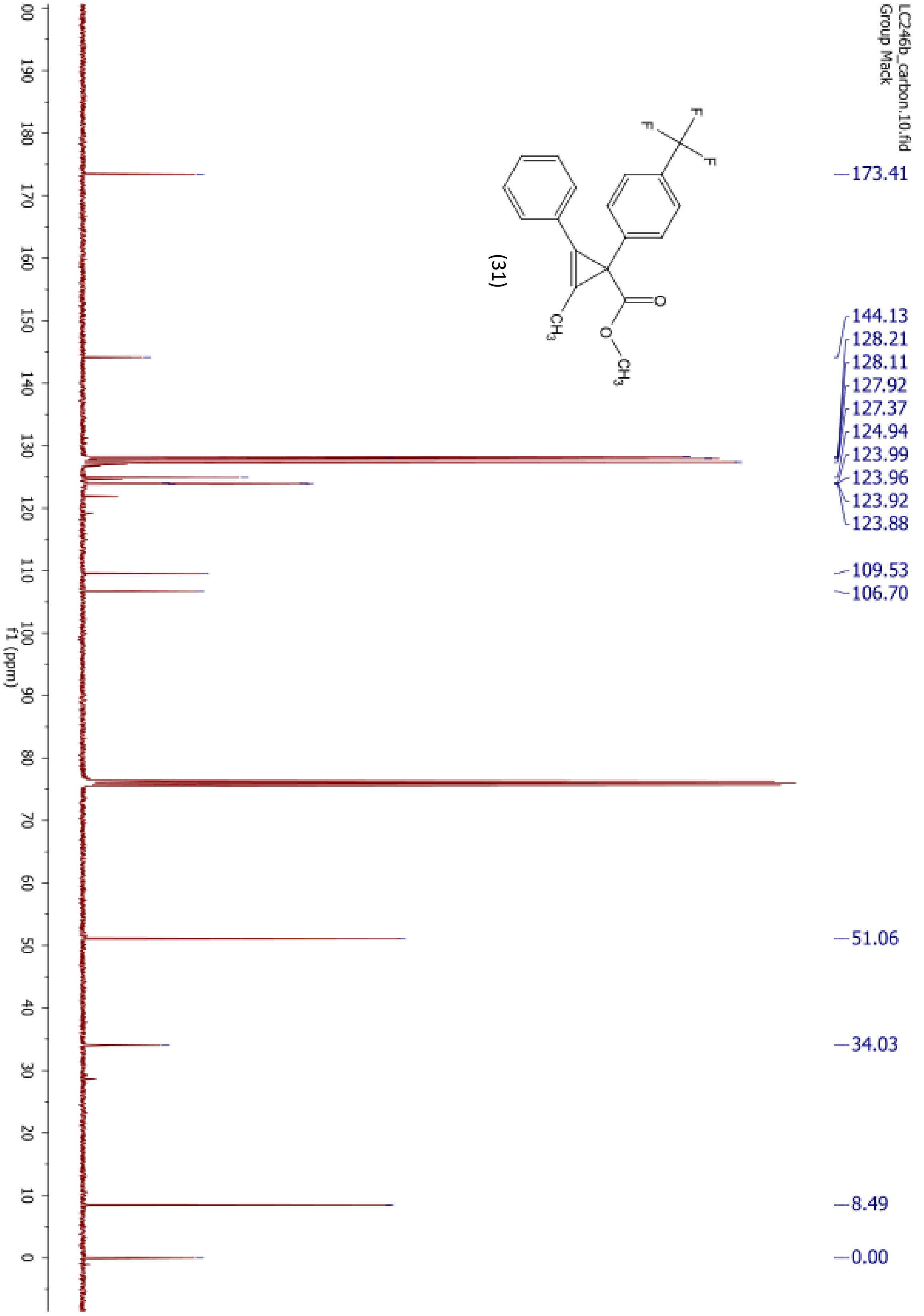

LC246b_carbon.10.fid
Group Mack
(31)
CH3
O
CH3
F
F
F
173.41
144.13
128.21
128.11
127.92
127.37
124.94
123.99
123.96
123.92
123.88
109.53
106.70
51.06
34.03
8.49
0.00
f1 (ppm)

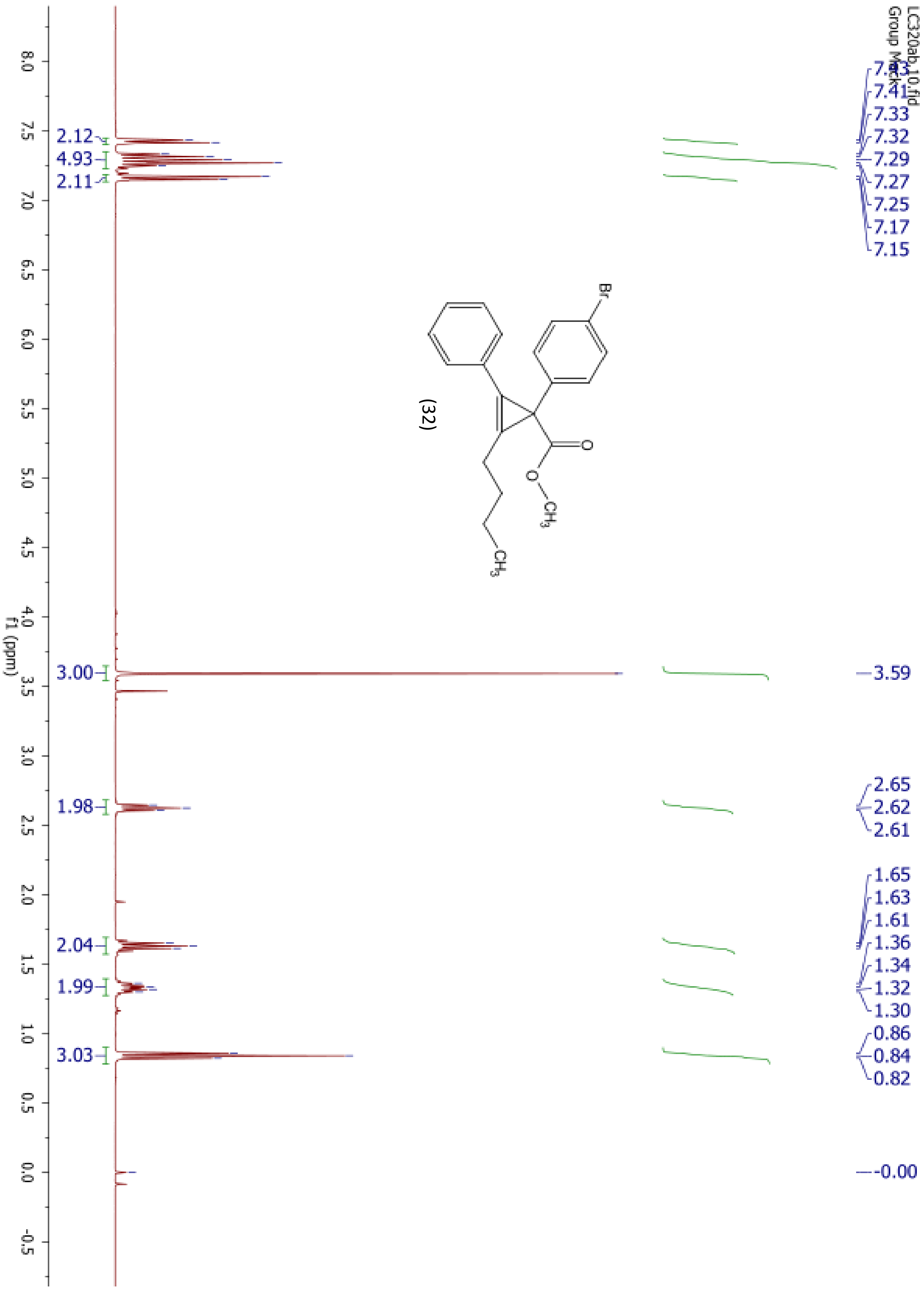
LC320ab_10.fid
7.43
7.41
7.33
7.32
7.29
7.27
7.25
7.17
7.15
3.59
2.65
2.62
2.61
1.65
1.63
1.61
1.36
1.34
1.32
1.30
0.86
0.84
0.82
-0.00
2.12
4.93
2.11
3.00
1.98
2.04
1.99
3.03
Br
O
O
CH3
CH3
(32)
8.0
7.5
7.0
6.5
6.0
5.5
5.0
4.5
4.0
3.5
3.0
2.5
2.0
1.5
1.0
0.5
0.0
-0.5
f1 (ppm)

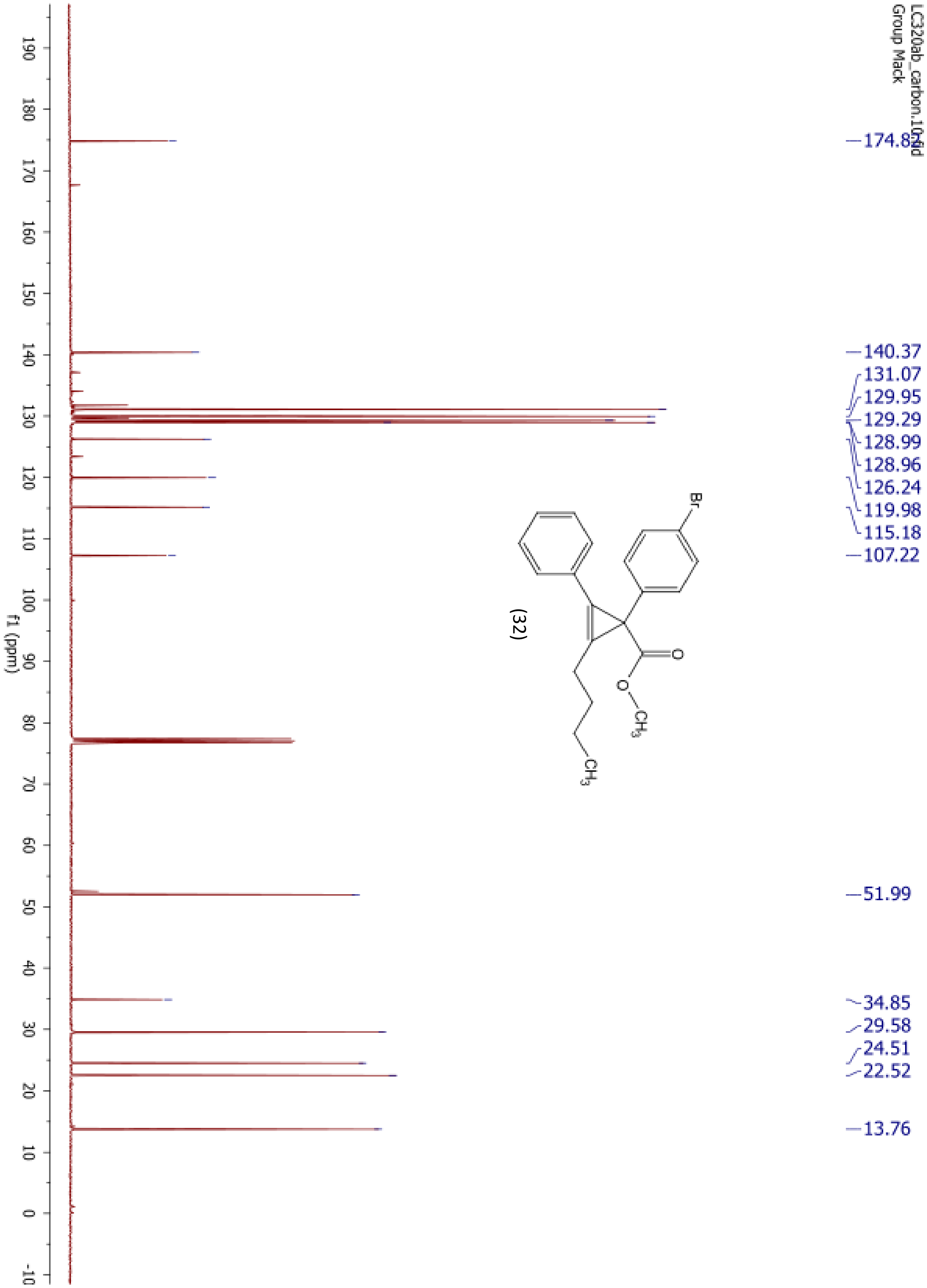
LC320ab_carbon.10.fid
Group Mack
174.82
140.37
131.07
129.95
129.29
128.99
128.96
126.24
119.98
115.18
107.22
51.99
34.85
29.58
24.51
22.52
13.76
f1 (ppm)
Br
O
O
CH3
CH3
(32)

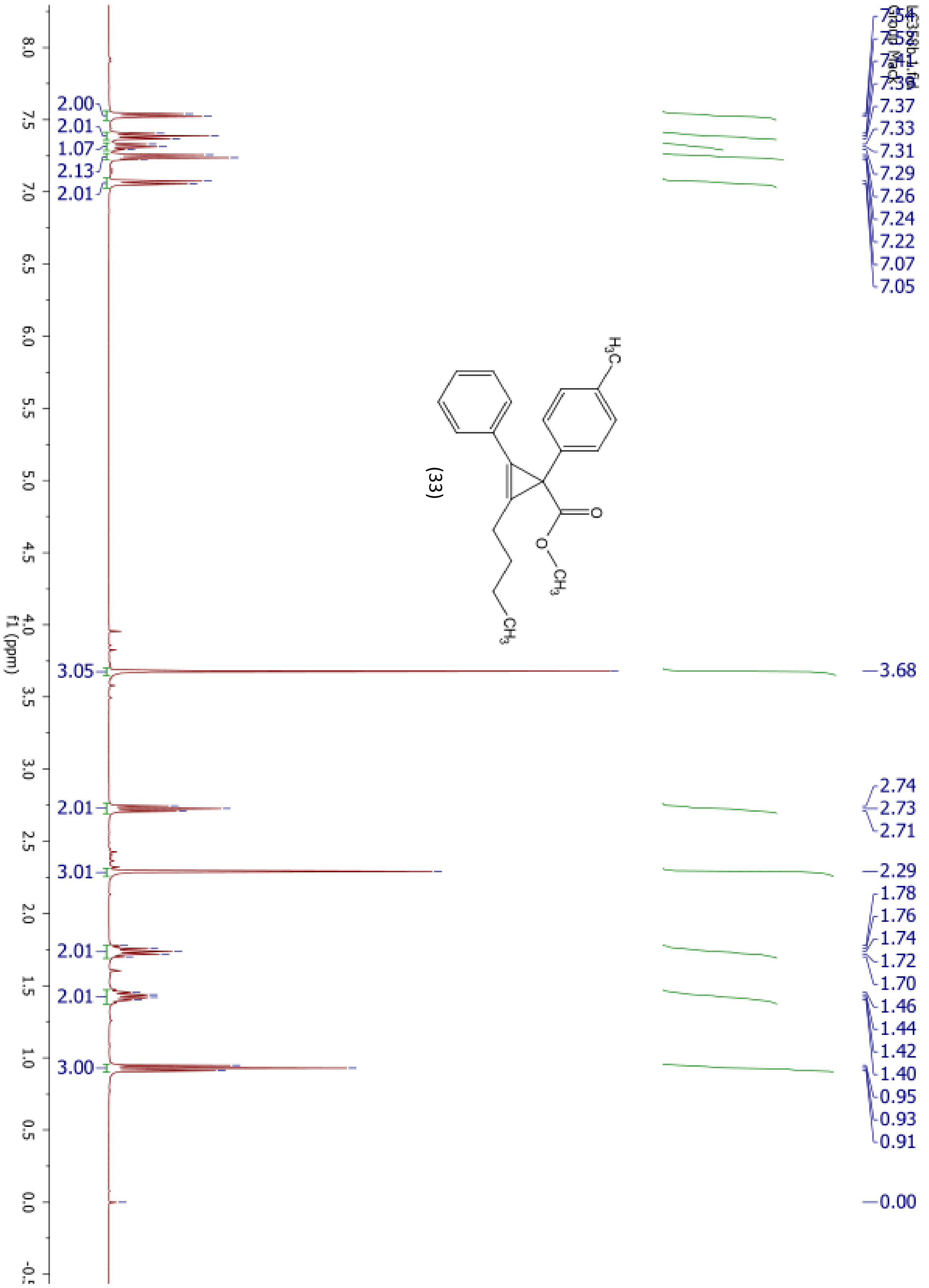

(33)
7.54
7.52
7.41
7.39
7.37
7.33
7.31
7.29
7.26
7.24
7.22
7.07
7.05
3.68
2.74
2.73
2.71
2.29
1.78
1.76
1.74
1.72
1.70
1.46
1.44
1.42
1.40
0.95
0.93
0.91
0.00
2.00
2.01
1.07
2.13
2.01
3.05
2.01
3.01
2.01
2.01
3.00
f1 (ppm)

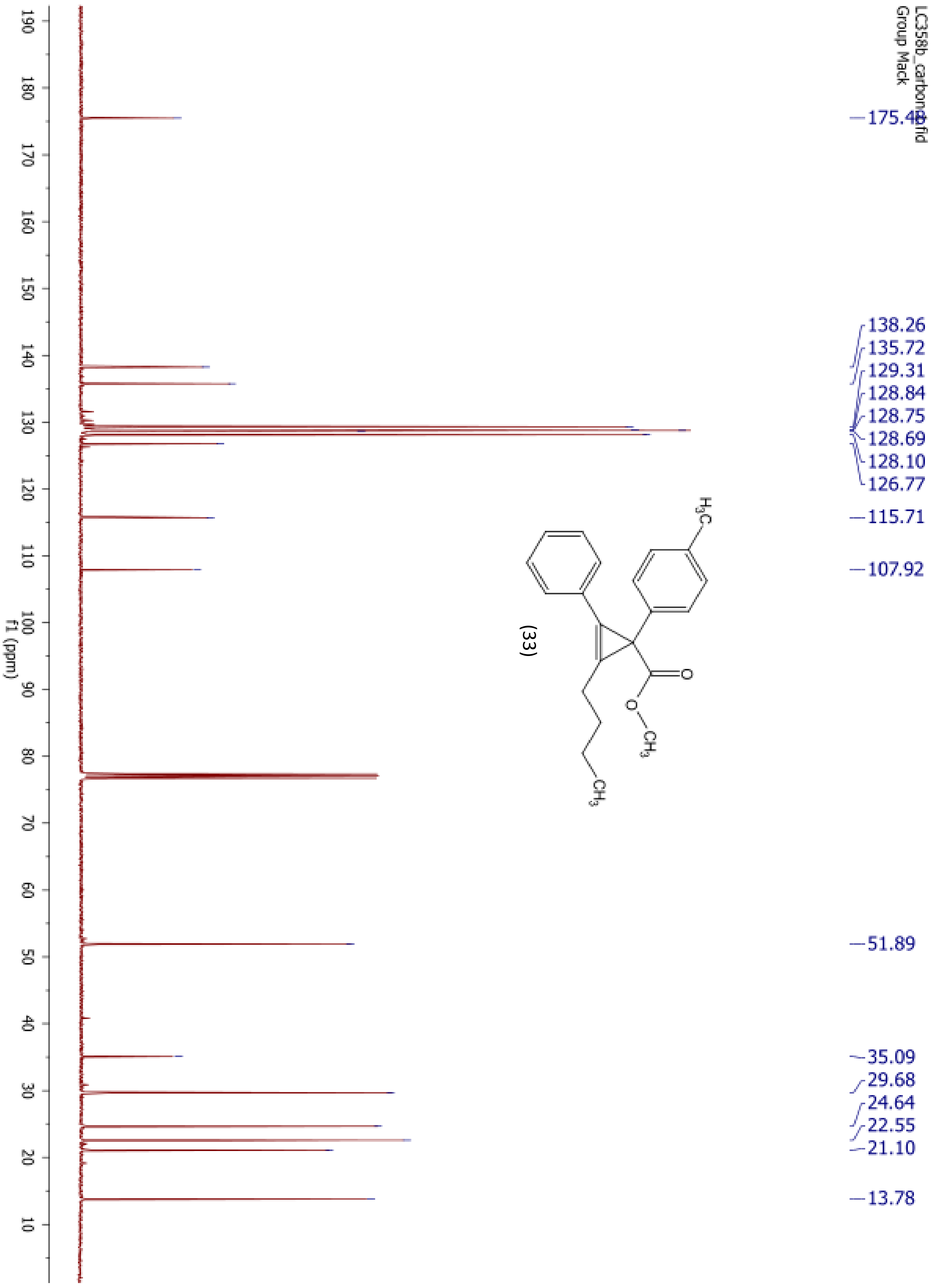
LC358b_carbon.fid
Group Mack
175.48
138.26
135.72
129.31
128.84
128.75
128.69
128.10
126.77
115.71
107.92
51.89
35.09
29.68
24.64
22.55
21.10
13.78
(33)
f1 (ppm)

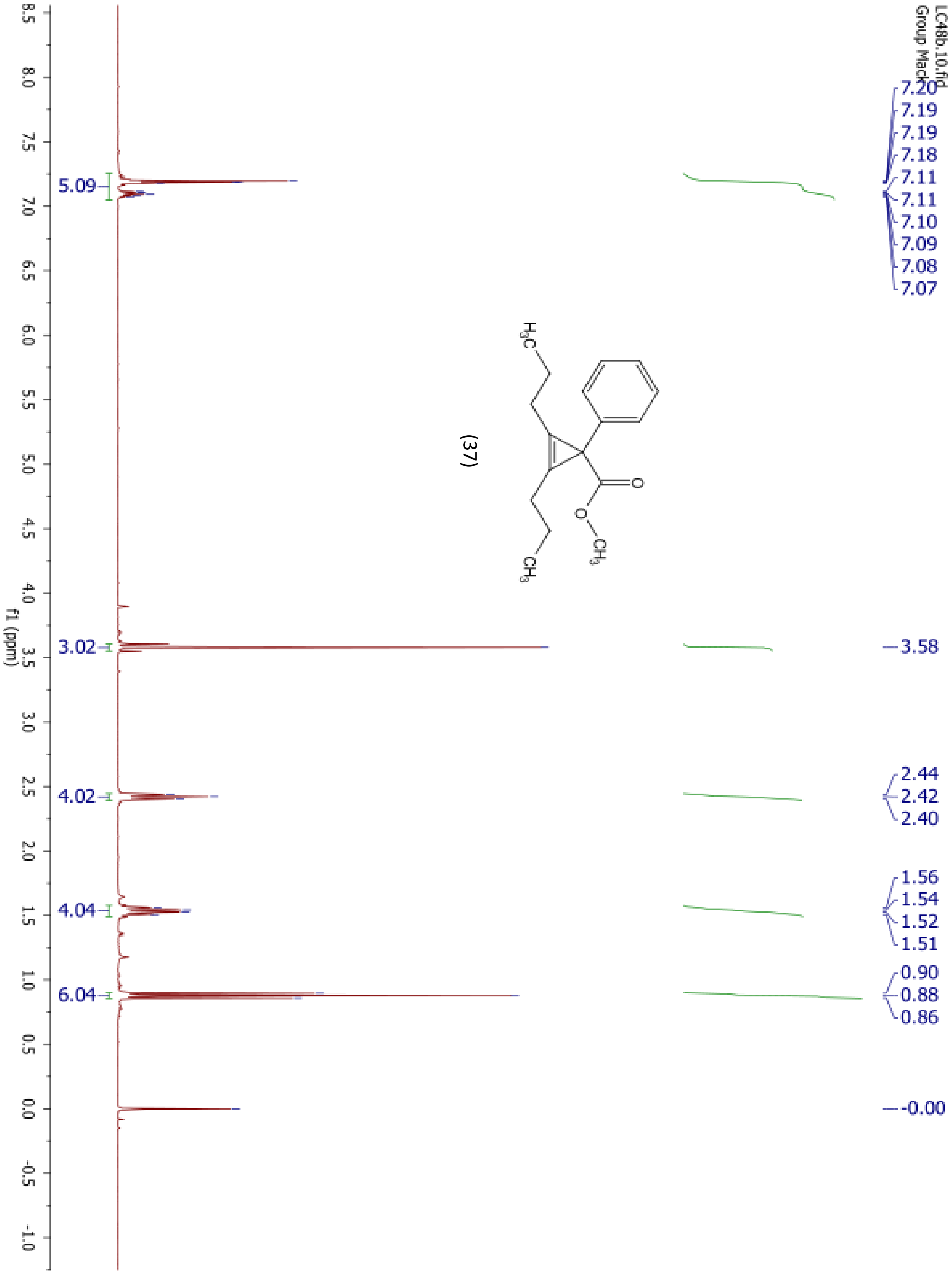
LC48b.10.fid
Group Mack
7.20
7.19
7.19
7.18
7.11
7.11
7.10
7.09
7.08
7.07
3.58
2.44
2.42
2.40
1.56
1.54
1.52
1.51
0.90
0.88
0.86
-0.00
5.09
3.02
4.02
4.04
6.04
(37)
O
O
CH3
H3C
CH3
f1 (ppm)
8.5
8.0
7.5
7.0
6.5
6.0
5.5
5.0
4.5
4.0
3.5
3.0
2.5
2.0
1.5
1.0
0.5
0.0
-0.5
-1.0

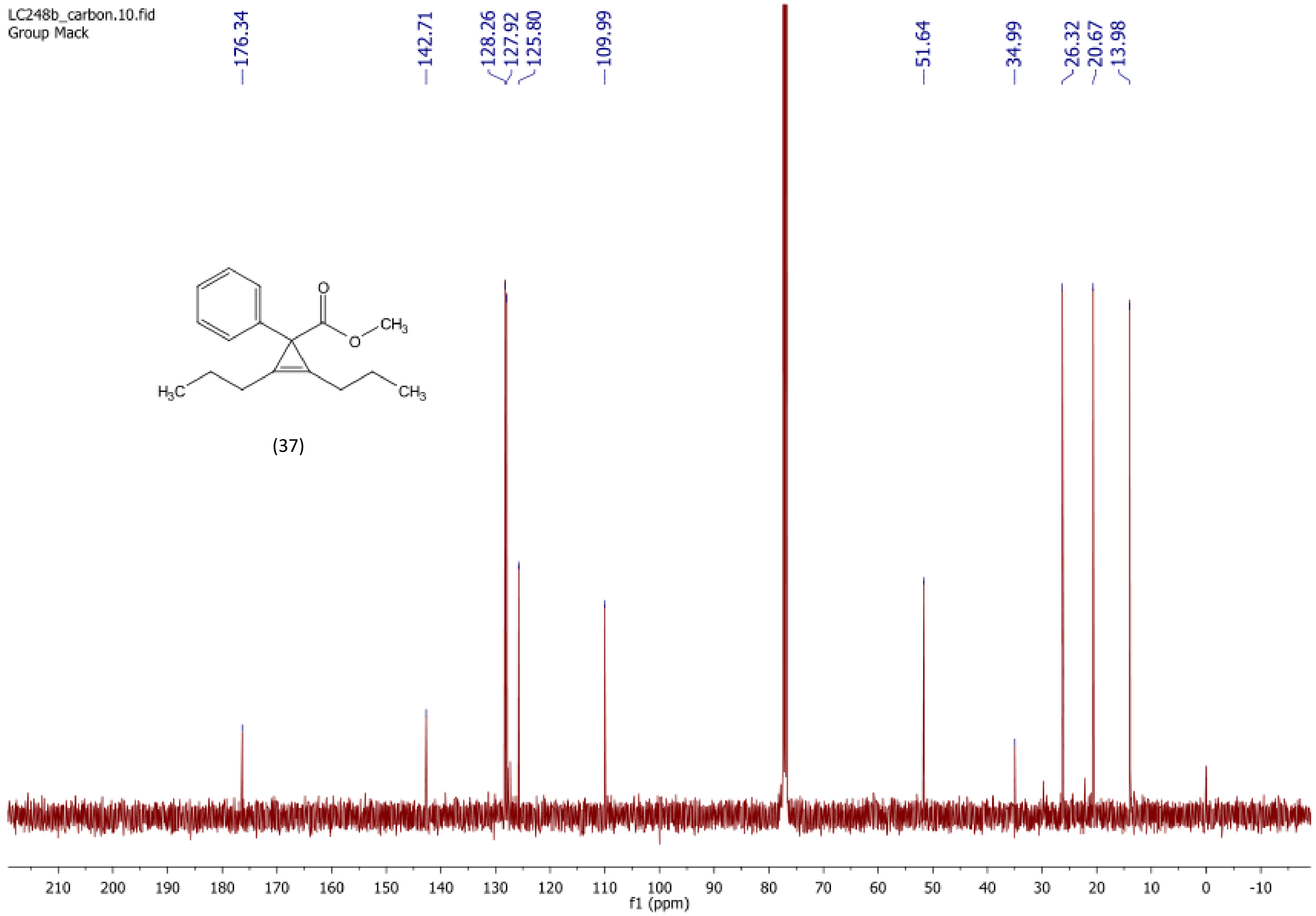
LC248b_carbon.10.fid
Group Mack
176.34
142.71
128.26
127.92
125.80
109.99
51.64
34.99
26.32
20.67
13.98
O
O
CH3
H3C
CH3
(37)
210 200 190 180 170 160 150 140 130 120 110 100 90 80 70 60 50 40 30 20 10 0 -10
f1 (ppm)

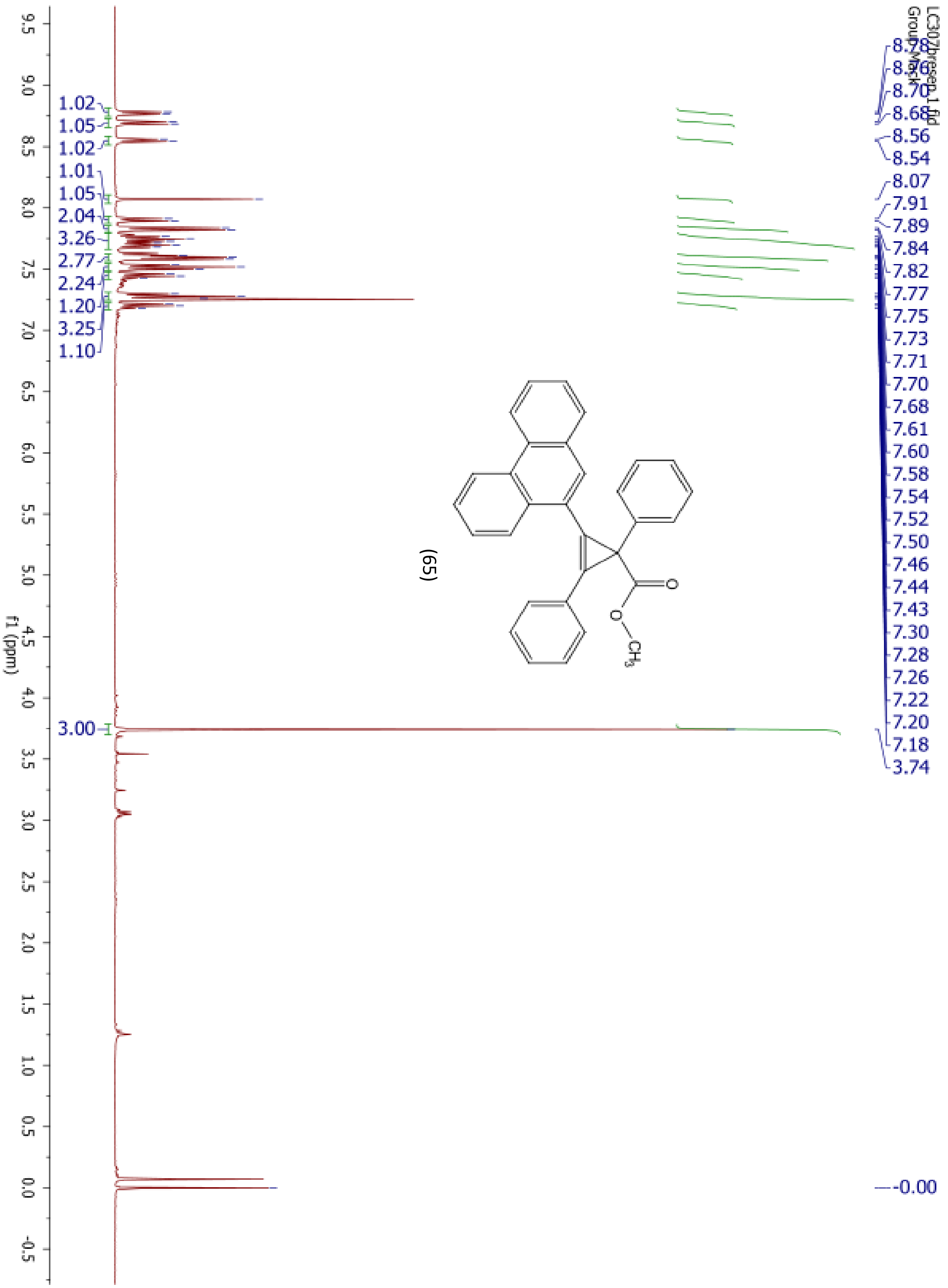

(65)
f1 (ppm)
1.02
1.05
1.02
1.01
1.05
2.04
3.26
2.77
2.24
1.20
3.25
1.10
3.00
8.56
8.54
8.07
7.91
7.89
7.84
7.82
7.77
7.75
7.73
7.71
7.70
7.68
7.61
7.60
7.58
7.54
7.52
7.50
7.46
7.44
7.43
7.30
7.28
7.26
7.22
7.20
7.18
3.74
-0.00

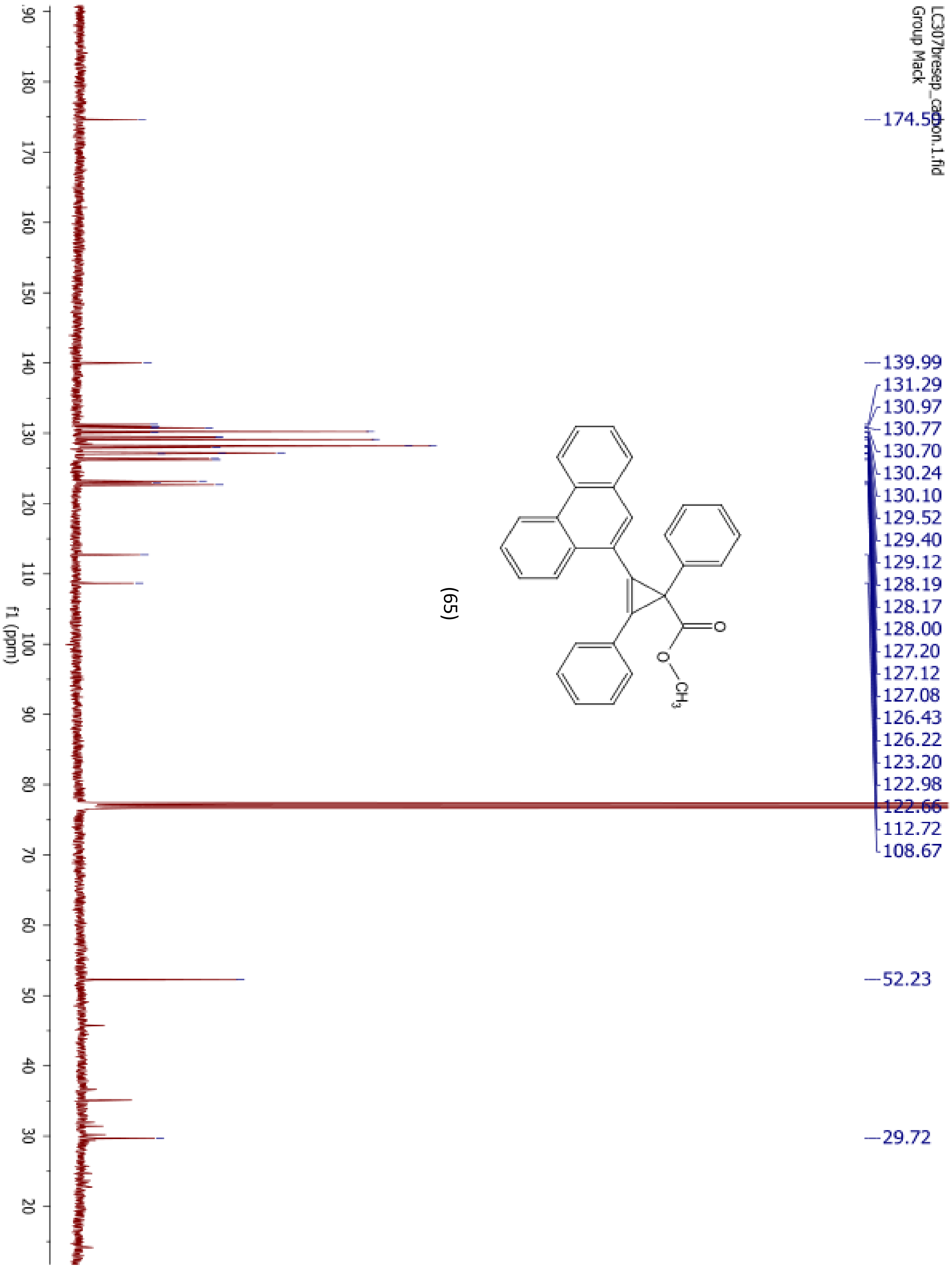
LC307bresep_carbon.1.fid
Group Mack
174.50
139.99
131.29
130.97
130.77
130.70
130.24
130.10
129.52
129.40
129.12
128.19
128.17
128.00
127.20
127.12
127.08
126.43
126.22
123.20
122.98
122.66
112.72
108.67
52.23
29.72
CH_3
f1 (ppm)

(65)

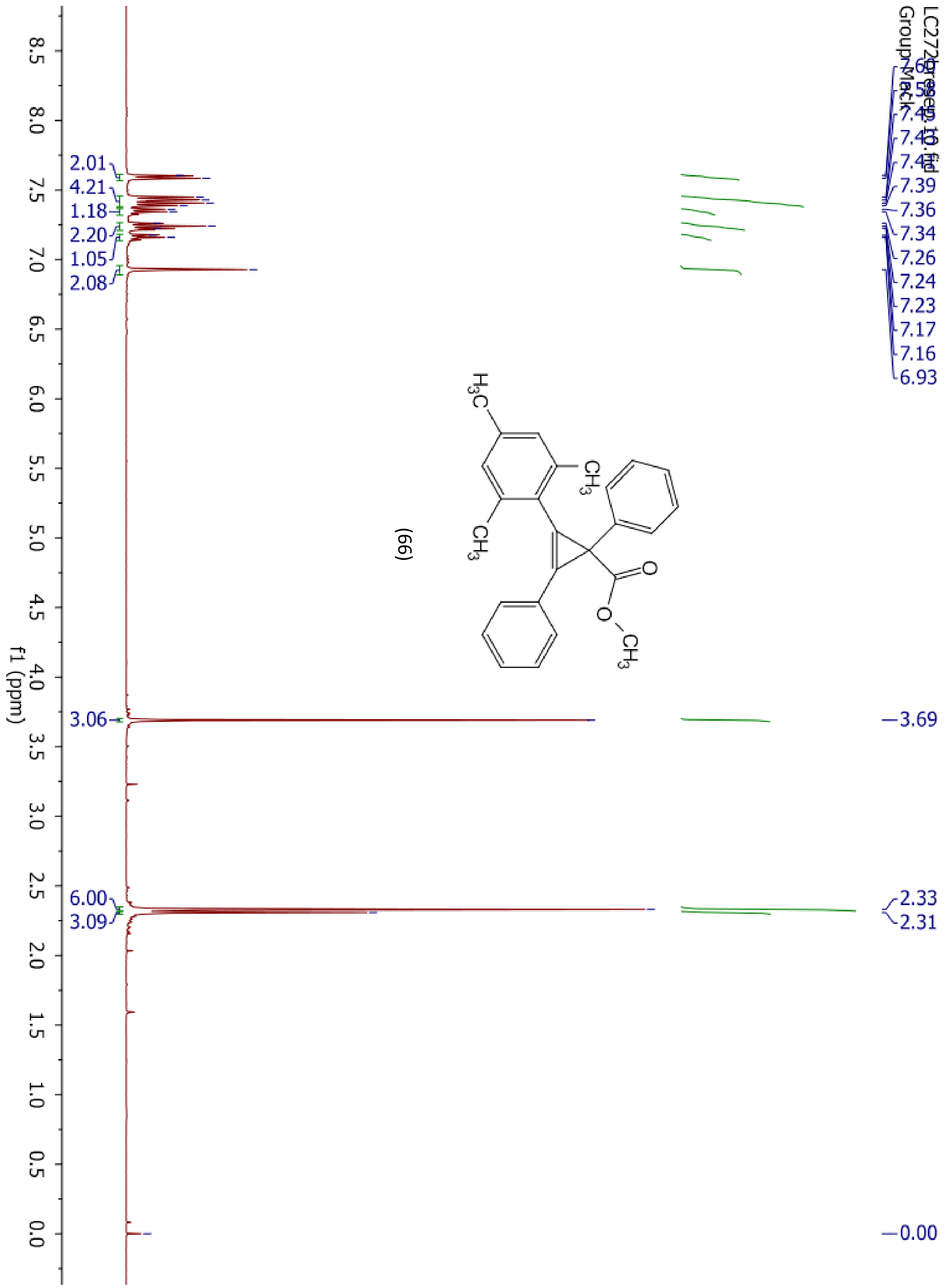

Group Mack
7.39
7.36
7.34
7.26
7.24
7.23
7.17
7.16
6.93
3.69
2.33
2.31
0.00
2.01
4.21
1.18
2.20
1.05
2.08
3.06
6.00
3.09
f1 (ppm)
8.5 8.0 7.5 7.0 6.5 6.0 5.5 5.0 4.5 4.0 3.5 3.0 2.5 2.0 1.5 1.0 0.5 0.0
H3C
CH3
CH3
O
O
CH3
(66)

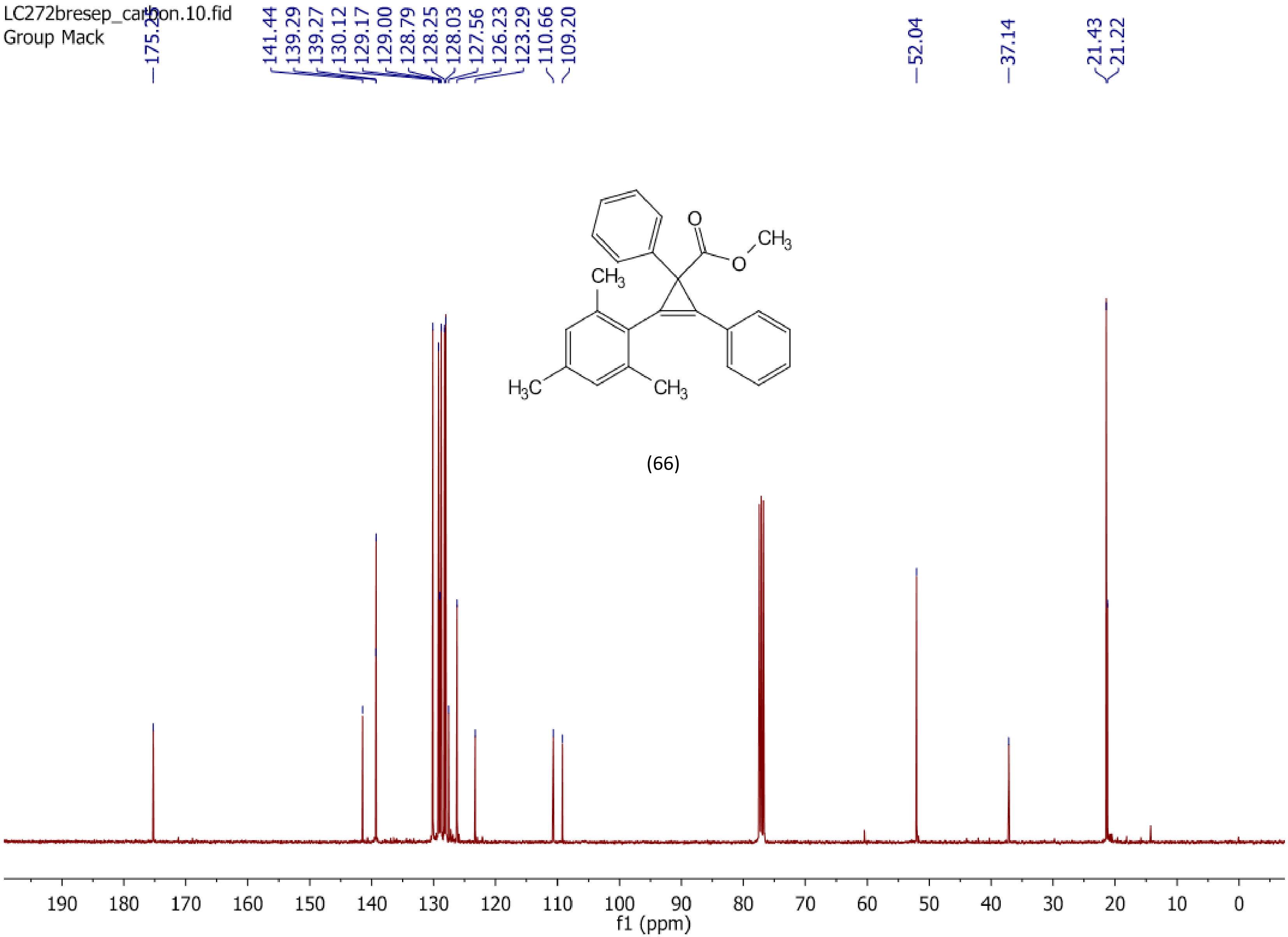
LC272bresep_carbon.10.fid
Group Mack
175.25
141.44
139.29
139.27
130.12
129.17
129.00
128.79
128.25
128.03
127.56
126.23
123.29
110.66
109.20
52.04
37.14
21.43
21.22
CH3
O
O
CH3
H3C
CH3
190
180
170
160
150
140
130
120
110
100
90
80
70
60
50
40
30
20
10
0
f1 (ppm)

(66)

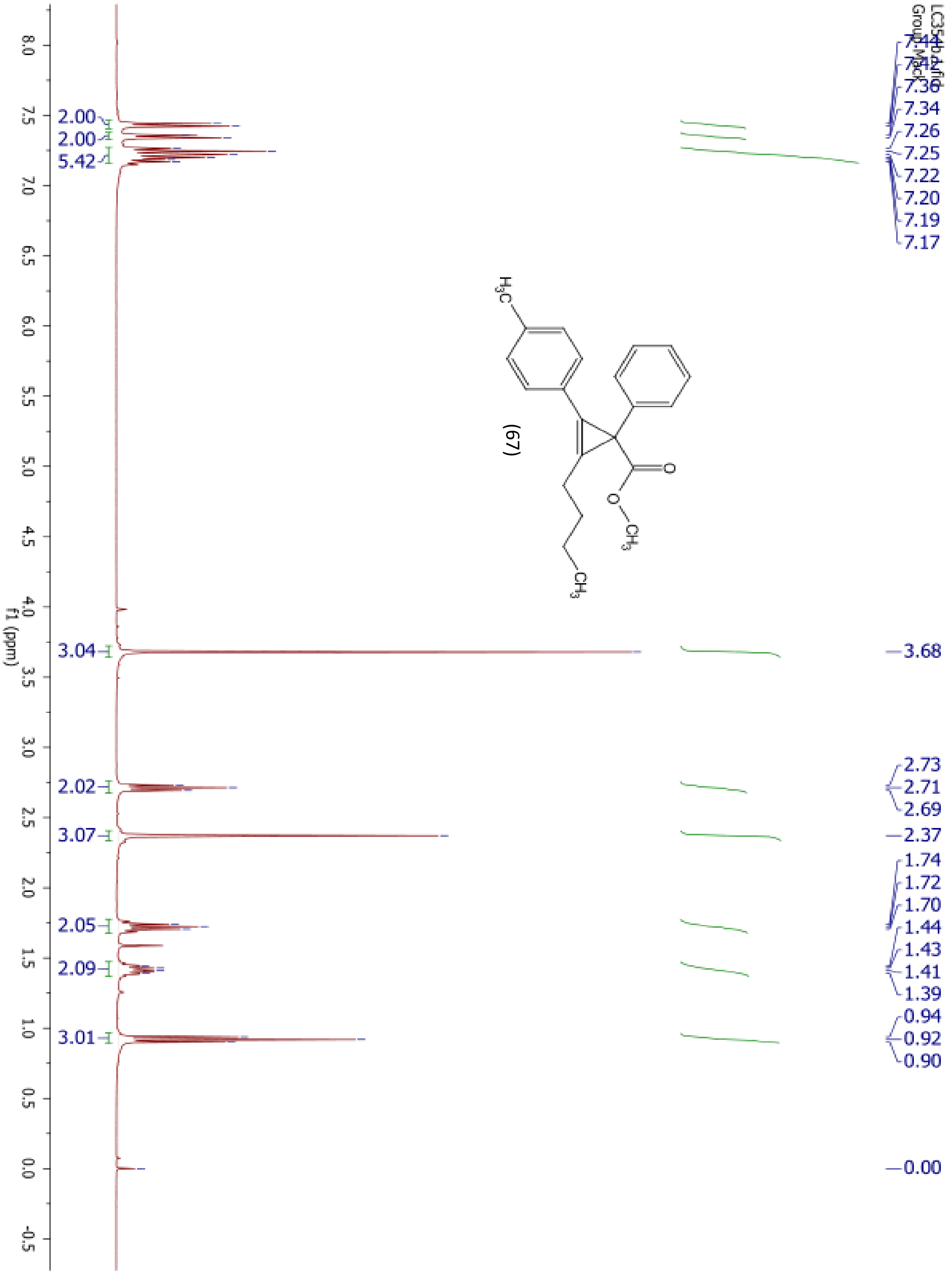
(67)
f1 (ppm)
7.26
7.25
7.22
7.20
7.19
7.17
3.68
2.73
2.71
2.69
2.37
1.74
1.72
1.70
1.44
1.43
1.41
1.39
0.94
0.92
0.90
0.00
2.00
2.00
5.42
3.04
2.02
3.07
2.05
2.09
3.01

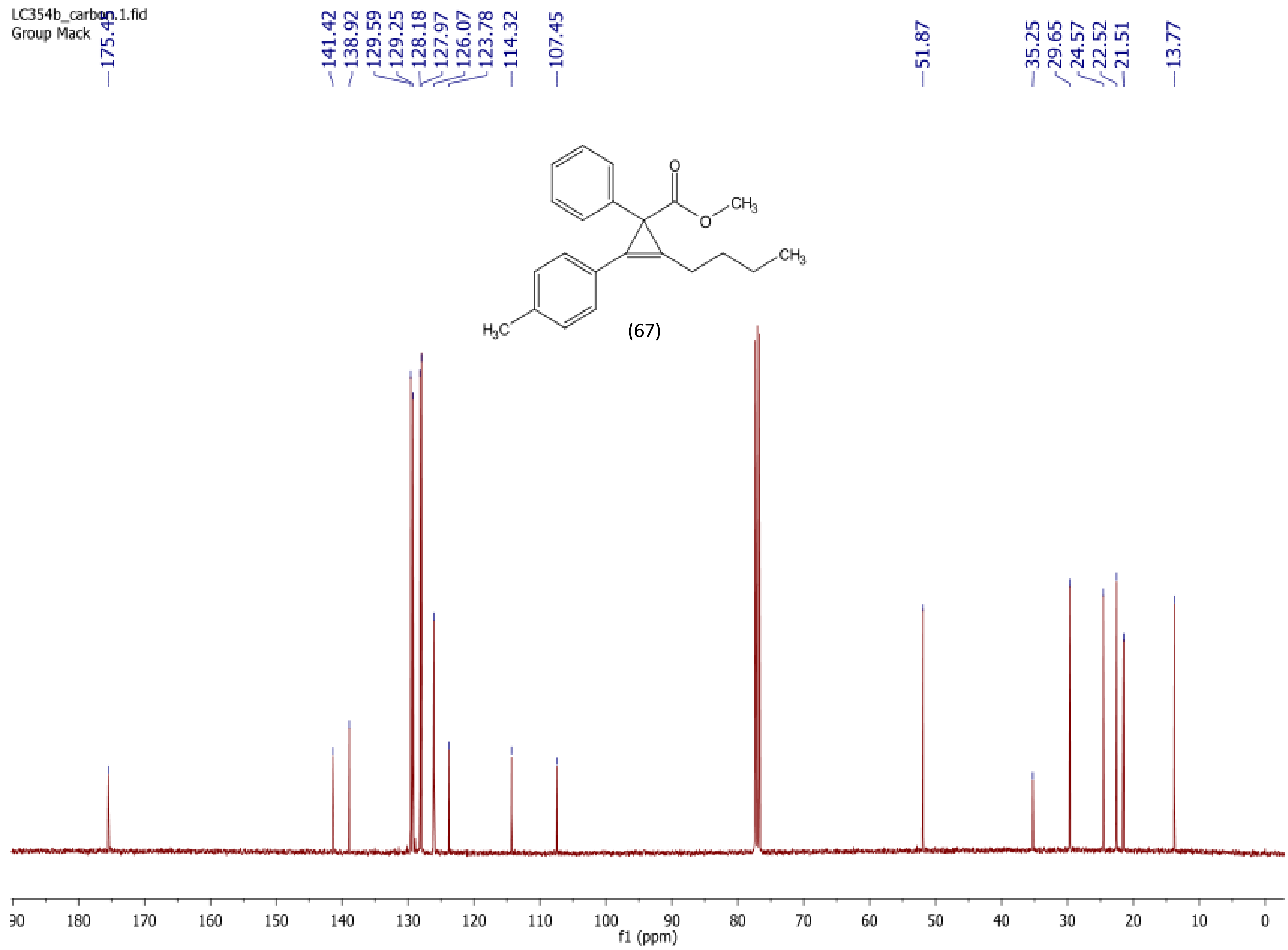
LC354b_carbon.1.fid
Group Mack
175.45
141.42
138.92
129.59
129.25
128.18
127.97
126.07
123.78
114.32
107.45
51.87
35.25
29.65
24.57
22.52
21.51
13.77
O
O
CH3
CH3
H3C
f1 (ppm)

(67)

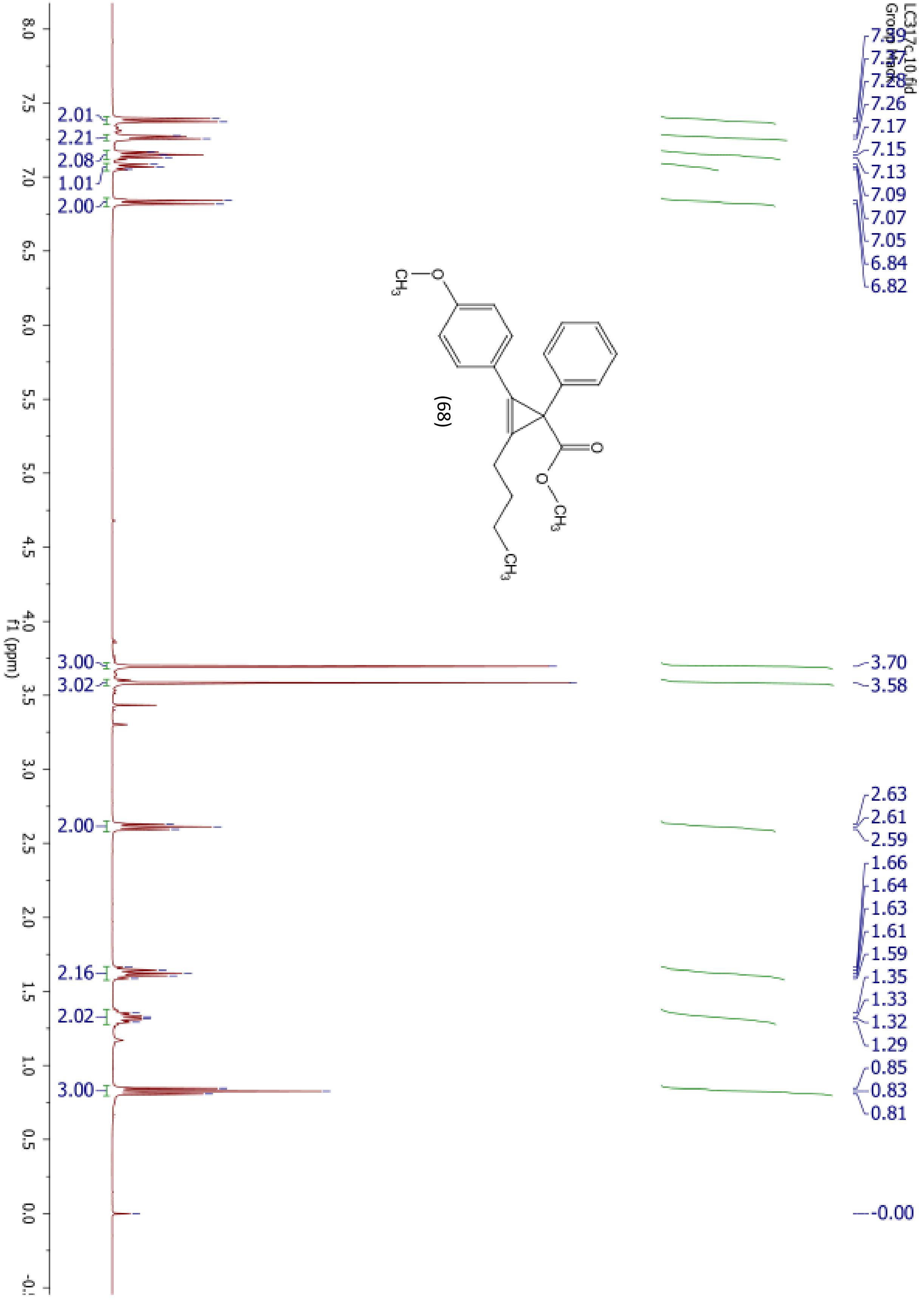

f1 (ppm)
(68)

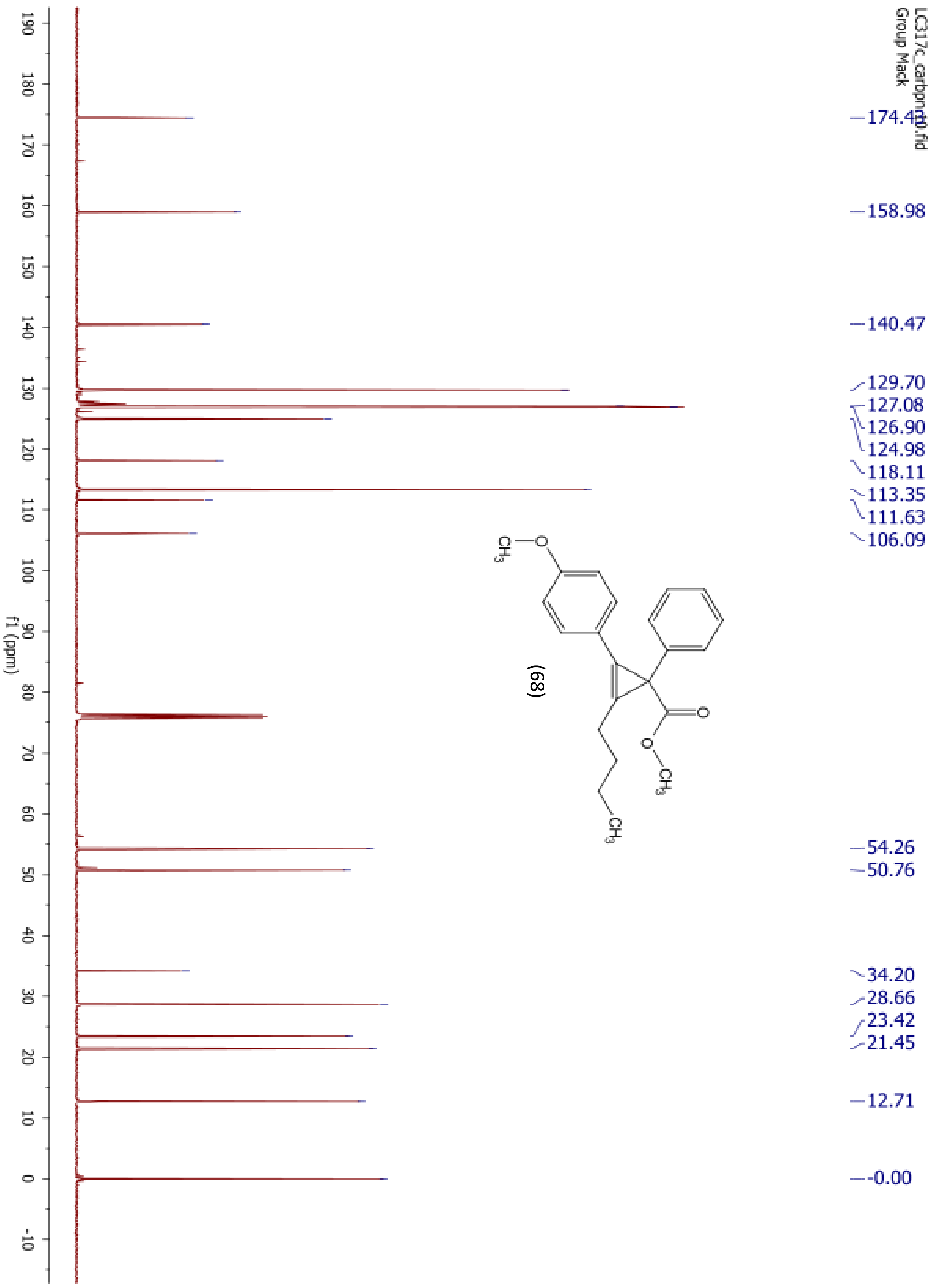
LC317c_carbpnd0.fid
Group Mack
174.40
158.98
140.47
129.70
127.08
126.90
124.98
118.11
113.35
111.63
106.09
54.26
50.76
34.20
28.66
23.42
21.45
12.71
-0.00
(68)
f1 (ppm)

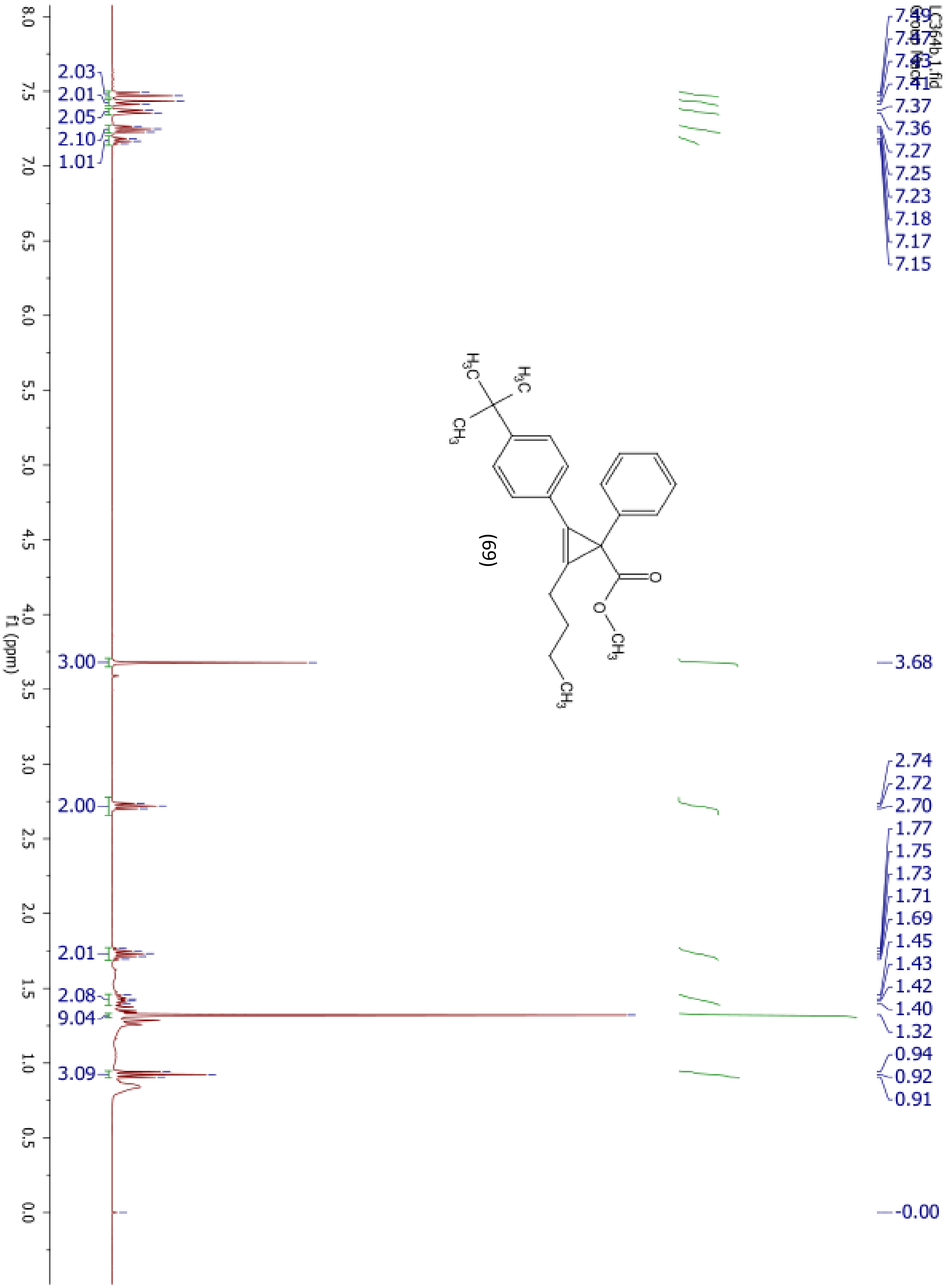
2.03
2.01
2.05
2.10
1.01
3.00
2.00
2.01
2.08
9.04
3.09
7.49
7.47
7.43
7.41
7.37
7.36
7.27
7.25
7.23
7.18
7.17
7.15
3.68
2.74
2.72
2.70
1.77
1.75
1.73
1.71
1.69
1.45
1.43
1.42
1.40
1.32
0.94
0.92
0.91
-0.00
(69)

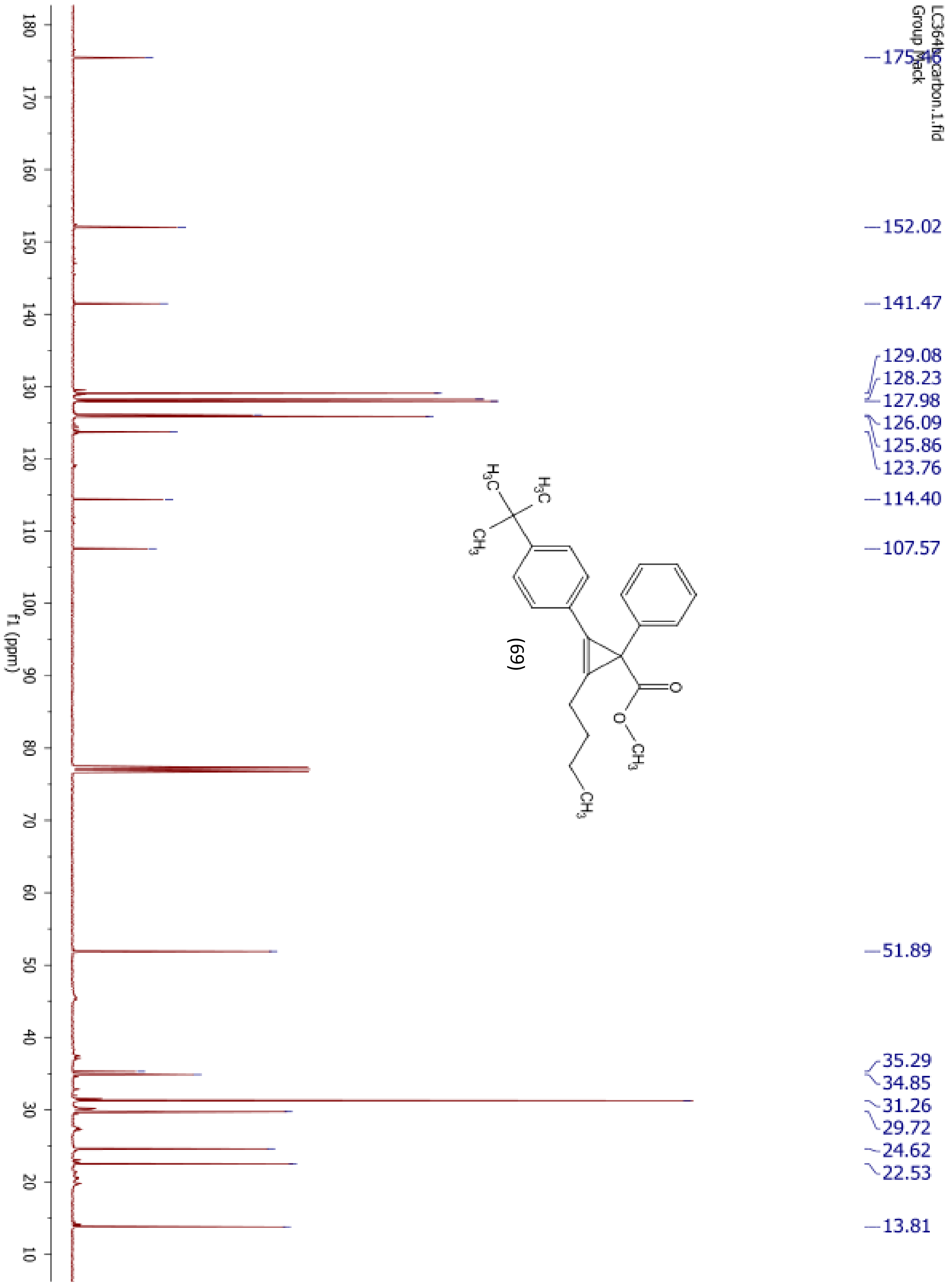
LC364bcarbon.1.fid
Group Mack
152.02
141.47
129.08
128.23
127.98
126.09
125.86
123.76
114.40
107.57
51.89
35.29
34.85
31.26
29.72
24.62
22.53
13.81
(69)
f1 (ppm)

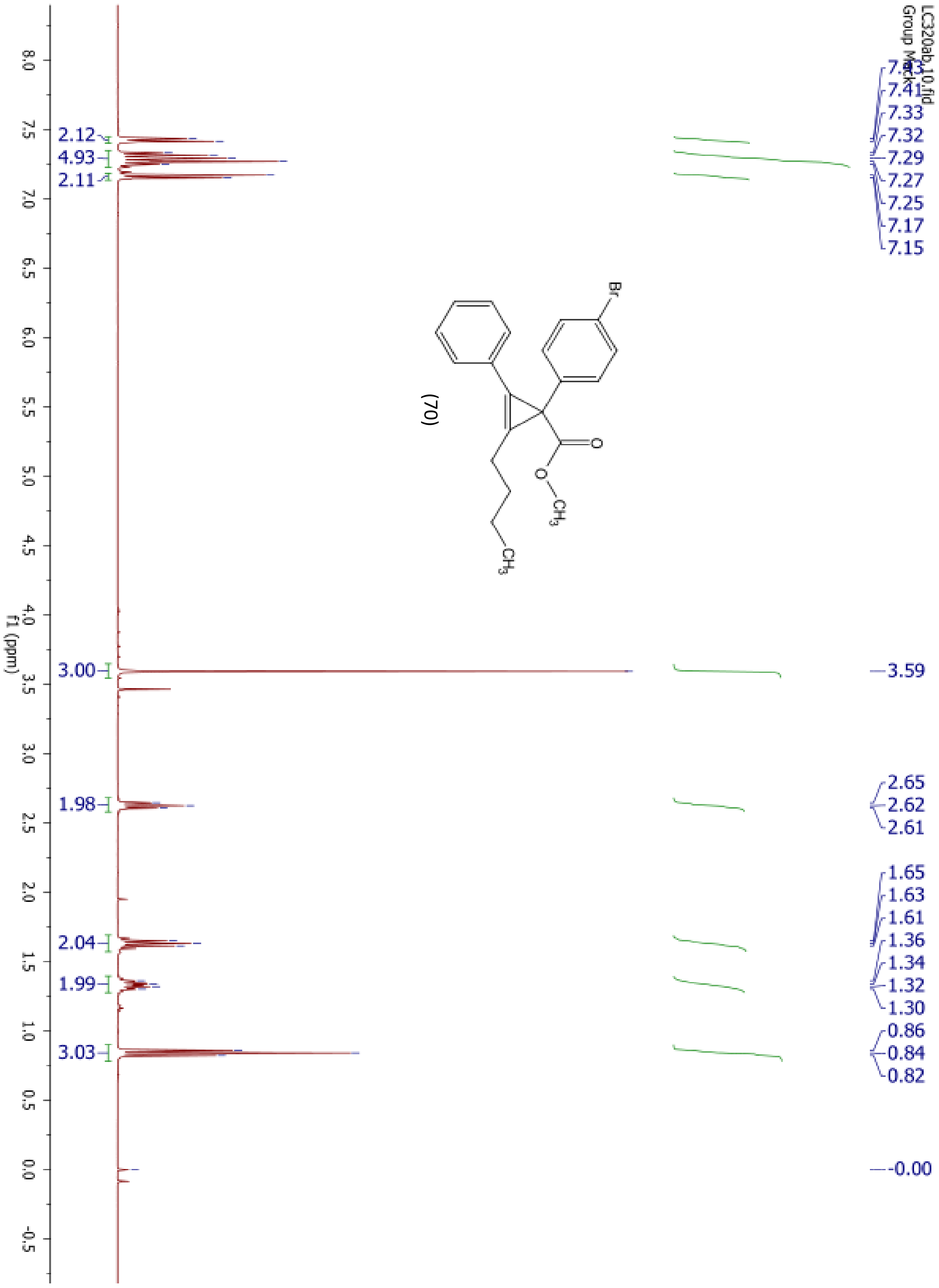
LC320ab_10.fid
Group Mack
7.43
7.41
7.33
7.32
7.29
7.27
7.25
7.17
7.15
3.59
2.65
2.62
2.61
1.65
1.63
1.61
1.36
1.34
1.32
1.30
0.86
0.84
0.82
-0.00
2.12
4.93
2.11
3.00
1.98
2.04
1.99
3.03
Br
O
O
CH_3
CH_3
(70)
f1 (ppm)

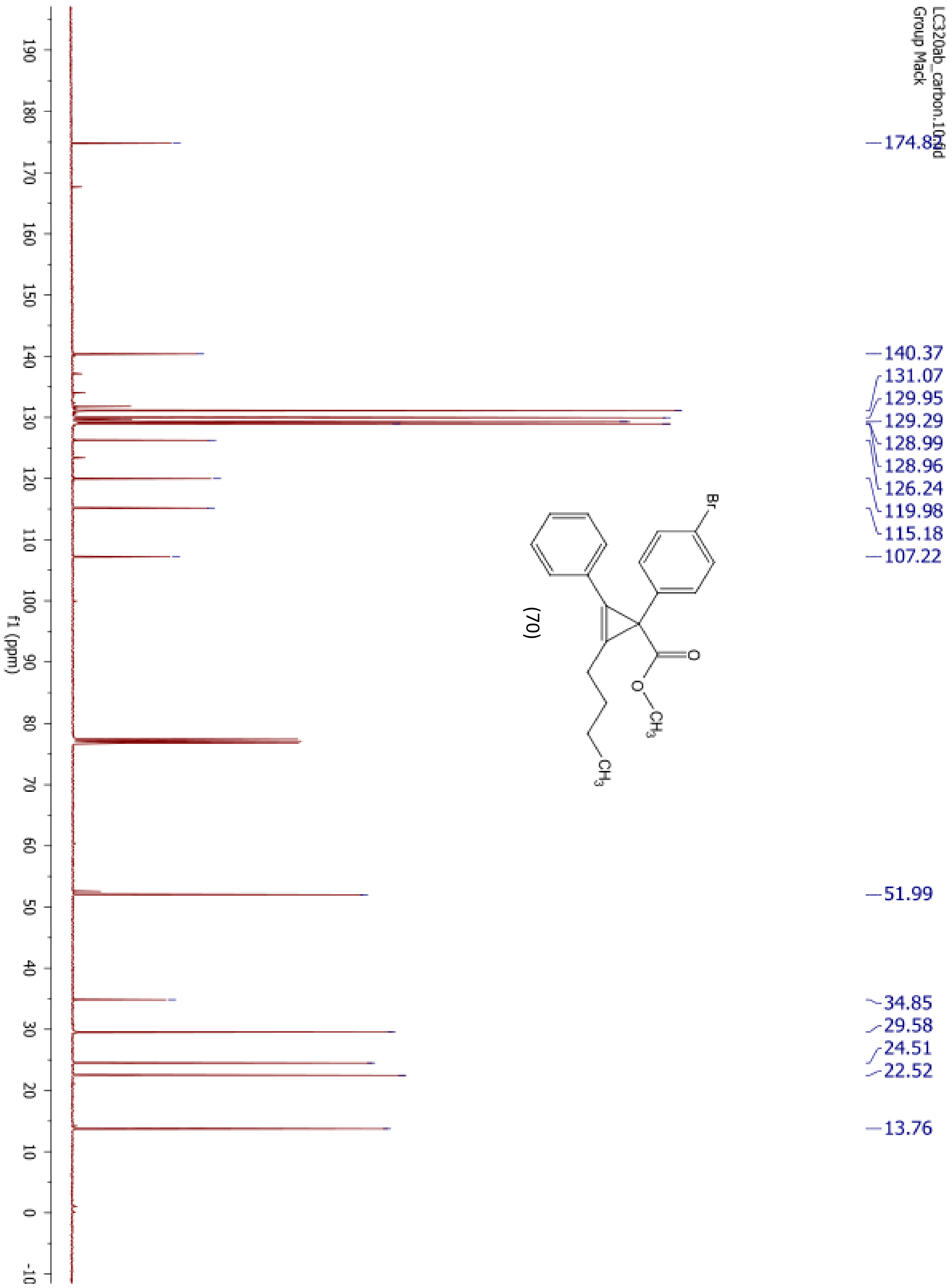

LC320ab_carbon.10.fid
Group Mack
174.82
140.37
131.07
129.95
129.29
128.99
128.96
126.24
119.98
115.18
107.22
51.99
34.85
29.58
24.51
22.52
13.76
(70)
Br
O
CH3
f1 (ppm)
190
180
170
160
150
140
130
120
110
100
90
80
70
60
50
40
30
20
10
0
-10

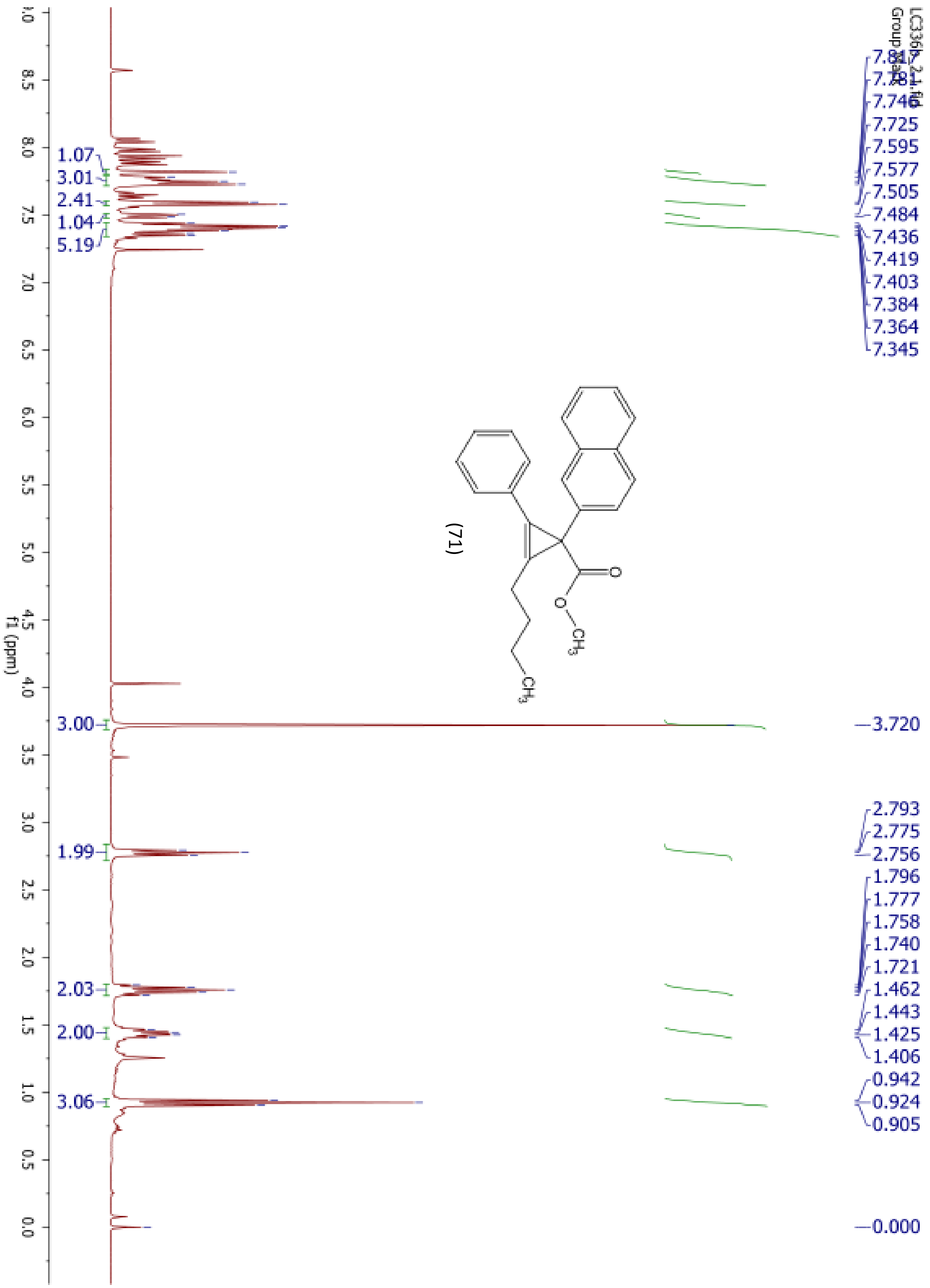

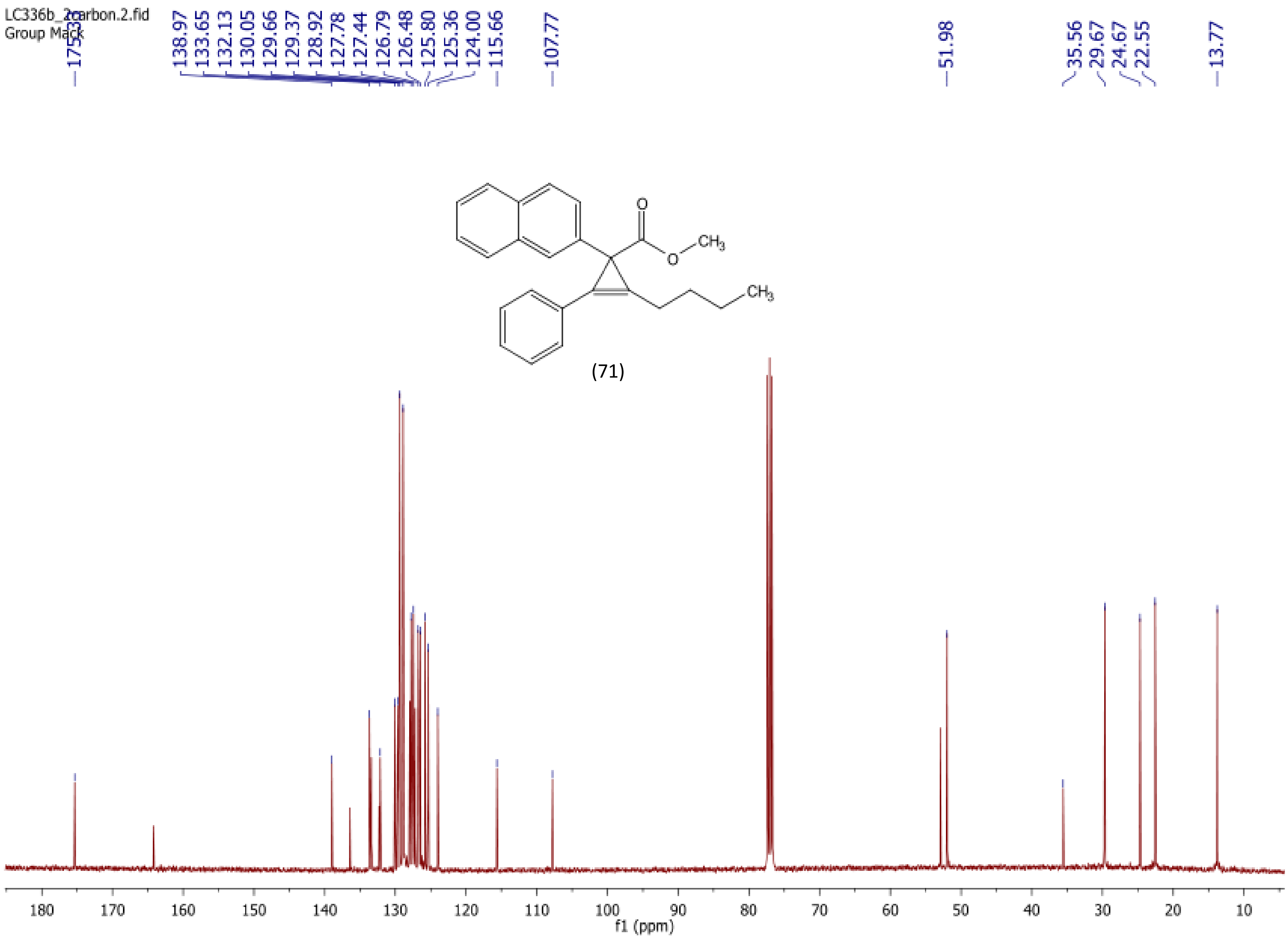
LC336b_2carbon.2.fid
Group Mack
175.30
138.97
133.65
132.13
130.05
129.66
129.37
128.92
127.78
127.44
126.79
126.48
125.80
125.36
124.00
115.66
107.77
51.98
35.56
29.67
24.67
22.55
13.77
O
O
CH3
CH3
(71)
180
170
160
150
140
130
120
110
100
90
80
70
60
50
40
30
20
10
f1 (ppm)

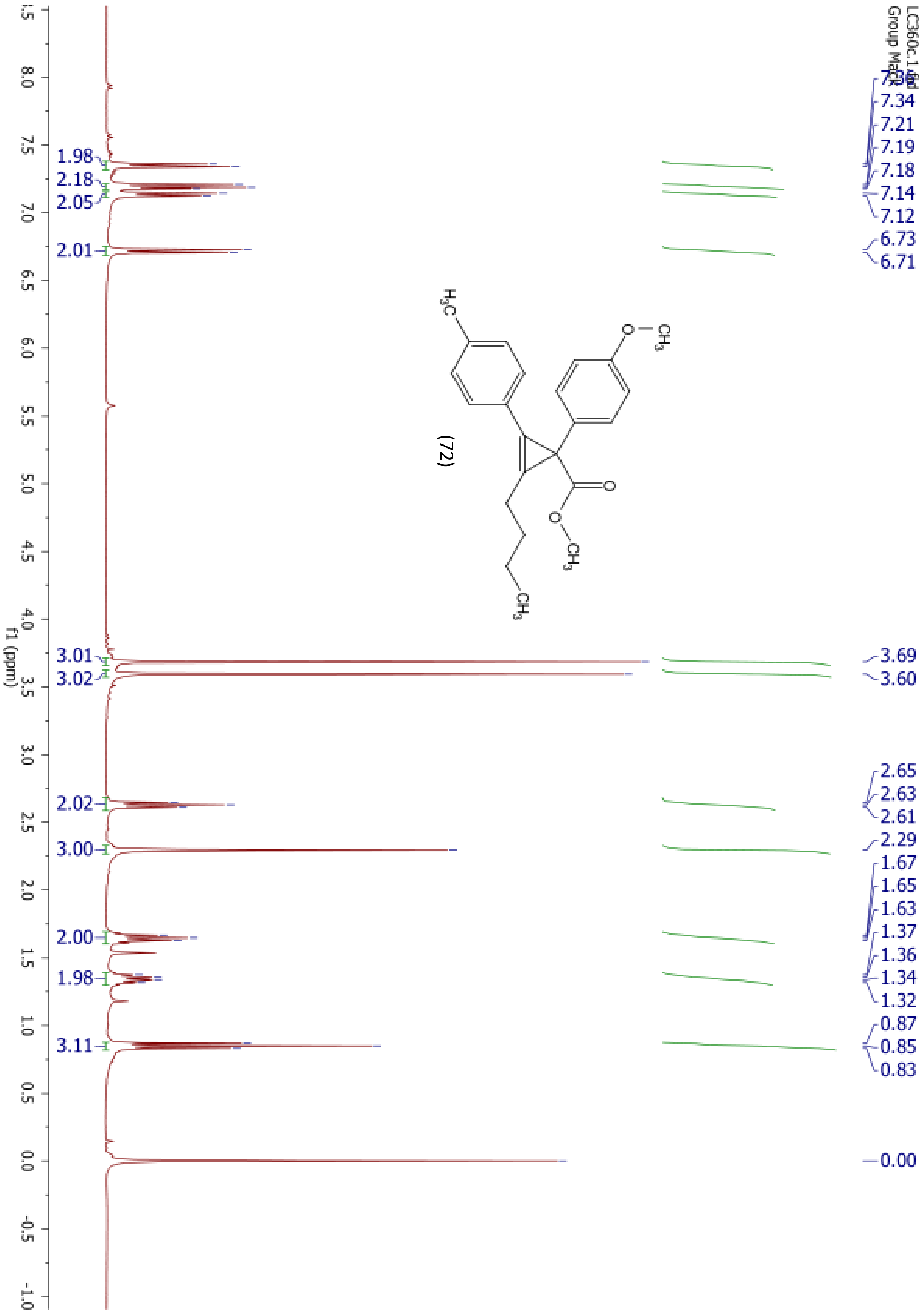

LC360c.1.fid
Group Mack
7.3[illegible]
7.34
7.21
7.19
7.18
7.14
7.12
6.73
6.71
3.69
3.60
2.65
2.63
2.61
2.29
1.67
1.65
1.63
1.37
1.36
1.34
1.32
0.87
0.85
0.83
0.00
1.98
2.18
2.05
2.01
3.01
3.02
2.02
3.00
2.00
1.98
3.11
f1 (ppm)
(72)

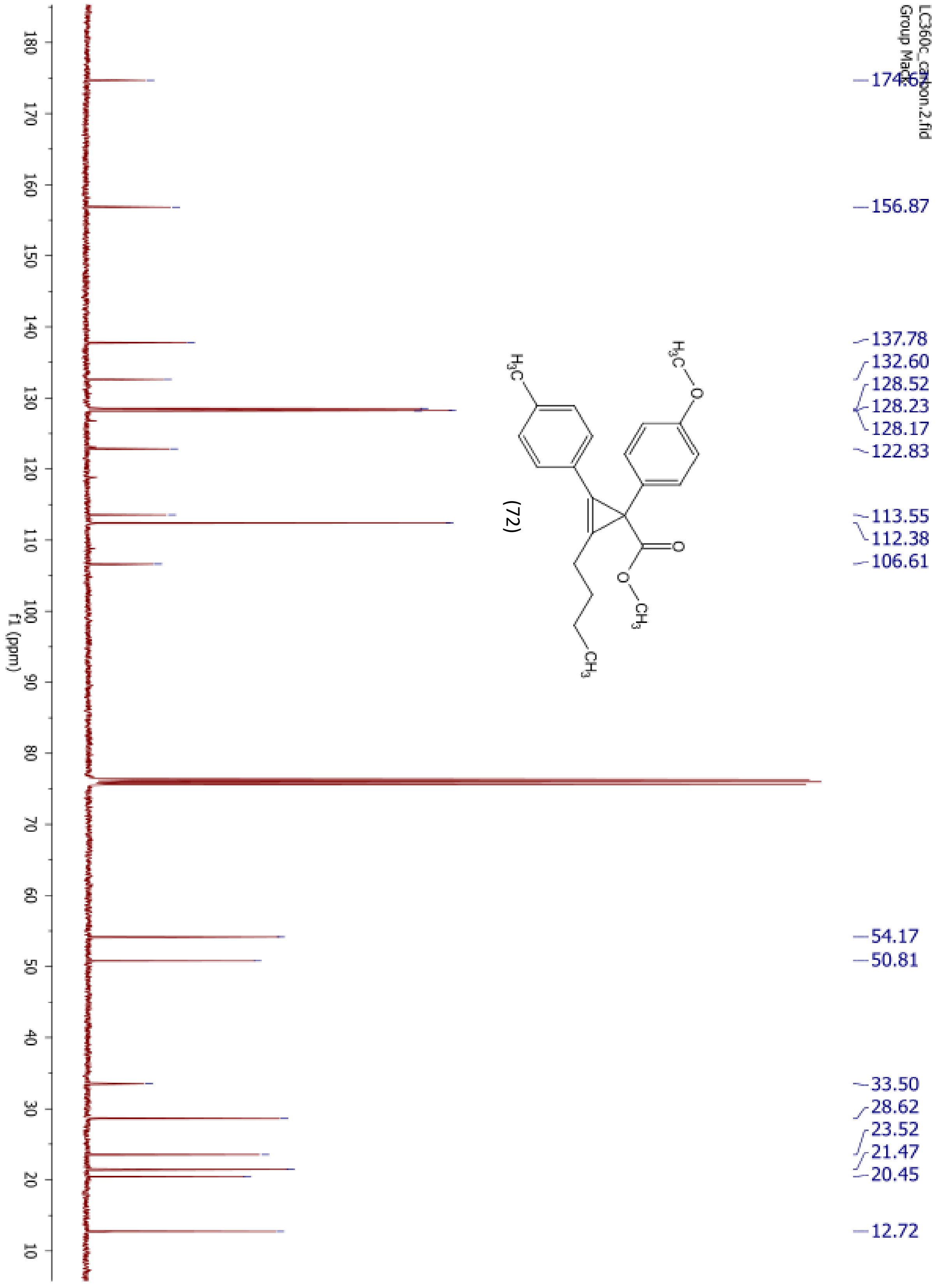
LC360c_carbon.2.fid
Group Mack
174.62
156.87
137.78
132.60
128.52
128.23
128.17
122.83
113.55
112.38
106.61
54.17
50.81
33.50
28.62
23.52
21.47
20.45
12.72
(72)
f1 (ppm)

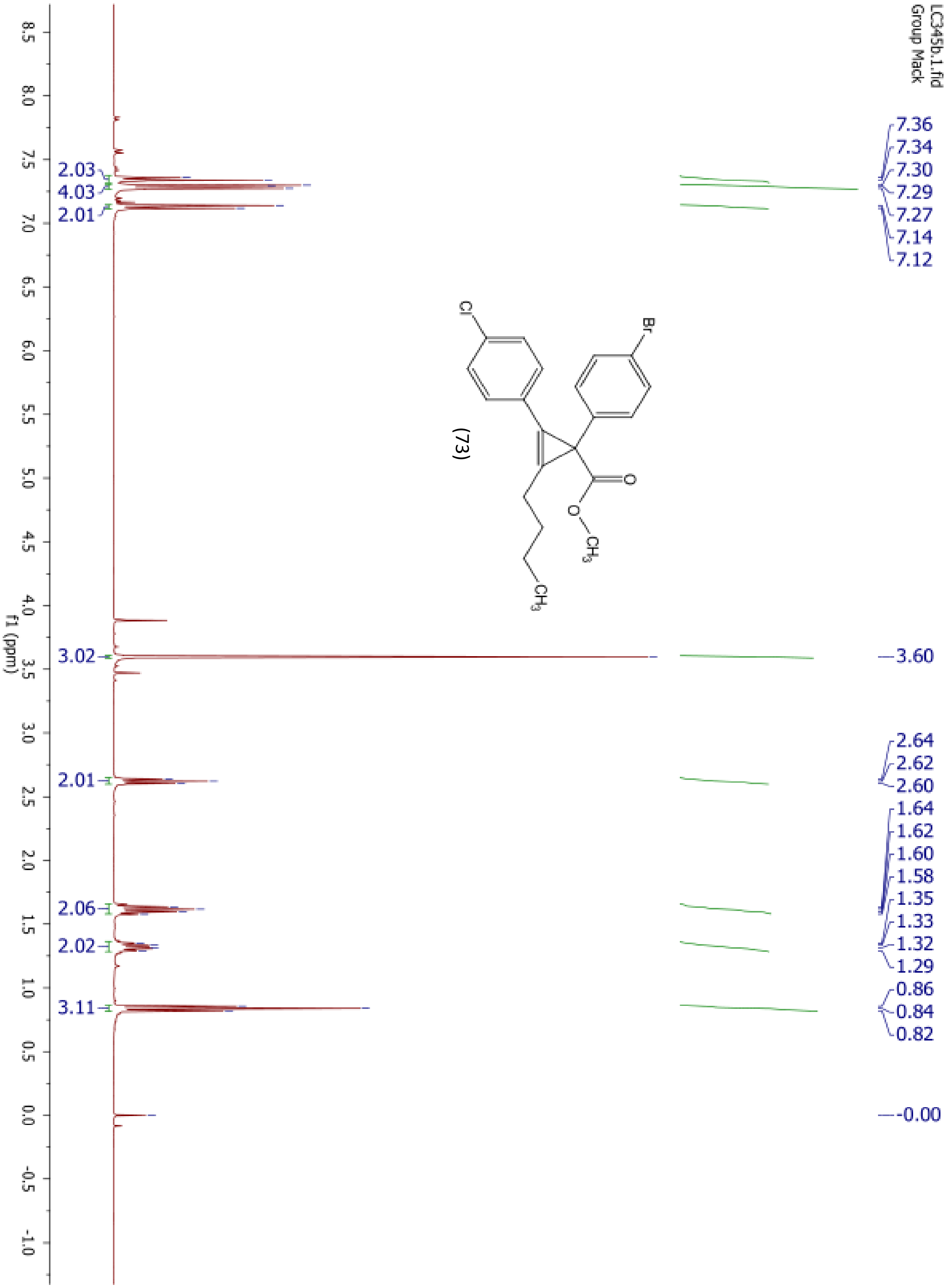
LC345b.1.fid
Group Mack
7.36
7.34
7.30
7.29
7.27
7.14
7.12
3.60
2.64
2.62
2.60
1.64
1.62
1.60
1.58
1.35
1.33
1.32
1.29
0.86
0.84
0.82
-0.00
2.03
4.03
2.01
3.02
2.01
2.06
2.02
3.11
f1 (ppm)
Cl
Br
O
O
CH_3
CH_3
(73)

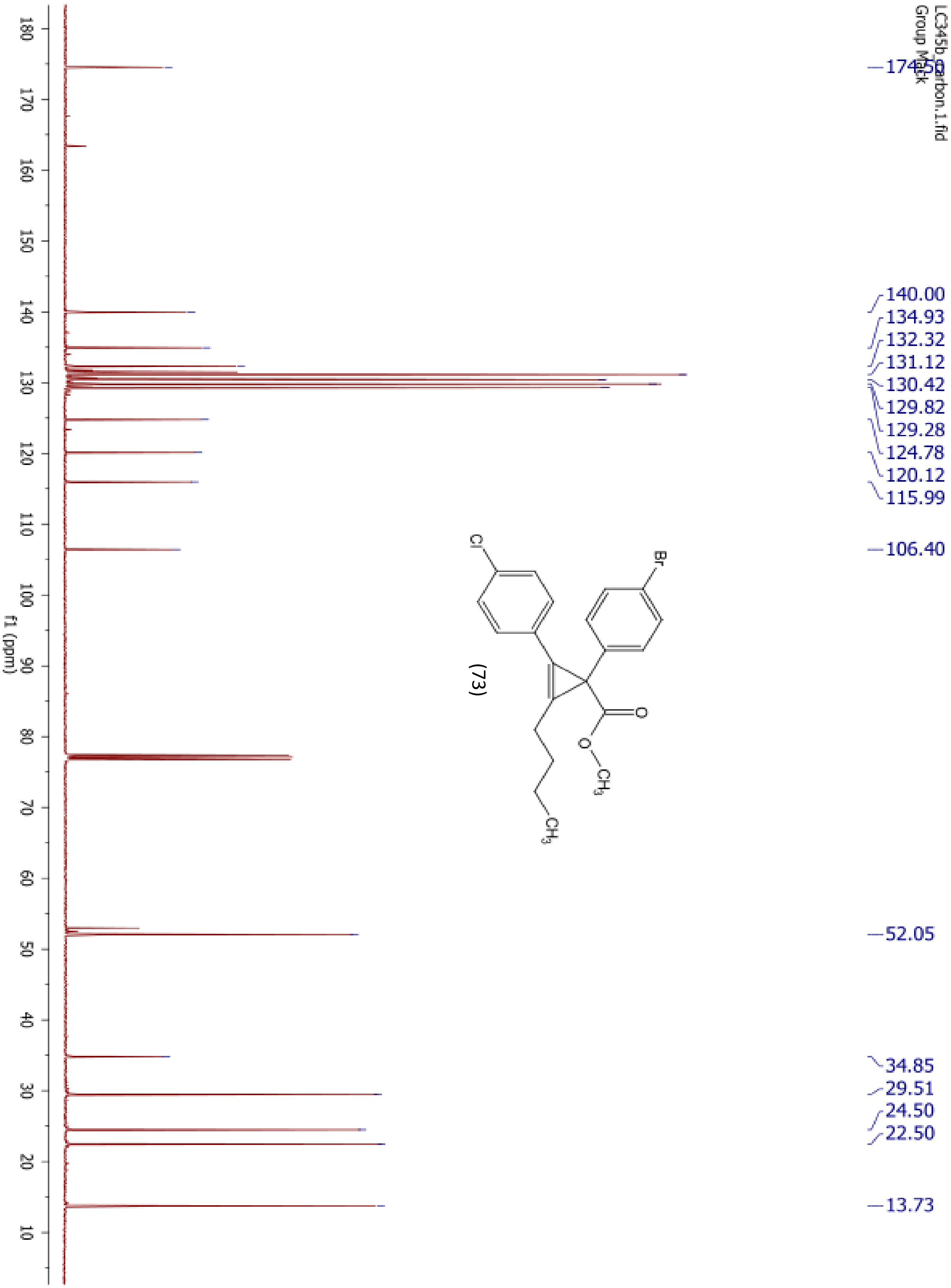
LC345b_carbon.1.fid
174.50
140.00
134.93
132.32
131.12
130.42
129.82
129.28
124.78
120.12
115.99
106.40
52.05
34.85
29.51
24.50
22.50
13.73
f1 (ppm)
Cl
Br
O
CH3
CH3
(73)

References

1. Davies, H. M. L.; Lee, G. H., *Organic Letters* **2004**, *6*, 1233.
2. Briones, J. F.; Davies, H. M. L., *Org. Lett.* **2011**, *13*, 3984.
3. Fordyce, E. A. F.; Wang, Y.; Luebbers, T.; Lam, H. W., *Chem. Commun. (Cambridge, U. K.)* **2008**, 1124.
4. Mueller, P.; Graenicher, C., *Helv. Chim. Acta* **1993**, *76*, 521.
5. Davies, H. M. L.; Venkataramani, C., *Org. Lett.* **2003**, *5*, 1403.
6. Thompson, J. L.; Davies, H. M. L., *J. Am. Chem. Soc.* **2007**, *129*, 6090.
7. Edwin Raja, G. C.; Irudayanathan, F. M.; Kim, H.-S.; Kim, J.; Lee, S., *J. Org. Chem.* **2016**, *81*, 5244.
8. Zhang, M.; Jia, T.; Wang, C. Y.; Walsh, P. J., *J. Am. Chem. Soc.* **2015**, *137*, 10346.
9. Ojha, D. P.; Prabhu, K. R., *Org. Lett.* **2015**, *17*, 18.
10. Chen, M.; Zheng, X.; Li, W.; He, J.; Lei, A., *J. Am. Chem. Soc.* **2010**, *132*, 4101.
11. Bizier, N. P.; Wackerly, J. W.; Braunstein, E. D.; Zhang, M.; Nodder, S. T.; Carlin, S. M.; Katz, J. L., *J. Org. Chem.* **2013**, *78*, 5987.
12. Molander, G. A.; Katona, B. W.; Machrouhi, F., *J. Org. Chem.* **2002**, *67*, 8416.
13. Qrareya, H.; Protti, S.; Fagnoni, M., *J. Org. Chem.* **2014**, *79*, 11527.
14. Mao, J.; Xie, G.; Wu, M.; Guo, J.; Ji, S., *Adv. Synth. Catal.* **2008**, *350*, 2477.
15. Warner, A. J.; Lawson, J. R.; Fasano, V.; Ingleson, M. J., *Angew. Chem., Int. Ed.* **2015**, *54*, 11245.
16. Thomas, T. J.; Merritt, B. A.; Lemma, B. E.; McKoy, A. M.; Nguyen, T.; Swenson, A. K.; Mills, J. L.; Coleman, M. G., *Org. Biomol. Chem.* **2016**, *14*, 1742.
